# SNP array genomic analysis of matched pairs of brain and liver metastases in primary colorectal cancer

**DOI:** 10.1007/s00432-023-05505-4

**Published:** 2023-11-27

**Authors:** Vivian-Pascal Brandt, Heidrun Holland, Marco Wallenborn, Ronald Koschny, Clara Frydrychowicz, Mandy Richter, Lydia Holland, Ulf Nestler, Caroline Sander

**Affiliations:** 1 Department of Neurosurgery, University Hospital Leipzig, Leipzig, Saxony Germany; 2 Saxonian Incubator for Clinical Translation (SIKT), University of Leipzig, Leipzig, Saxony Germany; 3 Interdisciplinary Endoscopy Center (IEZ), Department of Gastroenterology and Hepatology, University Hospital Heidelberg, Heidelberg, Baden-Wuerttemberg Germany; 4 Paul Flechsig Institute of Neuropathology, University Medicine Leipzig, Leipzig, Saxony Germany

**Keywords:** Colorectal cancer, Liver metastasis, Brain metastasis, SNP array, Chromosomal aberration, Cn-LOH

## Abstract

**Purpose:**

Brain metastasis formation is a rare and late event in colorectal cancer (CRC) patients and associated with poor survival. In contrast to other metastatic sites, the knowledge on chromosomal aberrations in brain metastases is very limited.

**Methods:**

Therefore, we carried out single nucleotide polymorphism (SNP) array analyses on matched primary CRC and brain metastases of four patients as well as on liver metastases of three patients.

**Results:**

Brain metastases showed more chromosomal aberrations than primary tumors or liver metastases. Commonly occurring aberrations were gain of 8q11.1-q24.3 (primary CRC), gain of 13q12.13-q12.3 (liver metastases), and gain of 20q11.1-q13.33 (brain metastases). Furthermore, we found one copy-neutral loss of heterozygosity (cn-LOH) region on chromosome 3 in primary CRC, three cn-LOH regions in liver metastases and 23 cn-LOH regions in brain metastases, comprising 26 previously undescribed sites.

**Conclusion:**

The more frequent occurrence of cn-LOHs and subsequently affected genes in brain metastases shed light on the pathophysiology of brain metastasis formation. Further pairwise genetic analyses between primary tumors and their metastases will help to define the role of affected genes in cn-LOH regions.

**Supplementary Information:**

The online version contains supplementary material available at 10.1007/s00432-023-05505-4.

## Introduction

Colorectal cancer (CRC) is one of the most common cancers and the third leading cause of death in the western hemisphere (Ferlay et al. 2015). Each year, approximately 1.4 million cases of colon cancer are diagnosed worldwide (Ferlay et al. 2015; Sefrioui et al. 2017). The standard curative therapy of CRC comprises surgical resection with or without neoadjuvant or adjuvant chemo- or radio chemotherapy. The most frequent localization of primary metastasis is the liver, followed by the lung (Vatandoust et al. 2015; Weiss et al. 1986). In rare cases (0.6–3.2%), brain metastases develop as late events of CRC. Despite aggressive neurosurgical therapy and radiation, the prognosis of brain metastases remains poor. The overall survival of patients with metachronous brain metastases is 2–8 months after diagnosis (Damiens et al. 2012).

In CRC, three processes have been described to influence pathogenesis: chromosomal instability (CIN), microsatellite instability (MSI), and the CpG island methylator phenotype (CIMP) (Jasmine et al. 2012; Mauri et al. 2019; Pino and Chung 2010). Most CRC cases arise through the CIN pathway. Characteristic features are chromosomal rearrangements and copy number variations. Consequences of CIN could be loss of tumor suppressor genes and amplification of oncogenes in the affected chromosomal regions (Jasmine et al. 2012). Recent investigations suggest the accumulation of genetic alterations as an essential step for the tumor evolution of CRC. The timing of specific genetic events could influence the metastatic potential, whereby copy number aberrations already acquire early in tumor development (Golas et al. 2022; Nguyen et al. 2022). In comparison to CIN, MSI is less common and seems to be associated with a better prognosis (Christensen et al. 2016; Guinney et al. 2015; Knösel et al. 2002; Watanabe et al. 2001).

However, colorectal carcinogenesis involves both genetic and epigenetic events, which are still not completely understood (Fearon and Vogelstein 1990; Grady and Carethers 2008; Sefrioui et al. 2017). The genetic profile of CRC comprises several hundred genetic aberrations, such as chromosomal gains on 6p, 7p, 7q, 8q, 13q, 17q and 20q, as well as losses of 4p, 4q, 5q, 8p, 14q, 17p, 18p, 18q, and 20p (Bacolod and Barany 2011; Gutenberg et al. 2010; Sefrioui et al. 2017). In liver metastases of CRC, copy number variations (CNV) have been identified at 1q, 11, 12qter, 17q12-q21, 19, and 22q and deletions have been described at 2q, 5q, 8p, 9p, 10q, and 21q21 (Knosel et al. 2005; Knösel et al. 2002, 2004). Using comparative genomic hybridization (CGH), Gutenberg et al. found significantly more chromosomal aberrations in brain metastases than in the corresponding primary CRC. In detail, gains on 8q, 12p, 12q, and 20p and loss of 5q were identified only in brain metastasis but not in the primary CRC, suggesting a further selection of different genetic alterations during brain metastasis formation (Gutenberg et al. 2010). Recently, accumulation of *Her2* amplification has been described in brain metastases of CRC (Mitra et al. 2019). Furthermore, *KRAS* and *BRAF* mutations were significantly correlated with brain and lung metastasis formation (Liu et al. 2018).

Previous studies, reviewed by Diep et al. and Cardoso et al., showed limitations due to intertumoral heterogeneity when analyzing unmatched sample groups by gene expression or CGH (Cardoso et al. 2007; Diep et al. 2006). Thus, especially intraindividual comparisons of genetic aberrations between the primary CRC tissue and brain metastases are scarce.

In the present study we analyzed the genetic profile of matched pairs of primary CRC, liver metastases and brain metastases. In comparison to CGH, SNP array enables additional determination of copy-neutral loss of heterozygosity (cn-LOH). To the best of our knowledge, this is the first analysis of the genetic profile of CRC and the corresponding liver metastases and brain metastases using SNP array.

## Material and methods

### Patient material

Ethics approval was obtained from the ethics committee of the University of Leipzig (Az.: 005/17-ek). The study confirms the provisions of the Declaration of Helsinki (as revised in Fortaleza, Brazil, in 2013). Informed consent was given by patients and appropriate anonymity considerations were taken into account.

Patients with CRC, liver metastasis and brain metastasis who were diagnosed at the University Hospital of Leipzig (in a time slot of 7 years) were included into our study. Data were collected with regard to patient characteristics including overall survival, localization of the primary colorectal cancer and metastases, their extent and treatment modalities.

**Fig. 1:**
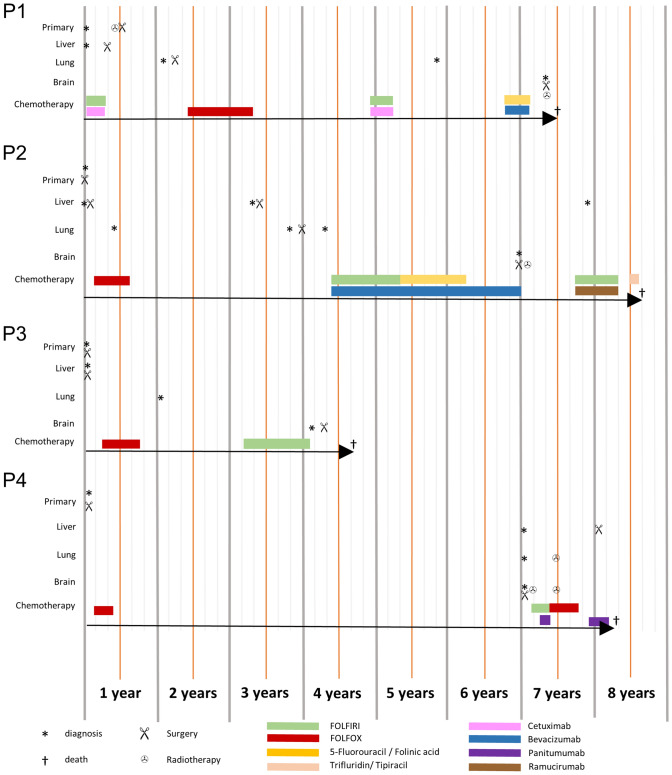
Time course of tumor manifestation and therapy in the four patients (P1-P4)

In total, tissue samples of 4 patients were collected from primary colorectal cancer, hepatic and brain metastases (see Fig. 1).

The average age of the patients was 55 years at the diagnosis of the primary CRC with tumor localization in the rectum (n = 2) and descending colon (n = 2). Surgical resection or biopsy of liver metastasis was performed at a mean of 22.25 months after first diagnosis of CRC. Brain metastases occurred at an average of ~ 64.5 months after first diagnosis. The mean overall survival after first diagnosis was ~ 75.25 months and after resection of brain metastasis ~ 9,5 months. The mean age at the time of death was 60.75 years (Table 1).

**Table 1:** Overview of patient characteristics, tumor progression and treatment for colorectal carcinoma (CRC), liver metastasis and brain metastasis

Characteristics	Patient 1	Patient 2	Patient 3	Patient 4
Gender	m	m	f	m
Age at first diagnosis [years]	60	57	71	30
Localization CRC	Rectum	Rectum	Descending colon	Descending colon
Initial TNM Stage	cT3, cN1, pM1a (hepar)	pT3b, pN2a, pM1 (hepar), L1, V0, Pn1	pT3, pN0, pM1 (hepar), L0, V1	pT3b, pN2b, M0, L1, M1, Pn1
Initial UICC Stage	IV A	IV A	IV A	III C
Grading Latest documented M Stage	G3 brain, liver, vertebra, lung	G2 brain, liver, lung	G3 brain, liver	G2 brain, liver, lung, mediastinum
CRC Subtype	KRAS wildtype ○	KRAS mutation ○	KRAS mutation ○	KRAS mutation, BRAF wildtype, Microsatellite stable
Chemotherapy	Neoadjuvant, adjuvant *	Adjuvant **	Adjuvant ***	Adjuvant ****
Radiation	Yes (rectum + , vertebra + + , brain + + + , + + + +)	Yes (brain + + +)	No	Yes (lung + + , brain + + + , + + + +)
Interval between resection of colorectal cancer and liver metastasis [months]	liver surgery two months before CRC resection	0	0	84
Localization brain metastasis	Left frontoparietal lobe, right temporal lobe, cerebellum	Cerebellum	Right temporal lobe	Right occipital lobe
Volume brain metastasis [cm^3^]	10.6	15.2	27.3	7.0
Interval between diagnosis of colorectal cancer and brain metastasis [months]	76	72	37	72
Total Overall Survival [months]	78	92	44	87
Overall Survival after brain surgery [months]	1	18	5	14

*: neoadjuvant chemotherapy with Cetuximab/FOLFIRI (Irinotecan, Folinic acid, Fluorouracil) before resection of CRC, followed by adjuvant chemotherapy with FOLFOX (Oxaliplatin, Folinic acid, Fluorouracil), Cetuximab/FOLFIRI and Bevacizumab/Ardalan regime (Folinic acid, Fluorouracil) after resection of CRC and liver metastasis, before surgery of brain metastasis

**: adjuvant Chemotherapy with FOLFOX6 after resection of CRC and liver metastasis followed by adjuvant chemotherapy with FOLFIRI, Folinic acid and Fluorouracil after resection of the lung metastasis, before surgery of brain metastasis, followed by chemotherapy with FOLFIRI, Ramucirumab and Trifluridin/ Tipiracil after brain surgery

***: adjuvant chemotherapy with FOLFOX4, followed by FOLFIRI after resection of CRC and liver metastasis, before surgery of brain metastasis

****: Adjuvant chemotherapy with FOLFOX4 after resection of CRC before surgery of brain metastasis, followed by chemotherapy with FOLFIRI, FOLFOX and Panitumumab after brain surgery before resection of the liver metastasis

+ : Neoadjuvant radiation of rectum cancer before CRC surgery

+  + : Radiation therapy without surgical treatment of metastasis

+  +  + : Stereotactic radiation of tumor site after surgery

+  +  +  + : Radiation therapy of the whole brain

○: BRAF status and microsatellite status are not available

#### Isolation and conservation of tumor tissue

For tumor cell isolation, fresh non-necrotic surgical specimens were collected, fixed in formalin, embedded in paraffin and tissue sections were mounted onto glass slides.

#### DNA isolation and molecular karyotyping using SNP array

After pathological investigations, the samples from primary colorectal cancer tissue, liver metastasis and brain metastasis were used for the analyses. The samples were subjected to genome-wide copy number variation (CNV) analysis and assessment of copy number neutral loss of heterozygosity (cn-LOH) chromosomal regions using SNP array (OncoScan® FFPE Assay Kit, ThermoFisher Scientific, Dreieich, Hesse, Germany). Genomic DNA was extracted from tumor samples of colorectal cancer, liver metastasis (except from patient 4 due to technical reasons) and brain metastasis according to the manufacturers’ protocols (QIAamp® DNA FFPE Tissue Handbook, QIAGEN, Hilden, North Rhine-Westphalia, Germany). Paraffin was dissolved in Xylene and the samples were lysed under denaturing conditions using Proteinase K. After binding of DNA to the column, residual contaminants were removed and pure DNA was eluted. Subsequently, we performed OncoScan® array according to the manufacturers’ protocol (UserGuide OncoScan® FFPE Assay Kit, P/N 703175 Rev. 2, Thermo Fisher Scientific, Affymetrix, USA; Waltham). The evaluation was carried out using the software Chromosome Analysis Suite, Version. 3.3.0.139 (Thermo Fisher Scientific Inc., USA, Waltham) and based on the User Guide for Chromosome Analysis Suite 3.3 (ChAS 3.3), Publication Number 702943, Revision 12, available by Thermo Fisher Scientific (Thermo Fisher Scientific Inc., USA, Waltham). The following reference models were used: OncoScan.FFPE.na33.r2.REF_MODEL (as copy number reference) and OncoScan.FFPE.na33.r2.SOM_REF_MODEL (as somatic mutation reference). Pre-processing steps includes the Dual Quantile Normalization followed by Array Data QC Metrics and TuScan Algorithm (see User Guide for Chromosome Analysis Suite 3.3 – Appendix G). According to the literature, we considered chromosomal aberrations ≥ 3 Mb as reliable gain or loss and cn-LOH regions ≥ 5 Mb as representing (segmental) uniparental disomy (UPD) (Beroukhim et al. 2006; Žilina et al. 2015). Subsequent statistical analyses were performed using Prism 6 (GraphPad Software, La Jolla, USA). A detailed workflow is given in S1 Appendix.

## Results

In total (primary tumors, liver metastases and brain metastases of all patients), we identified 283 genetic aberrations. The number of aberrations, including 152 gains, 104 losses, and 27 cn-LOH regions for all sample materials are listed in Table 2.

**Table 2:** Number of all detected chromosomal aberrations for primary tumor as well as liver metastasis and brain metastasis

Patient ID	1	2	3	4
Primary tumor	CNV: 17 gains: 9 losses: 8 cn-LOH: 0	CNV: 15 gains: 9 losses: 6 cn-LOHs: 0	CNV: 10 gains: 3 losses: 7 cn-LOHs: 1	CNV: 26 gains: 22 losses: 4 cn-LOHs: 0
Liver metastasis	CNV: 14 gains: 10 losses: 4 cn-LOHs: 0	CNV: 15 gains: 9 losses: 6 cn-LOHs: 3	CNV: 24 gains: 7 losses: 17 cn-LOHs: 0	Not available
Brain metastasis	CNV: 28 gains: 23 losses: 5 cn-LOHs: 9	CNV: 18 gains: 9 losses: 9 cn-LOHs: 1	CNV: 31 gains: 15 losses: 16 cn-LOHs: 0	CNV: 58 gains: 36 losses: 22 cn-LOHs: 13

### Primary colorectal carcinoma

For primary tumor, we identified an average of 17.3 (± 6.3) chromosomal aberrations. Frequent gains were identified on 8q11.1-q24.3, 13q14.3-q34, and 20q11.21-q13.33 (4/4 patients). Common losses comprised 8p23.3-p12 (3/4 patients) and 18q21.1-q22.2 (4/4 patients). The detected gains 10p11.23-q11.21, 10q24.31-q26.3 and 15q11.2-q15.1 are not previously described.

### Liver metastases

The mean number of chromosomal aberrations for liver metastases are 18.7 (± 5.0). Consistent chromosomal aberrations were gains of 8q11.23-q24.3, 13q12.13-q12.3, and 20q11.21-q13.33 as well as loss of 8p23.3-p12 (3/3 patients, liver metastasis tissue available only from 3 patients). Not previously described aberrations were identified for gains on 2q33.1-q33.3, 5q33.3-q35.3, and 17q24.1-q24.3.

### Brain metastases

Mean chromosomal aberrations were 39.5 (± 22.3). Common gains were identified on chromosomal regions 8q22.3-q24., 13q11.1-q13.2, 13q14.3–32.3 and 20q11.21-q13.33 (4/4 patients). Furthermore, losses were frequently detected of 21q11.2-q21.1 (4/4 patients) as well as of chromosomal regions 4p16.3-p15.33, 4q23-q25, 4q32.3-q35.2, 8p23.3-p12, and 18q21.1-q21.33 for three out of four patients. Totally, 30 out of 135 detected CNV in brain metastases are not previously described: gains on chromosomal regions 3p26.3-p22.1, 4p15.33-p11, 5q14.3-q21.1, 10p14-p11.1, 10p11.23-p11.21, 10p11.23-p11.1, 10q24.31-q26.3, 14q11.2-q24.1, 14q32.12-q32.33, 15q11.2-q21.1, 15q11.2-q26.3, 15q21.2-q21.3, 15q24.2-q26.3, 16p12.2-q24.3, 18p11.32-p11.21, 18q11.1-q21.1, 18q21.33-q23, and 19p13.3-p12 as well as losses on chromosomal regions 2p25.3-p23.1, 2q13-q22.3, 9q21.11-q31.2, 9q22.33-q34.3, 10p15.3-p11.23, 10p15.2-p11.23, 11p15.5-p12, 11q14.1-q25, 12p13.33-p11.1, 19p13.3-q13.43, 20p13-p11.1, and 20p12.3-p11.21. (detailed chromosomal sequences are shown in Table 5, appendix). An overview of all detected chromosomal aberrations for CRC, liver metastasis and brain metastasis of each patient is given in Fig. 4 and Table 5 (including not previously described CNV, marked in bold) in the appendix.

### Comparison of primary colorectal carcinoma, liver metastasis and brain metastasis

In accordance to the description of Sylvester et al. 2015, we detected genetic aberrations displaying a heterogeneity between the different sample materials (intertumoral heterogeneity, intra-individual) and between different patients (intratumoral, inter-individual) (Sylvester and Vakiani 2015). Intertumoral heterogeneity was detected for chromosomal region 9q31.2-q34.3 (Primary tumor and liver metastasis: loss; brain metastasis: gain and loss), chromosomal region 10q24.31-q26.3 (primary tumor: gain; liver metastasis: loss; brain metastasis: gain and loss) as well as chromosomal region 16q23.1–24.1 (primary tumor and liver metastasis: gain; brain metastasis: gain and loss). Intratumoral heterogeneity was identified for chromosomal region 4p15.33-p11 in brain metastasis (patient 1 and 4: gain; patient 2 and 3: loss) and chromosomal region 12p13.33-p12.1 in brain metastasis (patient 1 and 4: gain; patient 2 and 3: loss). Gains on chromosome 5p15.33-q11.2, 5q34-q35.3, 7p22.3-q36.3, 8q11.23-q24.3, 12q11-q24.33, 13q11-q34, 16p13.3-q24.1, and 20q11.21-q13.33 as well as losses on chromosome 5q11.2-q35.3 and 15q11.2-q26.3 were identified in both primary tumor tissue and liver metastases. Occasionally, genetic differences of primary tumor vs. both metastases were identified too. Gain in 11q14.3-q24.2 as well as losses in 1p36.33-p11.1, 3p26.3-p14.1, 4p13-q13.3, 4q21.23-q22.2, 10q23.33-q26.3, 19p13.3-q13.43, and 22q11.1-q13.33 were exclusively identified in liver and brain metastases. Some aberrations were only identified in brain metastases. Our results of SNP array detected gains on chromosomal regions 3p26.3-p22.1, 4p16.3-q35.2 (except the chromosomal region 4q28.2-q31.21), 5q14.3-q21.1, 5q33.2-q33.3, 9p21.3-p21.1, 10q21.2–22.1, 11q13.2-q14.1, 14q11.2-q24.1, and 17q24.3-q25.3 as well as losses on the chromosomal regions 2q13-q22.3, 3p12.2-q29, 4p16.3-q35.2 (except chromosomal regions 4p13-q13.3 and 4q21.3-q22.2), 6q27, 8q21.11-q21.13, 10p15.3-p11.23, 11p15.5-p12, 11q14.1-q25, 12p11.1-q24.33, 13q32.3-q34, 14q24.2-q32.33, and 16q23.1-q24.3.

Interestingly, cn-LOH aberrations were mainly detected in brain metastases compared with primary tumor and liver metastases (primary tumor: one cn-LOH aberration, liver metastases: three cn-LOH aberration, brain metastases: 23 cn-LOH aberrations) (Table 3). Most chromosomal cn-LOH aberrations for liver metastasis (LM) and brain metastasis (BM) have not been previously described.

**Table 3:** Overview of all detected chromosomal cn-LOH regions

Chromosomal region	Aberration	Size	Physical position		CRC	LM	BM	Patient ID	Literature description
		(kb)	Start (kb)	End (kb)					
1q25.1-q31.3	cn-LOH	20,520	175,904,808	196,425,133	–	–	+	4	No
2p25.3-p23.1	cn-LOH	30,622	21,493	30,643,811	–	–	+	4	No
2q31.1-q32.3	cn-LOH	21,330	174,675,256	196,005,511	–	–	+	4	No
3p21.31-p21.1	cn-LOH	6,163	46,663,371	52,826,707	+	–	–	3	Yes
5q11.2-q35.3	cn-LOH	126,807	53,891,523	180,698,312	–	–	+	1	No
5q13.2-q33.3	cn-LOH	86,024	72,304,196	158,328,181	–	+	+	2	No
5q31.3-q32.3	cn-LOH	39,397	141,301,095	180,698,312	–	–	+	4	No
7p22.3-p15.3	cn-LOH	24,024	41,420	24,065,179	–	–	+	4	No
8p23.3-p21.2	cn-LOH	26,795	172,416	26,967,488	–	–	+	4	No
8q11.21-q21.11	cn-LOH	23,250	51,908,365	75,157,868	–	–	+	1	No
9p24.3-p13.1	cn-LOH	38,979	204,737	39,184,065	–	–	+	1	No
9q21.11-q34.3	cn-LOH	70,070	70,984,371	141,054,761	–	–	+	1	No
10p15.3-p14	cn-LOH	10,938	126,069	11,064,241	–	–	+	1	No
12q24.11-q24.33	cn-LOH	20,472	110,127,413	130,599,360	–	–	+	4	No
13q11-q12.12	cn-LOH	6,335	19,084,822	25,419,725	–	+	–	2	No
13q12.3-q34	cn-LOH	83,197	31,905,941	115,103,150	–	+	–	2	No
13q13.2-q14.3	cn-LOH	19,215	34,399,262	53,614,554	–	–	+	4	No
14q24.1-q32.12	cn-LOH	23,227	69,641,739	92,868,468	–	–	+	4	No
17p13.3-p11.1	cn-LOH	21,817	400,958	22,217,883	–	–	+	4	No
17p13.3-p11.2	cn-LOH	20,348	400,958	20,749,243	–	–	+	1	No
17q21.2-q25.3	cn-LOH	41,426	38,837,738	80,263,427	–	–	+	4	No
17q24.3-q25.2	cn-LOH	6,328	68,641,402	74,965,958	–	–	+	1	No
18p11.32-q23	cn-LOH	77,995	12,841	78,007,784	–	–	+	1	No
19p13.3-q13.43	cn-LOH	58,846	247,231	59,093,239	–	–	+	4	No
21q21.2-q22.3	cn-LOH	23,954	24,143,817	48,097,610	–	–	+	1	No
22q11.1-q13.33	cn-LOH	34,351	16,863,069	51,213,826	–	–	+	4	No

*CRC* colorectal carcinoma; *LM* liver metastasis; *BM* brain metastasis; *cn-LOH*: copy neutral loss of heterozygosity

+ : aberration present; – : aberration not present

Based on the detected cn-LOH regions of the CRC-based brain metastases, we identified a total of 902 different cancer genes within the 23 chromosomal cn-LOH regions using NCG7.0 Network of Cancer Genes & Healthy Drivers (Dressler et al. 2022). Subsequently performed pathway analyses of these cancer genes revealed possible impacts on transcriptional processes as well as receptor-mediated pathways. The most relevant pathways are listed in Table 4.

**Table 4:** The most relevant pathways according to Reactome database, including the p-values respectively

Pathway name	p-value
RNA Polymerase II Transcription	1.99*10^–8^
Gene expression (Transcription)	1.10*10^–7^
Signaling by Receptor Tyrosine Kinases	2.29*10^–7^
Oncogene Induced Senescence	5.78*10^–7^
Generic Transcription Pathway	8.27*10^–7^
Signaling by TGF-beta Receptor Complex	1.40*10^–5^
Signaling by MET	2.77*10^–5^
TGF-beta receptor signaling activates SMADs	8.32*10^–5^
Transcriptional regulation by RUNX3	1.60*10^–4^
RUNX1 regulates transcription of genes involved in BCR signaling	1.65*10^–4^

**Fig. 2:**
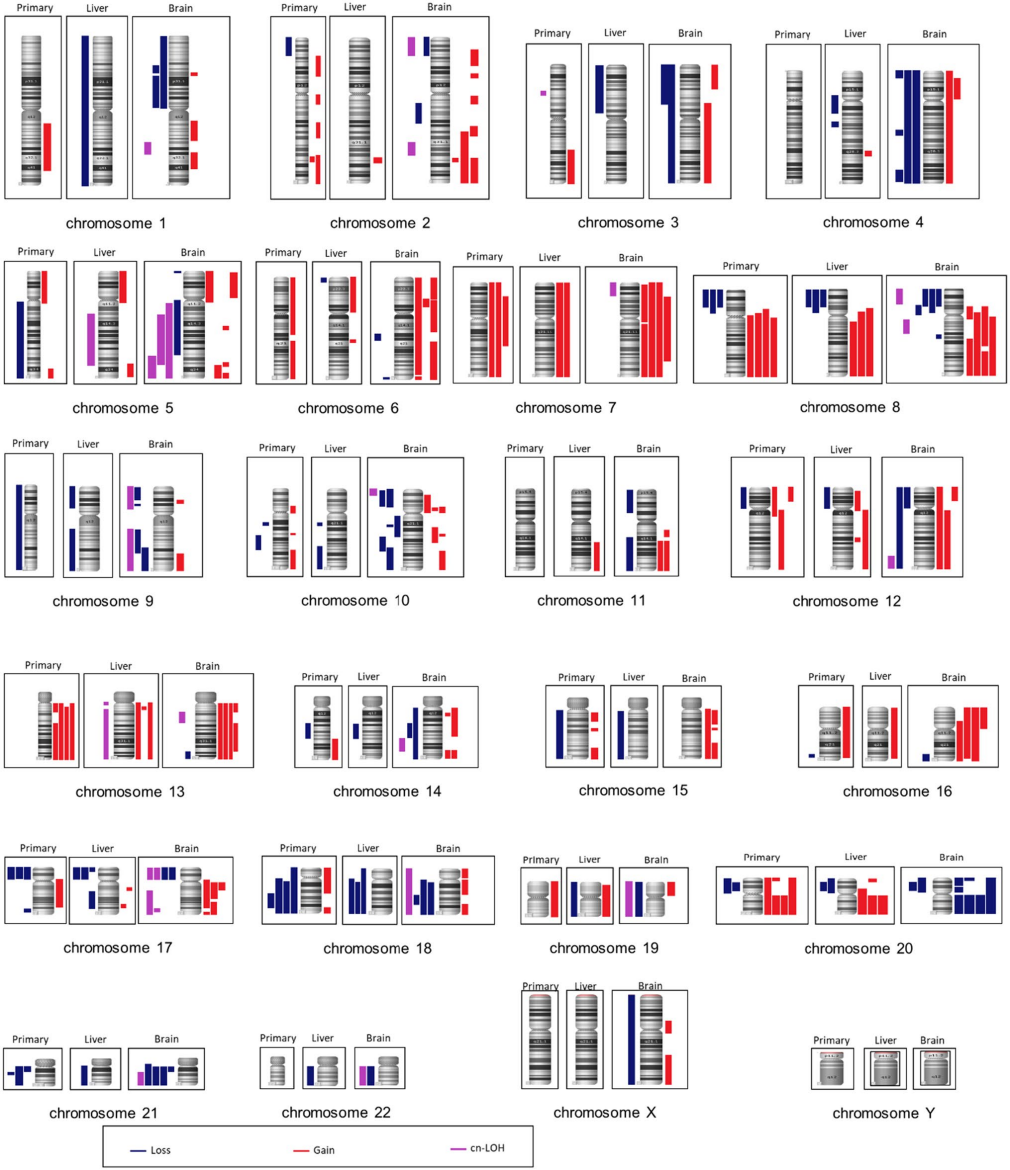
Frequencies of occurrence of chromosomal aberrations in four patients (blue: loss; red: gain, violet: cn-LOH region). Black framed chromosomes (from left to right) show aberrations of colorectal carcinoma (CRC), liver metastasis (LM), and brain metastasis (BM)

Less frequent, the analyses revealed similar chromosomal aberrations among matched primary tumor vs*.* metastases. Such intratumoral and intertumoral similarities were detected for chromosomal regions 8q22.3-q24.3, 13q12.13-q12.3 and 20q11.21-q13.33 (Fig. 2).

## Discussion

Brain metastasis occurs as a late-stage phenomenon of CRC and is associated with an unfavorable prognosis. Although the incidence of brain metastasis is low (2.3%), most patients do not survive the first year after the diagnosis (Diep et al. 2006). In this study, we analyzed genetic aberrations in CRC, liver metastasis and brain metastasis in matched pairs to reveal specific aberrations at the transition to metastatic disease, especially into the brain. An advantage of matched pairs is to reduce intertumoral heterogeneity, which is well known in colorectal cancer (Sylvester and Vakiani 2015).

We could confirm many previously described chromosomal aberrations for primary CRC as well as liver and brain metastases, whereby most of chromosomal imbalances were detected for brain metastases. Also recently presented data of Golas et al. figured out significant differences in the total number of detected aberrations, with most imbalances identified in brain metastases (34th GfH annual meeting, Abstract ID. 183, unpublished data). Gain for chromosomal region 13q11-q34 obviously represents an early event, since it was present in primary CRC and all metastatic sites (Golas et al. 2022). Accordingly, this aberration had been described at the transition from adenoma to primary CRC (Hermsen et al. 2002).

Gains of 7, 8q and 20q as well as losses of 8p and 18q (*DCC/SMAD4*) were conserved from the primary CRC to the metastatic sites. In the literature, loss of 8p had not been more frequently detected in liver metastases compared to primary CRC by Knösel et al. (Knösel et al. 2004). Conversely, gain of 8q could play a role in CRC, since this region harbors genes which are altered in CRC (e.g. *UBR5*, *KLF10, EIF3H*) or which are associated with the metastatic process (e.g. *RRM2B, NOV, RAD21*) (Muñoz-Bellvis et al. 2012). Gutenberg et al. had identified 20q as the most frequently occurring copy number change in 11 patients with brain metastases (Gutenberg et al. 2010). Additionally, this gain had often been detected in CRC primary tumors and been described as an early event in CRC tumor progress (Golas et al. 2022).

Some chromosomal region aberrations, like gain of 10q24.1-q26.3 (primary CRC and brain metastasis in patient 4) have not yet been previously published in analyses of CRC. Within chromosomal region 10q24.1, the *FRAT1* gene (frequently rearranged in advanced T-Cell lymphomas 1) is localized. The gene is involved in the process of carcinogenesis in CRC by the Wnt/β-catenin pathway. According to Zhu et al., FRAT1 regulates the proliferation in colon cancer cells and constitutes a potential target for the treatment of CRC (Zhu et al. 2016).

Homologous recombination as a result of repairing a double-strand break, has been proposed as a possible cause for segmental UPD or cn-LOH formation in cancer, and thus may constitute a further genetic mechanism involved in tumorigenesis (Teh et al. 2005). Cn-LOHs may be a mechanism for inactivating tumor suppressor genes (Torabi et al. 2015).

In a recent study by Nguyen et al., the results indicate an early occurrence of CNVs during tumor development of CRC. These suggest that the more frequent detection of cn-LOH aberrations in brain metastases could be a result of a subsequent somatic reduplication/repair mechanism after previously acquired CNVs during tumor genesis (Golas et al. 2022; Nguyen et al. 2022). Only in one primary tumor, we detected cn-LOH at the chromosomal region 3p21.31-p21.1, which harbors the tumor suppressor gene von Hippel-Lindau (*VHL)* (Torabi et al. 2015). Torabi et al. had detected a UPD in the region 5q of a primary tumor, which then may lead to functional inactivation of the *APC* gene in colon carcinoma (Torabi et al. 2019). Loss of chromosome 5q (including *APC* gene) has been described for the CRC (Takayama et al. 2006).Within these region, we detected a loss in only one of the four primary CRCs, but aberrations (loss or cn-LOHs) in all four brain metastases.

Similar aberrations were detected on chromosome 18 (primary tumor and liver metastasis: loss, brain: loss or cn-LOH). The losses of chromosome 18 are consistent with pre-described aberrations for primary tumors as well as liver metastases (Diep et al. 2006; Gutenberg et al. 2010). Cn-LOH on chromosome 18 has not been previously disclosed in CRC-based brain metastases. Among the previously undescribed detected cn-LOH regions, the aberration within the region 19p13.3-q13.43 (including *CEA* gene) seems to be interesting. A few studies discussed the association of an increased carcinoembryonic antigen (*CEA*) level and brain metastases, whereas Bryani et al. did not found this relationship. In our study, the *CEA* gene, affected by a cn-LOH aberration, could have an impact on the incidence of CRC-based brain metastases. Further analyses on this gene may further elucidate the importance of *CEA* in context of brain metastases development.

Applying genome-wide SNP array analyses, we identified most of the cn-LOH aberrations in brain metastases. For the chromosomal region 17p13.3-p11.1, a cn-LOH aberration was detected in two patients and a loss in the other two patients (Fig. 2). Loss of 17p had been described as the most common numerical change in brain metastases (Gutenberg et al. 2010), containing the tumor suppressor gene *TP53*.

The cancer genes located within the cn-LOH regions of the brain metastases are involved in different interesting pathways. For instance, the transcriptional regulation of the tumor suppressor gene RUNX3 is described for colorectal cancer (Weisenberger et al. 2006). The detected cancer genes NOTCH1 and RUNX1 could possibly influence the RUNX3 signaling pathway and could be meaningful for metastases formation (Gao et al. 2010; Nishina et al. 2011; Whittle et al. 2015). Furthermore, these genes are also described in context with brain tumors (Hai et al. 2018; Steponaitis et al. 2019; Zhao et al. 2019).

However, for primary CRC, many genetic studies had revealed a high grade of heterogeneity within the tumor, with most frequent losses at chromosomal regions 1p, 5q, 8p, 17p, 18p, 18q, 20p and 20q. These losses were confirmed in all four patients of the present study at 5q, 8p, 17p, 18p, 18q and 20p (Diep et al. 2006; Jasmine et al. 2012; Jones et al. 2005; Zarzour et al. 2015).

Besides the detected heterogeneity, chromosomal regions with a similar pattern of chromosomal imbalances between primary tumor and the associated metastases were confirmed for some chromosomal aberrations, e.g., gain on 8q, 13q and 20q. According to Gutenberg et al. (2010), gain of chromosomal regions 13q and 20q had been the most frequent aberrations in both primary tumor and brain metastases (Gutenberg et al. 2010). Diep et al. had found gains of 13q and 20q in both primary tumor and the corresponding liver metastases (Diep et al. 2004). Gains of 13q and 20q are significant molecular events in adenoma-carcinoma progression as well as metastasis formation. These aberrations seem to be important prognostic markers in CRC with a significantly worse outcome for rectal carcinoma (Maharaj et al. 2022).

In the present study, sample materials of a small cohort including primary tumors with liver metastases and brain metastases were analyzed. Therefore, statistical statements are limited. In follow-up studies, a larger cohort would be useful to verify the detected results. Rectal cancer seems to be associated with an increased risk of BM (in comparison to colon cancer) (Christensen et al. 2016). Therefore, a comparative genetic analysis using colon cancer and rectal cancer could be of special interest.

## Conclusions

Employing SNP array analyses on primary colorectal cancer and matched metachronous liver and brain metastases, we detected undescribed chromosomal aberrations in both, primary tumor as well as corresponding metastases. Despite considerable heterogeneity, comprehensive analyses of CRC and matched metastases revealed regions with intratumoral (e.g., gain 13q12.13-q12.3) and intertumoral homogeneity (e.g., gain for 8q22.3-q24.3). Furthermore, the SNP array data revealed a high number of cn-LOH aberrations in brain metastases, which suggests cn-LOHs to be involved when CRC cells develop into the cerebral seeding phenotype. Therefore, further analyses are needed to get more information on the increased occurrence of cn-LOHs in brain metastases.

### Supplementary Information

Below is the link to the electronic supplementary material.

Supplementary file1 (PDF 479 KB)

Supplementary file2 (PDF 531 KB)

Supplementary file3 (DOCX 526 KB)

Supplementary file4 (DOCX 42 KB)

## Data Availability

The datasets generated and/ or analyzed during the current study are available from the corresponding author on reasonable request.

## Abbreviations

CRC: Colorectal carcinoma
CIN: Chromosomal instability
MSI: Microsatellite instability
CIMP: CpG island methylator phenotype
CNV: Copy Number Variation
CGH: Comparative genomic hybridization
SNP: Single nucleotide polymorphism
cn-LOH: Copy-neutral loss of heterozygosity
UPD: Uniparental disomy
LM: Liver metastasis
BM: Brain metastasis
