# Supplementary material for: SNP array genomic analysis of matched pairs of brain and liver metastases in primary colorectal cancer
Source: J Cancer Res Clin Oncol. 2023 Nov 27;149(20):18173–83. doi: 10.1007/s00432-023-05505-4 (PMC10725338; doi:10.1007/s00432-023-05505-4)
Supplement: Supplementary file 1 — Supplementary file1 (PDF 479 KB) [file 432_2023_5505_MOESM1_ESM.pdf]

S1 Appendix: Detailed Workflow for the genetic analyses including SNP array processing and the following bioinformatical evaluation

After pathological investigations, the samples from primary colorectal cancer tissue, liver metastasis and brain metastasis were used for the analyses. The samples were subjected to genome-wide copy number variation (CNV) analysis and assessment of copy number neutral loss of heterozygosity (cn-LOH) chromosomal regions using SNP array (OncoScan® FFPE Assay Kit, ThermoFisher Scientific, Dreieich, Hesse, Germany). Genomic DNA was extracted from tumor samples of colorectal cancer, liver metastasis (except from patient 4 due to technical reasons) and brain metastasis according to the manufacturers' protocols (QIAamp® DNA FFPE Tissue Handbook, QIAGEN, Hilden, North Rhine-Westphalia, Germany). Paraffin was dissolved in Xylene and the samples were lysed under denaturing conditions using Proteinase K. After binding of DNA on column, residual contaminants were removed and pure DNA was eluted. We performed OncoScan® array according to the manufacturers' protocol.

First, in the annealing step, the genomic DNA was mixed with the Annealing Master Mix in an Anneal plate and incubated in the thermocycler for 5 min at 95°C and then for 16-18h at 58°C. Samples were transferred to the 1st PCR Plate and the following reagents were added: Gap Fill Mix, dNTP Mix, Exo Mix, Cleavage Mix and PCR Mix. After the first PCR, an aliquot from each sample was taken and a QC gel was performed. A 2nd PCR amplification was performed and smaller DNA fragments were generated by a HaeIII digest to improve sample hybridization onto the OncoScan® Arrays. The DNA fragment size was checked by the second QC gel. The samples were loaded onto an OncoScan® Array, one well per array. The arrays were placed into the "GeneChip Hybridization Oven 645" (ThermoFisher Scientific, Dreieich, Hesse, Germany; Cat. 00-0331) and hybridized for 16-18h at 49°C. The arrays were stained and washed using the "GeneChip Fluidics Station 450" (ThermoFisher Scientific, Dreieich, Hesse, Germany; Cat. 00-0079), loaded into the "GeneChip Scanner 7G" and array fluorescence intensity was

scanned. Array fluorescence intensity files were generated by the "Affymetrix GeneChip Command Console (AGCC) Software" (ThermoFisher Scientific, Dreieich, Hesse, Germany). For SNP array analyses we used the Chromosome Analysis Suite (ChAS 3.3.0.139, ThermoFisher Scientific, Dreieich, Hesse, Germany) using the copy number and LOH workflows with standard settings. The evaluation was carried out out on the basis of the User Guide for Chromosome Analysis Suite 3.3 (ChAS 3.3), Publication Number 702943, Revision 12, available by Thermo Fisher Scientific (Thermo Fisher Scientific Inc., USA, Waltham).
