# Supplementary material for: SNP array genomic analysis of matched pairs of brain and liver metastases in primary colorectal cancer
Source: J Cancer Res Clin Oncol. 2023 Nov 27;149(20):18173–83. doi: 10.1007/s00432-023-05505-4 (PMC10725338; doi:10.1007/s00432-023-05505-4)
Supplement: Supplementary file 2 — Supplementary file2 (PDF 531 KB) [file 432_2023_5505_MOESM2_ESM.pdf]

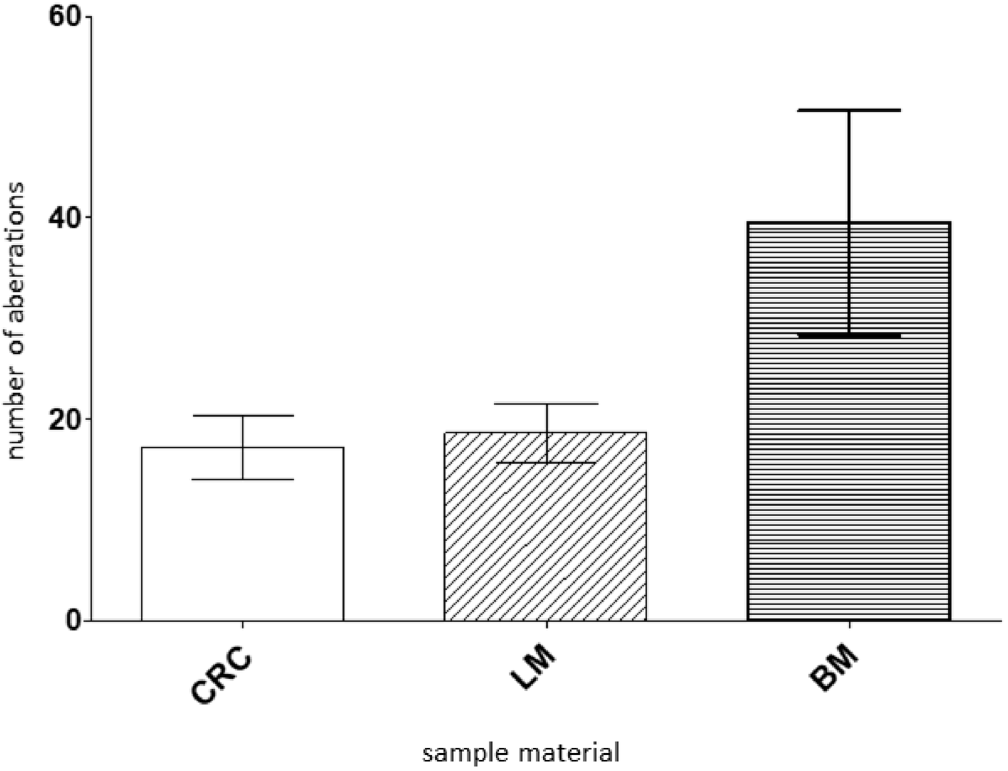

Fig 3 Statistical analysis. Mean number of chromosomal aberrations for primary tumor as well as liver metastases and brain metastases, including standard derivation are shown.

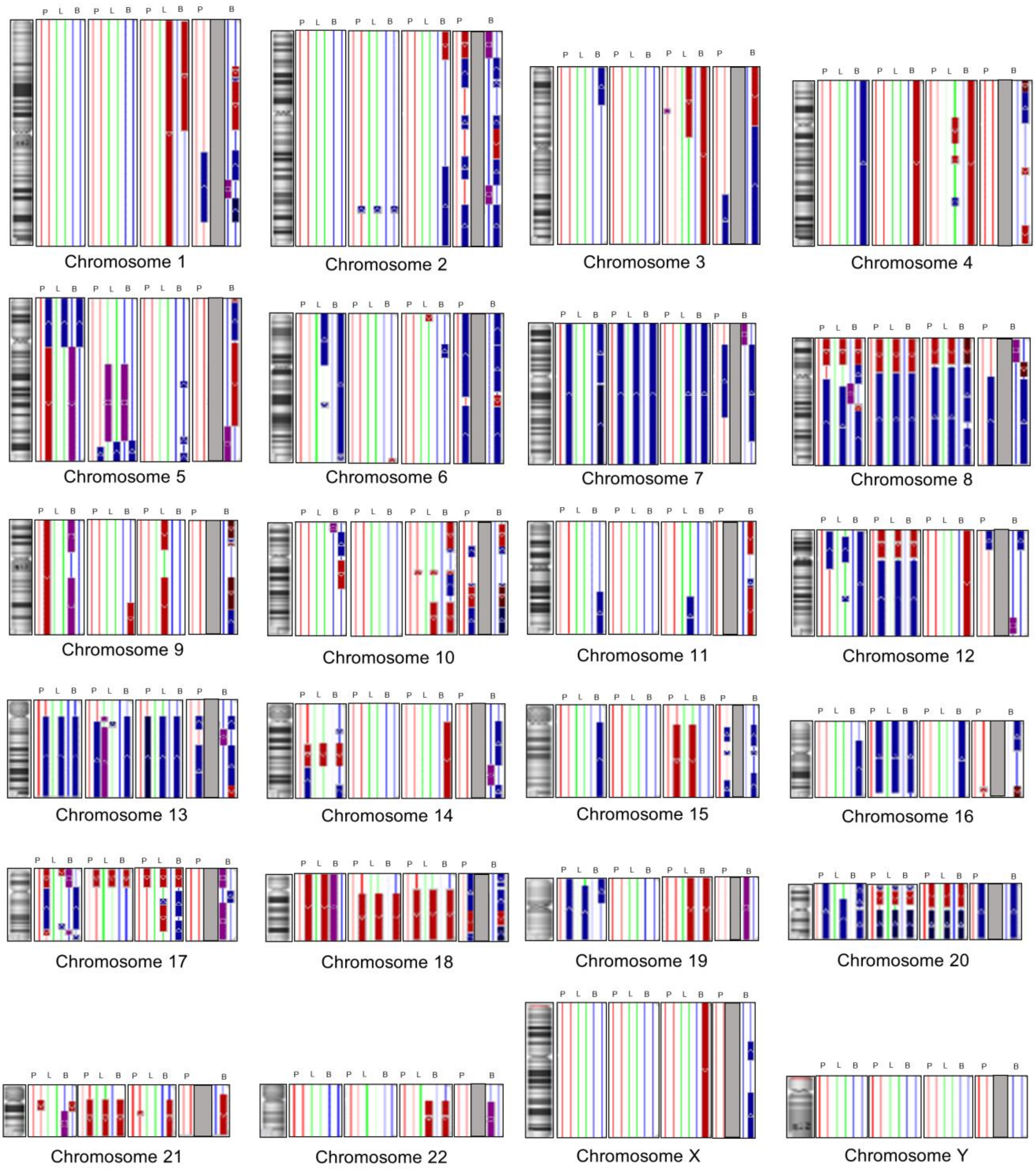

Fig 4 Overview of all detected chromosomal aberrations for each patient and sorted for CRC, liver metastasis and brain metastasis. The detected aberrations on each chromosome are shown for the patients from left to right (Patient 1,2,3, and 4). Gains = blue; loss = red; cn-LOH = violet. P = primary CRC; LM = liver metastasis; BM = brain metastasis
