# Supplementary material for: SNP array genomic analysis of matched pairs of brain and liver metastases in primary colorectal cancer
Source: J Cancer Res Clin Oncol. 2023 Nov 27;149(20):18173–83. doi: 10.1007/s00432-023-05505-4 (PMC10725338; doi:10.1007/s00432-023-05505-4)
Supplement: Supplementary file 3 — Supplementary file3 (DOCX 526 KB) [file 432_2023_5505_MOESM3_ESM.docx]

**Chromosomal aberrant regions - details**

Table with all detected chromosomal aberrant regions, including the potentially affected genes and ISCN-based microarray nomenclature.

| **ID** | **Type** | **Chr.** | **Cytoband Start** | **Cytoband End** | **Size (kbp)** | **Genes** | **Microarray Nomenclature**  **(according to ISCN)** |
| --- | --- | --- | --- | --- | --- | --- | --- |
| 1  CRC | Gain | 5 | p15.33 | q11.2 | 54,082 | PLEKHG4B, LRRC14B, CCDC127, SDHA, HRAT5, PDCD6, AHRR, EXOC3-AS1, EXOC3, PP7080, SLC9A3, LOC100288152, MIR4456, LOC100996325, CEP72, TPPP, ZDHHC11, BRD9, TRIP13, LOC100506688, NKD2, SLC12A7, MIR4635, CTD-3080P12.3, SLC6A19, SLC6A18, TERT, MIR4457, CLPTM1L, LINC01511, SLC6A3, LPCAT1, MIR6075, SDHAP3, LOC728613, MIR4277, MRPL36, NDUFS6, LOC101929034, IRX4, CTD-2194D22.4, LOC100506858, IRX2, C5orf38, LOC105374620, LINC01377, LINC01019, LINC01017, IRX1, LOC101929153, LINC01020, LOC105374631, CTD-2297D10.2, ADAMTS16, ICE1, FLJ33360, MED10, UBE2QL1, LINC01018, NSUN2, SRD5A1, LOC100505625, PAPD7, MIR4278, MIR4454, LOC442132, LOC101929261, ADCY2, C5orf49, FASTKD3, MTRR, LOC729506, MIR4458HG, MIR4458, LOC101929284, SEMA5A, MIR4636, CTD-2201E9.1, SNHG18, SNORD123, TAS2R1, LOC285692, FAM173B, CCT5, CMBL, MARCH6, ROPN1L-AS1, ROPN1L, MIR6131, LOC101929412, LOC389273, ANKRD33B, DAP, CTNND2, LINC01194, DNAH5, TRIO, FAM105A, SNORD141B, SNORD141A, OTULIN, ANKH, LOC100130744, MIR4637, LOC101929454, FBXL7, CTD-2350J17.1, MIR887, MARCH11, LOC101929505, ZNF622, FAM134B, LOC101929524, MYO10, LOC285696, BASP1, LOC401177, LOC101929544, LOC102723526, LOC646241, CDH18, GUSBP1, CDH12, SNORA105A, SNORA105B, PMCHL1, PRDM9, C5orf17, CDH10, LOC340107, LOC105374693, CDH9, LINC01021, LOC105374698, LSP1P3, LOC101929645, LOC101929660, LOC101929681, LOC105374704, CDH6, DROSHA, C5orf22, PDZD2, MIR4279, GOLPH3, MTMR12, ZFR, MIR579, SUB1, NPR3, LOC340113, TARS, ADAMTS12, RXFP3, SLC45A2, AMACR, C1QTNF3-AMACR, C1QTNF3, RAI14, MIR7641-2, TTC23L, RAD1, BRIX1, DNAJC21, AGXT2, PRLR, SPEF2, IL7R, CAPSL, LOC100506406, UGT3A1, UGT3A2, LMBRD2, MIR580, SKP2, NADK2, RANBP3L, SLC1A3, NIPBL-AS1, NIPBL, C5orf42, LOC105374727, NUP155, WDR70, GDNF, GDNF-AS1, LOC105374729, LOC101929745, EGFLAM, EGFLAM-AS4, EGFLAM-AS2, LIFR, LIFR-AS1, MIR3650, OSMR-AS1, LINC01265, OSMR, RICTOR, FYB, C9, DAB2, LOC101926940, LINC00603, PTGER4, TTC33, PRKAA1, LOC100506548, RPL37, SNORD72, CARD6, C7, MROH2B, C6, PLCXD3, OXCT1, OXCT1-AS1, C5orf51, FBXO4, LOC101926960, GHR, CCDC152, SEPP1, FLJ32255, LOC648987, ANXA2R, LOC153684, LOC100132356, LOC100506639, ZNF131, NIM1K, HMGCS1, CCL28, TMEM267, C5orf34, PAIP1, NNT-AS1, NNT, FGF10, FGF10-AS1, BRCAT107, BRCAT54, MRPS30, HCN1, EMB, PARP8, LOC100287592, LOC642366, ISL1, PELO, ITGA1, ITGA2, MOCS2, LOC257396, FST, NDUFS4, ARL15, MIR581, MIR4459, LINC01033, HSPB3, SNX18, LOC102467080 | arr[GRCh37] 5p15.33q11.2(38138_54120346)x3 |
| 1  CRC | Gain | 7 | p22.3 | q36.3 | 159,077 | LOC102723672, LOC100507642, LOC105375115, FAM20C, WI2-2373I1.2, LOC442497, PDGFA, HRAT92, PRKAR1B, LOC101927000, LOC101926963, DNAAF5, SUN1, GET4, ADAP1, COX19, CYP2W1, C7orf50, MIR339, GPR146, GPER1, ZFAND2A, LOC101927021, UNCX, MICALL2, INTS1, MAFK, TMEM184A, PSMG3, PSMG3-AS1, TFAMP1, ELFN1, ELFN1-AS1, MAD1L1, MIR4655, MRM2, NUDT1, SNX8, MIR6836, EIF3B, CHST12, LOC101927181, GRIFIN, LFNG, MIR4648, BRAT1, IQCE, TTYH3, AMZ1, GNA12, CARD11, LOC100129603, SDK1, FOXK1, AP5Z1, MIR4656, RADIL, PAPOLB, MMD2, RNF216P1, RBAK, RBAK-RBAKDN, RBAKDN, ZNF890P, WIPI2, SLC29A4, TNRC18, FBXL18, MIR589, LOC221946, ACTB, FSCN1, RNF216, RNF216-IT1, MIR6874, ZNF815P, OCM, CCZ1, RSPH10B2, RSPH10B, PMS2, AIMP2, EIF2AK1, ANKRD61, USP42, CYTH3, FAM220A, RAC1, DAGLB, KDELR2, GRID2IP, ZDHHC4, C7orf26, ZNF853, ZNF316, ZNF12, PMS2CL, CCZ1B, MIR3683, LOC100131257, C1GALT1, LOC101927354, COL28A1, LOC101927391, MIOS, RPA3, UMAD1, LOC100505921, GLCCI1, ICA1, LOC100505938, NXPH1, PER4, NDUFA4, PHF14, THSD7A, TMEM106B, VWDE, LOC102725191, SCIN, ARL4A, ETV1, DGKB, AGMO, MEOX2, LOC105375166, MEOX2-AS1, ISPD, ISPD-AS1, SOSTDC1, LRRC72, ANKMY2, BZW2, TSPAN13, AGR2, AGR3, AHR, KCCAT333, LOC101927630, SNX13, PRPS1L1, HDAC9, MIR1302-6, TWIST1, FERD3L, TWISTNB, MIR3146, TMEM196, LOC101927668, MACC1, MACC1-AS1, LOC100506098, LOC101927769, LOC101927811, ITGB8, ABCB5, SP8, RPL23P8, LINC01162, SP4, MIR1183, DNAH11, CDCA7L, RAPGEF5, STEAP1B, LOC100506178, LOC401312, LOC541472, IL6, TOMM7, SNORD93, FAM126A, KLHL7-AS1, KLHL7, NUPL2, GPNMB, MALSU1, IGF2BP3, RPS2P32, TRA2A, CLK2P1, CCDC126, FAM221A, STK31, NPY, MPP6, DFNA5, OSBPL3, CYCS, C7orf31, NPVF, MIR148A, NFE2L3, HNRNPA2B1, CBX3, SNX10, LOC105375304, LOC441204, KIAA0087, C7orf71, SKAP2, HOXA1, HOTAIRM1, HOXA2, HOXA3, HOXA-AS2, HOXA4, HOXA-AS3, HOXA5, HOXA6, HOXA7, HOXA9, HOXA10-HOXA9, HOXA10-AS, MIR196B, HOXA10, HOXA11, HOXA11-AS, HOXA13, HOTTIP, EVX1-AS, EVX1, HIBADH, TSL, TAX1BP1, JAZF1, JAZF1-AS1, CREB5, TRIL, LOC100506497, CPVL, LOC101928168, CHN2, LOC102724484, PRR15, LOC646762, MIR550A3, ZNRF2P2, DPY19L2P3, WIPF3, SCRN1, FKBP14, PLEKHA8, MTURN, LOC105375218, ZNRF2, MIR550B1, MIR550A1, DKFZP586I1420, LINC01176, NOD1, GGCT, LOC401320, GARS, CRHR2, INMT, INMT-FAM188B, FAM188B, AQP1, GHRHR, ADCYAP1R1, NEUROD6, CCDC129, PPP1R17, PDE1C, LOC100130673, LSM5, AVL9, DPY19L1P1, ZNRF2P1, MIR550B2, MIR550A2, LINC00997, DPY19L1P2, KBTBD2, RP9P, FKBP9, NT5C3A, RP9, BBS9, BMPER, NPSR1-AS1, NPSR1, DPY19L1, DPY19L2P1, TBX20, LOC401324, HERPUD2, LOC101930085, LOC100506725, SEPT7-AS1, SEPT7, LOC101928618, EEPD1, KIAA0895, ANLN, AOAH, AOAH-IT1, ELMO1, MIR1200, ELMO1-AS1, GPR141, NME8, SFRP4, EPDR1, STARD3NL, TARP, TRG-AS1, AMPH, FAM183BP, VPS41, POU6F2, POU6F2-AS1, YAE1D1, RALA, LINC00265, CDK13, MPLKIP, SUGCT, LINC01450, LINC01449, INHBA, INHBA-AS1, GLI3, LINC01448, C7orf25, PSMA2, MRPL32, HECW1, HECW1-IT1, MIR3943, LOC100506895, STK17A, COA1, BLVRA, MRPS24, URGCP-MRPS24, URGCP, UBE2D4, POLR2J4, SPDYE1, RASA4CP, LINC00957, DBNL, MIR6837, PGAM2, POLM, MIR6838, AEBP1, MIR4649, POLD2, MYL7, GCK, YKT6, CAMK2B, NUDCD3, NPC1L1, DDX56, TMED4, OGDH, ZMIZ2, PPIA, H2AFV, PURB, MIR4657, MYO1G, SNHG15, SNORA9, CCM2, NACAD, TBRG4, SNORA5A, SNORA5C, SNORA5B, RAMP3, ADCY1, SEPT7P2, IGFBP1, IGFBP3, LOC730338, TNS3, LINC01447, C7orf65, LINC00525, PKD1L1, C7orf69, HUS1, SUN3, C7orf57, UPP1, ABCA13, CDC14C, VWC2, ZPBP, C7orf72, IKZF1, FIGNL1, DDC, DDC-AS1, GRB10, COBL, POM121L12, LINC01446, HPVC1, LINC01445, VSTM2A, VSTM2A-OT1, SEC61G, LOC100996654, EGFR, EGFR-AS1, ELDR, LANCL2, VOPP1, FKBP9P1, SEPT14, ZNF713, MRPS17, GBAS, PSPH, CCT6A, SNORA15, SUMF2, PHKG1, CHCHD2, NUPR2, LOC650226, LOC100240728, DKFZp434L192, LOC101928401, LOC401357, LOC100130849, MIR4283-1, MIR4283-2, ZNF479, GUSBP10, LOC105375297, MIR3147, ZNF716, ZNF733P, LOC102724738, LOC100287704, LOC100287834, LINC01005, ZNF727, ZNF735, ZNF679, ZNF736, YWHAEP1, ZNF680, LOC100128885, LOC641746, ZNF107, MIR6839, ZNF138, ZNF273, ZNF117, ERV3-1, CCT6P3, ZNF92, LOC441242, INTS4P2, CCT6P1, SNORA22, VKORC1L1, GUSB, ASL, CRCP, TPST1, LINC00174, GS1-124K5.4, GS1-124K5.11, KCTD7, LOC100996437, RABGEF1, GTF2IRD1P1, GTF2IP23, LOC644794, TMEM248, SBDS, TYW1, MIR4650-2, MIR4650-1, PMS2P4, STAG3L4, LINC01372, LOC102723427, LOC100507468, AUTS2, WBSCR17, MIR3914-1, MIR3914-2, CALN1, TYW1B, SBDSP1, SPDYE7P, POM121, NSUN5P2, TRIM74, LOC541473, LOC100101148, STAG3L1, STAG3L3, PMS2P7, PMS2P5, PMS2P2, SPDYE8P, GTF2IP4, GTF2IP1, NCF1B, NSUN5, TRIM50, FKBP6, FZD9, BAZ1B, BCL7B, TBL2, MLXIPL, VPS37D, DNAJC30, WBSCR22, STX1A, MIR4284, ABHD11-AS1, ABHD11, CLDN3, CLDN4, WBSCR27, WBSCR28, ELN, LIMK1, EIF4H, MIR590, LAT2, RFC2, CLIP2, GTF2IRD1, GTF2I, LOC101926943, NCF1, GTF2IRD2, STAG3L2, RCC1L, GTF2IRD2B, NCF1C, GATSL2, TRIM73, NSUN5P1, POM121C, SPDYE5, PMS2P3, HIP1, CCL26, CCL24, RHBDD2, POR, MIR4651, SNORA14A, TMEM120A, STYXL1, MDH2, GTF2IP7, SRRM3, HSPB1, YWHAG, SSC4D, ZP3, DTX2, FDPSP2, UPK3B, LOC100133091, POMZP3, DTX2P1-UPK3BP1-PMS2P11, PMS2P9, CCDC146, FGL2, GSAP, LOC101927243, PTPN12, APTR, RSBN1L, TMEM60, PHTF2, MAGI2, RPL13AP17, MAGI2-AS2, MAGI2-AS3, GNAI1, LOC101927269, GNAT3, CD36, SEMA3C, LOC105369146, LOC100128317, HGF, CACNA2D1, LOC101927356, PCLO, SEMA3E, SEMA3A, LOC101927378, SEMA3D, LINC00972, GRM3, KIAA1324L, LOC101927420, DMTF1, TMEM243, TP53TG1, CROT, ABCB4, ABCB1, RUNDC3B, SLC25A40, DBF4, ADAM22, SRI, LOC102723885, STEAP4, ZNF804B, C7orf62, STEAP2-AS1, DPY19L2P4, STEAP1, STEAP2, CFAP69, LOC101927446, GTPBP10, LOC101409256, CLDN12, CDK14, FZD1, MTERF1, AKAP9, CYP51A1, CYP51A1-AS1, LRRD1, KRIT1, ANKIB1, LOC105375396, GATAD1, PEX1, RBM48, FAM133B, FAM133DP, CDK6, LOC101927497, SAMD9, SAMD9L, HEPACAM2, VPS50, CALCR, MIR653, MIR489, MIR4652, TFPI2, LOC105375401, GNGT1, GNG11, BET1, COL1A2, CASD1, SGCE, PEG10, PPP1R9A, PON1, PON3, PON2, ASB4, PDK4, DYNC1I1, SLC25A13, MIR591, C7orf76, LOC100506136, SHFM1, DLX6-AS1, DLX6, DLX5, SDHAF3, TAC1, ASNS, MIR5692A1, MIR5692A2, MIR5692C2, MGC72080, OCM2, LMTK2, BHLHA15, TECPR1, BRI3, BAIAP2L1, NPTX2, TMEM130, TRRAP, MIR3609, SCARNA28, LOC101927550, SMURF1, KPNA7, MYH16, ARPC1A, ARPC1B, PDAP1, BUD31, PTCD1, ATP5J2-PTCD1, CPSF4, ATP5J2, ZNF789, ZNF394, ZKSCAN5, FAM200A, ZNF655, GS1-259H13.2, ZSCAN25, CYP3A5, CYP3A7-CYP3A51P, CYP3A7, CYP3A4, CYP3A43, OR2AE1, TRIM4, GJC3, AZGP1, AZGP1P1, ZKSCAN1, ZSCAN21, ZNF3, COPS6, MCM7, MIR25, MIR93, MIR106B, AP4M1, TAF6, CNPY4, MBLAC1, LAMTOR4, C7orf43, MIR4658, GAL3ST4, GPC2, STAG3, GATS, PVRIG, SPDYE3, PMS2P1, STAG3L5P-PVRIG2P-PILRB, STAG3L5P, PVRIG2P, MIR6840, PILRB, PILRA, ZCWPW1, MEPCE, PPP1R35, C7orf61, TSC22D4, NYAP1, AGFG2, SAP25, LRCH4, ZASP, FBXO24, PCOLCE-AS1, PCOLCE, MOSPD3, TFR2, ACTL6B, LOC105375429, GNB2, GIGYF1, POP7, EPO, ZAN, EPHB4, SLC12A9, TRIP6, MIR6875, SRRT, UFSP1, ACHE, MUC3A, MUC12, LOC102724094, MUC17, TRIM56, SERPINE1, AP1S1, MIR4653, VGF, NAT16, MOGAT3, PLOD3, ZNHIT1, CLDN15, FIS1, LOC101927746, IFT22, COL26A1, LINC01007, MYL10, CUX1, SH2B2, MIR4285, SPDYE6, LOC100289561, LOC100630923, PRKRIP1, MIR548O, ORAI2, ALKBH4, LRWD1, MIR5090, MIR4467, POLR2J, RASA4B, POLR2J3, SPDYE2B, SPDYE2, RASA4, POLR2J2, UPK3BL, FAM185A, FBXL13, LRRC17, ARMC10, NAPEPLD, RPL19P12, DPY19L2P2, PMPCB, DNAJC2, PSMC2, SLC26A5, LOC101927870, RELN, ORC5, LHFPL3, LHFPL3-AS1, LHFPL3-AS2, LINC01004, KMT2E-AS1, KMT2E, SRPK2, PUS7, RINT1, EFCAB10, ATXN7L1, CDHR3, SYPL1, NAMPT, CCDC71L, PIK3CG, PRKAR2B, HBP1, COG5, GPR22, DUS4L, BCAP29, SLC26A4-AS1, SLC26A4, CBLL1, SLC26A3, DLD, LAMB1, LAMB4, NRCAM, PNPLA8, THAP5, DNAJB9, C7orf66, EIF3IP1, IMMP2L, LRRN3, DOCK4, DOCK4-AS1, ZNF277, IFRD1, LSMEM1, LOC100996249, LOC101928012, TMEM168, BMT2, HRAT17, GPR85, LINC00998, PPP1R3A, FOXP2, MIR3666, MDFIC, LINC01393, LINC01392, TFEC, TES, LOC102724434, CAV2, CAV1, LINC01510, MET, CAPZA2, ST7-AS1, ST7, ST7-OT4, MIR6132, ST7-AS2, ST7-OT3, WNT2, ASZ1, CFTR, CTTNBP2, LSM8, ANKRD7, LVCAT5, KCND2, TSPAN12, ING3, CPED1, WNT16, FAM3C, PTPRZ1, AASS, FEZF1, FEZF1-AS1, CADPS2, RNF133, RNF148, TAS2R16, SLC13A1, IQUB, NDUFA5, ASB15, LOC102724555, LMOD2, WASL, RNU6-2, HYALP1, HYAL4, SPAM1, LOC105375483, TMEM229A, LOC101928211, GPR37, C7orf77, POT1, POT1-AS1, LOC101928283, LOC101928254, GRM8, MIR592, LOC101928333, ZNF800, LOC100506682, GCC1, ARF5, FSCN3, PAX4, SND1, SND1-IT1, LRRC4, MIR593, MIR129-1, LEP, MGC27345, RBM28, PRRT4, IMPDH1, HILPDA, METTL2B, LINC01000, FAM71F2, FAM71F1, CALU, OPN1SW, CCDC136, FLNC, ATP6V1F, LOC100130705, KCP, IRF5, TNPO3, TPI1P2, LOC407835, TSPAN33, SMO, AHCYL2, STRIP2, SMKR1, NRF1, MIR182, MIR96, MIR183, UBE2H, ZC3HC1, KLHDC10, TMEM209, SSMEM1, CPA2, CPA4, CPA5, LOC105375504, CPA1, CEP41, MEST, MESTIT1, MIR335, COPG2, TSGA13, KLF14, MIR29A, MIR29B1, LINC-PINT, LOC100506860, MKLN1, MKLN1-AS, PODXL, LOC101928782, PLXNA4, LOC101928807, FLJ40288, LOC100506937, CHCHD3, MIR3654, LOC105375512, EXOC4, MIR6133, LOC101928861, LRGUK, SLC35B4, AKR1B1, AKR1B10, AKR1B15, BPGM, CALD1, AGBL3, C7orf49, TMEM140, WDR91, MIR6509, STRA8, CNOT4, NUP205, C7orf73, SLC13A4, FAM180A, MTPN, LUZP6, CHRM2, LOC349160, MIR490, PTN, DGKI, CREB3L2, LOC100130880, AKR1D1, MIR4468, TRIM24, SVOPL, ATP6V0A4, TMEM213, KIAA1549, ZC3HAV1L, ZC3HAV1, TTC26, UBN2, LUC7L2, FMC1, C7orf55-LUC7L2, LOC100129148, KLRG2, CLEC2L, HIPK2, TBXAS1, PARP12, KDM7A, JHDM1D-AS1, SLC37A3, RAB19, MKRN1, DENND2A, ADCK2, NDUFB2-AS1, NDUFB2, BRAF, MRPS33, TMEM178B, AGK, KIAA1147, WEE2-AS1, WEE2, SSBP1, TAS2R3, TAS2R4, TAS2R5, PRSS37, OR9A4, CLEC5A, TAS2R38, MGAM, MGAM2, MOXD2P, PRSS58, TRY2P, MTRNR2L6, PRSS1, PRSS3P2, EPHB6, TRPV6, TRPV5, C7orf34, KEL, OR9A2, OR6V1, OR6W1P, PIP, TAS2R39, TAS2R40, LOC105375545, GSTK1, TMEM139, CASP2, CLCN1, FAM131B, LOC100507507, ZYX, MIR6892, EPHA1, EPHA1-AS1, TAS2R60, TAS2R41, CTAGE15, TCAF2, TCAF2P1, CTAGE6, LOC154761, TCAF1, OR2F2, OR2F1, OR6B1, OR2A5, OR2A25, OR2A12, OR2A2, OR2A14, CTAGE4, ARHGEF35, LOC101928605, OR2A1-AS1, OR2A1, OR2A42, OR2A9P, OR2A20P, OR2A7, ARHGEF34P, CTAGE8, ARHGEF5, NOBOX, TPK1, CNTNAP2, LOC101928700, MIR548F4, LOC105375556, MIR548T, C7orf33, CUL1, EZH2, GHET1, PDIA4, ZNF786, ZNF425, ZNF398, ZNF282, ZNF212, ZNF783, LOC155060, ZNF777, ZNF746, ZNF767P, KRBA1, ZNF467, SSPO, ZNF862, ATP6V0E2-AS1, ATP6V0E2, ACTR3C, LRRC61, ZBED6CL, RARRES2, REPIN1, ZNF775, LOC728743, LINC00996, GIMAP8, GIMAP7, GIMAP4, GIMAP6, GIMAP2, GIMAP1, GIMAP1-GIMAP5, GIMAP5, TMEM176B, TMEM176A, AOC1, KCNH2, NOS3, ATG9B, ABCB8, ASIC3, CDK5, SLC4A2, FASTK, TMUB1, AGAP3, GBX1, ASB10, IQCA1L, ABCF2, CHPF2, MIR671, SMARCD3, NUB1, WDR86, WDR86-AS1, CRYGN, MIR3907, RHEB, PRKAG2, PRKAG2-AS1, GALNTL5, GALNT11, KMT2C, FABP5P3, LINC01003, XRCC2, ACTR3B, LINC01287, DPP6, LOC101929998, PAXIP1-AS2, PAXIP1, PAXIP1-AS1, HTR5A-AS1, HTR5A, INSIG1, BLACE, EN2, CNPY1, LOC100506302, RBM33, SHH, LOC389602, LOC285889, LINC01006, LINC00244, C7orf13, RNF32, LMBR1, NOM1, MNX1, MNX1-AS1, UBE3C, DNAJB6, LOC101927914, PTPRN2, MIR153-2, LOC100506585, MIR595, LINC01022, MIR5707, NCAPG2, ESYT2, WDR60, LINC00689, VIPR2 | arr[GRCh37] 7p22.3q36.3(41420_159118443)x3 |
| 1  CRC | Gain | 8 | q11.1 | q24.3 | 99,396 | LINC00293, LOC100287846, SPIDR, CEBPD, PRKDC, MCM4, UBE2V2, LOC101929268, LOC101929217, EFCAB1, SNAI2, C8orf22, LOC100507464, SNTG1, PXDNL, PCMTD1, ST18, LOC101929341, FAM150A, RB1CC1, NPBWR1, OPRK1, ATP6V1H, RGS20, TCEA1, LYPLA1, MRPL15, SOX17, RP1, XKR4, SBF1P1, LOC105375843, TMEM68, TGS1, LYN, RPS20, SNORD54, MOS, PLAG1, CHCHD7, SDR16C5, SDR16C6P, PENK, LOC101929415, LINC00968, IMPAD1, LINC01606, LOC286177, LINC00588, LOC101929488, LOC286178, LINC01602, FAM110B, LOC101929528, UBXN2B, CYP7A1, SDCBP, NSMAF, TOX, CA8, LINC01301, RAB2A, CHD7, LOC100130298, CLVS1, ASPH, MIR4470, NKAIN3, UG0898H09, GGH, TTPA, YTHDF3-AS1, YTHDF3, LOC102724612, LINC01289, LOC102724623, MIR124-2HG, MIR124-2, LOC401463, BHLHE22, CYP7B1, LINC00251, LINC01299, ARMC1, MTFR1, PDE7A, DNAJC5B, TRIM55, CRH, LINC00967, RRS1-AS1, RRS1, ADHFE1, C8orf46, MYBL1, VCPIP1, C8orf44, C8orf44-SGK3, SGK3, PTTG3P, MCMDC2, SNHG6, SNORD87, TCF24, PPP1R42, COPS5, CSPP1, ARFGEF1, LOC102724708, CPA6, PREX2, C8orf34-AS1, C8orf34, LINC01592, LINC01603, SULF1, SLCO5A1, PRDM14, NCOA2, LOC101926892, TRAM1, LACTB2-AS1, LACTB2, XKR9, EYA1, MSC, MSC-AS1, TRPA1, LOC392232, KCNB2, LOC101926908, TERF1, SBSPON, C8orf89, RPL7, RDH10, RDH10-AS1, STAU2-AS1, STAU2, UBE2W, TCEB1, TMEM70, LY96, JPH1, GDAP1, MIR5681A, MIR5681B, MIR2052HG, MIR2052, PI15, CRISPLD1, CASC9, HNF4G, LINC01111, ZFHX4-AS1, ZFHX4, MIR3149, PEX2, LOC102724874, PKIA, PKIA-AS1, ZC2HC1A, LOC101241902, IL7, STMN2, HEY1, LINC01607, LOC101927040, MRPS28, TPD52, MIR5708, ZBTB10, ZNF704, PAG1, FABP5, PMP2, FABP9, FABP4, FABP12, IMPA1, SLC10A5, ZFAND1, CHMP4C, SNX16, LOC101927141, LINC01419, RALYL, LRRCC1, LOC102723322, E2F5, C8orf59, CA13, CA1, CA3, CA3-AS1, CA2, REXO1L2P, PSKH2, ATP6V0D2, SLC7A13, WWP1, RMDN1, CPNE3, CNGB3, CNBD1, DCAF4L2, MMP16, LOC101929709, RIPK2, OSGIN2, NBN, DECR1, CALB1, LINC00534, LINC01030, TMEM64, NECAB1, C8orf88, TMEM55A, OTUD6B-AS1, OTUD6B, LRRC69, MIR4661, SLC26A7, RUNX1T1, MIR7641-2, LOC102724710, FLJ46284, TRIQK, MIR8084, C8orf87, LINC00535, FAM92A1, RBM12B, RBM12B-AS1, TMEM67, MIR378D2, PDP1, CDH17, GEM, RAD54B, FSBP, KIAA1429, LOC100288748, ESRP1, DPY19L4, INTS8, CCNE2, TP53INP1, NDUFAF6, LOC105375650, MIR3150B, MIR3150A, PLEKHF2, LINC01298, C8orf37, C8orf37-AS1, LOC100500773, GDF6, UQCRB, MTERF3, PTDSS1, LOC102724804, SDC2, CPQ, LOC101927066, TSPYL5, MTDH, LAPTM4B, MATN2, RPL30, SNORA72, ERICH5, RIDA, POP1, NIPAL2, KCNS2, STK3, OSR2, VPS13B, MIR599, MIR875, COX6C, RGS22, MIR1273A, FBXO43, POLR2K, SPAG1, RNF19A, MIR4471, ANKRD46, SNX31, PABPC1, MIR7705, YWHAZ, FLJ42969, ZNF706, NACAP1, GRHL2, NCALD, LOC104054148, MIR5680, RRM2B, UBR5-AS1, UBR5, ODF1, KLF10, AZIN1, AZIN1-AS1, ATP6V1C1, BAALC-AS2, BAALC, MIR3151, BAALC-AS1, FZD6, CTHRC1, SLC25A32, DCAF13, RIMS2, DCSTAMP, DPYS, LRP12, ZFPM2, ZFPM2-AS1, OXR1, ABRA, ANGPT1, RSPO2, EIF3E, EMC2, TMEM74, TRHR, NUDCD1, ENY2, PKHD1L1, EBAG9, SYBU, LOC100132813, KCNV1, LINC01608, LINC01609, CSMD3, MIR2053, TRPS1, LINC00536, EIF3H, LOC105375713, UTP23, RAD21, RAD21-AS1, MIR3610, AARD, SLC30A8, MED30, EXT1, SAMD12, SAMD12-AS1, TNFRSF11B, COLEC10, LOC101927513, MAL2, MIR548AZ, NOV, ENPP2, TAF2, DSCC1, DEPTOR, COL14A1, MRPL13, MTBP, SNTB1, LOC101927543, HAS2, HAS2-AS1, LOC105375734, LINC01151, ZHX2, DERL1, TBC1D31, FAM83A, FAM83A-AS1, MIR4663, C8orf76, ZHX1-C8orf76, ZHX1, ATAD2, MIR548D1, WDYHV1, FBXO32, KLHL38, ANXA13, FAM91A1, FER1L6, FER1L6-AS1, FER1L6-AS2, LOC101927588, TMEM65, TRMT12, RNF139-AS1, RNF139, TATDN1, MIR6844, NDUFB9, MTSS1, MIR4662B, MIR4662A, LINC00964, ZNF572, LOC105375744, SQLE, KIAA0196, NSMCE2, TRIB1, LINC00861, LOC101927657, FAM84B, PCAT1, PCAT2, PRNCR1, CASC19, CCAT1, CASC21, CASC8, CCAT2, POU5F1B, CASC11, MYC, PVT1, MIR1204, TMEM75, MIR1205, MIR1206, MIR1207, MIR1208, LINC00824, LINC00977, CCDC26, MIR3686, GSDMC, FAM49B, MIR5194, ASAP1, ASAP1-IT2, ASAP1-IT1, ADCY8, EFR3A, OC90, HHLA1, KCNQ3, HPYR1, LRRC6, TMEM71, PHF20L1, TG, SLA, MIR7848, WISP1, NDRG1, ST3GAL1, LOC105375773, LOC101927798, LOC101927822, ZFAT, ZFAT-AS1, MIR30B, MIR30D, NCRNA00250, LOC101927845, LINC01591, KHDRBS3, LOC101927915, FAM135B, COL22A1, KCNK9, TRAPPC9, CHRAC1, AGO2, PTK2, DENND3, SLC45A4, LOC105375787, LINC01300, GPR20, PTP4A3, MROH5, MIR1302-7, MIR4539, MIR4472-1, LINC00051, TSNARE1, ADGRB1, ARC, LOC101928087, JRK, PSCA, LY6K, LOC100288181, THEM6, SLURP1, LYPD2, LYNX1, LY6D, GML, CYP11B1, CYP11B2, LOC100133669, CDC42P3, LY6E, C8orf31, LY6H, GPIHBP1, ZFP41, GLI4, MINCR, ZNF696, TOP1MT, RHPN1-AS1, RHPN1, MAFA-AS1, MAFA, ZC3H3, GSDMD, MROH6, NAPRT, EEF1D, TIGD5, PYCRL, TSTA3, ZNF623, ZNF707, BREA2, CCDC166, LOC101928160, MAPK15, FAM83H, MIR4664, FAM83H-AS1, LOC105375800, SCRIB, MIR937, PUF60, NRBP2, MIR6845, EPPK1, PLEC, MIR661, PARP10, GRINA, SPATC1, OPLAH, MIR6846, EXOSC4, MIR6847, GPAA1, CYC1, SHARPIN, MAF1, WDR97, HGH1, MROH1, MIR7112, SCX, BOP1, HSF1, DGAT1, MIR6848, SCRT1, TMEM249, FBXL6, SLC52A2, LOC101928902, ADCK5, CPSF1, MIR939, MIR6849, SLC39A4, VPS28, TONSL, TONSL-AS1, MIR6893, CYHR1, KIFC2, FOXH1, PPP1R16A, GPT, MFSD3, RECQL4, LRRC14, LRRC24, C8orf82, ARHGAP39, ZNF251, ZNF34, RPL8, MIR6850, ZNF517, ZNF7, COMMD5, ZNF250, ZNF16, ZNF252P, TMED10P1, ZNF252P-AS1, C8orf33 | arr[GRCh37] 8q11.1q24.3(46896971_146292734)x3 |
| 1  CRC | Gain | 12 | p13.33 | q13.11 | 47,932 | IQSEC3, LOC574538, SLC6A12, LOC101929384, SLC6A13, LOC102723544, KDM5A, CCDC77, B4GALNT3, NINJ2, LOC105369595, LOC100049716, WNK1, RAD52, ERC1, LINC00942, FBXL14, WNT5B, MIR3649, ADIPOR2, CACNA2D4, LRTM2, LINC00940, DCP1B, CACNA1C-IT2, CACNA1C, CACNA1C-AS4, CACNA1C-IT3, CACNA1C-AS2, CACNA1C-AS1, LOC283440, FKBP4, ITFG2, NRIP2, LOC100507424, FOXM1, RHNO1, TULP3, TEAD4, TSPAN9, PRMT8, THCAT155, CRACR2A, PARP11, CCND2-AS1, CCND2, TIGAR, FGF23, FGF6, C12orf4, RAD51AP1, DYRK4, AKAP3, NDUFA9, LOC101929549, GALNT8, KCNA6, KCNA1, KCNA5, LOC101929584, NTF3, ANO2, VWF, CD9, PLEKHG6, TNFRSF1A, SCNN1A, LTBR, CD27-AS1, CD27, TAPBPL, VAMP1, MRPL51, NCAPD2, SCARNA10, GAPDH, IFFO1, NOP2, CHD4, SCARNA11, LPAR5, ACRBP, ING4, ZNF384, PIANP, COPS7A, MLF2, PTMS, LAG3, CD4, GPR162, P3H3, GNB3, CDCA3, USP5, TPI1, SPSB2, LOC105369632, RPL13P5, DSTNP2, LRRC23, ENO2, ATN1, C12orf57, PTPN6, LOC105369635, MIR200C, MIR141, PHB2, SCARNA12, EMG1, LPCAT3, C1S, C1R, C1RL, C1RL-AS1, RBP5, CLSTN3, PEX5, ACSM4, CD163L1, CD163, APOBEC1, GDF3, DPPA3, CLEC4C, NANOGNB, NANOG, SLC2A14, SLC2A3, FOXJ2, C3AR1, NECAP1, CLEC4A, POU5F1P3, ZNF705A, FAM66C, FAM90A1, FAM86FP, LOC101927905, LINC00937, CLEC6A, CLEC4D, CLEC4E, AICDA, MFAP5, RIMKLB, A2ML1, PHC1, M6PR, KLRG1, LINC00612, A2M-AS1, A2M, PZP, A2MP1, MIR1244-4, MIR1244-3, MIR1244-1, MIR1244-2, LINC00987, LOC642846, LOC101930452, LOC101928030, DDX12P, KLRB1, LOC374443, CLEC2D, CLECL1, CD69, KLRF1, CLEC2B, KLRF2, CLEC2A, LOC100506159, LOC400002, CLEC12A, CLEC1B, CLEC12B, LOC102724020, CLEC9A, CLEC1A, CLEC7A, OLR1, TMEM52B, GABARAPL1, KLRD1, LOC101928100, KLRK1, KLRC4-KLRK1, KLRC4, KLRC3, KLRC2, KLRC1, KLRA1P, MAGOHB, STYK1, YBX3, LOC101928162, TAS2R7, TAS2R8, TAS2R9, TAS2R10, PRR4, PRH1-PRR4, PRH1, TAS2R13, PRH2, PRH1-TAS2R14, TAS2R14, TAS2R50, TAS2R20, TAS2R19, TAS2R31, TAS2R46, TAS2R43, TAS2R30, SMIM10L1, TAS2R42, PRB3, PRB4, PRB1, PRB2, LINC01252, ETV6, BCL2L14, LRP6, MANSC1, LOH12CR2, BORCS5, DUSP16, CREBL2, GPR19, CDKN1B, APOLD1, MIR613, DDX47, RPL13AP20, GPRC5A, MIR614, GPRC5D, HEBP1, LOC100506314, HTR7P1, FAM234B, MIR7641-2, GSG1, EMP1, LINC01559, GRIN2B, ATF7IP, PLBD1, PLBD1-AS1, GUCY2C, HIST4H4, H2AFJ, WBP11, C12orf60, SMCO3, ART4, MGP, ERP27, ARHGDIB, PDE6H, LINC01489, RERG, RERG-AS1, PTPRO, EPS8, STRAP, DERA, SLC15A5, MGST1, LMO3, SKP1P2, MIR3974, RERGL, PIK3C2G, PLCZ1, CAPZA3, PLEKHA5, AEBP2, LOC100506393, PDE3A, SLCO1C1, SLCO1B3, SLCO1B7, SLCO1B1, SLCO1A2, IAPP, PYROXD1, RECQL, GOLT1B, SPX, GYS2, LDHB, KCNJ8, ABCC9, CMAS, ST8SIA1, C2CD5, LOC105369691, ETNK1, LOC101928441, SOX5, MIR920, LOC101928471, LINC00477, BCAT1, C12orf77, LOC645177, LRMP, CASC1, LYRM5, KRAS, LMNTD1, MIR4302, RASSF8-AS1, RASSF8, BHLHE41, SSPN, ITPR2, ASUN, FGFR1OP2, TM7SF3, MED21, C12orf71, STK38L, ARNTL2, ARNTL2-AS1, SMCO2, PPFIBP1, REP15, MRPS35, MANSC4, KLHL42, PTHLH, CCDC91, FAR2, LOC100506606, ERGIC2, OVCH1-AS1, OVCH1, TMTC1, IPO8, CAPRIN2, LOC645485, LINC00941, TSPAN11, DDX11-AS1, DDX11, FAM60A, FLJ13224, DENND5B, DENND5B-AS1, ETFBKMT, AMN1, H3F3C, LOC105369723, KIAA1551, BICD1, FGD4, DNM1L, YARS2, PKP2, SYT10, ALG10, ALG10B, CPNE8, KIF21A, ABCD2, C12orf40, SLC2A13, LRRK2, MUC19, CNTN1, PDZRN4, LOC101927038, GXYLT1, YAF2, ZCRB1, MIR7851, PPHLN1, PRICKLE1, LOC101927058, LOC105369738, LOC105369739, ADAMTS20, PUS7L, IRAK4, TWF1, TMEM117, NELL2, DBX2, RACGAP1P, PLEKHA8P1, RNY5, ANO6, LINC00938, ARID2, SCAF11, SLC38A1, SLC38A2, LOC100288798, SLC38A4, AMIGO2, PCED1B, MIR4698, PCED1B-AS1, LOC105369747, MIR4494, RPAP3, ENDOU | arr[GRCh37] 12p13.33q13.11(189399_48121889)x3 |
| 1  CRC | Gain | 13 | q11 | q34 | 96,018 | LINC00417, ANKRD20A9P, LINC00408, LINC00442, TUBA3C, LOC101928697, ANKRD26P3, LINC00421, TPTE2, LINC00350, MPHOSPH8, PSPC1, ZMYM5, ZMYM2, LINC01072, GJA3, GJB2, GJB6, CRYL1, MIR4499, IFT88, IL17D, EEF1AKMT1, XPO4, LINC00367, LATS2, SAP18, SKA3, MRPL57, LINC01046, MIPEPP3, LINC00539, ZDHHC20, MICU2, FGF9, LINC00424, LINC00540, BASP1P1, SGCG, SACS, SACS-AS1, LINC00327, TNFRSF19, MIPEP, C1QTNF9B-AS1, C1QTNF9B, ANKRD20A19P, SPATA13, MIR2276, SPATA13-AS1, C1QTNF9, LINC00566, PARP4, TPTE2P6, ATP12A, RNF17, CENPJ, TPTE2P1, PABPC3, AMER2, LINC00463, LINC01053, MTMR6, NUP58, ATP8A2, SHISA2, RNF6, CDK8, WASF3, GPR12, USP12, USP12-AS1, USP12-AS2, LINC00412, RPL21, RPL21P28, SNORD102, SNORA27, RASL11A, GTF3A, MTIF3, LNX2, POLR1D, GSX1, PDX1-AS1, PDX1, ATP5EP2, LINC00543, CDX2, URAD, FLT3, PAN3-AS1, PAN3, FLT1, POMP, SLC46A3, MTUS2, MTUS2-AS1, SLC7A1, LOC102723345, UBL3, LINC00297, LINC00572, LINC00544, LINC00365, KATNAL1, LINC00426, LINC01058, HMGB1, USPL1, ALOX5AP, LINC00398, LINC00545, TEX26-AS1, MEDAG, TEX26, HSPH1, B3GLCT, RXFP2, EEF1DP3, FRY-AS1, FRY, ZAR1L, BRCA2, N4BP2L1, N4BP2L2, MINOS1P1, N4BP2L2-IT2, PDS5B, LINC00423, KL, STARD13, STARD13-AS, RFC3, LINC00457, NBEA, MAB21L1, LINC00445, DCLK1, SOHLH2, CCDC169-SOHLH2, CCDC169, SPG20, SPG20-AS1, CCNA1, SERTM1, RFXAP, SMAD9, ALG5, EXOSC8, SUPT20H, CSNK1A1L, LINC01048, LINC00547, POSTN, TRPC4, LINC00571, UFM1, LINC00437, LINC00366, FREM2, STOML3, PROSER1, NHLRC3, LHFP, COG6, MIR4305, LINC00332, LINC00548, LINC00598, FOXO1, MIR320D1, MRPS31, SLC25A15, TPTE2P5, MIR621, SUGT1P3, ELF1, WBP4, MIR3168, KBTBD6, LOC101929140, KBTBD7, MTRF1, NAA16, OR7E37P, RGCC, VWA8, MIR5006, VWA8-AS1, DGKH, AKAP11, LOC105370177, TNFSF11, FAM216B, LINC01050, LINC00428, EPSTI1, DNAJC15, LINC00400, ENOX1, ENOX1-AS2, CCDC122, LACC1, LINC00284, LINC00390, SMIM2-AS1, SMIM2, SMIM2-IT1, MIR8079, SERP2, TUSC8, TSC22D1, TSC22D1-AS1, LINC00330, NUFIP1, GPALPP1, LOC101929259, GTF2F2, KCTD4, TPT1, SNORA31, TPT1-AS1, SLC25A30, SLC25A30-AS1, COG3, ERICH6B, LINC01055, SPERT, SIAH3, ZC3H13, CPB2-AS1, CPB2, LCP1, LRRC63, LINC00563, KIAA0226L, LINC01198, LRCH1, ESD, HTR2A, HTR2A-AS1, LINC00562, SUCLA2, NUDT15, MED4, MED4-AS1, ITM2B, LINC00441, RB1, LPAR6, RCBTB2, LINC00462, CYSLTR2, FNDC3A, MLNR, LOC105370203, CDADC1, CAB39L, SETDB2, SETDB2-PHF11, PHF11, RCBTB1, ARL11, EBPL, KPNA3, CTAGE10P, SPRYD7, DLEU2, MIR3613, TRIM13, KCNRG, MIR16-1, MIR15A, DLEU1, ST13P4, DLEU1-AS1, DLEU7, DLEU7-AS1, RNASEH2B-AS1, RNASEH2B, GUCY1B2, LINC00371, FAM124A, SERPINE3, MIR5693, INTS6, INTS6-AS1, MIR4703, WDFY2, DHRS12, LINC00282, CCDC70, ATP7B, ALG11, UTP14C, NEK5, LOC101929657, NEK3, MRPS31P5, LOC103191607, THSD1, VPS36, CKAP2, TPTE2P3, HNRNPA1L2, SUGT1, LECT1, MIR759, PCDH8, OLFM4, LINC01065, LINC00558, LINC00458, MIR1297, MIR5007, PRR20A, PRR20D, PRR20E, PRR20B, PRR20C, PCDH17, LOC101926897, LINC00374, DIAPH3, DIAPH3-AS1, DIAPH3-AS2, LINC00434, TDRD3, LINC00378, MIR3169, PCDH20, LOC101926951, LINC00358, LINC01075, LINC00448, LINC00376, LINC00395, OR7E156P, LOC102723968, LINC01052, MIR548X2, MIR4704, PCDH9, PCDH9-AS2, PCDH9-AS3, PCDH9-AS4, LINC00364, LINC00550, LINC00383, KLHL1, ATXN8OS, LINC00348, DACH1, MZT1, BORA, DIS3, PIBF1, KLF5, LINC00392, KLF12, LINC00381, LINC00347, CTAGE11P, LINC01078, TBC1D4, COMMD6, UCHL3, LMO7-AS1, LMO7, LMO7DN, LMO7DN-IT1, KCTD12, BTF3P11, ACOD1, CLN5, FBXL3, MYCBP2, MYCBP2-AS1, SCEL, SCEL-AS1, LOC100129307, SLAIN1, MIR3665, EDNRB-AS1, EDNRB, LINC01069, LINC00446, RNF219-AS1, POU4F1, RNF219, LINC00331, RBM26, RBM26-AS1, NDFIP2-AS1, NDFIP2, LINC01068, LINC01038, LINC00382, LINC01080, SPRY2, LINC00377, LINC00564, SLITRK1, LINC00333, SNORA107, LINC00375, LINC00351, SLITRK6, LINC00430, MIR4500HG, MIR4500, SLITRK5, LINC00397, LOC105370306, LINC00433, LINC01047, LINC00440, LINC01040, LINC00353, LINC00559, MIR622, LINC01049, LINC00410, LINC00380, LINC00379, MIR17HG, MIR17, MIR18A, MIR19A, MIR20A, MIR19B1, MIR92A1, GPC5, GPC5-AS2, MIR548AS, GPC5-AS1, LINC00363, GPC6, GPC6-AS2, GPC6-AS1, DCT, TGDS, GPR180, LOC101927248, SOX21, SOX21-AS1, LOC101927284, LINC00557, ABCC4, CLDN10, CLDN10-AS1, DZIP1, DNAJC3-AS1, DNAJC3, UGGT2, HS6ST3, MIR4501, LINC00359, OXGR1, LINC00456, MBNL2, RAP2A, IPO5, FARP1, RNF113B, MIR3170, STK24, SLC15A1, DOCK9, DOCK9-AS1, DOCK9-AS2, UBAC2-AS1, UBAC2, GPR18, GPR183, FKSG29, MIR623, LINC01232, LINC00449, TM9SF2, LINC01039, CLYBL, MIR4306, CLYBL-AS2, CLYBL-AS1, LOC101927437, ZIC5, ZIC2, LINC00554, LOC105370333, PCCA, PCCA-AS1, GGACT, TMTC4, NALCN-AS1, LINC00411, NALCN, ITGBL1, FGF14, MIR2681, MIR4705, FGF14-IT1, FGF14-AS1, FGF14-AS2, TPP2, METTL21C, CCDC168, TEX30, KDELC1, BIVM, BIVM-ERCC5, ERCC5, METTL21EP, SLC10A2, LINC01309, DAOA-AS1, DAOA, LINC00343, LINC00460, EFNB2, ARGLU1, LINC00551, LINC00443, FAM155A, MIR1267, FAM155A-IT1, LIG4, ABHD13, TNFSF13B, MYO16, MYO16-AS1, LINC00399, LINC00676, IRS2, LINC00396, COL4A1, COL4A2, MIR8073, COL4A2-AS2, COL4A2-AS1, RAB20, NAXD, CARS2, ING1, LINC00567, LINC00346, ANKRD10, LINC00431, LINC00368, ARHGEF7-AS2, ARHGEF7, ARHGEF7-AS1, LOC101060553, TEX29, LOC105370369, LINC00354, LINC00403, SOX1, LOC100506016, LINC01070, LOC101928730, LINC01043, LINC01044, SPACA7, TUBGCP3, ATP11AUN, ATP11A, ATP11A-AS1, MCF2L-AS1, MCF2L, F7, F10, F10-AS1, PROZ, PCID2, CUL4A, MIR8075, LAMP1, GRTP1, GRTP1-AS1, LOC101928841, ADPRHL1, DCUN1D2, TMCO3, TFDP1, ATP4B, GRK1, LINC00552, TMEM255B, GAS6-AS1, GAS6, GAS6-AS2, LINC00452, LINC00565, RASA3, CDC16, MIR548AR, MIR4502, UPF3A, CHAMP1, LINC01054 | arr[GRCh37] 13q11q34(19084822_115103150)x3 |
| 1  CRC | Gain | 14 | q24.2 | q32.33 | 35,894 | PCNX1, SNORD56B, LOC145474, SIPA1L1, RGS6, MIR7843, DPF3, DCAF4, ZFYVE1, RBM25, PSEN1, PAPLN, LOC101928123, NUMB, LOC101928143, HEATR4, C14orf169, ACOT1, ACOT2, ACOT4, ACOT6, DNAL1, PNMA1, ELMSAN1, MIR4505, LOC100506476, LOC100506498, PTGR2, ZNF410, FAM161B, COQ6, ENTPD5, BBOF1, ALDH6A1, LIN52, VSX2, ABCD4, VRTN, SYNDIG1L, NPC2, MIR4709, ISCA2, LTBP2, AREL1, FCF1, YLPM1, PROX2, DLST, RPS6KL1, PGF, EIF2B2, MLH3, ACYP1, ZC2HC1C, NEK9, TMED10, FOS, LINC01220, JDP2, BATF, LOC102724153, FLVCR2, MIR7641-2, C14orf1, TTLL5, TGFB3, IFT43, GPATCH2L, ESRRB, VASH1, LOC100506603, ANGEL1, LRRC74A, LINC01629, IRF2BPL, LOC283575, LOC102724190, CIPC, ZDHHC22, TMEM63C, NGB, MIR1260A, POMT2, GSTZ1, TMED8, SAMD15, NOXRED1, VIPAS39, AHSA1, ISM2, SPTLC2, ALKBH1, SLIRP, SNW1, C14orf178, ADCK1, NRXN3, LOC105370586, DIO2, DIO2-AS1, CEP128, TSHR, GTF2A1, SNORA79, LOC101928504, STON2, LOC100506700, SEL1L, LINC01467, LOC105370605, LINC00911, FLRT2, LOC101928767, LOC283585, GALC, GPR65, LOC101928791, LINC01146, KCNK10, SPATA7, PTPN21, ZC3H14, EML5, TTC8, FOXN3, FOXN3-AS1, FOXN3-AS2, EFCAB11, TDP1, KCNK13, PSMC1, NRDE2, CALM1, LINC00642, LOC105370619, TTC7B, LOC101928909, LOC105370622, RPS6KA5, C14orf159, SNORA11B, GPR68, CCDC88C, PPP4R3A, CATSPERB, TC2N, FBLN5, TRIP11, ATXN3, NDUFB1, CPSF2, SLC24A4, RIN3, LGMN, GOLGA5, LOC101929002, CHGA, ITPK1, ITPK1-AS1, MOAP1, TMEM251, C14orf142, UBR7, BTBD7, UNC79, COX8C, PRIMA1, FAM181A-AS1, FAM181A, ASB2, MIR4506, LINC00521, OTUB2, DDX24, IFI27L1, IFI27, IFI27L2, PPP4R4, SERPINA10, SERPINA6, SERPINA2, SERPINA1, SERPINA11, SERPINA9, SERPINA12, SERPINA4, SERPINA5, SERPINA3, SERPINA13P, GSC, DICER1, MIR3173, DICER1-AS1, CLMN, LOC101929080, LINC00341, SYNE3, SNHG10, SCARNA13, GLRX5, TCL6, TCL1B, TCL1A, TUNAR, C14orf132, BDKRB2, BDKRB1, ATG2B, GSKIP, AK7, LOC730202, PAPOLA, VRK1, LINC00618, LOC101929241, LOC100129345, LINC01550, C14orf177, BCL11B, SETD3, CCNK, CCDC85C, HHIPL1, CYP46A1, EML1, EVL, MIR151B, MIR342, DEGS2, YY1, MIR6764, SLC25A29, MIR345, SLC25A47, WARS, WDR25, BEGAIN, LINC00523, DLK1, MIR2392, MEG3, MIR770, MIR493, MIR337, MIR665, RTL1, MIR431, MIR433, MIR127, MIR432, MIR136, MEG8, MIR370, SNORD113-1, SNORD113-2, SNORD113-4, SNORD113-5, SNORD113-6, SNORD113-7, SNORD113-9, SNORD114-1, SNORD114-2, SNORD114-3, SNORD114-4, SNORD114-5, SNORD114-6, SNORD114-7, SNORD114-8, SNORD114-9, SNORD114-10, SNHG24, SNORD114-11, SNORD114-12, SNORD114-13, SNORD114-14, SNORD114-15, SNORD114-16, SNORD114-17, SNORD114-18, SNORD114-19, SNORD114-20, SNORD114-21, SNORD114-22, SNORD114-23, SNORD114-24, SNORD114-25, SNORD114-26, SNORD114-27, SNORD114-28, SNORD114-29, SNORD114-30, SNORD114-31, MIR379, MIR411, MIR299, MIR380, MIR1197, MIR323A, MIR758, MIR329-1, MIR329-2, MIR494, MIR1193, MIR543, MIR495, MIR376C, MIR376A2, MIR654, MIR376B, MIR376A1, MIR300, MIR1185-1, MIR1185-2, MIR381HG, MIR381, MIR487B, MIR539, MIR889, MIR544A, MIR655, MIR487A, MIR382, MIR134, MIR668, MIR485, MIR323B, MIR154, MIR496, MIR377, MIR541, MIR409, MIR412, MIR369, MIR410, MIR656, MEG9, LINC00524, LOC100507277, DIO3OS, MIR1247, DIO3, LINC00239, PPP2R5C, DYNC1H1, HSP90AA1, WDR20, MOK, ZNF839, CINP, TECPR2, ANKRD9, MIR4309, RCOR1, TRAF3, AMN, CDC42BPB, EXOC3L4, TNFAIP2, LINC00605, LOC105378183, EIF5, SNORA28, MARK3, CKB, TRMT61A, BAG5, APOPT1, KLC1, XRCC3, ZFYVE21, PPP1R13B, LINC00637, C14orf2, TDRD9, RD3L, ASPG, MIR203A, MIR203B, KIF26A, C14orf180, TMEM179, LOC101929634, MIR4710, INF2, ADSSL1, SIVA1, AKT1, ZBTB42, LINC00638, CEP170B, PLD4, AHNAK2, C14orf79, CDCA4, GPR132, LOC102723354, JAG2, MIR6765, NUDT14, BRF1, BTBD6, PACS2, TEX22, LOC100507437, MTA1, CRIP2, CRIP1, C14orf80, TMEM121, LOC105370697, MIR8071-1, MIR8071-2, ELK2AP, MIR4507, MIR4538, MIR4537, MIR4539, KIAA0125, ADAM6, LINC00226, LINC00221, MIR5195 | arr[GRCh37] 14q24.2q32.33(71387979_107282024)x3 |
| 1  CRC | Gain | 17 | p11.2 | q24.3 | 47,773 | NATD1, MAP2K3, KCNJ12, KCNJ18, C17orf51, FAM27E5, FLJ36000, MTRNR2L1, LOC105371703, MIR4522, WSB1, TBC1D3P5, KSR1, LGALS9, NOS2, LYRM9, NLK, PYY2, PPY2P, KRT18P55, TMEM97, IFT20, TNFAIP1, POLDIP2, TMEM199, MIR4723, SEBOX, VTN, SARM1, SLC46A1, SLC13A2, FOXN1, UNC119, PIGS, ALDOC, SPAG5, SPAG5-AS1, SGK494, KIAA0100, SDF2, SUPT6H, PROCA1, RAB34, NARR, RPL23A, SNORD42B, SNORD4A, SNORD42A, SNORD4B, TLCD1, NEK8, TRAF4, FAM222B, ERAL1, MIR451A, MIR451B, MIR144, MIR4732, FLOT2, DHRS13, PHF12, LOC101927018, SEZ6, PIPOX, MYO18A, TIAF1, CRYBA1, NUFIP2, MIR4523, TAOK1, ABHD15, TP53I13, GIT1, ANKRD13B, CORO6, SSH2, EFCAB5, NSRP1, MIR423, MIR3184, SLC6A4, BLMH, TMIGD1, CPD, GOSR1, TBC1D29, LOC107133515, SH3GL1P2, SUZ12P1, CRLF3, ATAD5, TEFM, ADAP2, RNF135, DPRXP4, MIR4733, NF1, OMG, EVI2B, EVI2A, RAB11FIP4, MIR4724, MIR193A, MIR4725, MIR365B, COPRS, UTP6, SUZ12, LRRC37B, SH3GL1P1, LOC105371730, RHOT1, ARGFXP2, RHBDL3, C17orf75, MIR632, ZNF207, PSMD11, CDK5R1, MYO1D, TMEM98, SPACA3, ASIC2, AA06, LOC101927239, CCL2, CCL7, CCL11, CCL8, CCL13, CCL1, C17orf102, TMEM132E, CCT6B, ZNF830, LIG3, RFFL, RAD51L3-RFFL, RAD51D, FNDC8, NLE1, UNC45B, SLC35G3, SLFN5, SLFN11, SLFN12, SLFN13, SLFN12L, SLFN14, LOC105371743, SNORD7, PEX12, AP2B1, RASL10B, GAS2L2, MMP28, C17orf50, TAF15, HEATR9, CCL5, LRRC37A8P, RDM1, LYZL6, CCL16, CCL14, CCL15-CCL14, CCL15, CCL23, CCL18, CCL3, CCL4, CCL3L3, CCL3L1, CCL4L1, CCL4L2, TBC1D3H, TBC1D3G, TBC1D3F, TBC1D3B, ZNHIT3, MYO19, PIGW, GGNBP2, DHRS11, MRM1, LOC102723471, LHX1, AATF, MIR2909, ACACA, SNORA90, C17orf78, TADA2A, DUSP14, SYNRG, DDX52, MIR378J, HNF1B, YWHAEP7, TBC1D3C, TBC1D3L, TBC1D3, TBC1D3I, TBC1D3K, LOC101060389, TBC1D3E, LOC440434, MRPL45, GPR179, SOCS7, ARHGAP23, SRCIN1, C17orf96, MIR4734, MLLT6, MIR4726, CISD3, PCGF2, PSMB3, PIP4K2B, CWC25, MIR4727, C17orf98, RPL23, SNORA21, LASP1, MIR6779, LINC00672, FBXO47, LOC105371766, LRRC37A11P, LOC100131347, PLXDC1, ARL5C, CACNB1, RPL19, STAC2, FBXL20, MED1, CDK12, NEUROD2, PPP1R1B, STARD3, TCAP, PNMT, PGAP3, ERBB2, MIR4728, MIEN1, GRB7, IKZF3, ZPBP2, GSDMB, ORMDL3, LRRC3C, GSDMA, PSMD3, CSF3, MED24, MIR6884, SNORD124, THRA, NR1D1, MSL1, CASC3, MIR6866, RAPGEFL1, MIR6867, WIPF2, CDC6, RARA, RARA-AS1, GJD3, TOP2A, IGFBP4, TNS4, CCR7, SMARCE1, KRT222, KRT24, KRT25, KRT26, KRT27, KRT28, KRT10, TMEM99, KRT12, KRT20, KRT23, KRT39, KRT40, KRTAP3-3, KRTAP3-2, KRTAP3-1, KRTAP1-5, KRTAP1-4, KRTAP1-3, KRTAP1-1, KRTAP2-1, KRTAP2-2, KRTAP2-3, KRTAP2-4, KRTAP4-7, KRTAP4-8, KRTAP4-9, KRTAP4-11, KRTAP4-12, KRTAP4-6, KRTAP4-5, KRTAP4-4, KRTAP4-3, KRTAP4-2, KRTAP4-1, KRTAP9-1, KRTAP9-2, KRTAP9-3, KRTAP9-9, KRTAP9-8, KRTAP9-4, KRTAP9-6, KRTAP9-7, KRTAP29-1, KRTAP16-1, KRTAP17-1, KRT33A, KRT33B, KRT34, KRT31, LOC100505782, KRT37, KRT38, KRT32, KRT35, KRT36, KRT13, KRT15, MIR6510, KRT19, LINC00974, KRT9, KRT14, KRT16, KRT17, KRT42P, EIF1, GAST, HAP1, JUP, P3H4, FKBP10, NT5C3B, KLHL10, KLHL11, ACLY, TTC25, CNP, DNAJC7, NKIRAS2, ZNF385C, DHX58, KAT2A, HSPB9, RAB5C, KCNH4, HCRT, GHDC, STAT5B, STAT5A, STAT3, PTRF, ATP6V0A1, MIR548AT, MIR5010, NAGLU, HSD17B1, COASY, MLX, PSMC3IP, FAM134C, TUBG1, TUBG2, PLEKHH3, CCR10, CNTNAP1, EZH1, MIR6780A, RAMP2-AS1, RAMP2, VPS25, WNK4, COA3, CNTD1, BECN1, MIR6781, PSME3, AOC2, AOC3, AOC4P, LINC00671, G6PC, AARSD1, PTGES3L-AARSD1, PTGES3L, RUNDC1, RPL27, IFI35, VAT1, RND2, BRCA1, NBR2, LOC101929767, NBR1, TMEM106A, LINC00854, LINC00910, ARL4D, MIR2117, DHX8, ETV4, MEOX1, SOST, DUSP3, C17orf105, MPP3, CD300LG, MPP2, FAM215A, LOC107546764, PPY, PYY, NAGS, TMEM101, LSM12, G6PC3, HDAC5, LOC105371789, C17orf53, ASB16, ASB16-AS1, TMUB2, ATXN7L3, UBTF, MIR6782, SLC4A1, RUNDC3A-AS1, RUNDC3A, SLC25A39, GRN, FAM171A2, ITGA2B, GPATCH8, FZD2, LINC01180, MEIOC, CCDC43, DBF4B, ADAM11, GJC1, HIGD1B, EFTUD2, CCDC103, GFAP, KIF18B, MIR6783, C1QL1, DCAKD, NMT1, PLCD3, MIR6784, ACBD4, HEXIM1, HEXIM2, LOC105371795, FMNL1, MAP3K14-AS1, SPATA32, MAP3K14, ARHGAP27, PLEKHM1, MIR4315-2, MIR4315-1, LRRC37A4P, LOC644172, MGC57346, MGC57346-CRHR1, CRHR1-IT1, CRHR1, MAPT-AS1, SPPL2C, MAPT, MAPT-IT1, STH, KANSL1, KANSL1-AS1, LRRC37A, ARL17A, ARL17B, NSFP1, LRRC37A2, NSF, WNT3, WNT9B, GOSR2, MIR5089, RPRML, CDC27, MYL4, ITGB3, THCAT158, EFCAB13, MRPL45P2, NPEPPS, KPNB1, TBKBP1, TBX21, OSBPL7, MRPL10, LRRC46, SCRN2, SP6, LOC102724532, SP2, SP2-AS1, PNPO, PRR15L, CDK5RAP3, COPZ2, MIR152, NFE2L1, CBX1, SNX11, SKAP1, MIR1203, THRA1/BTR, LOC101927166, HOXB1, HOXB2, HOXB-AS1, HOXB3, HOXB4, MIR10A, HOXB-AS3, HOXB5, HOXB6, HOXB7, HOXB8, HOXB9, MIR196A1, PRAC1, PRAC2, MIR3185, HOXB13, TTLL6, CALCOCO2, LOC105371814, ATP5G1, UBE2Z, SNF8, GIP, IGF2BP1, B4GALNT2, GNGT2, ABI3, PHOSPHO1, FLJ40194, MIR6129, ZNF652, LOC102724596, PHB, LOC101927207, NGFR, LOC100288866, MIR6165, NXPH3, SPOP, SLC35B1, FAM117A, KAT7, TAC4, FLJ45513, DLX4, DLX3, LOC284080, ITGA3, PDK2, SAMD14, PPP1R9B, SGCA, HILS1, COL1A1, LOC101927230, TMEM92, TMEM92-AS1, XYLT2, MRPL27, EME1, LRRC59, ACSF2, CHAD, RSAD1, MYCBPAP, EPN3, LOC105371824, SPATA20, CACNA1G-AS1, CACNA1G, ABCC3, ANKRD40, LUC7L3, LINC00483, MIR8059, WFIKKN2, TOB1, TOB1-AS1, SPAG9, NME1-NME2, NME1, NME2, MBTD1, UTP18, LOC101927274, LOC440446, CA10, C17orf112, KIF2B, TOM1L1, COX11, STXBP4, HLF, MMD, TMEM100, PCTP, ANKFN1, NOG, C17orf67, DGKE, MTVR2, TRIM25, MIR3614, COIL, SCPEP1, RNF126P1, AKAP1, MSI2, LOC101927557, LOC101927539, CCDC182, MRPS23, CUEDC1, VEZF1, SRSF1, LOC101927666, DYNLL2, OR4D1, MSX2P1, OR4D2, EPX, MKS1, LPO, MPO, TSPOAP1, TSPOAP1-AS1, MIR142, MIR4736, SUPT4H1, RNF43, HSF5, MTMR4, SEPT4-AS1, SEPT4, C17orf47, TEX14, RAD51C, PPM1E, TRIM37, SKA2, MIR454, MIR301A, PRR11, SMG8, GDPD1, YPEL2, MIR4729, LINC01476, DHX40, CLTC, PTRH2, VMP1, MIR21, TUBD1, RPS6KB1, RNFT1, TBC1D3P1-DHX40P1, LOC101927755, MIR4737, HEATR6, LOC105371849, WFDC21P, LOC653653, CA4, USP32, SCARNA20, C17orf64, APPBP2, LOC388406, PPM1D, BCAS3, LOC101927855, TBX2-AS1, TBX2, C17orf82, TBX4, NACA2, BRIP1, INTS2, MED13, TBC1D3P2, EFCAB3, METTL2A, TLK2, MRC2, MARCH10, LOC101927877, MIR633, TANC2, CYB561, ACE, KCNH6, DCAF7, TACO1, MAP3K3, LIMD2, LOC729683, STRADA, CCDC47, DDX42, FTSJ3, PSMC5, SMARCD2, TCAM1P, CSH2, GH2, CSH1, CSHL1, GH1, CD79B, SCN4A, PRR29-AS1, PRR29, ICAM2, ERN1, SNHG25, SNORD104, SNORA50C, TEX2, PECAM1, MILR1, POLG2, DDX5, MIR3064, MIR5047, CEP95, SMURF2, LOC146880, MIR6080, PLEKHM1P1, LRRC37A3, AMZ2P1, GNA13, LOC100507002, RGS9, CRAT40, AXIN2, CEP112, APOH, PRKCA, PRKCA-AS1, MIR634, CACNG5, CACNG4, CACNG1, HELZ, LOC101928021, PSMD12, PITPNC1, MIR548AA2, MIR548D2, NOL11, SNORA38B, BPTF, C17orf58, KPNA2, LINC00674, LOC440461, AMZ2, ARSG, SLC16A6, PRKAR1A, WIPI1, MIR635, FAM20A, LINC01482, ABCA8, ABCA9, ABCA9-AS1, ABCA6, MIR4524B, MIR4524A, ABCA10, PRO1804, ABCA5, MAP2K6, LINC01483, LINC01497, LINC01028, KCNJ16, KCNJ2-AS1, KCNJ2 | arr[GRCh37] 17p11.2q24.3(21138333_68911339)x3 |
| 1  CRC | Gain | 19 | p13.3 | q13.43 | 58,846 | PLPP2, MIER2, THEG, C2CD4C, SHC2, ODF3L2, MADCAM1, TPGS1, CDC34, GZMM, BSG, HCN2, POLRMT, FGF22, RNF126, FSTL3, PRSS57, PALM, MISP, PTBP1, MIR4745, PLPPR3, MIR3187, AZU1, PRTN3, ELANE, CFD, MED16, RNU6-2, R3HDM4, KISS1R, ARID3A, WDR18, GRIN3B, TMEM259, CNN2, ABCA7, ARHGAP45, POLR2E, GPX4, SBNO2, STK11, CBARP, ATP5D, MIDN, CIRBP-AS1, CIRBP, C19orf24, EFNA2, MUM1, NDUFS7, GAMT, DAZAP1, RPS15, APC2, C19orf25, PCSK4, REEP6, ADAMTSL5, PLK5, MEX3D, MBD3, UQCR11, TCF3, ONECUT3, ATP8B3, REXO1, MIR1909, LOC100288123, KLF16, ABHD17A, ADAT3, SCAMP4, CSNK1G2, CSNK1G2-AS1, BTBD2, MKNK2, MOB3A, IZUMO4, AP3D1, DOT1L, PLEKHJ1, MIR1227, MIR6789, SF3A2, AMH, MIR4321, JSRP1, OAZ1, C19orf35, LINGO3, LSM7, SPPL2B, TMPRSS9, TIMM13, LMNB2, MIR7108, LOC101928602, GADD45B, GNG7, MIR7850, DIRAS1, SLC39A3, SGTA, THOP1, ZNF554, ZNF555, ZNF556, ZNF57, ZNF77, TLE6, TLE2, AES, GNA11, GNA15, LOC100996351, S1PR4, NCLN, CELF5, NFIC, SMIM24, DOHH, FZR1, C19orf71, MFSD12, HMG20B, GIPC3, TBXA2R, CACTIN-AS1, CACTIN, PIP5K1C, TJP3, MIR1268A, APBA3, MRPL54, RAX2, MATK, ZFR2, ATCAY, NMRK2, DAPK3, MIR637, EEF2, SNORD37, PIAS4, ZBTB7A, MAP2K2, CREB3L3, SIRT6, ANKRD24, EBI3, CCDC94, SHD, TMIGD2, FSD1, STAP2, MPND, SH3GL1, CHAF1A, UBXN6, MIR4746, HDGFRP2, PLIN4, PLIN5, LRG1, SEMA6B, TNFAIP8L1, MYDGF, DPP9, DPP9-AS1, MIR7-3HG, MIR7-3, FEM1A, TICAM1, PLIN3, ARRDC5, UHRF1, MIR4747, KDM4B, PTPRS, ZNRF4, TINCR, SAFB2, SAFB, C19orf70, HSD11B1L, RPL36, LONP1, CATSPERD, PRR22, DUS3L, NRTN, FUT6, FUT3, LOC101928844, FUT5, NDUFA11, VMAC, CAPS, RANBP3, LOC100128568, RFX2, ACSBG2, MLLT1, ACER1, CLPP, ALKBH7, PSPN, GTF2F1, MIR6885, MIR6790, LOC390877, KHSRP, MIR3940, SLC25A41, SLC25A23, CRB3, DENND1C, TUBB4A, TNFSF9, CD70, TNFSF14, C3, GPR108, MIR6791, TRIP10, SH2D3A, VAV1, ADGRE1, ADGRE4P, FLJ25758, MBD3L5, MBD3L4, MBD3L2, MBD3L3, ZNF557, INSR, ARHGEF18, LOC100128573, PEX11G, C19orf45, ZNF358, MCOLN1, PNPLA6, CAMSAP3, MIR6792, XAB2, PET100, PCP2, STXBP2, RETN, MCEMP1, TRAPPC5, FCER2, CLEC4G, CD209, CLEC4M, CLEC4GP1, EVI5L, PRR36, LYPLA2P2, LRRC8E, MAP2K7, TGFBR3L, SNAPC2, CTXN1, TIMM44, ELAVL1, CCL25, FBN3, CERS4, CD320, NDUFA7, RPS28, KANK3, ANGPTL4, RAB11B-AS1, MIR4999, RAB11B, MARCH2, HNRNPM, PRAM1, ZNF414, MYO1F, ADAMTS10, ACTL9, OR2Z1, ZNF558, MBD3L1, MUC16, OR1M1, OR7G2, OR7G1, OR7G3, ZNF317, OR7D2, OR7D4, OR7E24, ZNF699, ZNF559, ZNF559-ZNF177, ZNF177, ZNF266, ZNF560, ZNF426, LOC101928238, ZNF121, ZNF561, ZNF561-AS1, ZNF562, ZNF846, FBXL12, UBL5, PIN1, OLFM2, COL5A3, RDH8, MIR5589, C3P1, C19orf66, ANGPTL6, PPAN, PPAN-P2RY11, SNORD105, SNORD105B, P2RY11, EIF3G, DNMT1, S1PR2, MIR4322, MRPL4, ICAM1, ICAM4, ICAM5, ZGLP1, FDX1L, RAVER1, ICAM3, TYK2, CDC37, MIR1181, PDE4A, KEAP1, S1PR5, ATG4D, MIR1238, KRI1, CDKN2D, AP1M2, SLC44A2, ILF3-AS1, ILF3, QTRT1, DNM2, MIR638, MIR4748, MIR199A1, MIR6793, TMED1, C19orf38, CARM1, YIPF2, C19orf52, SMARCA4, LDLR, MIR6886, SPC24, KANK2, DOCK6, LOC105372273, ANGPTL8, TSPAN16, RAB3D, TMEM205, CCDC159, PLPPR2, SWSAP1, EPOR, RGL3, CCDC151, PRKCSH, ELAVL3, ZNF653, MIR7974, ECSIT, CNN1, ELOF1, ACP5, ZNF627, ZNF833P, ZNF823, ZNF441, ZNF491, ZNF440, ZNF439, ZNF69, ZNF700, ZNF763, LOC101928464, ZNF433, ZNF878, ZNF844, ZNF788, ZNF20, ZNF625-ZNF20, ZNF625, ZNF136, LOC100289333, ZNF44, ZNF563, ZNF442, ZNF799, ZNF443, ZNF709, ZNF564, ZNF490, ZNF791, MAN2B1, WDR83, WDR83OS, DHPS, LOC105372280, FBXW9, TNPO2, SNORD135, SNORD41, C19orf43, ASNA1, BEST2, HOOK2, MIR5684, JUNB, PRDX2, RNASEH2A, RTBDN, MAST1, MIR6794, DNASE2, KLF1, GCDH, SYCE2, FARSA, CALR, MIR6515, RAD23A, GADD45GIP1, DAND5, NFIX, LYL1, TRMT1, NACC1, STX10, IER2, CACNA1A, CCDC130, MRI1, C19orf53, ZSWIM4, LOC284454, MIR24-2, MIR27A, MIR23A, MIR181C, MIR181D, NANOS3, C19orf57, CC2D1A, PODNL1, DCAF15, RFX1, RLN3, IL27RA, PALM3, LOC113230, MIR1199, C19orf67, SAMD1, PRKACA, ASF1B, LOC100507373, ADGRL1, LOC105372288, LOC101928845, ADGRE5, DDX39A, PKN1, PTGER1, GIPC1, DNAJB1, MIR639, TECR, NDUFB7, CLEC17A, ADGRE3, SNORA104, ZNF333, ADGRE2, OR7C1, OR7A5, OR7A10, OR7A17, OR7C2, SLC1A6, CCDC105, CASP14, OR1I1, SYDE1, ILVBL, NOTCH3, MIR6795, EPHX3, BRD4, AKAP8, AKAP8L, WIZ, MIR1470, RASAL3, PGLYRP2, CYP4F22, CYP4F8, CYP4F3, CYP4F12, OR10H2, OR10H3, CYP4F24P, OR10H5, OR10H1, UCA1, LOC102724279, CYP4F2, CYP4F11, OR10H4, LINC00661, LINC00905, TPM4, RAB8A, HSH2D, CIB3, FAM32A, AP1M1, KLF2, EPS15L1, CALR3, C19orf44, CHERP, SLC35E1, MED26, SMIM7, TMEM38A, NWD1, SIN3B, F2RL3, CPAMD8, HAUS8, MYO9B, USE1, OCEL1, NR2F6, USHBP1, BABAM1, ANKLE1, ABHD8, MRPL34, DDA1, ANO8, GTPBP3, PLVAP, BST2, BISPR, MVB12A, TMEM221, NXNL1, SLC27A1, PGLS, FAM129C, COLGALT1, UNC13A, MAP1S, FCHO1, B3GNT3, INSL3, JAK3, RPL18A, SNORA68, SLC5A5, CCDC124, KCNN1, ARRDC2, IL12RB1, MAST3, PIK3R2, IFI30, MPV17L2, RAB3A, LOC102725254, PDE4C, LOC729966, KIAA1683, JUND, MIR3188, LSM4, PGPEP1, GDF15, MIR3189, LRRC25, SSBP4, ISYNA1, ELL, FKBP8, KXD1, UBA52, C19orf60, CRLF1, TMEM59L, KLHL26, CRTC1, COMP, UPF1, GDF1, CERS1, COPE, DDX49, HOMER3, LOC102724360, SUGP2, ARMC6, SLC25A42, TMEM161A, MEF2B, BORCS8-MEF2B, BORCS8, RFXANK, NR2C2AP, NCAN, HAPLN4, TM6SF2, SUGP1, MAU2, GATAD2A, MIR640, TSSK6, NDUFA13, YJEFN3, CILP2, PBX4, LPAR2, GMIP, ATP13A1, ZNF101, ZNF14, LINC00663, ZNF506, ZNF253, ZNF93, ZNF682, ZNF90, ZNF486, MIR1270, ZNF826P, ZNF737, ZNF626, ZNF85, ZNF430, ZNF714, ZNF431, ZNF708, ZNF738, ZNF493, LINC00664, ZNF429, ZNF100, LOC641367, ZNF43, ZNF208, ZNF257, ZNF676, ZNF729, ZNF98, LOC101929124, LINC01233, GOLGA2P9, LOC100996349, LOC374890, ZNF492, ZNF99, ZNF728, LOC101929164, LOC101929144, ZNF730, ZNF724P, IPO5P1, ZNF91, LINC01224, ZNF675, ZNF681, RPSAP58, ZNF726, ZNF254, HAVCR1P1, LINC00662, LOC101927151, LOC100420587, LOC102724908, LINC00906, LOC102724958, LINC01532, UQCRFS1, LOC284395, VSTM2B, POP4, PLEKHF1, C19orf12, CCNE1, URI1, ZNF536, TSHZ3, THEG5, LINC01533, LOC101927411, ZNF507, LOC400684, DPY19L3, PDCD5, ANKRD27, RGS9BP, NUDT19, TDRD12, SLC7A9, CEP89, FAAP24, RHPN2, GPATCH1, WDR88, LRP3, SLC7A10, CEBPA, CEBPA-AS1, CEBPG, PEPD, CHST8, KCTD15, LSM14A, KIAA0355, GPI, PDCD2L, UBA2, WTIP, SCGB1B2P, SCGB2B2, SCGB2B3P, ZNF302, ZNF181, ZNF599, LOC400685, LINC00904, ZNF30-AS1, ZNF30, ZNF792, GRAMD1A, SCN1B, HPN, HPN-AS1, FXYD3, MIR6887, LGI4, FXYD1, FXYD7, FXYD5, FAM187B, LSR, USF2, HAMP, MAG, CD22, MIR5196, FFAR1, FFAR3, LINC01531, FFAR2, KRTDAP, DMKN, SBSN, GAPDHS, TMEM147-AS1, TMEM147, ATP4A, LOC102723617, HAUS5, RBM42, ETV2, COX6B1, UPK1A, UPK1A-AS1, ZBTB32, KMT2B, IGFLR1, U2AF1L4, PSENEN, LIN37, HSPB6, PROSER3, ARHGAP33, LINC01529, PRODH2, NPHS1, KIRREL2, APLP1, NFKBID, HCST, TYROBP, LRFN3, LOC105372383, SDHAF1, SYNE4, ALKBH6, LOC101927572, CLIP3, THAP8, WDR62, OVOL3, POLR2I, TBCB, CAPNS1, COX7A1, ZNF565, ZNF146, LOC100134317, LINC00665, ZFP14, ZFP82, LOC644189, ZNF566, LOC728752, ZNF260, ZNF529, ZNF529-AS1, ZNF382, ZNF461, LINC01534, ZNF567, ZNF850, LOC728485, ZNF790-AS1, ZNF790, ZNF345, ZNF829, ZNF568, ZNF420, ZNF585A, ZNF585B, ZNF383, LINC01535, LOC284412, HKR1, ZNF527, ZNF569, ZNF570, ZNF793-AS1, ZNF793, ZNF571-AS1, ZNF540, ZNF571, ZFP30, ZNF781, ZNF607, ZNF573, LOC644554, LOC100631378, WDR87, SIPA1L3, DPF1, PPP1R14A, SPINT2, YIF1B, C19orf33, KCNK6, CATSPERG, PSMD8, GGN, SPRED3, FAM98C, RASGRP4, RYR1, MAP4K1, LOC105372397, EIF3K, ACTN4, CAPN12, LGALS7, LGALS7B, LGALS4, ECH1, HNRNPL, RINL, SIRT2, NFKBIB, CCER2, SARS2, MRPS12, FBXO17, FBXO27, ACP7, PAK4, NCCRP1, SYCN, IFNL3, IFNL4, IFNL2, IFNL1, LRFN1, GMFG, SAMD4B, PAF1, MED29, ZFP36, MIR4530, PLEKHG2, RPS16, SUPT5H, TIMM50, DLL3, SELV, EID2B, EID2, LGALS13, LOC100129935, LGALS16, LGALS17A, LGALS14, CLC, LEUTX, DYRK1B, MIR6719, FBL, FCGBP, PSMC4, ZNF546, ZNF780B, ZNF780A, MAP3K10, TTC9B, CNTD2, AKT2, MIR641, C19orf47, PLD3, MIR6796, HIPK4, PRX, SERTAD1, SERTAD3, BLVRB, SPTBN4, SHKBP1, LTBP4, NUMBL, ADCK4, ITPKC, C19orf54, SNRPA, MIA, MIA-RAB4B, RAB4B, RAB4B-EGLN2, EGLN2, CYP2A6, CYP2A7, CYP2G1P, CYP2B7P, CYP2B6, CYP2A13, CYP2F1, CYP2S1, AXL, HNRNPUL1, CCDC97, TGFB1, B9D2, TMEM91, EXOSC5, BCKDHA, B3GNT8, ATP5SL, ERICH4, PCAT19, LINC01480, CEACAM21, CEACAM4, CEACAM7, CEACAM5, CEACAM6, CEACAM3, LYPD4, DMRTC2, RPS19, MIR6797, CD79A, ARHGEF1, LOC100505585, RABAC1, ATP1A3, GRIK5, ZNF574, POU2F2, LOC100505622, MIR4323, DEDD2, ZNF526, GSK3A, ERF, CIC, PAFAH1B3, PRR19, TMEM145, MEGF8, MIR8077, CNFN, LOC101930071, LIPE-AS1, LIPE, CXCL17, CEACAM1, CEACAM8, PSG3, PSG8, LOC100289650, PSG10P, PSG1, PSG6, PSG7, PSG11, PSG2, PSG5, PSG4, LOC284344, PSG9, PRG1, CD177, TEX101, LYPD3, PHLDB3, ETHE1, ZNF575, XRCC1, PINLYP, IRGQ, ZNF576, ZNF428, SRRM5, CADM4, PLAUR, IRGC, SMG9, KCNN4, LYPD5, ZNF283, ZNF404, LOC100505715, ZNF45, ZNF221, ZNF155, LOC101928063, ZNF230, ZNF222, ZNF223, ZNF284, ZNF224, LOC100379224, ZNF225, ZNF234, ZNF226, ZNF227, ZNF233, ZNF235, ZNF112, ZNF285, ZNF229, ZNF180, CEACAM20, CEACAM22P, IGSF23, PVR, MIR4531, CEACAM19, CEACAM16, BCL3, MIR8085, CBLC, BCAM, NECTIN2, TOMM40, APOE, APOC1, APOC1P1, APOC4, APOC4-APOC2, APOC2, CLPTM1, RELB, CLASRP, ZNF296, GEMIN7, LOC105372419, PPP1R37, NKPD1, TRAPPC6A, BLOC1S3, EXOC3L2, MARK4, CKM, KLC3, ERCC2, PPP1R13L, CD3EAP, ERCC1, MIR6088, FOSB, RTN2, PPM1N, VASP, OPA3, GPR4, EML2, MIR330, EML2-AS1, GIPR, MIR642A, MIR642B, SNRPD2, QPCTL, FBXO46, BHMG1, SIX5, DMPK, DMWD, RSPH6A, SYMPK, FOXA3, IRF2BP1, MYPOP, NANOS2, NOVA2, CCDC61, MIR769, PGLYRP1, IGFL4, LOC400706, IGFL3, IGFL2, LOC645553, LOC93429, IGFL1, HIF3A, PPP5C, CCDC8, PNMAL1, PPP5D1, PNMAL2, CALM3, PTGIR, GNG8, DACT3, DACT3-AS1, PRKD2, MIR320E, STRN4, FKRP, SLC1A5, SNAR-E, AP2S1, ARHGAP35, NPAS1, TMEM160, ZC3H4, SAE1, BBC3, MIR3190, MIR3191, CCDC9, INAFM1, C5AR1, C5AR2, DHX34, MEIS3, SLC8A2, KPTN, NAPA-AS1, NAPA, ZNF541, GLTSCR1, EHD2, GLTSCR2, SNORD23, GLTSCR2-AS1, SEPW1, TPRX1, CRX, SULT2A1, SNAR-A13, SNAR-A12, SNAR-C2, SNAR-C1, SNAR-C5, SNAR-A1, SNAR-A2, SNAR-A10, SNAR-A3, SNAR-A8, SNAR-A4, SNAR-A11, SNAR-A7, SNAR-A9, SNAR-A5, SNAR-A6, SNAR-A14, SNAR-C4, SNAR-C3, BSPH1, ELSPBP1, CABP5, PLA2G4C, PLA2G4C-AS1, LIG1, C19orf68, CARD8, CARD8-AS1, ZNF114, CCDC114, EMP3, TMEM143, SYNGR4, KDELR1, GRIN2D, GRWD1, KCNJ14, CYTH2, LMTK3, SULT2B1, FAM83E, SPACA4, RPL18, SPHK2, DBP, CA11, SEC1P, NTN5, FUT2, LOC105447645, MAMSTR, RASIP1, IZUMO1, FUT1, FGF21, BCAT2, HSD17B14, PLEKHA4, PPP1R15A, TULP2, NUCB1, NUCB1-AS1, DHDH, BAX, FTL, GYS1, RUVBL2, MIR6798, LHB, LOC101059948, CGB3, SNAR-G2, CGB2, CGB1, SNAR-G1, CGB5, CGB8, CGB7, NTF4, KCNA7, SNRNP70, LIN7B, C19orf73, PPFIA3, HRC, TRPM4, SLC6A16, MIR4324, CD37, TEAD2, DKKL1, LOC101928295, CCDC155, PTH2, GFY, SLC17A7, PIH1D1, ALDH16A1, FLT3LG, RPL13A, RPL13AP5, SNORD32A, SNORD33, SNORD34, SNORD35A, RPS11, SNORD35B, MIR150, FCGRT, RCN3, NOSIP, PRRG2, PRR12, RRAS, SCAF1, IRF3, BCL2L12, PRMT1, MIR5088, ADM5, CPT1C, TSKS, AP2A1, MIR6799, FUZ, MED25, MIR6800, PTOV1-AS1, PTOV1, MIR4749, PTOV1-AS2, PNKP, AKT1S1, TBC1D17, MIR4750, IL4I1, NUP62, ATF5, MIR4751, SIGLEC11, SIGLEC16, VRK3, ZNF473, FLJ26850, SNAR-B1, SNAR-B2, SNAR-D, IZUMO2, MYH14, KCNC3, NAPSB, NAPSA, NR1H2, POLD1, SPIB, MYBPC2, FAM71E1, EMC10, JOSD2, ASPDH, LRRC4B, SNAR-F, SYT3, C19orf81, SHANK1, CLEC11A, GPR32, LOC105372440, ACPT, C19orf48, SNORD88B, SNORD88A, SNORD88C, MGC45922, KLK1, KLK15, LOC105372441, KLK3, KLK2, KLKP1, KLK4, KLK5, KLK6, KLK7, KLK8, KLK9, KLK10, KLK11, KLK12, KLK13, KLK14, CTU1, SIGLEC9, SIGLEC7, LOC101928517, SIGLEC17P, MIR8074, CD33, SIGLECL1, LOC105372444, IGLON5, VSIG10L, ETFB, CLDND2, NKG7, LIM2, C19orf84, SIGLEC10, LOC100129083, SIGLEC8, CEACAM18, SIGLEC12, SIGLEC6, ZNF175, LINC01530, SIGLEC5, SIGLEC14, SPACA6P-AS, SPACA6, MIR99B, MIRLET7E, MIR125A, HAS1, FPR1, FPR2, FPR3, ZNF577, ZNF649-AS1, ZNF649, ZNF613, ZNF350-AS1, ZNF350, ZNF615, ZNF614, ZNF432, ZNF841, ZNF616, ZNF836, PPP2R1A, MIR6801, ZNF766, MIR643, ZNF480, ZNF610, ZNF880, ZNF528-AS1, ZNF528, ZNF534, ZNF578, ZNF808, ZNF701, ZNF137P, ZNF83, ZNF611, ZNF600, ZNF28, ZNF468, ZNF320, ZNF888, ZNF321P, ZNF816-ZNF321P, ZNF816, ZNF702P, ERVV-1, ERVV-2, ZNF160, ZNF415, ZNF347, ZNF665, ZNF818P, ZNF677, VN1R2, VN1R4, FAM90A27P, BIRC8, ZNF845, ZNF525, ZNF765, TPM3P9, ZNF761, ZNF813, ZNF331, LOC284379, DPRX, MIR512-2, MIR512-1, MIR1323, MIR498, MIR520E, MIR515-2, MIR515-1, MIR519E, MIR520F, MIR519C, MIR1283-1, MIR520A, MIR526B, MIR519B, MIR525, MIR523, MIR518F, MIR520B, MIR518B, MIR526A1, MIR520C, MIR518C, MIR524, MIR517A, MIR519D, MIR521-2, MIR520D, MIR517B, MIR520G, MIR516B2, MIR526A2, MIR518E, MIR518A1, MIR518D, MIR516B1, MIR518A2, MIR517C, MIR520H, MIR521-1, MIR522, MIR519A1, MIR527, MIR516A1, MIR1283-2, MIR516A2, MIR519A2, MIR371A, MIR371B, MIR372, MIR373, NLRP12, MYADM, PRKCG, CACNG7, CACNG8, MIR935, CACNG6, VSTM1, TARM1, OSCAR, NDUFA3, TFPT, PRPF31, CNOT3, LENG1, TMC4, MBOAT7, TSEN34, RPS9, LILRB3, LILRA6, LILRB5, LILRB2, MIR4752, LILRA3, LILRA5, LILRA4, LAIR1, TTYH1, LENG8-AS1, LENG8, LENG9, CDC42EP5, LAIR2, KIR3DX1, LILRA2, LILRA1, LILRB1, MIR8061, LILRB4, LILRP2, KIR3DL3, KIR2DL3, LOC101928804, KIR2DL1, KIR2DL4, KIR3DL1, KIR2DS4, KIR3DL2, FCAR, NCR1, NLRP7, NLRP2, GP6, RDH13, EPS8L1, PPP1R12C, MIR7975, TNNT1, TNNI3, DNAAF3, SYT5, PTPRH, TMEM86B, PPP6R1, MIR6804, MIR6802, MIR6803, HSPBP1, BRSK1, TMEM150B, KMT5C, COX6B2, FAM71E2, IL11, TMEM190, TMEM238, RPL28, MIR6805, UBE2S, SHISA7, ISOC2, ZNF628, NAT14, SSC5D, SBK2, SBK3, ZNF579, FIZ1, ZNF524, ZNF865, ZNF784, ZNF580, ZNF581, CCDC106, U2AF2, EPN1, NLRP9, RFPL4A, RFPL4AL1, NLRP11, NLRP4, NLRP13, NLRP8, NLRP5, LOC101928886, ZNF787, ZNF444, GALP, ZSCAN5B, ZSCAN5A, ZNF542P, ZNF582, ZNF582-AS1, ZNF583, ZNF667, ZNF667-AS1, ZNF471, ZFP28, ZNF470, ZNF71, SMIM17, ZNF835, ZIM2-AS1, ZIM2, PEG3, PEG3-AS1, MIMT1, USP29, ZIM3, DUXA, ZNF264, AURKC, ZNF805, LOC105372476, ZNF460, ZNF543, ZNF304, ZNF547, TRAPPC2B, ZNF548, ZNF17, ZNF749, VN1R1, ZNF772, ZNF419, ZNF773, ZNF549, ZNF550, ZNF416, ZIK1, ZNF530, ZNF134, ZNF211, ZSCAN4, ZNF551, ZNF154, ZNF671, ZNF776, ZNF586, ZNF552, FKBP1AP1, ZNF587B, ZNF587, ZNF814, ZNF417, ZNF418, ZNF256, C19orf18, ZNF606, LOC100128398, ZSCAN1, ZNF135, ZSCAN18, ZNF329, ZNF274, ZNF544, ZNF8, ZSCAN22, MIR6806, A1BG, A1BG-AS1, ZNF497, LOC105372483, ZNF837, MIR4754, RPS5, RNF225, ZNF584, ZNF132, ZNF324B, ZNF324, ZNF446, SLC27A5, ZBTB45, TRIM28, MIR6807, CHMP2A, UBE2M, MZF1-AS1, MZF1, CENPBD1P1 | arr[GRCh37] 19p13.3q13.43(247231_59093239)x3 |
| 1  CRC | Gain | 20 | p13 | q13.33 | 62,843 | DEFB125, DEFB126, DEFB127, DEFB128, DEFB129, DEFB132, C20orf96, ZCCHC3, NRSN2-AS1, SOX12, NRSN2, TRIB3, RBCK1, TBC1D20, CSNK2A1, TCF15, SRXN1, SCRT2, SLC52A3, FAM110A, ANGPT4, RSPO4, PSMF1, LOC105372493, TMEM74B, C20orf202, RAD21L1, SNPH, SDCBP2, FKBP1A-SDCBP2, SDCBP2-AS1, FKBP1A, MIR6869, NSFL1C, SIRPB2, SIRPD, SIRPB1, SIRPG, SIRPG-AS1, LOC100289473, SIRPA, LOC727993, PDYN, STK35, LOC388780, TGM3, TGM6, SNRPB, SNORD119, ZNF343, TMC2, NOP56, MIR1292, SNORD110, SNORA51, SNORD86, SNORD56, SNORD57, IDH3B, EBF4, CPXM1, C20orf141, TMEM239, PCED1A, VPS16, PTPRA, GNRH2, MRPS26, OXT, AVP, UBOX5-AS1, UBOX5, FASTKD5, LZTS3, DDRGK1, ITPA, SLC4A11, C20orf194, ATRN, GFRA4, ADAM33, SIGLEC1, HSPA12B, C20orf27, SPEF1, CENPB, CDC25B, LOC101929125, AP5S1, MAVS, PANK2, MIR103A2, MIR103B2, RNF24, SMOX, LINC01433, ADRA1D, PRNP, PRND, PRNT, RASSF2, SLC23A2, TMEM230, PCNA, PCNA-AS1, CDS2, PROKR2, LINC00658, LOC643406, LINC00654, LOC101929207, GPCPD1, C20orf196, CHGB, TRMT6, MCM8, MCM8-AS1, CRLS1, LRRN4, FERMT1, CASC20, BMP2, LINC01428, LOC101929288, LOC101929312, MIR8062, HAO1, TMX4, PLCB1, PLCB4, LAMP5-AS1, LAMP5, PAK5, LOC101929371, SNAP25-AS1, ANKEF1, SNAP25, MKKS, SLX4IP, JAG1, MIR6870, LOC101929395, LOC101929413, LOC339593, LINC00687, BTBD3, LOC101929486, LOC102606466, LOC100505515, SPTLC3, ISM1, ISM1-AS1, TASP1, ESF1, NDUFAF5, SEL1L2, MACROD2, FLRT3, MACROD2-IT1, MACROD2-AS1, LOC613266, KIF16B, SNRPB2, OTOR, PCSK2, BFSP1, DSTN, RRBP1, BANF2, SNX5, SNORD17, MGME1, OVOL2, PET117, KAT14, ZNF133, LINC00851, DZANK1, POLR3F, MIR3192, RBBP9, SEC23B, LINC00493, DTD1, LOC101929526, LINC00652, LOC100270804, C20orf78, SCP2D1, SLC24A3, LOC100130264, RIN2, NAA20, CRNKL1, CFAP61, INSM1, RALGAPA2, KIZ, KIZ-AS1, XRN2, NKX2-4, NKX2-2, LOC101929625, LOC101929608, PAX1, LINC01432, LINC01427, LOC284788, LINC00261, FOXA2, LINC01384, SSTR4, THBD, CD93, LINC00656, NXT1, LINC01431, GZF1, NAPB, CSTL1, CST11, CST8, CST13P, CST9L, CST9, CST3, CST4, CST1, CST2, CST5, GGTLC1, FLJ33581, SYNDIG1, CST7, APMAP, ACSS1, VSX1, LOC284798, LOC101926889, ENTPD6, PYGB, ABHD12, GINS1, NINL, NANP, ZNF337-AS1, ZNF337, LOC105372582, LOC105379511, FAM182B, LOC101926935, LOC101926955, LOC100134868, FAM182A, NCOR1P1, MIR663AHG, MIR663A, LINC01598, FRG1BP, FRG1DP, MLLT10P1, DEFB115, DEFB116, DEFB118, DEFB119, DEFB121, DEFB122, DEFB123, DEFB124, REM1, LINC00028, HM13, HM13-AS1, ID1, MIR3193, COX4I2, BCL2L1, ABALON, TPX2, MYLK2, FOXS1, DUSP15, TTLL9, PDRG1, XKR7, MIR7641-2, CCM2L, HCK, TM9SF4, TSPY26P, PLAGL2, POFUT1, MIR1825, KIF3B, ASXL1, NOL4L, LOC101929698, LOC149950, C20orf203, COMMD7, DNMT3B, MAPRE1, SUN5, BPIFB2, BPIFB6, BPIFB3, BPIFB4, BPIFA2, BPIFA4P, BPIFA3, BPIFA1, BPIFB1, CDK5RAP1, SNTA1, CBFA2T2, NECAB3, C20orf144, ACTL10, E2F1, PXMP4, ZNF341, ZNF341-AS1, CHMP4B, RALY-AS1, RALY, MIR4755, EIF2S2, ASIP, AHCY, ITCH, MIR644A, DYNLRB1, MAP1LC3A, PIGU, TP53INP2, NCOA6, HMGB3P1, GGT7, ACSS2, GSS, MYH7B, MIR499A, MIR499B, TRPC4AP, EDEM2, PROCR, MMP24, MMP24-AS1, EIF6, FAM83C-AS1, FAM83C, UQCC1, GDF5, MIR1289-1, CEP250, C20orf173, ERGIC3, FER1L4, SPAG4, CPNE1, RBM12, NFS1, ROMO1, RBM39, PHF20, SCAND1, CNBD2, NORAD, EPB41L1, AAR2, DLGAP4, DLGAP4-AS1, MYL9, TGIF2, TGIF2-C20orf24, C20orf24, SLA2, NDRG3, DSN1, SOGA1, TLDC2, SAMHD1, RBL1, MROH8, RPN2, GHRH, MANBAL, SRC, BLCAP, NNAT, LINC00489, LOC100287792, CTNNBL1, VSTM2L, TTI1, RPRD1B, TGM2, KIAA1755, LOC149684, BPI, LBP, SNHG17, SNORA71B, SNORA71A, SNORA71C, SNORA71D, SNHG11, SNORA71E, SNORA60, RALGAPB, MIR548O2, ADIG, ARHGAP40, SLC32A1, ACTR5, PPP1R16B, FAM83D, DHX35, LOC339568, LINC01370, MAFB, LOC100128988, TOP1, PLCG1-AS1, PLCG1, MIR6871, ZHX3, LPIN3, EMILIN3, CHD6, PTPRT, LOC101927159, SRSF6, L3MBTL1, SGK2, IFT52, MYBL2, GTSF1L, LOC105372626, TOX2, JPH2, OSER1, OSER1-AS1, GDAP1L1, FITM2, R3HDML, HNF4A, HNF4A-AS1, MIR3646, LINC01430, LINC01620, TTPAL, SERINC3, PKIG, ADA, LINC01260, KCNK15-AS1, WISP2, KCNK15, RIMS4, YWHAB, PABPC1L, TOMM34, STK4-AS1, STK4, KCNS1, WFDC5, WFDC12, PI3, SEMG1, SEMG2, SLPI, MATN4, RBPJL, SDC4, SYS1, SYS1-DBNDD2, TP53TG5, DBNDD2, PIGT, MIR6812, WFDC2, SPINT3, WFDC6, EPPIN-WFDC6, EPPIN, WFDC8, WFDC9, WFDC10A, WFDC11, WFDC10B, WFDC13, MIR3617, SPINT4, WFDC3, DNTTIP1, UBE2C, TNNC2, SNX21, ACOT8, ZSWIM3, ZSWIM1, SPATA25, NEURL2, CTSA, PLTP, PCIF1, ZNF335, MMP9, SLC12A5, NCOA5, CD40, CDH22, SLC35C2, ELMO2, ZNF663P, MKRN7P, ZNF334, OCSTAMP, SLC13A3, TP53RK, SLC2A10, EYA2, MIR3616, ZMYND8, LOC100131496, LOC101927377, NCOA3, SULF2, LINC01522, LINC01523, LINC00494, PREX1, ARFGEF2, CSE1L-AS1, CSE1L, STAU1, DDX27, ZNFX1, ZFAS1, SNORD12C, SNORD12B, SNORD12, KCNB1, PTGIS, B4GALT5, SLC9A8, SPATA2, LOC105372653, RNF114, SNAI1, TRERNA1, UBE2V1, TMEM189-UBE2V1, TMEM189, LINC01273, CEBPB-AS1, CEBPB, LINC01272, LINC01270, LINC01271, PTPN1, MIR645, FAM65C, MIR1302-5, LOC100506175, PARD6B, BCAS4, ADNP, ADNP-AS1, DPM1, MOCS3, KCNG1, NFATC2, MIR3194, ATP9A, SALL4, LINC01429, ZFP64, LINC01524, TSHZ2, LOC101927770, ZNF217, LOC105372672, SUMO1P1, BCAS1, MIR4756, CYP24A1, PFDN4, DOK5, LINC01441, LINC01440, CBLN4, MC3R, FAM210B, AURKA, CSTF1, CASS4, RTFDC1, GCNT7, FAM209A, FAM209B, LOC105372682, TFAP2C, BMP7, BMP7-AS1, MIR4325, SPO11, RAE1, MTRNR2L3, RBM38, CTCFL, PCK1, ZBP1, PMEPA1, NKILA, MIR4532, C20orf85, ANKRD60, PPP4R1L, RAB22A, VAPB, APCDD1L, APCDD1L-AS1, LOC79160, STX16, STX16-NPEPL1, NPEPL1, LOC105372695, MIR296, MIR298, GNAS-AS1, GNAS, LOC101927932, NELFCD, CTSZ, TUBB1, ATP5E, SLMO2-ATP5E, PRELID3B, ZNF831, EDN3, PHACTR3, LOC100506384, SYCP2, FAM217B, PPP1R3D, CDH26, C20orf197, LOC729296, MIR646HG, MIR646, LOC101928048, MIR4533, MIR548AG2, LOC100506470, CDH4, MIR1257, TAF4, MIR3195, LSM14B, PSMA7, SS18L1, MTG2, HRH3, OSBPL2, ADRM1, LAMA5, MIR4758, LAMA5-AS1, RPS21, CABLES2, RBBP8NL, GATA5, C20orf166-AS1, MIR1-1HG, MIR1-1, MIR133A2, SLCO4A1, SLCO4A1-AS1, NTSR1, LINC00659, MRGBP, OGFR-AS1, OGFR, COL9A3, TCFL5, DPH3P1, DIDO1, GID8, SLC17A9, BHLHE23, LOC63930, LINC00029, LINC01056, HAR1B, HAR1A, MIR124-3, YTHDF1, BIRC7, MIR3196, NKAIN4, FLJ16779, ARFGAP1, MIR4326, COL20A1, CHRNA4, LOC100130587, KCNQ2, EEF1A2, PPDPF, PTK6, SRMS, FNDC11, HELZ2, GMEB2, LOC100505771, STMN3, RTEL1, RTEL1-TNFRSF6B, TNFRSF6B, ARFRP1, ZGPAT, LIME1, SLC2A4RG, ZBTB46, ZBTB46-AS1, ABHD16B, TPD52L2, DNAJC5, MIR941-1, MIR941-4, MIR941-3, MIR941-2, MIR941-5, UCKL1, MIR1914, MIR647, UCKL1-AS1, ZNF512B, SAMD10, PRPF6, LINC00176, SOX18, TCEA2, RGS19, MIR6813, OPRL1, LKAAEAR1, NPBWR2, MYT1, PCMTD2 | arr[GRCh37] 20p13q13.33(69093_62912463)x3 |
| 1  CRC | Loss | 5 | q11.2 | q35.3 | 126,097 | DHX29, SKIV2L2, PLPP1, MIR5687, RNF138P1, SLC38A9, DDX4, IL31RA, IL6ST, FLJ31104, ANKRD55, LOC102467147, C5orf67, MAP3K1, SETD9, MIER3, GPBP1, ACTBL2, LINCR-0003, LOC101928505, LOC101928539, LOC101928569, PLK2, GAPT, MIR548AE2, LOC101928600, RAB3C, PDE4D, PART1, DEPDC1B, ELOVL7, ERCC8, NDUFAF2, SMIM15, CTC-436P18.1, ZSWIM6, C5orf64, LOC101928651, LOC100506526, KIF2A, DIMT1, IPO11, LRRC70, IPO11-LRRC70, HTR1A, RNF180, RGS7BP, FAM159B, SREK1IP1, CWC27, ADAMTS6, CENPK, PPWD1, TRIM23, TRAPPC13, SGTB, NLN, ERBIN, LOC100303749, SREK1, LOC101928769, MAST4, LOC101928794, CD180, LOC101928858, LOC102467655, PIK3R1, LOC101928885, SLC30A5, CCNB1, CENPH, MRPS36, CDK7, CCDC125, AK6, TAF9, RAD17, MARVELD2, LOC101928924, OCLN, GTF2H2C, GTF2H2C_2, GUSBP3, SERF1A, SERF1B, SMN1, SMN2, SMA4, GTF2H2B, SMA5, LOC441081, GUSBP9, NAIP, GTF2H2, LOC647859, LOC102724392, PMCHL2, BDP1, MCCC2, CARTPT, MAP1B, MIR4803, MRPS27, PTCD2, ZNF366, LOC102503427, LOC102477328, TNPO1, MIR4804, FCHO2, TMEM171, LOC105379030, TMEM174, LOC340090, FOXD1, LINC01386, BTF3, ANKRA2, UTP15, ARHGEF28, LINC01335, LINC01333, LINC01331, ENC1, HEXB, GFM2, NSA2, FAM169A, LOC441086, GCNT4, LINC01336, ANKRD31, HMGCR, COL4A3BP, POLK, ANKDD1B, POC5, SV2C, IQGAP2, LOC101929109, F2RL2, NCRUPAR, F2R, F2RL1, S100Z, CRHBP, AGGF1, ZBED3, SNORA47, ZBED3-AS1, PDE8B, WDR41, OTP, TBCA, LOC101929154, AP3B1, SCAMP1-AS1, SCAMP1, LHFPL2, ARSB, DMGDH, BHMT2, BHMT, JMY, HOMER1, PAPD4, CMYA5, LINC01455, MTX3, THBS4, CTD-2201I18.1, SERINC5, LOC644936, SPZ1, CRSP8P, ZFYVE16, FAM151B, ANKRD34B, LINC01337, DHFR, MTRNR2L2, MSH3, RASGRF2-AS1, RASGRF2, RNU5E-1, RNU5D-1, CKMT2, CKMT2-AS1, ZCCHC9, ACOT12, SSBP2, ATG10, RPS23, ATP6AP1L, MIR3977, LINC01338, TMEM167A, SCARNA18, XRCC4, VCAN, LOC105379054, HAPLN1, EDIL3, NBPF22P, COX7C, MIR3607, LOC100505878, MIR4280, LOC101929380, LOC55338, RASA1, LOC644285, CCNH, TMEM161B, TMEM161B-AS1, LOC102546226, LINC00461, MIR9-2, MEF2C, MEF2C-AS1, MIR3660, LINC01339, CETN3, LOC731157, MBLAC2, POLR3G, LYSMD3, ADGRV1, LUCAT1, ARRDC3, ARRDC3-AS1, NR2F1-AS1, NR2F1, FAM172A, MIR2277, POU5F2, KIAA0825, SLF1, MCTP1, FAM81B, TTC37, ARSK, GPR150, RFESD, SPATA9, RHOBTB3, GLRX, LINC01554, ELL2, LOC101929710, MIR583, PCSK1, CAST, ERAP1, ERAP2, LNPEP, LIX1, RIOK2, LINC01340, RGMB, RGMB-AS1, CHD1, LOC100289230, CTD-2151A2.1, LOC100133050, FAM174A, ST8SIA4, MIR548P, SLCO4C1, SLCO6A1, LINC00492, LINC00491, PAM, GIN1, PPIP5K2, C5orf30, LOC102467212, NUDT12, RAB9BP1, LOC102467213, EFNA5, FBXL17, LINC01023, FER, PJA2, MAN2A1, LOC100289673, TMEM232, MIR548F3, SLC25A46, TSLP, WDR36, CAMK4, STARD4, STARD4-AS1, NREP, NREP-AS1, EPB41L4A-AS1, SNORA13, EPB41L4A, LOC101927023, EPB41L4A-AS2, LOC102467214, LOC102467216, APC, SRP19, REEP5, DCP2, MCC, TSSK1B, YTHDC2, KCNN2, LOC101927078, LOC101927059, TRIM36, PGGT1B, CCDC112, FEM1C, TICAM2, TMED7-TICAM2, LOC101927100, TMED7, LOC102467217, CDO1, ATG12, AP3S1, LVRN, ARL14EPL, COMMD10, LOC101927190, SEMA6A, SEMA6A-AS1, LOC102467223, LINC00992, LOC102467224, HNCAT21, HRAT56, LOC102467225, DTWD2, MIR1244-1, MIR1244-4, MIR1244-3, MIR1244-2, LOC105379143, DMXL1, MIR5706, TNFAIP8, HSD17B4, FAM170A, PRR16, LOC102467226, FTMT, SRFBP1, LOX, ZNF474, LOC100505841, SNCAIP, MGC32805, LOC101927357, LOC101927379, SNX2, SNX24, PPIC, PRDM6, CEP120, CSNK1G3, LINC01170, ZNF608, LOC101927421, LOC101927460, LOC102546228, LOC101927488, GRAMD3, ALDH7A1, PHAX, TEX43, LOC102723557, LMNB1, MARCH3, C5orf63, MEGF10, PRRC1, CTXN3, CCDC192, LINC01184, SLC12A2, FBN2, SLC27A6, ISOC1, MIR4633, MIR4460, ADAMTS19-AS1, ADAMTS19, KIAA1024L, CHSY3, HINT1, LYRM7, CDC42SE2, RAPGEF6, FNIP1, MEIKIN, ACSL6, IL3, CSF2, P4HA2-AS1, P4HA2, MIR6830, PDLIM4, SLC22A4, LOC553103, MIR3936, SLC22A5, C5orf56, IRF1, IL5, RAD50, TH2LCRR, IL13, IL4, LOC105379176, KIF3A, CCNI2, SEPT8, SOWAHA, SHROOM1, GDF9, UQCRQ, LEAP2, AFF4, ZCCHC10, HSPA4, FSTL4, MIR1289-2, WSPAR, C5orf15, LOC105379183, VDAC1, TCF7, SKP1, PPP2CA, MIR3661, CDKL3, UBE2B, CDKN2AIPNL, LOC102546229, LOC101927934, JADE2, SAR1B, SEC24A, CAMLG, DDX46, C5orf24, TXNDC15, PCBD2, MIR4461, CATSPER3, PITX1, C5orf66, C5orf66-AS1, C5orf66-AS2, H2AFY, DCANP1, TIFAB, NEUROG1, CXCL14, LOC340074, MIR5692C1, SLC25A48, IL9, FBXL21, LECT2, TGFBI, VTRNA2-1, SMAD5-AS1, SMAD5, LOC389332, TRPC7, TRPC7-AS2, MIR4454, SPOCK1, LOC105379192, KLHL3, MIR874, HNRNPA0, NPY6R, MYOT, PKD2L2, FAM13B, LOC100130172, WNT8A, NME5, BRD8, KIF20A, CDC23, GFRA3, CDC25C, FAM53C, KDM3B, REEP2, EGR1, ETF1, HSPA9, SNORD63, LOC105379194, CTNNA1, LRRTM2, SIL1, SNHG4, MATR3, SNORA74A, PAIP2, SLC23A1, MZB1, PROB1, SPATA24, DNAJC18, ECSCR, TMEM173, UBE2D2, CXXC5, LOC101929696, PSD2, NRG2, LINC01024, PURA, IGIP, LOC101929719, CYSTM1, PFDN1, HBEGF, SLC4A9, ANKHD1, ANKHD1-EIF4EBP3, EIF4EBP3, SRA1, APBB3, MIR6831, SLC35A4, CD14, TMCO6, NDUFA2, IK, MIR3655, WDR55, DND1, HARS, HARS2, ZMAT2, VTRNA1-1, VTRNA1-2, VTRNA1-3, PCDHA1, PCDHA2, PCDHA3, PCDHA4, PCDHA5, PCDHA6, PCDHA7, PCDHA8, PCDHA9, PCDHA10, PCDHA11, PCDHA12, PCDHA13, PCDHAC1, PCDHAC2, LOC101926905, PCDHB1, PCDHB2, PCDHB3, PCDHB4, PCDHB5, PCDHB6, PCDHB17P, PCDHB7, PCDHB8, PCDHB16, PCDHB9, PCDHB10, PCDHB11, PCDHB12, PCDHB13, PCDHB14, PCDHB18P, PCDHB19P, PCDHB15, SLC25A2, TAF7, PCDHGA1, PCDHGA2, PCDHGA3, PCDHGB1, PCDHGA4, PCDHGB2, PCDHGA5, PCDHGB3, PCDHGA6, PCDHGA7, PCDHGB4, PCDHGA8, PCDHGB5, PCDHGA9, PCDHGB6, PCDHGA10, PCDHGB7, PCDHGA11, PCDHGB8P, PCDHGA12, PCDHGC3, PCDHGC4, PCDHGC5, DIAPH1, LOC100505658, HDAC3, RELL2, FCHSD1, ARAP3, PCDH1, LOC729080, KIAA0141, PCDH12, RNF14, GNPDA1, NDFIP1, SPRY4, SPRY4-IT1, LOC101926941, FGF1, LOC101926975, ARHGAP26, ARHGAP26-AS1, ARHGAP26-IT1, NR3C1, MIR5197, HMHB1, YIPF5, KCTD16, PRELID2, GRXCR2, SH3RF2, PLAC8L1, LARS, RBM27, POU4F3, TCERG1, GPR151, PPP2R2B, PPP2R2B-IT1, STK32A, DPYSL3, JAKMIP2-AS1, JAKMIP2, SPINK1, SCGB3A2, C5orf46, SPINK5, SPINK14, SPINK6, LOC102546294, SPINK13, SPINK7, SPINK9, FBXO38, HTR4, ADRB2, SH3TC2, LOC255187, ABLIM3, AFAP1L1, GRPEL2, GRPEL2-AS1, PCYOX1L, IL17B, CARMN, MIR143, MIR145, CSNK1A1, ARHGEF37, PPARGC1B, MIR378A, PDE6A, LOC644762, SLC26A2, TIGD6, HMGXB3, CSF1R, PDGFRB, CDX1, SLC6A7, CAMK2A, ARSI, TCOF1, CD74, RPS14, LOC102546298, NDST1, SYNPO, MYOZ3, RBM22, DCTN4, SMIM3, IRGM, ZNF300, ZNF300P1, GPX3, TNIP1, ANXA6, CCDC69, GM2A, SLC36A3, SLC36A2, SLC36A1, FAT2, MIR6499, SPARC, CTB-113P19.1, ATOX1, LOC100652758, G3BP1, GLRA1, CTB-12O2.1, NMUR2, LINC01470, GRIA1, FAM114A2, MFAP3, GALNT10, MIR1294, SAP30L-AS1, SAP30L, HAND1, MIR3141, MIR1303, LARP1, FAXDC2, MIR378H, CNOT8, GEMIN5, MRPL22, KIF4B, SGCD, PPP1R2P3, TIMD4, HAVCR1, HAVCR2, MED7, FAM71B, ITK, CYFIP2, FNDC9, LOC102724404, NIPAL4, ADAM19, SOX30, C5orf52, THG1L, LSM11, CLINT1, LOC101927697, EBF1, LOC101927740, RNF145, LOC105377682, UBLCP1, IL12B, LOC285626, LOC285627, LOC101927766, ADRA1B, TTC1, PWWP2A, FABP6, CCNJL, C1QTNF2, ZBED8, SLU7, PTTG1, MIR3142HG, MIR3142, MIR146A, ATP10B, LOC285629, GABRB2, GABRA6, GABRA1, LINC01202, GABRG2, CCNG1, NUDCD2, HMMR, HMMR-AS1, MAT2B, LOC101927835, LOC102546299, CTB-7E3.1, LOC101927908, TENM2, CTB-178M22.2, WWC1, RARS, FBLL1, PANK3, MIR103A1, MIR103B1, SLIT3, LOC101927969, MIR218-2, LOC728095, MIR585, SPDL1, DOCK2, FAM196B, MIR378E, FOXI1, LINC01187, C5orf58, LCP2, LINC01366, KCNIP1, KCNMB1, CTD-2270F17.1, LOC105377716, GABRP, RANBP17, TLX3, MIR3912, NPM1, FGF18, SMIM23, FBXW11, STK10, EFCAB9, UBTD2, LOC100288254, SH3PXD2B, NEURL1B, MIR5003, LOC101928093, DUSP1, ERGIC1, LOC100268168, RPL26L1, ATP6V0E1, SNORA74B, CREBRF, BNIP1, NKX2-5, STC2, MIR8056, LOC285593, BOD1, LINC01484, LINC01485, CPEB4, C5orf47, HMP19, LINC01411, MSX2, MIR4634, FLJ16171, DRD1, SFXN1, HRH2, CPLX2, THOC3, LOC100996385, FAM153B, LOC100507387, LOC643201, SIMC1, KIAA1191, ARL10, MIR1271, NOP16, HIGD2A, CLTB, FAF2, RNF44, CDHR2, GPRIN1, SNCB, MIR4281, EIF4E1B, TSPAN17, LINC01574, UNC5A, HK3, UIMC1, ZNF346, FGFR4, NSD1, RAB24, PRELID1, MXD3, LMAN2, RGS14, SLC34A1, PFN3, F12, GRK6, PRR7-AS1, PRR7, DBN1, PDLIM7, DOK3, DDX41, FAM193B, TMED9, B4GALT7, LOC202181, FAM153A, LOC728554, PROP1, FAM153C, N4BP3, RMND5B, NHP2, GMCL1P1, HNRNPAB, PHYKPL, COL23A1, CLK4, ZNF354A, AACSP1, ZNF354B, ZFP2, ZNF454, GRM6, ZNF879, ZNF354C, ADAMTS2, RUFY1, LOC101928445, HNRNPH1, C5orf60, LOC105377763, CBY3, CANX, MAML1, LTC4S, MGAT4B, MIR1229, SQSTM1, C5orf45, LOC100996419, TBC1D9B, RNF130, MIR340, RASGEF1C, MAPK9, GFPT2, CNOT6, SCGB3A1, FLT4, OR2Y1, MGAT1, HEIH, LINC00847, ZFP62, BTNL8, BTNL3, BTNL9, MIR8089, OR2V1, OR2V2, LOC102577426, TRIM7, MIR4638, TRIM41, RACK1, SNORD96A, SNORD95, CTC-338M12.4, TRIM52, TRIM52-AS1 | arr[GRCh37] 5q11.2q35.3(54601451_180698312)x1 |
| 1  CRC | Loss | 8 | p23.3 | p12 | 29,873 | RPL23AP53, ZNF596, FAM87A, FBXO25, TDRP, ERICH1, ERICH1-AS1, LOC401442, LOC286083, DLGAP2, DLGAP2-AS1, LOC101927752, CLN8, MIR3674, MIR596, ARHGEF10, LOC101928058, KBTBD11-OT1, KBTBD11, MYOM2, MIR7160, LOC101927815, CSMD1, LOC100287015, MCPH1, ANGPT2, MCPH1-AS1, MIR8055, AGPAT5, MIR4659A, MIR4659B, XKR5, GS1-24F4.2, DEFB1, DEFA6, DEFA4, DEFA8P, DEFA9P, DEFA10P, DEFA1, DEFA1B, DEFT1P2, DEFT1P, DEFA3, DEFA11P, DEFA5, LINC00965, FAM66B, DEFB109P1B, USP17L1, USP17L4, ZNF705G, DEFB4B, DEFB103B, DEFB103A, SPAG11B, DEFB104A, DEFB104B, DEFB106B, DEFB106A, DEFB105B, DEFB105A, DEFB107B, DEFB107A, PRR23D1, PRR23D2, FAM90A7P, FAM90A10P, SPAG11A, DEFB4A, ZNF705B, FAM66E, USP17L8, USP17L3, MIR548I3, FAM86B3P, SGK223, CLDN23, MFHAS1, ERI1, MIR4660, PPP1R3B, LOC101929128, LOC157273, TNKS, MIR597, LINC00599, MIR124-1, MSRA, LINCR-0001, PRSS55, RP1L1, MIR4286, C8orf74, SOX7, PINX1, MIR1322, LOC101929229, XKR6, MIR598, LOC101929269, MTMR9, SLC35G5, TDH, FAM167A-AS1, FAM167A, BLK, LINC00208, GATA4, SNORA99, C8orf49, NEIL2, FDFT1, CTSB, DEFB136, DEFB135, DEFB134, LOC100133267, DEFB130, ZNF705D, FAM66D, LOC392196, USP17L7, USP17L2, FAM90A2P, FAM86B1, FAM66A, LOC649352, DEFB109P1, FAM90A25P, FAM86B2, LOC100506990, LOC729732, MIR5692A1, MIR5692A2, LONRF1, MIR3926-1, MIR3926-2, LOC340357, LINC00681, KIAA1456, DLC1, C8orf48, LOC102725080, SGCZ, MIR383, TUSC3, MSR1, FGF20, MICU3, ZDHHC2, CNOT7, VPS37A, MTMR7, SLC7A2, PDGFRL, MTUS1, MIR548V, FGL1, PCM1, ASAH1, LOC101929066, NAT1, NAT2, PSD3, LOC100128993, SH2D4A, CSGALNACT1, INTS10, LPL, SLC18A1, ATP6V1B2, LZTS1, LZTS1-AS1, LOC102467222, LOC286114, LOC101929172, GFRA2, DOK2, XPO7, NPM2, FGF17, DMTN, FAM160B2, NUDT18, HR, REEP4, LGI3, SFTPC, BMP1, PHYHIP, MIR320A, POLR3D, LOC100507071, PIWIL2, SLC39A14, PPP3CC, SORBS3, PDLIM2, C8orf58, CCAR2, BIN3, BIN3-IT1, EGR3, PEBP4, LOC101929237, RHOBTB2, TNFRSF10B, LOC286059, LOC254896, TNFRSF10C, TNFRSF10D, TNFRSF10A, LOC389641, CHMP7, R3HCC1, LOXL2, LOC100507156, ENTPD4, SLC25A37, NKX3-1, NKX2-6, STC1, ADAM28, LOC101929294, ADAMDEC1, ADAM7, LOC101929315, NEFM, NEFL, MIR6841, DOCK5, MIR6876, GNRH1, KCTD9, CDCA2, EBF2, PPP2R2A, BNIP3L, PNMA2, DPYSL2, ADRA1A, STMN4, TRIM35, PTK2B, MIR6842, CHRNA2, EPHX2, CLU, MIR6843, SCARA3, MIR3622B, MIR3622A, CCDC25, ESCO2, PBK, SCARA5, MIR4287, NUGGC, ELP3, PNOC, ZNF395, FBXO16, FZD3, MIR4288, MIR7641-2, EXTL3-AS1, EXTL3, INTS9, HMBOX1, KIF13B, DUSP4, LINC00589, LOC101929450, LOC101929470, FAM183CP, MIR3148, SARAF, LEPROTL1, MBOAT4, DCTN6 | arr[GRCh37] 8p23.3p12(172416_30045554)x1 |
| 1  CRC | Loss | 9 | p24.3 | q34.3 | 140,850 | C9orf66, DOCK8, KANK1, DMRT1, DMRT3, LINC01230, DMRT2, SMARCA2, VLDLR-AS1, VLDLR, KCNV2, PUM3, LINC01231, RFX3, RFX3-AS1, GLIS3, GLIS3-AS1, SLC1A1, SPATA6L, PLPP6, CDC37L1-AS1, CDC37L1, AK3, RCL1, MIR101-2, JAK2, INSL6, INSL4, RLN2, RLN1, PLGRKT, CD274, PDCD1LG2, RIC1, ERMP1, MLANA, KIAA2026, MIR4665, RANBP6, IL33, TPD52L3, UHRF2, GLDC, KDM4C, TMEM261, PTPRD, PTPRD-AS1, LOC105375972, PTPRD-AS2, TYRP1, LURAP1L-AS1, LURAP1L, SNORD137, MPDZ, FLJ41200, LINC00583, NFIB, ZDHHC21, CER1, FREM1, LOC389705, TTC39B, SNAPC3, PSIP1, CCDC171, C9orf92, BNC2, CNTLN, SH3GL2, ADAMTSL1, MIR3152, SAXO1, RRAGA, HAUS6, SCARNA8, PLIN2, DENND4C, RPS6, ACER2, SLC24A2, MLLT3, MIR4473, MIR4474, FOCAD, FOCAD-AS1, MIR491, HACD4, IFNB1, IFNW1, IFNA21, IFNA4, IFNA7, IFNA10, IFNA16, IFNA17, IFNA14, IFNA22P, IFNA5, KLHL9, IFNA6, IFNA13, IFNA2, IFNA8, IFNA1, MIR31HG, IFNE, MIR31, MTAP, CDKN2A-AS1, CDKN2A, CDKN2B-AS1, CDKN2B, DMRTA1, LINC01239, LOC101929563, ELAVL2, IZUMO3, TUSC1, LINC01241, LOC100506422, CAAP1, PLAA, IFT74, IFT74-AS1, LRRC19, TEK, LINC00032, EQTN, MOB3B, IFNK, C9orf72, LINGO2, MIR876, MIR873, LINC01242, LINC01243, ACO1, DDX58, TOPORS, TOPORS-AS1, NDUFB6, TAF1L, TMEM215, APTX, DNAJA1, SMU1, B4GALT1, B4GALT1-AS1, SPINK4, BAG1, CHMP5, NFX1, AQP7, AQP3, NOL6, MIR6851, SUGT1P1, ANKRD18B, ANXA2P2, PTENP1, PTENP1-AS, LINC01251, PRSS3, UBE2R2, UBAP2, SNORD121B, SNORD121A, DCAF12, UBAP1, KIF24, NUDT2, KIAA1161, C9orf24, FAM219A, DNAI1, ENHO, CNTFR, CNTFR-AS1, RPP25L, DCTN3, ARID3C, SIGMAR1, GALT, IL11RA, CCL27, LOC730098, CCL19, CCL21, FAM205A, FAM205BP, FAM205C, PHF24, DNAJB5-AS1, DNAJB5, C9orf131, VCP, FANCG, PIGO, STOML2, FAM214B, UNC13B, ATP8B5P, RUSC2, FAM166B, TESK1, MIR4667, CD72, LOC101926948, SIT1, RMRP, CCDC107, ARHGEF39, CA9, TPM2, TLN1, MIR6852, CREB3, MIR6853, GBA2, RGP1, MSMP, NPR2, SPAG8, HINT2, FAM221B, TMEM8B, LINC00950, OR13J1, HRCT1, LINC00961, OR2S2, RECK, GLIPR2, CCIN, CLTA, GNE, RNF38, MELK, MIR4475, PAX5, MIR4540, MIR4476, EBLN3, ZCCHC7, GRHPR, ZBTB5, POLR1E, FBXO10, TOMM5, FRMPD1, TRMT10B, EXOSC3, DCAF10, SLC25A51, SHB, ALDH1B1, IGFBPL1, FAM95C, ANKRD18A, FAM201A, CNTNAP3, SPATA31A1, FAM74A1, ZNF658B, LOC105379450, SPATA31A3, FAM74A3, ZNF658, SPATA31A5, SPATA31A7, FAM74A6, GLIDR, KGFLP2, LOC102724238, LOC554249, ANKRD20A2, ANKRD20A3, FAM95B1, LOC105379252, GXYLT1P3, FOXD4L4, LOC101928381, LOC101929583, LOC101928195, LOC286297, AQP7P3, LOC642929, FAM74A7, SPATA31A6, CNTNAP3B, CNTNAP3P2, XLOC_007697, LOC101927827, LOC103908605, FAM27C, LOC102723709, FAM27E2, LOC105376064, KGFLP1, LINC01189, FAM74A4, LINC01410, LOC100132249, PTGER4P2-CDK2AP2P2, LOC403323, LOC728673, AQP7P1, LOC102724580, FAM27E3, FAM27B, ANKRD20A1, MIR4477B, MIR4477A, FRG1JP, LOC102725126, FRG1HP, MIR1299, PGM5P2, LOC440896, FOXD4L6, CBWD6, CBWD5, ANKRD20A4, LOC100133920, FOXD4L5, CBWD3, FOXD4L3, PGM5-AS1, PGM5, TMEM252, LINC01506, PIP5K1B, FAM122A, LOC101927069, PRKACG, FXN, TJP2, BANCR, FAM189A2, APBA1, PTAR1, C9orf135-AS1, C9orf135, MAMDC2, MAMDC2-AS1, SMC5-AS1, SMC5, KLF9, TRPM3, MIR204, TMEM2, ABHD17B, C9orf85, C9orf57, GDA, LINC01504, ZFAND5, TMC1, LINC01474, ALDH1A1, ANXA1, LOC101927358, RORB-AS1, RORB, TRPM6, C9orf40, C9orf41-AS1, CARNMT1, NMRK1, OSTF1, MIR548H3, PCSK5, RFK, RPSAP9, GCNT1, PRUNE2, PCA3, FOXB2, VPS13A-AS1, VPS13A, GNA14, GNA14-AS1, GNAQ, CEP78, PSAT1, LOC101927450, TLE4, LINC01507, TLE1, LOC101927502, SPATA31D5P, SPATA31D4, SPATA31D3, SPATA31D1, RASEF, FRMD3, IDNK, UBQLN1, LOC105376114, GKAP1, KIF27, C9orf64, HNRNPK, MIR7-1, RMI1, LOC101927575, SLC28A3, NTRK2, AGTPBP1, LOC389765, NAA35, GOLM1, LOC101927623, C9orf153, ISCA1, ZCCHC6, GAS1, GAS1RR, LOC440173, LOC494127, C9orf170, DAPK1, CTSL, CTSL3P, CTSLP8, LOC392364, SPATA31E1, SPATA31C1, CDK20, LOC102724156, SPATA31C2, SPIN1, NXNL2, LOC286238, MIR4289, C9orf47, S1PR3, SHC3, CKS2, MIR3153, SECISBP2, SEMA4D, GADD45G, UNQ6494, LOC101927847, MIR4290HG, MIR4290, LINC01508, LINC01501, DIRAS2, SYK, LOC100129316, LINC00484, AUH, NFIL3, MIR3910-1, MIR3910-2, ROR2, SPTLC1, LOC100128076, LINC00475, IARS, MIR3651, SNORA84, NOL8, CENPP, OGN, OMD, ASPN, ECM2, MIR4670, IPPK, LOC100128361, BICD2, LOC101929748, ANKRD19P, ZNF484, LOC642943, FGD3, LOC101927954, SUSD3, CARD19, NINJ1, WNK2, C9orf129, FAM120AOS, FAM120A, PHF2, MIR548AU, MIR4291, BARX1, PTPDC1, MIRLET7A1, MIRLET7F1, MIRLET7DHG, MIRLET7D, ZNF169, NUTM2F, LOC100132077, MFSD14B, PCAT7, FBP2, FBP1, C9orf3, MIR2278, MIR6081, MIR23B, MIR27B, MIR3074, MIR24-1, FANCC, PTCH1, LOC100507346, LINC00476, ERCC6L2, LINC00092, LOC158435, LOC158434, HSD17B3, SLC35D2, ZNF367, HABP4, CDC14B, AAED1, LOC441455, ZNF510, ZNF782, LOC100132781, LOC441454, NUTM2G, MFSD14C, CTSV, GAS2L1P2, ANKRD18CP, LOC100499484, LOC100499484-C9ORF174, CCDC180, MIR1302-8, LOC286359, TDRD7, TMOD1, TSTD2, NCBP1, XPA, FOXE1, TRMO, HEMGN, ANP32B, NANS, TRIM14, CORO2A, TBC1D2, MIR6854, GABBR2, ANKS6, GALNT12, COL15A1, TGFBR1, ALG2, SEC61B, NAMA, LOC101928438, NR4A3, STX17-AS1, STX17, ERP44, INVS, TEX10, MSANTD3, MSANTD3-TMEFF1, TMEFF1, MURC, PLPPR1, BAAT, MRPL50, ZNF189, ALDOB, TMEM246-AS1, TMEM246, RNF20, GRIN3A, PPP3R2, LINC00587, CYLC2, LINC01492, LOC101928523, SMC2-AS1, SMC2, LOC105376194, OR13F1, OR13C4, OR13C3, OR13C8, OR13C5, OR13C2, OR13C9, OR13D1, NIPSNAP3A, NIPSNAP3B, ABCA1, SLC44A1, FSD1L, FKTN, TAL2, TMEM38B, MIR8081, LINC01505, ZNF462, LOC340512, RAD23B, LINC01509, KLF4, ACTL7B, ACTL7A, IKBKAP, FAM206A, CTNNAL1, TMEM245, MIR32, FRRS1L, EPB41L4B, PTPN3, MIR3927, PALM2, PALM2-AKAP2, AKAP2, C9orf152, TXN, TXNDC8, SVEP1, MUSK, LPAR1, MIR7702, OR2K2, KIAA0368, ZNF483, PTGR1, LRRC37A5P, DNAJC25, DNAJC25-GNG10, GNG10, C9orf84, UGCG, MIR4668, SUSD1, PTBP3, HSDL2, KIAA1958, INIP, SNX30, SLC46A2, ZNF883, ZFP37, FAM225B, FAM225A, SLC31A2, FKBP15, SLC31A1, CDC26, PRPF4, RNF183, WDR31, BSPRY, HDHD3, ALAD, POLE3, C9orf43, RGS3, ZNF618, AMBP, KIF12, COL27A1, MIR455, ORM1, ORM2, AKNA, WHRN, ATP6V1G1, TMEM268, LOC100505478, TNFSF15, TNFSF8, TNC, LOC101928748, DEC1, LOC101928775, LINC00474, PAPPA, PAPPA-AS1, ASTN2, ASTN2-AS1, TRIM32, SNORA70C, LOC101928797, TLR4, BRINP1, LINC01613, MIR147A, CDK5RAP2, MEGF9, FBXW2, LOC100288842, PSMD5, PSMD5-AS1, PHF19, TRAF1, C5, CNTRL, RAB14, GSN, GSN-AS1, STOM, GGTA1P, DAB2IP, TTLL11, MIR4478, NDUFA8, MORN5, LHX6, RBM18, MRRF, PTGS1, OR1J1, OR1J2, OR1J4, OR1N1, OR1N2, OR1L8, OR1Q1, OR1B1, OR1L1, OR1L3, OR1L4, OR1L6, OR5C1, OR1K1, PDCL, RC3H2, SNORD90, ZBTB6, ZBTB26, RABGAP1, GPR21, MIR600HG, MIR600, STRBP, CRB2, DENND1A, MIR601, MIR7150, LOC100505588, LHX2, NEK6, PSMB7, LOC100129034, NR5A1, NR6A1, MIR181A2HG, MIR181A2, MIR181B2, OLFML2A, WDR38, RPL35, ARPC5L, GOLGA1, SCAI, PPP6C, LOC105376271, RABEPK, HSPA5, GAPVD1, MAPKAP1, LOC51145, PBX3, LOC101929116, MVB12B, NRON, LMX1B, ZBTB43, ZBTB34, RALGPS1, ANGPTL2, GARNL3, SLC2A8, ZNF79, RPL12, SNORA65, LRSAM1, FAM129B, STXBP1, MIR3911, CFAP157, PTRH1, TTC16, TOR2A, SH2D3C, MIR3960, MIR2861, CDK9, FPGS, ENG, LOC102723566, AK1, MIR4672, ST6GALNAC6, ST6GALNAC4, PIP5KL1, DPM2, FAM102A, NAIF1, SLC25A25, SLC25A25-AS1, PTGES2, PTGES2-AS1, LCN2, C9orf16, CIZ1, DNM1, MIR199B, MIR3154, GOLGA2, SWI5, TRUB2, COQ4, SLC27A4, MIR1268A, URM1, MIR219A2, MIR219B, CERCAM, ODF2, GLE1, SPTAN1, WDR34, SET, PKN3, ZDHHC12, LOC100506100, ZER1, TBC1D13, ENDOG, C9orf114, KYAT1, LRRC8A, PHYHD1, DOLK, NUP188, SH3GLB2, MIGA2, DOLPP1, CRAT, PTPA, IER5L, LOC101929331, C9orf106, LINC01503, LINC00963, NTMT1, C9orf50, ASB6, PRRX2, PRRX2-AS1, PTGES, TOR1B, TOR1A, C9orf78, USP20, MIR6855, FNBP1, GPR107, LOC401554, NCS1, HMCN2, ASS1, LOC100272217, FUBP3, MIR6856, PRDM12, EXOSC2, ABL1, QRFP, FIBCD1, LAMC3, AIF1L, NUP214, FAM78A, PLPP7, PRRC2B, SNORD62B, SNORD62A, POMT1, UCK1, RAPGEF1, MED27, NTNG2, SETX, TTF1, CFAP77, BARHL1, DDX31, GTF3C4, AK8, SPACA9, TSC1, GFI1B, MIR548AW, SNORD141B, SNORD141A, LOC105376306, GTF3C5, MIR6877, CEL, CELP, RALGDS, GBGT1, OBP2B, ABO, SURF6, MED22, RPL7A, SNORD24, SNORD36B, SNORD36A, SNORD36C, SURF1, SURF2, SURF4, STKLD1, REXO4, ADAMTS13, CACFD1, SLC2A6, TMEM8C, ADAMTSL2, FAM163B, DBH, DBH-AS1, SARDH, VAV2, LINC00094, BRD3, LOC100130548, WDR5, RNU6ATAC, RXRA, MIR4669, COL5A1, LOC101448202, MIR3689A, MIR3689C, MIR3689D1, MIR3689B, MIR3689D2, MIR3689E, MIR3689F, FCN2, FCN1, OLFM1, LOC401557, C9orf62, PPP1R26-AS1, PPP1R26, C9orf116, MRPS2, LOC101928525, LCN1, OBP2A, PAEP, LINC01502, GLT6D1, LCN9, SOHLH1, KCNT1, CAMSAP1, UBAC1, NACC2, C9orf69, LHX3, QSOX2, DKFZP434A062, GPSM1, DNLZ, CARD9, SNAPC4, SDCCAG3, PMPCA, INPP5E, SEC16A, C9orf163, NOTCH1, MIR4673, MIR4674, NALT1, LINC01451, HSPC324, EGFL7, MIR126, AGPAT2, FAM69B, SNHG7, SNORA17B, SNORA17A, LCN10, LCN6, LOC100128593, MIR6722, LCN8, LCN15, TMEM141, CCDC183, CCDC183-AS1, RABL6, MIR4292, C9orf172, PHPT1, MAMDC4, EDF1, TRAF2, MIR4479, FBXW5, C8G, LCN12, PTGDS, LCNL1, C9orf142, CLIC3, ABCA2, C9orf139, FUT7, NPDC1, ENTPD2, SAPCD2, UAP1L1, MAN1B1-AS1, MAN1B1, DPP7, GRIN1, LRRC26, MIR3621, TMEM210, ANAPC2, SSNA1, TPRN, TMEM203, NDOR1, RNF208, CYSRT1, RNF224, SLC34A3, TUBB4B, FAM166A, C9orf173-AS1, STPG3, NELFB, TOR4A, NRARP, EXD3, NOXA1, ENTPD8, NSMF, MIR7114, PNPLA7, MRPL41, DPH7, ZMYND19, ARRDC1, ARRDC1-AS1, EHMT1, EHMT1-IT1, MIR602, LOC100133077, CACNA1B, LOC105376331, LOC101928786, TUBBP5 | arr[GRCh37] 9p24.3q34.3(204737_141054761)x1 |
| 1  CRC | Loss | 14 | q21.2 | q24.2 | 25,288 | LINC00871, RPL10L, MDGA2, MIR548Y, LINC00648, RPS29, LRR1, RPL36AL, MGAT2, DNAAF2, POLE2, KLHDC1, KLHDC2, NEMF, ARF6, MIR6076, LINC01588, LINC01599, VCPKMT, SOS2, L2HGDH, MIR4504, ATP5S, CDKL1, MAP4K5, ATL1, SAV1, NIN, LOC105370489, ABHD12B, PYGL, TRIM9, TMX1, LINC00640, FRMD6-AS2, FRMD6, FRMD6-AS1, GNG2, LOC102723604, C14orf166, NID2, PTGDR, PTGER2, TXNDC16, GPR137C, ERO1A, PSMC6, STYX, GNPNAT1, FERMT2, DDHD1, LOC101927620, MIR5580, BMP4, CDKN3, CNIH1, GMFB, CGRRF1, SAMD4A, GCH1, MIR4308, WDHD1, SOCS4, MAPK1IP1L, LGALS3, DLGAP5, FBXO34, ATG14, TBPL2, KTN1-AS1, KTN1, RPL13AP3, LINC00520, PELI2, LOC101927690, TMEM260, OTX2, OTX2-AS1, EXOC5, AP5M1, NAA30, C14orf105, SLC35F4, C14orf37, ACTR10, PSMA3, PSMA3-AS1, ARID4A, TOMM20L, TIMM9, KIAA0586, DACT1, LINC01500, DAAM1, GPR135, L3HYPDH, JKAMP, CCDC175, RTN1, MIR5586, LRRC9, PCNX4, DHRS7, PPM1A, C14orf39, SIX6, SALRNA1, SIX1, SIX4, MNAT1, TRMT5, SLC38A6, TMEM30B, PRKCH, LOC101927780, FLJ22447, HIF1A-AS1, HIF1A, HIF1A-AS2, SNAPC1, SYT16, LINC00643, LINC00644, KCNH5, RHOJ, GPHB5, PPP2R5E, WDR89, SGPP1, SYNE2, MIR548H1, ESR2, TEX21P, MTHFD1, ZBTB25, AKAP5, ZBTB1, LOC102723809, HSPA2, PPP1R36, PLEKHG3, SPTB, MIR7855, CHURC1, CHURC1-FNTB, GPX2, RAB15, FNTB, MAX, MIR4706, LOC100506321, LOC100128233, MIR4708, FUT8, FUT8-AS1, MIR625, LINC00238, GPHN, FAM71D, MPP5, ATP6V1D, EIF2S1, PLEK2, MIR5694, TMEM229B, PLEKHH1, PIGH, ARG2, VTI1B, RDH11, RDH12, ZFYVE26, RAD51B, LOC100996664, ZFP36L1, ACTN1, ACTN1-AS1, DCAF5, EXD2, GALNT16, ERH, SLC39A9, PLEKHD1, CCDC177, SUSD6, LOC100289511, SRSF5, SLC10A1, SMOC1, SLC8A3, LOC646548, ADAM21P1, COX16, SYNJ2BP-COX16, SYNJ2BP, ADAM21, ADAM20P1, ADAM20, MED6, LOC101928075, TTC9, LINC01269, MAP3K9 | arr[GRCh37] 14q21.2q24.2(46079882_71367861)x1-2 |
| 1  CRC | Loss | 17 | p13.3 | p11.2 | 20,721 | VPS53, FAM57A, GEMIN4, DBIL5P, GLOD4, MRM3, NXN, LOC101927727, TIMM22, ABR, MIR3183, BHLHA9, TUSC5, YWHAE, CRK, MYO1C, INPP5K, PITPNA-AS1, PITPNA, SLC43A2, SCARF1, RILP, PRPF8, TLCD2, MIR22HG, MIR22, WDR81, SERPINF2, SERPINF1, SMYD4, RPA1, RTN4RL1, LOC105371485, DPH1, OVCA2, MIR132, MIR212, HIC1, SMG6, LOC101927839, SRR, TSR1, SNORD91B, SNORD91A, SGSM2, MNT, LOC284009, METTL16, PAFAH1B1, CLUH, MIR6776, LOC105371592, MIR1253, RAP1GAP2, LOC101927911, OR1D5, OR1D2, OR1G1, OR1A2, OR1A1, OR1D4, OR3A2, OR3A1, OR3A4P, OR1E1, OR3A3, OR1E2, SPATA22, ASPA, TRPV3, TRPV1, SHPK, CTNS, TAX1BP3, P2RX5-TAX1BP3, EMC6, P2RX5, ITGAE, GSG2, NCBP3, CAMKK1, P2RX1, ATP2A3, ZZEF1, CYB5D2, ANKFY1, UBE2G1, LOC103021295, SPNS3, SPNS2, MYBBP1A, GGT6, SMTNL2, ALOX15, PELP1, LOC101559451, ARRB2, MED11, CXCL16, ZMYND15, TM4SF5, VMO1, GLTPD2, PSMB6, PLD2, MINK1, CHRNE, C17orf107, GP1BA, SLC25A11, RNF167, PFN1, ENO3, SPAG7, CAMTA2, MIR6864, MIR6865, INCA1, KIF1C, LOC102724009, SLC52A1, ZFP3, ZNF232, LOC101928000, USP6, ZNF594, LOC100130950, SCIMP, RABEP1, NUP88, RPAIN, C1QBP, DHX33, LOC105371506, DERL2, MIS12, LOC728392, NLRP1, LOC339166, WSCD1, AIPL1, FAM64A, PITPNM3, KIAA0753, TXNDC17, MED31, C17orf100, MIR4520-1, MIR4520-2, ALOX15P1, SLC13A5, XAF1, FBXO39, TEKT1, ALOX12P2, ALOX12-AS1, ALOX12, RNASEK, RNASEK-C17orf49, C17orf49, MIR497HG, MIR195, MIR497, BCL6B, SLC16A13, SLC16A11, CLEC10A, ASGR2, ASGR1, DLG4, ACADVL, MIR324, DVL2, PHF23, GABARAP, CTDNEP1, ELP5, CLDN7, SLC2A4, YBX2, EIF5A, GPS2, NEURL4, ACAP1, KCTD11, TMEM95, TNK1, PLSCR3, TMEM256-PLSCR3, TMEM256, NLGN2, SPEM1, C17orf74, TMEM102, FGF11, CHRNB1, ZBTB4, SLC35G6, POLR2A, TNFSF12, TNFSF12-TNFSF13, TNFSF13, SENP3, SENP3-EIF4A1, EIF4A1, SNORA48, SNORD10, SNORA67, CD68, LOC100996842, MPDU1, SOX15, FXR2, SHBG, SAT2, ATP1B2, TP53, WRAP53, EFNB3, DNAH2, RPL29P2, KDM6B, TMEM88, NAA38, CYB5D1, CHD3, SCARNA21, LOC284023, KCNAB3, TRAPPC1, CNTROB, GUCY2D, ALOX15B, ALOX12B, MIR4314, ALOXE3, HES7, PER1, MIR6883, VAMP2, TMEM107, MIR4521, BORCS6, AURKB, LINC00324, CTC1, PFAS, SLC25A35, RANGRF, ARHGEF15, ODF4, LOC100128288, KRBA2, RPL26, RNF222, NDEL1, MYH10, CCDC42, SPDYE4, MFSD6L, PIK3R6, PIK3R5, NTN1, LOC101928266, STX8, CFAP52, USP43, DHRS7C, GSG1L2, GLP2R, RCVRN, GAS7, MYH13, MYHAS, MYH8, MYH4, MYH1, MYH2, MYH3, SCO1, ADPRM, TMEM220, MAGOH2P, TMEM220-AS1, LINC00675, PIRT, SHISA6, DNAH9, ZNF18, MAP2K4, MIR744, LINC00670, MYOCD, LOC101928418, LOC100128006, ARHGAP44, MIR1269B, ELAC2, HS3ST3A1, CDRT15P1, COX10-AS1, COX10, CDRT15, HS3ST3B1, MGC12916, LOC101928475, CDRT7, CDRT8, PMP22, MIR4731, TEKT3, CDRT4, TVP23C-CDRT4, TVP23C, CDRT1, TRIM16, ZNF286A, TBC1D26, CDRT15P2, MEIS3P1, LOC101928567, ADORA2B, ZSWIM7, TTC19, NCOR1, PIGL, MIR1288, CENPV, UBB, TRPV2, LRRC75A-AS1, SNORD49B, SNORD49A, SNORD65, LRRC75A, ZNF287, ZNF624, CCDC144A, USP32P1, FAM106CP, KRT16P2, TNFRSF13B, MPRIP, PLD6, FLCN, COPS3, NT5M, MED9, RASD1, PEMT, SMCR2, RAI1, RAI1-AS1, SMCR5, SREBF1, MIR6777, MIR33B, TOM1L2, DRC3, ATPAF2, GID4, DRG2, MYO15A, ALKBH5, LLGL1, FLII, MIEF2, TOP3A, SMCR8, SHMT1, MIR6778, EVPLL, FLJ35934, KRT17P5, KRT16P1, LGALS9C, USP32P2, FAM106A, CCDC144B, TBC1D28, ZNF286B, FOXO3B, TRIM16L, FBXW10, TVP23B, PRPSAP2, SLC5A10, FAM83G, GRAP, LOC79999, LOC388436, GRAPL, EPN2, EPN2-IT1, EPN2-AS1, B9D1, MIR1180, MAPK7, MFAP4, RNF112, SLC47A1, SNORA59B, SNORA59A, ALDH3A2, SLC47A2, ALDH3A1, ULK2, AKAP10, SPECC1, CCDC144CP, FAM106B, LGALS9B, KRT16P3, CDRT15L2, LOC100287072, CCDC144NL, CCDC144NL-AS1, LOC339260, USP22, LINC01563, DHRS7B, TMEM11 | arr[GRCh37] 17p13.3p11.2(400958_21122322)x1 |
| 1  CRC | Loss | 17 | q24.3 | q25.2 | 6,072 | CASC17, LOC102723505, LINC01152, LOC102723517, SOX9-AS1, LOC101928205, SOX9, LOC146795, LINC00673, LINC00511, SLC39A11, SSTR2, COG1, FAM104A, C17orf80, CPSF4L, CDC42EP4, SDK2, LOC101928251, LOC100134391, LINC00469, LOC400620, RPL38, MGC16275, TTYH2, DNAI2, KIF19, BTBD17, GPR142, GPRC5C, CD300A, CD300LB, CD300C, LOC100130520, CD300LD, C17orf77, CD300E, RAB37, CD300LF, SLC9A3R1, MIR3615, NAT9, TMEM104, GRIN2C, FDXR, FADS6, USH1G, OTOP2, OTOP3, HID1, HID1-AS1, CDR2L, MRPL58, KCTD2, ATP5H, SLC16A5, ARMC7, NT5C, HN1, SUMO2, NUP85, GGA3, MRPS7, MIF4GD, LOC100287042, SLC25A19, GRB2, MIR3678, TMEM94, MIR6785, CASKIN2, TSEN54, LLGL2, MYO15B, RECQL5, SMIM5, SMIM6, SAP30BP, ITGB4, GALK1, H3F3B, MIR4738, UNK, UNC13D, WBP2, TRIM47, TRIM65, MRPL38, FBF1, ACOX1, TEN1, TEN1-CDK3, CDK3, MIR4538, EVPL, SRP68, GALR2, ZACN, EXOC7, MIR6868, FOXJ1, RNF157-AS1, RNF157, UBALD2, QRICH2, PRPSAP1, SPHK1, UBE2O, AANAT, RHBDF2, CYGB, PRCD, SNHG16, SNORD1C, SNORD1B, SNORD1A, ST6GALNAC2, ST6GALNAC1, LOC105274304, MXRA7, JMJD6, METTL23, SRSF2, MIR636, MFSD11, LOC101928514, LINC00868, MGAT5B, LOC105371899 | arr[GRCh37] 17q24.3q25.2(68913463_74985472)x1-2 |
| 1  CRC | Loss | 18 | p11.32 | q23 | 77,995 | LOC102723376, ROCK1P1, MIR8078, USP14, THOC1, COLEC12, LOC105376854, CETN1, CLUL1, TYMSOS, TYMS, ENOSF1, YES1, ADCYAP1, LINC00470, METTL4, NDC80, CBX3P2, SMCHD1, EMILIN2, LPIN2, LOC727896, MYOM1, MYL12A, LOC104968399, MYL12B, TGIF1, GAPLINC, DLGAP1, DLGAP1-AS1, DLGAP1-AS2, DLGAP1-AS3, MIR6718, DLGAP1-AS4, DLGAP1-AS5, AKAIN1, LINC00526, LINC00667, ZBTB14, EPB41L3, MIR3976HG, MIR3976, TMEM200C, L3MBTL4, L3MBTL4-AS1, MIR4317, LINC01387, LOC101927168, ARHGAP28, LINC00668, LAMA1, LOC101927188, LRRC30, PTPRM, LOC100192426, RAB12, GACAT2, MTCL1, NDUFV2, NDUFV2-AS1, ANKRD12, TWSG1, RALBP1, PPP4R1, PPP4R1-AS1, RAB31, TXNDC2, VAPA, LINC01254, APCDD1, NAPG, LOC101927410, PIEZO2, MIR6788, LINC01255, SLC35G4, MIR7153, GNAL, CHMP1B, MPPE1, IMPA2, ANKRD62, C18orf61, CIDEA, TUBB6, AFG3L2, PRELID3A, LOC105371998, SPIRE1, PSMG2, CEP76, LOC100996324, PTPN2, SEH1L, CEP192, LDLRAD4, LDLRAD4-AS1, MIR5190, MIR4526, FAM210A, RNMT, MC5R, MC2R, ZNF519, ANKRD20A5P, CYP4F35P, CXADRP3, POTEC, ANKRD30B, MIR3156-2, LINC01443, LINC01444, LOC644669, ROCK1, GREB1L, ESCO1, SNRPD1, ABHD3, MIR320C1, MIB1, MIR133A1HG, MIR133A1, MIR1-2, GATA6-AS1, GATA6, CTAGE1, LOC101927571, RBBP8, MIR4741, CABLES1, TMEM241, RIOK3, C18orf8, NPC1, ANKRD29, LAMA3, TTC39C, TTC39C-AS1, CABYR, OSBPL1A, MIR320C2, IMPACT, HRH4, LOC729950, LOC105372028, ZNF521, SS18, PSMA8, TAF4B, LINC01543, KCTD1, MIR8057, PCAT18, AQP4, AQP4-AS1, CHST9, LOC105372038, CDH2, MIR302F, DSC3, DSC2, DSCAS, DSC1, DSG1, DSG1-AS1, DSG4, DSG3, DSG2, DSG2-AS1, TTR, B4GALT6, SLC25A52, TRAPPC8, RNF125, RNF138, MEP1B, GAREM1, WBP11P1, KLHL14, CCDC178, ASXL3, NOL4, DTNA, MAPRE2, ZNF397, ZSCAN30, ZNF271P, ZNF24, ZNF396, INO80C, MIR3975, GALNT1, MIR187, MIR3929, C18orf21, RPRD1A, SLC39A6, ELP2, LOC101927809, MOCOS, FHOD3, LOC105372071, TPGS2, KIAA1328, LOC105372069, CELF4, LOC105372068, SNORA111, MIR4318, MIR924HG, MIR924, MIR5583-2, MIR5583-1, LINC01477, KC6, PIK3C3, LINC00907, RIT2, SYT4, LINC01478, LOC105667213, SETBP1, MIR4319, SLC14A2, SLC14A2-AS1, SLC14A1, SIGLEC15, EPG5, PSTPIP2, ATP5A1, HAUS1, C18orf25, RNF165, LOXHD1, ST8SIA5, PIAS2, KATNAL2, TCEB3CL, TCEB3CL2, TCEB3C, TCEB3B, HDHD2, IER3IP1, SKOR2, MIR4527, SMAD2, ZBTB7C, CTIF, MIR4743, SMAD7, DYM, MIR4744, C18orf32, RPL17-C18orf32, MIR1539, RPL17, SNORD58C, SNORD58A, SNORD58B, LIPG, ACAA2, SCARNA17, SNHG22, MYO5B, MIR4320, CFAP53, MBD1, CXXC1, SKA1, MAPK4, MRO, ME2, ELAC1, SMAD4, MEX3C, LINC01630, DCC, MIR4528, LOC102724651, LOC101928167, MBD2, SNORA37, POLI, STARD6, C18orf54, DYNAP, RAB27B, CCDC68, LOC101927229, TCF4, TCF4-AS1, MIR4529, LINC01416, LINC01539, TXNL1, WDR7, LINC-ROR, BOD1L2, ST8SIA3, ONECUT2, FECH, NARS, LOC100505549, ATP8B1, NEDD4L, MIR122, MIR3591, ALPK2, SNORA108, LOC101927322, MALT1, ZNF532, OACYLP, SEC11C, GRP, RAX, CPLX4, LMAN1, CCBE1, PMAIP1, MC4R, CDH20, LINC01544, RNF152, PIGN, KIAA1468, TNFRSF11A, ZCCHC2, PHLPP1, BCL2, KDSR, VPS4B, SERPINB5, SERPINB12, SERPINB13, SERPINB4, SERPINB3, SERPINB11, SERPINB7, SERPINB2, SERPINB10, HMSD, SERPINB8, LINC00305, LOC284294, LINC01538, CDH7, CDH19, MIR5011, DSEL, LOC643542, TMX3, CCDC102B, DOK6, LOC105372179, CD226, RTTN, SOCS6, LOC101927481, LOC101060542, GTSCR1, LINC01541, LOC102724913, CBLN2, NETO1, MIR548AV, LOC100505797, LOC400655, LOC100505817, FBXO15, TIMM21, CYB5A, C18orf63, LOC101927606, FAM69C, CNDP2, CNDP1, LINC00909, ZNF407, ZADH2, TSHZ1, SMIM21, LOC100505853, LOC339298, ZNF516, LOC101927989, C18orf65, LINC00908, LINC00683, LOC101927651, LOC400661, LOC100131655, ZNF236, MBP, GALR1, LINC01029, SALL3, ATP9B, NFATC1, LOC284241, CTDP1, KCNG2, PQLC1, HSBP1L1, TXNL4A, RBFA, RBFADN, ADNP2, PARD6G-AS1, PARD6G | arr[GRCh37] 18p11.32q23(12841_78007784)x1 |
| 1  CRC | Loss | 21 | q11.2 | q21.1 | 9,271 | ANKRD30BP2, MIR3156-3, LOC102724188, POTED, MIR3118-1, MIR8069-1, MIR8069-2, CYP4F29P, ANKRD20A11P, LIPI, RBM11, ABCC13, HSPA13, SAMSN1, SAMSN1-AS1, LOC388813, NRIP1, USP25, MIR99AHG, MIR99A, MIRLET7C, MIR125B2, LINC01549, CXADR, BTG3, C21orf91-OT1, C21orf91, CHODL-AS1, CHODL, TMPRSS15, MIR548XHG, MIR548X, LINC00320, NCAM2, LINC00317, LINC01425, LOC101927843, LINC00308 | arr[GRCh37] 21q11.2q21.1(14344536_23615318)x1 |
| 1  LM | Gain | 5 | p15.33 | q11.2 | 54,289 | PLEKHG4B, LRRC14B, CCDC127, SDHA, HRAT5, PDCD6, AHRR, EXOC3-AS1, EXOC3, PP7080, SLC9A3, LOC100288152, MIR4456, LOC100996325, CEP72, TPPP, ZDHHC11, BRD9, TRIP13, LOC100506688, NKD2, SLC12A7, MIR4635, CTD-3080P12.3, SLC6A19, SLC6A18, TERT, MIR4457, CLPTM1L, LINC01511, SLC6A3, LPCAT1, MIR6075, SDHAP3, LOC728613, MIR4277, MRPL36, NDUFS6, LOC101929034, IRX4, CTD-2194D22.4, LOC100506858, IRX2, C5orf38, LOC105374620, LINC01377, LINC01019, LINC01017, IRX1, LOC101929153, LINC01020, LOC105374631, CTD-2297D10.2, ADAMTS16, ICE1, FLJ33360, MED10, UBE2QL1, LINC01018, NSUN2, SRD5A1, LOC100505625, PAPD7, MIR4278, MIR4454, LOC442132, LOC101929261, ADCY2, C5orf49, FASTKD3, MTRR, LOC729506, MIR4458HG, MIR4458, LOC101929284, SEMA5A, MIR4636, CTD-2201E9.1, SNHG18, SNORD123, TAS2R1, LOC285692, FAM173B, CCT5, CMBL, MARCH6, ROPN1L-AS1, ROPN1L, MIR6131, LOC101929412, LOC389273, ANKRD33B, DAP, CTNND2, LINC01194, DNAH5, TRIO, FAM105A, SNORD141B, SNORD141A, OTULIN, ANKH, LOC100130744, MIR4637, LOC101929454, FBXL7, CTD-2350J17.1, MIR887, MARCH11, LOC101929505, ZNF622, FAM134B, LOC101929524, MYO10, LOC285696, BASP1, LOC401177, LOC101929544, LOC102723526, LOC646241, CDH18, GUSBP1, CDH12, SNORA105A, SNORA105B, PMCHL1, PRDM9, C5orf17, CDH10, LOC340107, LOC105374693, CDH9, LINC01021, LOC105374698, LSP1P3, LOC101929645, LOC101929660, LOC101929681, LOC105374704, CDH6, DROSHA, C5orf22, PDZD2, MIR4279, GOLPH3, MTMR12, ZFR, MIR579, SUB1, NPR3, LOC340113, TARS, ADAMTS12, RXFP3, SLC45A2, AMACR, C1QTNF3-AMACR, C1QTNF3, RAI14, MIR7641-2, TTC23L, RAD1, BRIX1, DNAJC21, AGXT2, PRLR, SPEF2, IL7R, CAPSL, LOC100506406, UGT3A1, UGT3A2, LMBRD2, MIR580, SKP2, NADK2, RANBP3L, SLC1A3, NIPBL-AS1, NIPBL, C5orf42, LOC105374727, NUP155, WDR70, GDNF, GDNF-AS1, LOC105374729, LOC101929745, EGFLAM, EGFLAM-AS4, EGFLAM-AS2, LIFR, LIFR-AS1, MIR3650, OSMR-AS1, LINC01265, OSMR, RICTOR, FYB, C9, DAB2, LOC101926940, LINC00603, PTGER4, TTC33, PRKAA1, LOC100506548, RPL37, SNORD72, CARD6, C7, MROH2B, C6, PLCXD3, OXCT1, OXCT1-AS1, C5orf51, FBXO4, LOC101926960, GHR, CCDC152, SEPP1, FLJ32255, LOC648987, ANXA2R, LOC153684, LOC100132356, LOC100506639, ZNF131, NIM1K, HMGCS1, CCL28, TMEM267, C5orf34, PAIP1, NNT-AS1, NNT, FGF10, FGF10-AS1, BRCAT107, BRCAT54, MRPS30, HCN1, EMB, PARP8, LOC100287592, LOC642366, ISL1, PELO, ITGA1, ITGA2, MOCS2, LOC257396, FST, NDUFS4, ARL15, MIR581, MIR4459, LINC01033, HSPB3, SNX18, LOC102467080, ESM1, LOC102467081, GZMK | arr[GRCh37] 5p15.33q11.2(38138_54326830)x3 |
| 1  LM | Gain | 6 | p25.3 | p11.1 | 58,522 | LOC285766, DUSP22, IRF4, EXOC2, HUS1B, LOC101927691, LINC01622, FOXQ1, FOXF2, MIR6720, FOXCUT, FOXC1, GMDS, GMDS-AS1, LINC01600, MYLK4, WRNIP1, SERPINB1, MIR4645, SERPINB9P1, LOC101927730, SERPINB9, SERPINB6, LINC01011, NQO2, HTATSF1P2, LOC101927759, RIPK1, BPHL, TUBB2A, LOC100507194, TUBB2B, PSMG4, SLC22A23, PXDC1, FAM50B, PRPF4B, FAM217A, C6orf201, ECI2, LOC100507506, LOC102724096, MIR7641-2, KU-MEL-3, CDYL, RPP40, LYRM4-AS1, LYRM4, PPP1R3G, MIR3691, FARS2, LOC101927972, LOC101927950, NRN1, F13A1, MIR7853, MIR5683, LY86-AS1, LY86, RREB1, SSR1, CAGE1, RIOK1, DSP, SNRNP48, BMP6, TXNDC5, BLOC1S5-TXNDC5, PIP5K1P1, BLOC1S5, EEF1E1-BLOC1S5, EEF1E1, SCARNA27, SLC35B3, LOC100506207, HULC, TFAP2A, TFAP2A-AS1, LINC00518, MIR5689HG, MIR5689, GCNT2, C6orf52, PAK1IP1, TMEM14C, TMEM14B, MAK, GCM2, SYCP2L, LOC101928191, ELOVL2, ELOVL2-AS1, SMIM13, ERVFRD-1, NEDD9, TMEM170B, ADTRP, LOC101928253, HIVEP1, EDN1, PHACTR1, TBC1D7-LOC100130357, LOC100130357, TBC1D7, GFOD1, SIRT5, NOL7, RANBP9, MCUR1, RNF182, CD83, LINC01108, JARID2, JARID2-AS1, DTNBP1, MYLIP, MIR4639, GMPR, ATXN1, LOC101928433, STMND1, RBM24, CAP2, LOC101928491, FAM8A1, NUP153, LOC105374952, KIF13A, NHLRC1, TPMT, KDM1B, DEK, RNF144B, MIR548A1, LOC101928519, LOC105374960, LOC100506885, ID4, MBOAT1, E2F3, CDKAL1, LINC00581, SOX4, CASC15, NBAT1, PRL, HDGFL1, LOC105374972, NRSN1, DCDC2, KAAG1, MRS2, GPLD1, ALDH5A1, KIAA0319, TDP2, ACOT13, C6orf62, GMNN, C6orf229, FAM65B, CMAHP, LOC101928663, CARMIL1, SCGN, HIST1H2AA, HIST1H2BA, HIST1H2APS1, SLC17A4, SLC17A1, SLC17A3, SLC17A2, TRIM38, HIST1H1A, HIST1H3A, HIST1H4A, HIST1H4B, HIST1H3B, HIST1H2AB, HIST1H2BB, HIST1H3C, HIST1H1C, HFE, HIST1H4C, HIST1H1T, HIST1H2BC, HIST1H2AC, HIST1H1E, HIST1H2BD, HIST1H2BE, HIST1H4D, HIST1H3D, HIST1H2AD, HIST1H2BF, HIST1H4E, HIST1H2BG, HIST1H2AE, HIST1H3E, HIST1H1D, HIST1H4F, HIST1H4G, HIST1H3F, HIST1H2BH, HIST1H3G, HIST1H2BI, HIST1H4H, BTN3A2, BTN2A2, BTN3A1, BTN2A3P, BTN3A3, BTN2A1, LOC285819, BTN1A1, HCG11, HMGN4, LOC105374988, ABT1, ZNF322, GUSBP2, LINC00240, LOC100270746, HIST1H2BJ, HIST1H2AG, HIST1H2BK, HIST1H4I, HIST1H2AH, MIR3143, PRSS16, POM121L2, VN1R10P, ZNF204P, ZNF391, ZNF184, LINC01012, LOC100131289, HIST1H2BL, HIST1H2AI, HIST1H3H, HIST1H2AJ, HIST1H2BM, HIST1H4J, HIST1H4K, HIST1H2AK, HIST1H2BN, HIST1H2AL, HIST1H1B, HIST1H3I, HIST1H4L, HIST1H3J, HIST1H2AM, HIST1H2BO, OR2B2, OR2B6, ZNF165, ZSCAN12P1, ZSCAN16-AS1, ZSCAN16, ZKSCAN8, ZNF192P1, TOB2P1, ZSCAN9, ZKSCAN4, NKAPL, ZSCAN26, PGBD1, ZSCAN31, ZKSCAN3, ZSCAN12, ZSCAN23, GPX6, GPX5, ZBED9, LINC00533, LINC01623, HCG14, TRIM27, LINC01556, HCG16, ZNF311, LOC100129636, OR2W1, OR2B3, OR2J3, OR2J2, LOC101929006, OR14J1, OR5V1, OR12D3, OR12D2, OR11A1, OR10C1, OR2H1, MAS1L, LINC01015, UBD, SNORD32B, OR2H2, GABBR1, MOG, ZFP57, HLA-F, HLA-F-AS1, IFITM4P, HCG4, LOC554223, HLA-G, HLA-H, HCG4B, HLA-A, HCG9, ZNRD1ASP, HLA-J, HCG8, ZNRD1, PPP1R11, RNF39, TRIM31, TRIM31-AS1, TRIM40, TRIM10, TRIM15, TRIM26, HCG17, HLA-L, MIR6891, HCG18, TRIM39, TRIM39-RPP21, RPP21, HLA-E, GNL1, PRR3, ABCF1, MIR877, PPP1R10, MRPS18B, ATAT1, C6orf136, DHX16, PPP1R18, NRM, MDC1, MDC1-AS1, TUBB, FLOT1, IER3, LINC00243, LOC105375014, DDR1, MIR4640, GTF2H4, VARS2, SFTA2, DPCR1, MUC21, MUC22, HCG22, C6orf15, PSORS1C1, CDSN, PSORS1C2, CCHCR1, TCF19, POU5F1, PSORS1C3, HCG27, HLA-C, HLA-B, MICA, HCP5, HCG26, MICB, MCCD1, DDX39B, ATP6V1G2-DDX39B, SNORD117, SNORD84, DDX39B-AS1, ATP6V1G2, NFKBIL1, LTA, TNF, LTB, LST1, NCR3, AIF1, PRRC2A, SNORA38, MIR6832, BAG6, APOM, C6orf47, GPANK1, CSNK2B, LY6G5B, LY6G5C, ABHD16A, MIR4646, LY6G6F, LY6G6E, LY6G6D, LY6G6C, C6orf25, DDAH2, CLIC1, MSH5, MSH5-SAPCD1, SAPCD1, SAPCD1-AS1, VWA7, VARS, LSM2, HSPA1L, HSPA1A, HSPA1B, C6orf48, SNORD48, SNORD52, NEU1, SLC44A4, EHMT2, C2, ZBTB12, C2-AS1, CFB, NELFE, MIR1236, SKIV2L, DXO, STK19, C4A, C4B_2, C4B, CYP21A2, CYP21A1P, TNXA, TNXB, ATF6B, FKBPL, PRRT1, LOC100507547, PPT2, PPT2-EGFL8, EGFL8, AGPAT1, MIR6721, RNF5, RNF5P1, MIR6833, AGER, PBX2, GPSM3, NOTCH4, LOC101929163, C6orf10, HCG23, BTNL2, HLA-DRA, HLA-DRB5, HLA-DRB6, HLA-DRB1, HLA-DQA1, HLA-DQB1, HLA-DQB1-AS1, HLA-DQA2, MIR3135B, HLA-DQB2, HLA-DOB, TAP2, PSMB8, PSMB8-AS1, TAP1, PSMB9, LOC100294145, HLA-DMB, HLA-DMA, BRD2, HLA-DOA, HLA-DPA1, HLA-DPB1, HLA-DPB2, COL11A2, RXRB, SLC39A7, HSD17B8, MIR219A1, RING1, HCG25, VPS52, RPS18, B3GALT4, WDR46, MIR6873, PFDN6, MIR6834, RGL2, TAPBP, ZBTB22, MIR1234, DAXX, KIFC1, PHF1, CUTA, SYNGAP1, MIR5004, ZBTB9, BAK1, GGNBP1, LINC00336, ITPR3, LOC101929188, UQCC2, MIR3934, IP6K3, LEMD2, MLN, LINC01016, MIR7159, MIR1275, GRM4, HMGA1, MIR6835, C6orf1, NUDT3, RPS10-NUDT3, RPS10, PACSIN1, SPDEF, C6orf106, LOC101929243, SNRPC, UHRF1BP1, TAF11, ANKS1A, TCP11, SCUBE3, ZNF76, DEF6, PPARD, FANCE, RPL10A, MIR7111, TEAD3, TULP1, FKBP5, MIR5690, LOC285847, ARMC12, CLPSL2, CLPSL1, CLPS, LHFPL5, SRPK1, SLC26A8, MAPK14, MAPK13, BRPF3, PNPLA1, C6orf222, ETV7, PXT1, KCTD20, STK38, SRSF3, MIR3925, PANDAR, CDKN1A, RAB44, CPNE5, PPIL1, C6orf89, PI16, MTCH1, FGD2, PIM1, TMEM217, TBC1D22B, RNF8, CMTR1, CCDC167, LOC100505530, MIR4462, MDGA1, ZFAND3, BTBD9, GLO1, DNAH8, LOC100131047, GLP1R, SAYSD1, KCNK5, KCNK17, KCNK16, KIF6, DAAM2, LOC100505635, MOCS1, LINC00951, TDRG1, LRFN2, LOC101929555, UNC5CL, TSPO2, APOBEC2, OARD1, NFYA, ADCY10P1, TREML1, TREM2, TREML2, TREML3P, TREML4, TREML5P, TREM1, NCR2, LINC01276, FOXP4-AS1, FOXP4, MIR4641, MDFI, TFEB, PGC, FRS3, PRICKLE4, TOMM6, USP49, MED20, BYSL, CCND3, TAF8, C6orf132, GUCA1A, GUCA1B, MRPS10, TRERF1, UBR2, PRPH2, ATP6V0CP3, TBCC, GLTSCR1L, LOC401261, RPL7L1, C6orf226, PTCRA, CNPY3, CNPY3-GNMT, GNMT, PEX6, PPP2R5D, MEA1, KLHDC3, RRP36, CUL7, MRPL2, KLC4, PTK7, SRF, CUL9, DNPH1, TTBK1, SLC22A7, CRIP3, ZNF318, ABCC10, MIR6780B, DLK2, TJAP1, LRRC73, YIPF3, POLR1C, XPO5, POLH, GTPBP2, MAD2L1BP, RSPH9, MRPS18A, VEGFA, LINC01512, LOC101929705, C6orf223, MRPL14, TMEM63B, CAPN11, LOC101929726, SLC29A1, HSP90AB1, SLC35B2, MIR4647, NFKBIE, TMEM151B, TCTE1, AARS2, SPATS1, CDC5L, MIR4642, LOC105375075, SUPT3H, MIR586, RUNX2, CLIC5, ENPP4, ENPP5, RCAN2, LOC101926915, LOC101926898, CYP39A1, SLC25A27, LOC101926934, TDRD6, PLA2G7, ANKRD66, MEP1A, ADGRF5, LOC101926962, ADGRF1, TNFRSF21, CD2AP, ADGRF2, ADGRF4, OPN5, PTCHD4, MUT, CENPQ, GLYATL3, C6orf141, RHAG, CRISP2, CRISP3, PGK2, LOC101927020, LOC101927048, CRISP1, DEFB133, DEFB114, DEFB113, DEFB110, DEFB112, TFAP2D, TFAP2B, PKHD1, LOC101927082, MIR206, LINCMD1, MIR133B, IL17A, IL17F, MCM3, PAQR8, EFHC1, TRAM2, TRAM2-AS1, LOC730101, TMEM14A, GSTA7P, GSTA2, GSTA1, GSTA5, GSTA3, GSTA4, ICK, FBXO9, GCM1, ELOVL5, MIR5685, RPS16P5, GCLC, LOC101927136, LINC01564, KLHL31, LRRC1, LOC101927189, MLIP-IT1, MLIP, TINAG, FAM83B, HCRTR2, GFRAL, HMGCLL1, BMP5, COL21A1, DST, LOC101930010, BEND6, KIAA1586, ZNF451, LOC101927211, BAG2, RAB23, LOC100506188, PRIM2, MIR548U, GUSBP4, LINC00680-GUSBP4, LINC00680 | arr[GRCh37] 6p25.3p11.1(204908_58727179)x2-3 |
| 1  LM | Gain | 6 | q16.3 | q21 | 5,539 | GRIK2, HACE1, LIN28B-AS1, LIN28B, BVES, BVES-AS1, POPDC3, PREP, PRDM1, ATG5, LOC105377924, AIM1, RTN4IP1, QRSL1, LOC100422737, MIR587, C6orf203, BEND3, PDSS2 | arr[GRCh37] 6q16.3q21(102099978_107638569)x2-3 |
| 1  LM | Gain | 8 | q11.23 | q24.3 | 91,012 | SOX17, RP1, XKR4, SBF1P1, LOC105375843, TMEM68, TGS1, LYN, RPS20, SNORD54, MOS, PLAG1, CHCHD7, SDR16C5, SDR16C6P, PENK, LOC101929415, LINC00968, IMPAD1, LINC01606, LOC286177, LINC00588, LOC101929488, LOC286178, LINC01602, FAM110B, LOC101929528, UBXN2B, CYP7A1, SDCBP, NSMAF, TOX, CA8, LINC01301, RAB2A, CHD7, LOC100130298, CLVS1, ASPH, MIR4470, NKAIN3, UG0898H09, GGH, TTPA, YTHDF3-AS1, YTHDF3, LOC102724612, LINC01289, LOC102724623, MIR124-2HG, MIR124-2, LOC401463, BHLHE22, CYP7B1, LINC00251, LINC01299, ARMC1, MTFR1, PDE7A, DNAJC5B, TRIM55, CRH, LINC00967, RRS1-AS1, RRS1, ADHFE1, C8orf46, MYBL1, VCPIP1, C8orf44, C8orf44-SGK3, SGK3, PTTG3P, MCMDC2, SNHG6, SNORD87, TCF24, PPP1R42, COPS5, CSPP1, ARFGEF1, LOC102724708, CPA6, PREX2, C8orf34-AS1, C8orf34, LINC01592, LINC01603, SULF1, SLCO5A1, PRDM14, NCOA2, LOC101926892, TRAM1, LACTB2-AS1, LACTB2, XKR9, EYA1, MSC, MSC-AS1, TRPA1, LOC392232, KCNB2, LOC101926908, TERF1, SBSPON, C8orf89, RPL7, RDH10, RDH10-AS1, STAU2-AS1, STAU2, UBE2W, TCEB1, TMEM70, LY96, JPH1, GDAP1, MIR5681A, MIR5681B, MIR2052HG, MIR2052, PI15, CRISPLD1, CASC9, HNF4G, LINC01111, ZFHX4-AS1, ZFHX4, MIR3149, PEX2, LOC102724874, PKIA, PKIA-AS1, ZC2HC1A, LOC101241902, IL7, STMN2, HEY1, LINC01607, LOC101927040, MRPS28, TPD52, MIR5708, ZBTB10, ZNF704, PAG1, FABP5, PMP2, FABP9, FABP4, FABP12, IMPA1, SLC10A5, ZFAND1, CHMP4C, SNX16, LOC101927141, LINC01419, RALYL, LRRCC1, LOC102723322, E2F5, C8orf59, CA13, CA1, CA3, CA3-AS1, CA2, REXO1L2P, PSKH2, ATP6V0D2, SLC7A13, WWP1, RMDN1, CPNE3, CNGB3, CNBD1, DCAF4L2, MMP16, LOC101929709, RIPK2, OSGIN2, NBN, DECR1, CALB1, LINC00534, LINC01030, TMEM64, NECAB1, C8orf88, TMEM55A, OTUD6B-AS1, OTUD6B, LRRC69, MIR4661, SLC26A7, RUNX1T1, MIR7641-2, LOC102724710, FLJ46284, TRIQK, MIR8084, C8orf87, LINC00535, FAM92A1, RBM12B, RBM12B-AS1, TMEM67, MIR378D2, PDP1, CDH17, GEM, RAD54B, FSBP, KIAA1429, LOC100288748, ESRP1, DPY19L4, INTS8, CCNE2, TP53INP1, NDUFAF6, LOC105375650, MIR3150B, MIR3150A, PLEKHF2, LINC01298, C8orf37, C8orf37-AS1, LOC100500773, GDF6, UQCRB, MTERF3, PTDSS1, LOC102724804, SDC2, CPQ, LOC101927066, TSPYL5, MTDH, LAPTM4B, MATN2, RPL30, SNORA72, ERICH5, RIDA, POP1, NIPAL2, KCNS2, STK3, OSR2, VPS13B, MIR599, MIR875, COX6C, RGS22, MIR1273A, FBXO43, POLR2K, SPAG1, RNF19A, MIR4471, ANKRD46, SNX31, PABPC1, MIR7705, YWHAZ, FLJ42969, ZNF706, NACAP1, GRHL2, NCALD, LOC104054148, MIR5680, RRM2B, UBR5-AS1, UBR5, ODF1, KLF10, AZIN1, AZIN1-AS1, ATP6V1C1, BAALC-AS2, BAALC, MIR3151, BAALC-AS1, FZD6, CTHRC1, SLC25A32, DCAF13, RIMS2, DCSTAMP, DPYS, LRP12, ZFPM2, ZFPM2-AS1, OXR1, ABRA, ANGPT1, RSPO2, EIF3E, EMC2, TMEM74, TRHR, NUDCD1, ENY2, PKHD1L1, EBAG9, SYBU, LOC100132813, KCNV1, LINC01608, LINC01609, CSMD3, MIR2053, TRPS1, LINC00536, EIF3H, LOC105375713, UTP23, RAD21, RAD21-AS1, MIR3610, AARD, SLC30A8, MED30, EXT1, SAMD12, SAMD12-AS1, TNFRSF11B, COLEC10, LOC101927513, MAL2, MIR548AZ, NOV, ENPP2, TAF2, DSCC1, DEPTOR, COL14A1, MRPL13, MTBP, SNTB1, LOC101927543, HAS2, HAS2-AS1, LOC105375734, LINC01151, ZHX2, DERL1, TBC1D31, FAM83A, FAM83A-AS1, MIR4663, C8orf76, ZHX1-C8orf76, ZHX1, ATAD2, MIR548D1, WDYHV1, FBXO32, KLHL38, ANXA13, FAM91A1, FER1L6, FER1L6-AS1, FER1L6-AS2, LOC101927588, TMEM65, TRMT12, RNF139-AS1, RNF139, TATDN1, MIR6844, NDUFB9, MTSS1, MIR4662B, MIR4662A, LINC00964, ZNF572, LOC105375744, SQLE, KIAA0196, NSMCE2, TRIB1, LINC00861, LOC101927657, FAM84B, PCAT1, PCAT2, PRNCR1, CASC19, CCAT1, CASC21, CASC8, CCAT2, POU5F1B, CASC11, MYC, PVT1, MIR1204, TMEM75, MIR1205, MIR1206, MIR1207, MIR1208, LINC00824, LINC00977, CCDC26, MIR3686, GSDMC, FAM49B, MIR5194, ASAP1, ASAP1-IT2, ASAP1-IT1, ADCY8, EFR3A, OC90, HHLA1, KCNQ3, HPYR1, LRRC6, TMEM71, PHF20L1, TG, SLA, MIR7848, WISP1, NDRG1, ST3GAL1, LOC105375773, LOC101927798, LOC101927822, ZFAT, ZFAT-AS1, MIR30B, MIR30D, NCRNA00250, LOC101927845, LINC01591, KHDRBS3, LOC101927915, FAM135B, COL22A1, KCNK9, TRAPPC9, CHRAC1, AGO2, PTK2, DENND3, SLC45A4, LOC105375787, LINC01300, GPR20, PTP4A3, MROH5, MIR1302-7, MIR4539, MIR4472-1, LINC00051, TSNARE1, ADGRB1, ARC, LOC101928087, JRK, PSCA, LY6K, LOC100288181, THEM6, SLURP1, LYPD2, LYNX1, LY6D, GML, CYP11B1, CYP11B2, LOC100133669, CDC42P3, LY6E, C8orf31, LY6H, GPIHBP1, ZFP41, GLI4, MINCR, ZNF696, TOP1MT, RHPN1-AS1, RHPN1, MAFA-AS1, MAFA, ZC3H3, GSDMD, MROH6, NAPRT, EEF1D, TIGD5, PYCRL, TSTA3, ZNF623, ZNF707, BREA2, CCDC166, LOC101928160, MAPK15, FAM83H, MIR4664, FAM83H-AS1, LOC105375800, SCRIB, MIR937, PUF60, NRBP2, MIR6845, EPPK1, PLEC, MIR661, PARP10, GRINA, SPATC1, OPLAH, MIR6846, EXOSC4, MIR6847, GPAA1, CYC1, SHARPIN, MAF1, WDR97, HGH1, MROH1, MIR7112, SCX, BOP1, HSF1, DGAT1, MIR6848, SCRT1, TMEM249, FBXL6, SLC52A2, LOC101928902, ADCK5, CPSF1, MIR939, MIR6849, SLC39A4, VPS28, TONSL, TONSL-AS1, MIR6893, CYHR1, KIFC2, FOXH1, PPP1R16A, GPT, MFSD3, RECQL4, LRRC14, LRRC24, C8orf82, ARHGAP39, ZNF251, ZNF34, RPL8, MIR6850, ZNF517, ZNF7, COMMD5, ZNF250, ZNF16, ZNF252P, TMED10P1, ZNF252P-AS1, C8orf33 | arr[GRCh37] 8q11.23q24.3(55280830_146292734)x3 |
| 1  LM | Gain | 12 | p13.31 | q12 | 32,680 | EMG1, LPCAT3, C1S, C1R, C1RL, C1RL-AS1, RBP5, CLSTN3, PEX5, ACSM4, CD163L1, CD163, APOBEC1, GDF3, DPPA3, CLEC4C, NANOGNB, NANOG, SLC2A14, SLC2A3, FOXJ2, C3AR1, NECAP1, CLEC4A, POU5F1P3, ZNF705A, FAM66C, FAM90A1, FAM86FP, LOC101927905, LINC00937, CLEC6A, CLEC4D, CLEC4E, AICDA, MFAP5, RIMKLB, A2ML1, PHC1, M6PR, KLRG1, LINC00612, A2M-AS1, A2M, PZP, A2MP1, MIR1244-4, MIR1244-3, MIR1244-1, MIR1244-2, LINC00987, LOC642846, LOC101930452, LOC101928030, DDX12P, KLRB1, LOC374443, CLEC2D, CLECL1, CD69, KLRF1, CLEC2B, KLRF2, CLEC2A, LOC100506159, LOC400002, CLEC12A, CLEC1B, CLEC12B, LOC102724020, CLEC9A, CLEC1A, CLEC7A, OLR1, TMEM52B, GABARAPL1, KLRD1, LOC101928100, KLRK1, KLRC4-KLRK1, KLRC4, KLRC3, KLRC2, KLRC1, KLRA1P, MAGOHB, STYK1, YBX3, LOC101928162, TAS2R7, TAS2R8, TAS2R9, TAS2R10, PRR4, PRH1-PRR4, PRH1, TAS2R13, PRH2, PRH1-TAS2R14, TAS2R14, TAS2R50, TAS2R20, TAS2R19, TAS2R31, TAS2R46, TAS2R43, TAS2R30, SMIM10L1, TAS2R42, PRB3, PRB4, PRB1, PRB2, LINC01252, ETV6, BCL2L14, LRP6, MANSC1, LOH12CR2, BORCS5, DUSP16, CREBL2, GPR19, CDKN1B, APOLD1, MIR613, DDX47, RPL13AP20, GPRC5A, MIR614, GPRC5D, HEBP1, LOC100506314, HTR7P1, FAM234B, MIR7641-2, GSG1, EMP1, LINC01559, GRIN2B, ATF7IP, PLBD1, PLBD1-AS1, GUCY2C, HIST4H4, H2AFJ, WBP11, C12orf60, SMCO3, ART4, MGP, ERP27, ARHGDIB, PDE6H, LINC01489, RERG, RERG-AS1, PTPRO, EPS8, STRAP, DERA, SLC15A5, MGST1, LMO3, SKP1P2, MIR3974, RERGL, PIK3C2G, PLCZ1, CAPZA3, PLEKHA5, AEBP2, LOC100506393, PDE3A, SLCO1C1, SLCO1B3, SLCO1B7, SLCO1B1, SLCO1A2, IAPP, PYROXD1, RECQL, GOLT1B, SPX, GYS2, LDHB, KCNJ8, ABCC9, CMAS, ST8SIA1, C2CD5, LOC105369691, ETNK1, LOC101928441, SOX5, MIR920, LOC101928471, LINC00477, BCAT1, C12orf77, LOC645177, LRMP, CASC1, LYRM5, KRAS, LMNTD1, MIR4302, RASSF8-AS1, RASSF8, BHLHE41, SSPN, ITPR2, ASUN, FGFR1OP2, TM7SF3, MED21, C12orf71, STK38L, ARNTL2, ARNTL2-AS1, SMCO2, PPFIBP1, REP15, MRPS35, MANSC4, KLHL42, PTHLH, CCDC91, FAR2, LOC100506606, ERGIC2, OVCH1-AS1, OVCH1, TMTC1, IPO8, CAPRIN2, LOC645485, LINC00941, TSPAN11, DDX11-AS1, DDX11, FAM60A, FLJ13224, DENND5B, DENND5B-AS1, ETFBKMT, AMN1, H3F3C, LOC105369723, KIAA1551, BICD1, FGD4, DNM1L, YARS2, PKP2, SYT10, ALG10, ALG10B, CPNE8, KIF21A | arr[GRCh37] 12p13.31q12(7096280_39776702)x3 |
| 1  LM | Gain | 12 | q21.31 | q21.33 | 7,251 | CCDC59, METTL25, TMTC2, SLC6A15, TSPAN19, LRRIQ1, ALX1, RASSF9, NTS, MGAT4C, LOC105369879, MKRN9P, C12orf50, C12orf29, CEP290, TMTC3, KITLG, LOC728084, DUSP6, POC1B, GALNT4, POC1B-GALNT4 | arr[GRCh37] 12q21.31q21.33(82687505_89938590)x3 |
| 1  LM | Gain | 13 | q11 | q34 | 96,018 | LINC00417, ANKRD20A9P, LINC00408, LINC00442, TUBA3C, LOC101928697, ANKRD26P3, LINC00421, TPTE2, LINC00350, MPHOSPH8, PSPC1, ZMYM5, ZMYM2, LINC01072, GJA3, GJB2, GJB6, CRYL1, MIR4499, IFT88, IL17D, EEF1AKMT1, XPO4, LINC00367, LATS2, SAP18, SKA3, MRPL57, LINC01046, MIPEPP3, LINC00539, ZDHHC20, MICU2, FGF9, LINC00424, LINC00540, BASP1P1, SGCG, SACS, SACS-AS1, LINC00327, TNFRSF19, MIPEP, C1QTNF9B-AS1, C1QTNF9B, ANKRD20A19P, SPATA13, MIR2276, SPATA13-AS1, C1QTNF9, LINC00566, PARP4, TPTE2P6, ATP12A, RNF17, CENPJ, TPTE2P1, PABPC3, AMER2, LINC00463, LINC01053, MTMR6, NUP58, ATP8A2, SHISA2, RNF6, CDK8, WASF3, GPR12, USP12, USP12-AS1, USP12-AS2, LINC00412, RPL21, RPL21P28, SNORD102, SNORA27, RASL11A, GTF3A, MTIF3, LNX2, POLR1D, GSX1, PDX1-AS1, PDX1, ATP5EP2, LINC00543, CDX2, URAD, FLT3, PAN3-AS1, PAN3, FLT1, POMP, SLC46A3, MTUS2, MTUS2-AS1, SLC7A1, LOC102723345, UBL3, LINC00297, LINC00572, LINC00544, LINC00365, KATNAL1, LINC00426, LINC01058, HMGB1, USPL1, ALOX5AP, LINC00398, LINC00545, TEX26-AS1, MEDAG, TEX26, HSPH1, B3GLCT, RXFP2, EEF1DP3, FRY-AS1, FRY, ZAR1L, BRCA2, N4BP2L1, N4BP2L2, MINOS1P1, N4BP2L2-IT2, PDS5B, LINC00423, KL, STARD13, STARD13-AS, RFC3, LINC00457, NBEA, MAB21L1, LINC00445, DCLK1, SOHLH2, CCDC169-SOHLH2, CCDC169, SPG20, SPG20-AS1, CCNA1, SERTM1, RFXAP, SMAD9, ALG5, EXOSC8, SUPT20H, CSNK1A1L, LINC01048, LINC00547, POSTN, TRPC4, LINC00571, UFM1, LINC00437, LINC00366, FREM2, STOML3, PROSER1, NHLRC3, LHFP, COG6, MIR4305, LINC00332, LINC00548, LINC00598, FOXO1, MIR320D1, MRPS31, SLC25A15, TPTE2P5, MIR621, SUGT1P3, ELF1, WBP4, MIR3168, KBTBD6, LOC101929140, KBTBD7, MTRF1, NAA16, OR7E37P, RGCC, VWA8, MIR5006, VWA8-AS1, DGKH, AKAP11, LOC105370177, TNFSF11, FAM216B, LINC01050, LINC00428, EPSTI1, DNAJC15, LINC00400, ENOX1, ENOX1-AS2, CCDC122, LACC1, LINC00284, LINC00390, SMIM2-AS1, SMIM2, SMIM2-IT1, MIR8079, SERP2, TUSC8, TSC22D1, TSC22D1-AS1, LINC00330, NUFIP1, GPALPP1, LOC101929259, GTF2F2, KCTD4, TPT1, SNORA31, TPT1-AS1, SLC25A30, SLC25A30-AS1, COG3, ERICH6B, LINC01055, SPERT, SIAH3, ZC3H13, CPB2-AS1, CPB2, LCP1, LRRC63, LINC00563, KIAA0226L, LINC01198, LRCH1, ESD, HTR2A, HTR2A-AS1, LINC00562, SUCLA2, NUDT15, MED4, MED4-AS1, ITM2B, LINC00441, RB1, LPAR6, RCBTB2, LINC00462, CYSLTR2, FNDC3A, MLNR, LOC105370203, CDADC1, CAB39L, SETDB2, SETDB2-PHF11, PHF11, RCBTB1, ARL11, EBPL, KPNA3, CTAGE10P, SPRYD7, DLEU2, MIR3613, TRIM13, KCNRG, MIR16-1, MIR15A, DLEU1, ST13P4, DLEU1-AS1, DLEU7, DLEU7-AS1, RNASEH2B-AS1, RNASEH2B, GUCY1B2, LINC00371, FAM124A, SERPINE3, MIR5693, INTS6, INTS6-AS1, MIR4703, WDFY2, DHRS12, LINC00282, CCDC70, ATP7B, ALG11, UTP14C, NEK5, LOC101929657, NEK3, MRPS31P5, LOC103191607, THSD1, VPS36, CKAP2, TPTE2P3, HNRNPA1L2, SUGT1, LECT1, MIR759, PCDH8, OLFM4, LINC01065, LINC00558, LINC00458, MIR1297, MIR5007, PRR20A, PRR20D, PRR20E, PRR20B, PRR20C, PCDH17, LOC101926897, LINC00374, DIAPH3, DIAPH3-AS1, DIAPH3-AS2, LINC00434, TDRD3, LINC00378, MIR3169, PCDH20, LOC101926951, LINC00358, LINC01075, LINC00448, LINC00376, LINC00395, OR7E156P, LOC102723968, LINC01052, MIR548X2, MIR4704, PCDH9, PCDH9-AS2, PCDH9-AS3, PCDH9-AS4, LINC00364, LINC00550, LINC00383, KLHL1, ATXN8OS, LINC00348, DACH1, MZT1, BORA, DIS3, PIBF1, KLF5, LINC00392, KLF12, LINC00381, LINC00347, CTAGE11P, LINC01078, TBC1D4, COMMD6, UCHL3, LMO7-AS1, LMO7, LMO7DN, LMO7DN-IT1, KCTD12, BTF3P11, ACOD1, CLN5, FBXL3, MYCBP2, MYCBP2-AS1, SCEL, SCEL-AS1, LOC100129307, SLAIN1, MIR3665, EDNRB-AS1, EDNRB, LINC01069, LINC00446, RNF219-AS1, POU4F1, RNF219, LINC00331, RBM26, RBM26-AS1, NDFIP2-AS1, NDFIP2, LINC01068, LINC01038, LINC00382, LINC01080, SPRY2, LINC00377, LINC00564, SLITRK1, LINC00333, SNORA107, LINC00375, LINC00351, SLITRK6, LINC00430, MIR4500HG, MIR4500, SLITRK5, LINC00397, LOC105370306, LINC00433, LINC01047, LINC00440, LINC01040, LINC00353, LINC00559, MIR622, LINC01049, LINC00410, LINC00380, LINC00379, MIR17HG, MIR17, MIR18A, MIR19A, MIR20A, MIR19B1, MIR92A1, GPC5, GPC5-AS2, MIR548AS, GPC5-AS1, LINC00363, GPC6, GPC6-AS2, GPC6-AS1, DCT, TGDS, GPR180, LOC101927248, SOX21, SOX21-AS1, LOC101927284, LINC00557, ABCC4, CLDN10, CLDN10-AS1, DZIP1, DNAJC3-AS1, DNAJC3, UGGT2, HS6ST3, MIR4501, LINC00359, OXGR1, LINC00456, MBNL2, RAP2A, IPO5, FARP1, RNF113B, MIR3170, STK24, SLC15A1, DOCK9, DOCK9-AS1, DOCK9-AS2, UBAC2-AS1, UBAC2, GPR18, GPR183, FKSG29, MIR623, LINC01232, LINC00449, TM9SF2, LINC01039, CLYBL, MIR4306, CLYBL-AS2, CLYBL-AS1, LOC101927437, ZIC5, ZIC2, LINC00554, LOC105370333, PCCA, PCCA-AS1, GGACT, TMTC4, NALCN-AS1, LINC00411, NALCN, ITGBL1, FGF14, MIR2681, MIR4705, FGF14-IT1, FGF14-AS1, FGF14-AS2, TPP2, METTL21C, CCDC168, TEX30, KDELC1, BIVM, BIVM-ERCC5, ERCC5, METTL21EP, SLC10A2, LINC01309, DAOA-AS1, DAOA, LINC00343, LINC00460, EFNB2, ARGLU1, LINC00551, LINC00443, FAM155A, MIR1267, FAM155A-IT1, LIG4, ABHD13, TNFSF13B, MYO16, MYO16-AS1, LINC00399, LINC00676, IRS2, LINC00396, COL4A1, COL4A2, MIR8073, COL4A2-AS2, COL4A2-AS1, RAB20, NAXD, CARS2, ING1, LINC00567, LINC00346, ANKRD10, LINC00431, LINC00368, ARHGEF7-AS2, ARHGEF7, ARHGEF7-AS1, LOC101060553, TEX29, LOC105370369, LINC00354, LINC00403, SOX1, LOC100506016, LINC01070, LOC101928730, LINC01043, LINC01044, SPACA7, TUBGCP3, ATP11AUN, ATP11A, ATP11A-AS1, MCF2L-AS1, MCF2L, F7, F10, F10-AS1, PROZ, PCID2, CUL4A, MIR8075, LAMP1, GRTP1, GRTP1-AS1, LOC101928841, ADPRHL1, DCUN1D2, TMCO3, TFDP1, ATP4B, GRK1, LINC00552, TMEM255B, GAS6-AS1, GAS6, GAS6-AS2, LINC00452, LINC00565, RASA3, CDC16, MIR548AR, MIR4502, UPF3A, CHAMP1, LINC01054 | arr[GRCh37] 13q11q34(19084822_115103150)x3 |
| 1  LM | Gain | 17 | q24.1 | q24.3 | 6,266 | SMURF2, LOC146880, MIR6080, PLEKHM1P1, MIR4315-2, MIR4315-1, LRRC37A3, AMZ2P1, GNA13, LOC100507002, RGS9, CRAT40, AXIN2, CEP112, APOH, PRKCA, PRKCA-AS1, MIR634, CACNG5, CACNG4, CACNG1, HELZ, LOC101928021, PSMD12, PITPNC1, MIR548AA2, MIR548D2, NOL11, SNORA38B, BPTF, C17orf58, KPNA2, LINC00674, LOC440461, AMZ2, ARSG, SLC16A6, PRKAR1A, WIPI1, MIR635, FAM20A, LINC01482, ABCA8, ABCA9, ABCA9-AS1, ABCA6, MIR4524B, MIR4524A, ABCA10, PRO1804, ABCA5, MAP2K6, LINC01483, LINC01497, LINC01028, KCNJ16, KCNJ2-AS1, KCNJ2 | arr[GRCh37] 17q24.1q24.3(62617312_68883534)x2-3 |
| 1  LM | Gain | 19 | p13.2 | q13.43 | 51,595 | ADGRE4P, FLJ25758, MBD3L5, MBD3L4, MBD3L2, MBD3L3, ZNF557, INSR, ARHGEF18, LOC100128573, PEX11G, C19orf45, ZNF358, MCOLN1, PNPLA6, CAMSAP3, MIR6792, XAB2, PET100, PCP2, STXBP2, RETN, MCEMP1, TRAPPC5, FCER2, CLEC4G, CD209, CLEC4M, CLEC4GP1, EVI5L, PRR36, LYPLA2P2, LRRC8E, MAP2K7, TGFBR3L, SNAPC2, CTXN1, TIMM44, ELAVL1, CCL25, FBN3, CERS4, CD320, NDUFA7, RPS28, KANK3, ANGPTL4, RAB11B-AS1, MIR4999, RAB11B, MARCH2, HNRNPM, PRAM1, ZNF414, MYO1F, ADAMTS10, ACTL9, OR2Z1, ZNF558, MBD3L1, MUC16, OR1M1, OR7G2, OR7G1, OR7G3, ZNF317, OR7D2, OR7D4, OR7E24, ZNF699, ZNF559, ZNF559-ZNF177, ZNF177, ZNF266, ZNF560, ZNF426, LOC101928238, ZNF121, ZNF561, ZNF561-AS1, ZNF562, ZNF846, FBXL12, UBL5, PIN1, OLFM2, COL5A3, RDH8, MIR5589, C3P1, C19orf66, ANGPTL6, PPAN, PPAN-P2RY11, SNORD105, SNORD105B, P2RY11, EIF3G, DNMT1, S1PR2, MIR4322, MRPL4, ICAM1, ICAM4, ICAM5, ZGLP1, FDX1L, RAVER1, ICAM3, TYK2, CDC37, MIR1181, PDE4A, KEAP1, S1PR5, ATG4D, MIR1238, KRI1, CDKN2D, AP1M2, SLC44A2, ILF3-AS1, ILF3, QTRT1, DNM2, MIR638, MIR4748, MIR199A1, MIR6793, TMED1, C19orf38, CARM1, YIPF2, C19orf52, SMARCA4, LDLR, MIR6886, SPC24, KANK2, DOCK6, LOC105372273, ANGPTL8, TSPAN16, RAB3D, TMEM205, CCDC159, PLPPR2, SWSAP1, EPOR, RGL3, CCDC151, PRKCSH, ELAVL3, ZNF653, MIR7974, ECSIT, CNN1, ELOF1, ACP5, ZNF627, ZNF833P, ZNF823, ZNF441, ZNF491, ZNF440, ZNF439, ZNF69, ZNF700, ZNF763, LOC101928464, ZNF433, ZNF878, ZNF844, ZNF788, ZNF20, ZNF625-ZNF20, ZNF625, ZNF136, LOC100289333, ZNF44, ZNF563, ZNF442, ZNF799, ZNF443, ZNF709, ZNF564, ZNF490, ZNF791, MAN2B1, WDR83, WDR83OS, DHPS, LOC105372280, FBXW9, TNPO2, SNORD135, SNORD41, C19orf43, ASNA1, BEST2, HOOK2, MIR5684, JUNB, PRDX2, RNASEH2A, RTBDN, MAST1, MIR6794, DNASE2, KLF1, GCDH, SYCE2, FARSA, CALR, MIR6515, RAD23A, GADD45GIP1, DAND5, NFIX, LYL1, TRMT1, NACC1, STX10, IER2, CACNA1A, CCDC130, MRI1, C19orf53, ZSWIM4, LOC284454, MIR24-2, MIR27A, MIR23A, MIR181C, MIR181D, NANOS3, C19orf57, CC2D1A, PODNL1, DCAF15, RFX1, RLN3, IL27RA, PALM3, LOC113230, MIR1199, C19orf67, SAMD1, PRKACA, ASF1B, LOC100507373, ADGRL1, LOC105372288, LOC101928845, ADGRE5, DDX39A, PKN1, PTGER1, GIPC1, DNAJB1, MIR639, TECR, NDUFB7, CLEC17A, ADGRE3, SNORA104, ZNF333, ADGRE2, OR7C1, OR7A5, OR7A10, OR7A17, OR7C2, SLC1A6, CCDC105, CASP14, OR1I1, SYDE1, ILVBL, NOTCH3, MIR6795, EPHX3, BRD4, AKAP8, AKAP8L, WIZ, MIR1470, RASAL3, PGLYRP2, CYP4F22, CYP4F8, CYP4F3, CYP4F12, OR10H2, OR10H3, CYP4F24P, OR10H5, OR10H1, UCA1, LOC102724279, CYP4F2, CYP4F11, OR10H4, LINC00661, LINC00905, TPM4, RAB8A, HSH2D, CIB3, FAM32A, AP1M1, KLF2, EPS15L1, CALR3, C19orf44, CHERP, SLC35E1, MED26, SMIM7, TMEM38A, NWD1, SIN3B, F2RL3, CPAMD8, HAUS8, MYO9B, USE1, OCEL1, NR2F6, USHBP1, BABAM1, ANKLE1, ABHD8, MRPL34, DDA1, ANO8, GTPBP3, PLVAP, BST2, BISPR, MVB12A, TMEM221, NXNL1, SLC27A1, PGLS, FAM129C, COLGALT1, UNC13A, MAP1S, FCHO1, B3GNT3, INSL3, JAK3, RPL18A, SNORA68, SLC5A5, CCDC124, KCNN1, ARRDC2, IL12RB1, MAST3, PIK3R2, IFI30, MPV17L2, RAB3A, LOC102725254, PDE4C, LOC729966, KIAA1683, JUND, MIR3188, LSM4, PGPEP1, GDF15, MIR3189, LRRC25, SSBP4, ISYNA1, ELL, FKBP8, KXD1, UBA52, C19orf60, CRLF1, TMEM59L, KLHL26, CRTC1, COMP, UPF1, GDF1, CERS1, COPE, DDX49, HOMER3, LOC102724360, SUGP2, ARMC6, SLC25A42, TMEM161A, MEF2B, BORCS8-MEF2B, BORCS8, RFXANK, NR2C2AP, NCAN, HAPLN4, TM6SF2, SUGP1, MAU2, GATAD2A, MIR640, TSSK6, NDUFA13, YJEFN3, CILP2, PBX4, LPAR2, GMIP, ATP13A1, ZNF101, ZNF14, LINC00663, ZNF506, ZNF253, ZNF93, ZNF682, ZNF90, ZNF486, MIR1270, ZNF826P, ZNF737, ZNF626, ZNF85, ZNF430, ZNF714, ZNF431, ZNF708, ZNF738, ZNF493, LINC00664, ZNF429, ZNF100, LOC641367, ZNF43, ZNF208, ZNF257, ZNF676, ZNF729, ZNF98, LOC101929124, LINC01233, GOLGA2P9, LOC100996349, LOC374890, ZNF492, ZNF99, ZNF728, LOC101929164, LOC101929144, ZNF730, ZNF724P, IPO5P1, ZNF91, LINC01224, ZNF675, ZNF681, RPSAP58, ZNF726, ZNF254, HAVCR1P1, LINC00662, LOC101927151, LOC100420587, LOC102724908, LINC00906, LOC102724958, LINC01532, UQCRFS1, LOC284395, VSTM2B, POP4, PLEKHF1, C19orf12, CCNE1, URI1, ZNF536, TSHZ3, THEG5, LINC01533, LOC101927411, ZNF507, LOC400684, DPY19L3, PDCD5, ANKRD27, RGS9BP, NUDT19, TDRD12, SLC7A9, CEP89, FAAP24, RHPN2, GPATCH1, WDR88, LRP3, SLC7A10, CEBPA, CEBPA-AS1, CEBPG, PEPD, CHST8, KCTD15, LSM14A, KIAA0355, GPI, PDCD2L, UBA2, WTIP, SCGB1B2P, SCGB2B2, SCGB2B3P, ZNF302, ZNF181, ZNF599, LOC400685, LINC00904, ZNF30-AS1, ZNF30, ZNF792, GRAMD1A, SCN1B, HPN, HPN-AS1, FXYD3, MIR6887, LGI4, FXYD1, FXYD7, FXYD5, FAM187B, LSR, USF2, HAMP, MAG, CD22, MIR5196, FFAR1, FFAR3, LINC01531, FFAR2, KRTDAP, DMKN, SBSN, GAPDHS, TMEM147-AS1, TMEM147, ATP4A, LOC102723617, HAUS5, RBM42, ETV2, COX6B1, UPK1A, UPK1A-AS1, ZBTB32, KMT2B, IGFLR1, U2AF1L4, PSENEN, LIN37, HSPB6, PROSER3, ARHGAP33, LINC01529, PRODH2, NPHS1, KIRREL2, APLP1, NFKBID, HCST, TYROBP, LRFN3, LOC105372383, SDHAF1, SYNE4, ALKBH6, LOC101927572, CLIP3, THAP8, WDR62, OVOL3, POLR2I, TBCB, CAPNS1, COX7A1, ZNF565, ZNF146, LOC100134317, LINC00665, ZFP14, ZFP82, LOC644189, ZNF566, LOC728752, ZNF260, ZNF529, ZNF529-AS1, ZNF382, ZNF461, LINC01534, ZNF567, ZNF850, LOC728485, ZNF790-AS1, ZNF790, ZNF345, ZNF829, ZNF568, ZNF420, ZNF585A, ZNF585B, ZNF383, LINC01535, LOC284412, HKR1, ZNF527, ZNF569, ZNF570, ZNF793-AS1, ZNF793, ZNF571-AS1, ZNF540, ZNF571, ZFP30, ZNF781, ZNF607, ZNF573, LOC644554, LOC100631378, WDR87, SIPA1L3, DPF1, PPP1R14A, SPINT2, YIF1B, C19orf33, KCNK6, CATSPERG, PSMD8, GGN, SPRED3, FAM98C, RASGRP4, RYR1, MAP4K1, LOC105372397, EIF3K, ACTN4, CAPN12, LGALS7, LGALS7B, LGALS4, ECH1, HNRNPL, RINL, SIRT2, NFKBIB, CCER2, SARS2, MRPS12, FBXO17, FBXO27, ACP7, PAK4, NCCRP1, SYCN, IFNL3, IFNL4, IFNL2, IFNL1, LRFN1, GMFG, SAMD4B, PAF1, MED29, ZFP36, MIR4530, PLEKHG2, RPS16, SUPT5H, TIMM50, DLL3, SELV, EID2B, EID2, LGALS13, LOC100129935, LGALS16, LGALS17A, LGALS14, CLC, LEUTX, DYRK1B, MIR6719, FBL, FCGBP, PSMC4, ZNF546, ZNF780B, ZNF780A, MAP3K10, TTC9B, CNTD2, AKT2, MIR641, C19orf47, PLD3, MIR6796, HIPK4, PRX, SERTAD1, SERTAD3, BLVRB, SPTBN4, SHKBP1, LTBP4, NUMBL, ADCK4, ITPKC, C19orf54, SNRPA, MIA, MIA-RAB4B, RAB4B, RAB4B-EGLN2, EGLN2, CYP2A6, CYP2A7, CYP2G1P, CYP2B7P, CYP2B6, CYP2A13, CYP2F1, CYP2S1, AXL, HNRNPUL1, CCDC97, TGFB1, B9D2, TMEM91, EXOSC5, BCKDHA, B3GNT8, ATP5SL, ERICH4, PCAT19, LINC01480, CEACAM21, CEACAM4, CEACAM7, CEACAM5, CEACAM6, CEACAM3, LYPD4, DMRTC2, RPS19, MIR6797, CD79A, ARHGEF1, LOC100505585, RABAC1, ATP1A3, GRIK5, ZNF574, POU2F2, LOC100505622, MIR4323, DEDD2, ZNF526, GSK3A, ERF, CIC, PAFAH1B3, PRR19, TMEM145, MEGF8, MIR8077, CNFN, LOC101930071, LIPE-AS1, LIPE, CXCL17, CEACAM1, CEACAM8, PSG3, PSG8, LOC100289650, PSG10P, PSG1, PSG6, PSG7, PSG11, PSG2, PSG5, PSG4, LOC284344, PSG9, PRG1, CD177, TEX101, LYPD3, PHLDB3, ETHE1, ZNF575, XRCC1, PINLYP, IRGQ, ZNF576, ZNF428, SRRM5, CADM4, PLAUR, IRGC, SMG9, KCNN4, LYPD5, ZNF283, ZNF404, LOC100505715, ZNF45, ZNF221, ZNF155, LOC101928063, ZNF230, ZNF222, ZNF223, ZNF284, ZNF224, LOC100379224, ZNF225, ZNF234, ZNF226, ZNF227, ZNF233, ZNF235, ZNF112, ZNF285, ZNF229, ZNF180, CEACAM20, CEACAM22P, IGSF23, PVR, MIR4531, CEACAM19, CEACAM16, BCL3, MIR8085, CBLC, BCAM, NECTIN2, TOMM40, APOE, APOC1, APOC1P1, APOC4, APOC4-APOC2, APOC2, CLPTM1, RELB, CLASRP, ZNF296, GEMIN7, LOC105372419, PPP1R37, NKPD1, TRAPPC6A, BLOC1S3, EXOC3L2, MARK4, CKM, KLC3, ERCC2, PPP1R13L, CD3EAP, ERCC1, MIR6088, FOSB, RTN2, PPM1N, VASP, OPA3, GPR4, EML2, MIR330, EML2-AS1, GIPR, MIR642A, MIR642B, SNRPD2, QPCTL, FBXO46, BHMG1, SIX5, DMPK, DMWD, RSPH6A, SYMPK, FOXA3, IRF2BP1, MYPOP, NANOS2, NOVA2, CCDC61, MIR769, PGLYRP1, IGFL4, LOC400706, IGFL3, IGFL2, LOC645553, LOC93429, IGFL1, HIF3A, PPP5C, CCDC8, PNMAL1, PPP5D1, PNMAL2, CALM3, PTGIR, GNG8, DACT3, DACT3-AS1, PRKD2, MIR320E, STRN4, FKRP, SLC1A5, SNAR-E, AP2S1, ARHGAP35, NPAS1, TMEM160, ZC3H4, SAE1, BBC3, MIR3190, MIR3191, CCDC9, INAFM1, C5AR1, C5AR2, DHX34, MEIS3, SLC8A2, KPTN, NAPA-AS1, NAPA, ZNF541, GLTSCR1, EHD2, GLTSCR2, SNORD23, GLTSCR2-AS1, SEPW1, TPRX1, CRX, SULT2A1, SNAR-A13, SNAR-A12, SNAR-C2, SNAR-C1, SNAR-C5, SNAR-A1, SNAR-A2, SNAR-A10, SNAR-A3, SNAR-A8, SNAR-A4, SNAR-A11, SNAR-A7, SNAR-A9, SNAR-A5, SNAR-A6, SNAR-A14, SNAR-C4, SNAR-C3, BSPH1, ELSPBP1, CABP5, PLA2G4C, PLA2G4C-AS1, LIG1, C19orf68, CARD8, CARD8-AS1, ZNF114, CCDC114, EMP3, TMEM143, SYNGR4, KDELR1, GRIN2D, GRWD1, KCNJ14, CYTH2, LMTK3, SULT2B1, FAM83E, SPACA4, RPL18, SPHK2, DBP, CA11, SEC1P, NTN5, FUT2, LOC105447645, MAMSTR, RASIP1, IZUMO1, FUT1, FGF21, BCAT2, HSD17B14, PLEKHA4, PPP1R15A, TULP2, NUCB1, NUCB1-AS1, DHDH, BAX, FTL, GYS1, RUVBL2, MIR6798, LHB, LOC101059948, CGB3, SNAR-G2, CGB2, CGB1, SNAR-G1, CGB5, CGB8, CGB7, NTF4, KCNA7, SNRNP70, LIN7B, C19orf73, PPFIA3, HRC, TRPM4, SLC6A16, MIR4324, CD37, TEAD2, DKKL1, LOC101928295, CCDC155, PTH2, GFY, SLC17A7, PIH1D1, ALDH16A1, FLT3LG, RPL13A, RPL13AP5, SNORD32A, SNORD33, SNORD34, SNORD35A, RPS11, SNORD35B, MIR150, FCGRT, RCN3, NOSIP, PRRG2, PRR12, RRAS, SCAF1, IRF3, BCL2L12, PRMT1, MIR5088, ADM5, CPT1C, TSKS, AP2A1, MIR6799, FUZ, MED25, MIR6800, PTOV1-AS1, PTOV1, MIR4749, PTOV1-AS2, PNKP, AKT1S1, TBC1D17, MIR4750, IL4I1, NUP62, ATF5, MIR4751, SIGLEC11, SIGLEC16, VRK3, ZNF473, FLJ26850, SNAR-B1, SNAR-B2, SNAR-D, IZUMO2, MYH14, KCNC3, NAPSB, NAPSA, NR1H2, POLD1, SPIB, MYBPC2, FAM71E1, EMC10, JOSD2, ASPDH, LRRC4B, SNAR-F, SYT3, C19orf81, SHANK1, CLEC11A, GPR32, LOC105372440, ACPT, C19orf48, SNORD88B, SNORD88A, SNORD88C, MGC45922, KLK1, KLK15, LOC105372441, KLK3, KLK2, KLKP1, KLK4, KLK5, KLK6, KLK7, KLK8, KLK9, KLK10, KLK11, KLK12, KLK13, KLK14, CTU1, SIGLEC9, SIGLEC7, LOC101928517, SIGLEC17P, MIR8074, CD33, SIGLECL1, LOC105372444, IGLON5, VSIG10L, ETFB, CLDND2, NKG7, LIM2, C19orf84, SIGLEC10, LOC100129083, SIGLEC8, CEACAM18, SIGLEC12, SIGLEC6, ZNF175, LINC01530, SIGLEC5, SIGLEC14, SPACA6P-AS, SPACA6, MIR99B, MIRLET7E, MIR125A, HAS1, FPR1, FPR2, FPR3, ZNF577, ZNF649-AS1, ZNF649, ZNF613, ZNF350-AS1, ZNF350, ZNF615, ZNF614, ZNF432, ZNF841, ZNF616, ZNF836, PPP2R1A, MIR6801, ZNF766, MIR643, ZNF480, ZNF610, ZNF880, ZNF528-AS1, ZNF528, ZNF534, ZNF578, ZNF808, ZNF701, ZNF137P, ZNF83, ZNF611, ZNF600, ZNF28, ZNF468, ZNF320, ZNF888, ZNF321P, ZNF816-ZNF321P, ZNF816, ZNF702P, ERVV-1, ERVV-2, ZNF160, ZNF415, ZNF347, ZNF665, ZNF818P, ZNF677, VN1R2, VN1R4, FAM90A27P, BIRC8, ZNF845, ZNF525, ZNF765, TPM3P9, ZNF761, ZNF813, ZNF331, LOC284379, DPRX, MIR512-2, MIR512-1, MIR1323, MIR498, MIR520E, MIR515-2, MIR515-1, MIR519E, MIR520F, MIR519C, MIR1283-1, MIR520A, MIR526B, MIR519B, MIR525, MIR523, MIR518F, MIR520B, MIR518B, MIR526A1, MIR520C, MIR518C, MIR524, MIR517A, MIR519D, MIR521-2, MIR520D, MIR517B, MIR520G, MIR516B2, MIR526A2, MIR518E, MIR518A1, MIR518D, MIR516B1, MIR518A2, MIR517C, MIR520H, MIR521-1, MIR522, MIR519A1, MIR527, MIR516A1, MIR1283-2, MIR516A2, MIR519A2, MIR371A, MIR371B, MIR372, MIR373, NLRP12, MYADM, PRKCG, CACNG7, CACNG8, MIR935, CACNG6, VSTM1, TARM1, OSCAR, NDUFA3, TFPT, PRPF31, CNOT3, LENG1, TMC4, MBOAT7, TSEN34, RPS9, LILRB3, LILRA6, LILRB5, LILRB2, MIR4752, LILRA3, LILRA5, LILRA4, LAIR1, TTYH1, LENG8-AS1, LENG8, LENG9, CDC42EP5, LAIR2, KIR3DX1, LILRA2, LILRA1, LILRB1, MIR8061, LILRB4, LILRP2, KIR3DL3, KIR2DL3, LOC101928804, KIR2DL1, KIR2DL4, KIR3DL1, KIR2DS4, KIR3DL2, FCAR, NCR1, NLRP7, NLRP2, GP6, RDH13, EPS8L1, PPP1R12C, MIR7975, TNNT1, TNNI3, DNAAF3, SYT5, PTPRH, TMEM86B, PPP6R1, MIR6804, MIR6802, MIR6803, HSPBP1, BRSK1, TMEM150B, KMT5C, COX6B2, FAM71E2, IL11, TMEM190, TMEM238, RPL28, MIR6805, UBE2S, SHISA7, ISOC2, ZNF628, NAT14, SSC5D, SBK2, SBK3, ZNF579, FIZ1, ZNF524, ZNF865, ZNF784, ZNF580, ZNF581, CCDC106, U2AF2, EPN1, NLRP9, RFPL4A, RFPL4AL1, NLRP11, NLRP4, NLRP13, NLRP8, NLRP5, LOC101928886, ZNF787, ZNF444, GALP, ZSCAN5B, ZSCAN5A, ZNF542P, ZNF582, ZNF582-AS1, ZNF583, ZNF667, ZNF667-AS1, ZNF471, ZFP28, ZNF470, ZNF71, SMIM17, ZNF835, ZIM2-AS1, ZIM2, PEG3, PEG3-AS1, MIMT1, USP29, ZIM3, DUXA, ZNF264, AURKC, ZNF805, LOC105372476, ZNF460, ZNF543, ZNF304, ZNF547, TRAPPC2B, ZNF548, ZNF17, ZNF749, VN1R1, ZNF772, ZNF419, ZNF773, ZNF549, ZNF550, ZNF416, ZIK1, ZNF530, ZNF134, ZNF211, ZSCAN4, ZNF551, ZNF154, ZNF671, ZNF776, ZNF586, ZNF552, FKBP1AP1, ZNF587B, ZNF587, ZNF814, ZNF417, ZNF418, ZNF256, C19orf18, ZNF606, LOC100128398 | arr[GRCh37] 19p13.2q13.43(6943409_58538762)x3 |
| 1  LM | Gain | 20 | p12.1 | q13.33 | 45,769 | PCSK2, BFSP1, DSTN, RRBP1, BANF2, SNX5, SNORD17, MGME1, OVOL2, PET117, KAT14, ZNF133, LINC00851, DZANK1, POLR3F, MIR3192, RBBP9, SEC23B, LINC00493, DTD1, LOC101929526, LINC00652, LOC100270804, C20orf78, SCP2D1, SLC24A3, LOC100130264, RIN2, NAA20, CRNKL1, CFAP61, INSM1, RALGAPA2, KIZ, KIZ-AS1, XRN2, NKX2-4, NKX2-2, LOC101929625, LOC101929608, PAX1, LINC01432, LINC01427, LOC284788, LINC00261, FOXA2, LINC01384, SSTR4, THBD, CD93, LINC00656, NXT1, LINC01431, GZF1, NAPB, CSTL1, CST11, CST8, CST13P, CST9L, CST9, CST3, CST4, CST1, CST2, CST5, GGTLC1, FLJ33581, SYNDIG1, CST7, APMAP, ACSS1, VSX1, LOC284798, LOC101926889, ENTPD6, PYGB, ABHD12, GINS1, NINL, NANP, ZNF337-AS1, ZNF337, LOC105372582, LOC105379511, FAM182B, LOC101926935, LOC101926955, LOC100134868, FAM182A, NCOR1P1, MIR663AHG, MIR663A, LINC01598, FRG1BP, FRG1DP, MLLT10P1, DEFB115, DEFB116, DEFB118, DEFB119, DEFB121, DEFB122, DEFB123, DEFB124, REM1, LINC00028, HM13, HM13-AS1, ID1, MIR3193, COX4I2, BCL2L1, ABALON, TPX2, MYLK2, FOXS1, DUSP15, TTLL9, PDRG1, XKR7, MIR7641-2, CCM2L, HCK, TM9SF4, TSPY26P, PLAGL2, POFUT1, MIR1825, KIF3B, ASXL1, NOL4L, LOC101929698, LOC149950, C20orf203, COMMD7, DNMT3B, MAPRE1, SUN5, BPIFB2, BPIFB6, BPIFB3, BPIFB4, BPIFA2, BPIFA4P, BPIFA3, BPIFA1, BPIFB1, CDK5RAP1, SNTA1, CBFA2T2, NECAB3, C20orf144, ACTL10, E2F1, PXMP4, ZNF341, ZNF341-AS1, CHMP4B, RALY-AS1, RALY, MIR4755, EIF2S2, ASIP, AHCY, ITCH, MIR644A, DYNLRB1, MAP1LC3A, PIGU, TP53INP2, NCOA6, HMGB3P1, GGT7, ACSS2, GSS, MYH7B, MIR499A, MIR499B, TRPC4AP, EDEM2, PROCR, MMP24, MMP24-AS1, EIF6, FAM83C-AS1, FAM83C, UQCC1, GDF5, MIR1289-1, CEP250, C20orf173, ERGIC3, FER1L4, SPAG4, CPNE1, RBM12, NFS1, ROMO1, RBM39, PHF20, SCAND1, CNBD2, NORAD, EPB41L1, AAR2, DLGAP4, DLGAP4-AS1, MYL9, TGIF2, TGIF2-C20orf24, C20orf24, SLA2, NDRG3, DSN1, SOGA1, TLDC2, SAMHD1, RBL1, MROH8, RPN2, GHRH, MANBAL, SRC, BLCAP, NNAT, LINC00489, LOC100287792, CTNNBL1, VSTM2L, TTI1, RPRD1B, TGM2, KIAA1755, LOC149684, BPI, LBP, SNHG17, SNORA71B, SNORA71A, SNORA71C, SNORA71D, SNHG11, SNORA71E, SNORA60, RALGAPB, MIR548O2, ADIG, ARHGAP40, SLC32A1, ACTR5, PPP1R16B, FAM83D, DHX35, LOC339568, LINC01370, MAFB, LOC100128988, TOP1, PLCG1-AS1, PLCG1, MIR6871, ZHX3, LPIN3, EMILIN3, CHD6, PTPRT, LOC101927159, SRSF6, L3MBTL1, SGK2, IFT52, MYBL2, GTSF1L, LOC105372626, TOX2, JPH2, OSER1, OSER1-AS1, GDAP1L1, FITM2, R3HDML, HNF4A, HNF4A-AS1, MIR3646, LINC01430, LINC01620, TTPAL, SERINC3, PKIG, ADA, LINC01260, KCNK15-AS1, WISP2, KCNK15, RIMS4, YWHAB, PABPC1L, TOMM34, STK4-AS1, STK4, KCNS1, WFDC5, WFDC12, PI3, SEMG1, SEMG2, SLPI, MATN4, RBPJL, SDC4, SYS1, SYS1-DBNDD2, TP53TG5, DBNDD2, PIGT, MIR6812, WFDC2, SPINT3, WFDC6, EPPIN-WFDC6, EPPIN, WFDC8, WFDC9, WFDC10A, WFDC11, WFDC10B, WFDC13, MIR3617, SPINT4, WFDC3, DNTTIP1, UBE2C, TNNC2, SNX21, ACOT8, ZSWIM3, ZSWIM1, SPATA25, NEURL2, CTSA, PLTP, PCIF1, ZNF335, MMP9, SLC12A5, NCOA5, CD40, CDH22, SLC35C2, ELMO2, ZNF663P, MKRN7P, ZNF334, OCSTAMP, SLC13A3, TP53RK, SLC2A10, EYA2, MIR3616, ZMYND8, LOC100131496, LOC101927377, NCOA3, SULF2, LINC01522, LINC01523, LINC00494, PREX1, ARFGEF2, CSE1L-AS1, CSE1L, STAU1, DDX27, ZNFX1, ZFAS1, SNORD12C, SNORD12B, SNORD12, KCNB1, PTGIS, B4GALT5, SLC9A8, SPATA2, LOC105372653, RNF114, SNAI1, TRERNA1, UBE2V1, TMEM189-UBE2V1, TMEM189, LINC01273, CEBPB-AS1, CEBPB, LINC01272, LINC01270, LINC01271, PTPN1, MIR645, FAM65C, MIR1302-5, LOC100506175, PARD6B, BCAS4, ADNP, ADNP-AS1, DPM1, MOCS3, KCNG1, NFATC2, MIR3194, ATP9A, SALL4, LINC01429, ZFP64, LINC01524, TSHZ2, LOC101927770, ZNF217, LOC105372672, SUMO1P1, BCAS1, MIR4756, CYP24A1, PFDN4, DOK5, LINC01441, LINC01440, CBLN4, MC3R, FAM210B, AURKA, CSTF1, CASS4, RTFDC1, GCNT7, FAM209A, FAM209B, LOC105372682, TFAP2C, BMP7, BMP7-AS1, MIR4325, SPO11, RAE1, MTRNR2L3, RBM38, CTCFL, PCK1, ZBP1, PMEPA1, NKILA, MIR4532, C20orf85, ANKRD60, PPP4R1L, RAB22A, VAPB, APCDD1L, APCDD1L-AS1, LOC79160, STX16, STX16-NPEPL1, NPEPL1, LOC105372695, MIR296, MIR298, GNAS-AS1, GNAS, LOC101927932, NELFCD, CTSZ, TUBB1, ATP5E, SLMO2-ATP5E, PRELID3B, ZNF831, EDN3, PHACTR3, LOC100506384, SYCP2, FAM217B, PPP1R3D, CDH26, C20orf197, LOC729296, MIR646HG, MIR646, LOC101928048, MIR4533, MIR548AG2, LOC100506470, CDH4, MIR1257, TAF4, MIR3195, LSM14B, PSMA7, SS18L1, MTG2, HRH3, OSBPL2, ADRM1, LAMA5, MIR4758, LAMA5-AS1, RPS21, CABLES2, RBBP8NL, GATA5, C20orf166-AS1, MIR1-1HG, MIR1-1, MIR133A2, SLCO4A1, SLCO4A1-AS1, NTSR1, LINC00659, MRGBP, OGFR-AS1, OGFR, COL9A3, TCFL5, DPH3P1, DIDO1, GID8, SLC17A9, BHLHE23, LOC63930, LINC00029, LINC01056, HAR1B, HAR1A, MIR124-3, YTHDF1, BIRC7, MIR3196, NKAIN4, FLJ16779, ARFGAP1, MIR4326, COL20A1, CHRNA4, LOC100130587, KCNQ2, EEF1A2, PPDPF, PTK6, SRMS, FNDC11, HELZ2, GMEB2, LOC100505771, STMN3, RTEL1, RTEL1-TNFRSF6B, TNFRSF6B, ARFRP1, ZGPAT, LIME1, SLC2A4RG, ZBTB46, ZBTB46-AS1, ABHD16B, TPD52L2, DNAJC5, MIR941-1, MIR941-4, MIR941-3, MIR941-2, MIR941-5, UCKL1, MIR1914, MIR647, UCKL1-AS1, ZNF512B, SAMD10, PRPF6, LINC00176, SOX18, TCEA2, RGS19, MIR6813, OPRL1, LKAAEAR1, NPBWR2, MYT1, PCMTD2 | arr[GRCh37] 20p12.1q13.33(17143374_62912463)x3 |
| 1  LM | Loss | 8 | p23.3 | p12 | 29,343 | RPL23AP53, ZNF596, FAM87A, FBXO25, TDRP, ERICH1, ERICH1-AS1, LOC401442, LOC286083, DLGAP2, DLGAP2-AS1, LOC101927752, CLN8, MIR3674, MIR596, ARHGEF10, LOC101928058, KBTBD11-OT1, KBTBD11, MYOM2, MIR7160, LOC101927815, CSMD1, LOC100287015, MCPH1, ANGPT2, MCPH1-AS1, MIR8055, AGPAT5, MIR4659A, MIR4659B, XKR5, GS1-24F4.2, DEFB1, DEFA6, DEFA4, DEFA8P, DEFA9P, DEFA10P, DEFA1, DEFA1B, DEFT1P2, DEFT1P, DEFA3, DEFA11P, DEFA5, LINC00965, FAM66B, DEFB109P1B, USP17L1, USP17L4, ZNF705G, DEFB4B, DEFB103B, DEFB103A, SPAG11B, DEFB104A, DEFB104B, DEFB106B, DEFB106A, DEFB105B, DEFB105A, DEFB107B, DEFB107A, PRR23D1, PRR23D2, FAM90A7P, FAM90A10P, SPAG11A, DEFB4A, ZNF705B, FAM66E, USP17L8, USP17L3, MIR548I3, FAM86B3P, SGK223, CLDN23, MFHAS1, ERI1, MIR4660, PPP1R3B, LOC101929128, LOC157273, TNKS, MIR597, LINC00599, MIR124-1, MSRA, LINCR-0001, PRSS55, RP1L1, MIR4286, C8orf74, SOX7, PINX1, MIR1322, LOC101929229, XKR6, MIR598, LOC101929269, MTMR9, SLC35G5, TDH, FAM167A-AS1, FAM167A, BLK, LINC00208, GATA4, SNORA99, C8orf49, NEIL2, FDFT1, CTSB, DEFB136, DEFB135, DEFB134, LOC100133267, DEFB130, ZNF705D, FAM66D, LOC392196, USP17L7, USP17L2, FAM90A2P, FAM86B1, FAM66A, LOC649352, DEFB109P1, FAM90A25P, FAM86B2, LOC100506990, LOC729732, MIR5692A1, MIR5692A2, LONRF1, MIR3926-1, MIR3926-2, LOC340357, LINC00681, KIAA1456, DLC1, C8orf48, LOC102725080, SGCZ, MIR383, TUSC3, MSR1, FGF20, MICU3, ZDHHC2, CNOT7, VPS37A, MTMR7, SLC7A2, PDGFRL, MTUS1, MIR548V, FGL1, PCM1, ASAH1, LOC101929066, NAT1, NAT2, PSD3, LOC100128993, SH2D4A, CSGALNACT1, INTS10, LPL, SLC18A1, ATP6V1B2, LZTS1, LZTS1-AS1, LOC102467222, LOC286114, LOC101929172, GFRA2, DOK2, XPO7, NPM2, FGF17, DMTN, FAM160B2, NUDT18, HR, REEP4, LGI3, SFTPC, BMP1, PHYHIP, MIR320A, POLR3D, LOC100507071, PIWIL2, SLC39A14, PPP3CC, SORBS3, PDLIM2, C8orf58, CCAR2, BIN3, BIN3-IT1, EGR3, PEBP4, LOC101929237, RHOBTB2, TNFRSF10B, LOC286059, LOC254896, TNFRSF10C, TNFRSF10D, TNFRSF10A, LOC389641, CHMP7, R3HCC1, LOXL2, LOC100507156, ENTPD4, SLC25A37, NKX3-1, NKX2-6, STC1, ADAM28, LOC101929294, ADAMDEC1, ADAM7, LOC101929315, NEFM, NEFL, MIR6841, DOCK5, MIR6876, GNRH1, KCTD9, CDCA2, EBF2, PPP2R2A, BNIP3L, PNMA2, DPYSL2, ADRA1A, STMN4, TRIM35, PTK2B, MIR6842, CHRNA2, EPHX2, CLU, MIR6843, SCARA3, MIR3622B, MIR3622A, CCDC25, ESCO2, PBK, SCARA5, MIR4287, NUGGC, ELP3, PNOC, ZNF395, FBXO16, FZD3, MIR4288, MIR7641-2, EXTL3-AS1, EXTL3, INTS9, HMBOX1, KIF13B, DUSP4 | arr[GRCh37] 8p23.3p12(172416_29515831)x1 |
| 1  LM | Loss | 14 | q21.2 | q24.2 | 25,075 | LINC00871, RPL10L, MDGA2, MIR548Y, LINC00648, RPS29, LRR1, RPL36AL, MGAT2, DNAAF2, POLE2, KLHDC1, KLHDC2, NEMF, ARF6, MIR6076, LINC01588, LINC01599, VCPKMT, SOS2, L2HGDH, MIR4504, ATP5S, CDKL1, MAP4K5, ATL1, SAV1, NIN, LOC105370489, ABHD12B, PYGL, TRIM9, TMX1, LINC00640, FRMD6-AS2, FRMD6, FRMD6-AS1, GNG2, LOC102723604, C14orf166, NID2, PTGDR, PTGER2, TXNDC16, GPR137C, ERO1A, PSMC6, STYX, GNPNAT1, FERMT2, DDHD1, LOC101927620, MIR5580, BMP4, CDKN3, CNIH1, GMFB, CGRRF1, SAMD4A, GCH1, MIR4308, WDHD1, SOCS4, MAPK1IP1L, LGALS3, DLGAP5, FBXO34, ATG14, TBPL2, KTN1-AS1, KTN1, RPL13AP3, LINC00520, PELI2, LOC101927690, TMEM260, OTX2, OTX2-AS1, EXOC5, AP5M1, NAA30, C14orf105, SLC35F4, C14orf37, ACTR10, PSMA3, PSMA3-AS1, ARID4A, TOMM20L, TIMM9, KIAA0586, DACT1, LINC01500, DAAM1, GPR135, L3HYPDH, JKAMP, CCDC175, RTN1, MIR5586, LRRC9, PCNX4, DHRS7, PPM1A, C14orf39, SIX6, SALRNA1, SIX1, SIX4, MNAT1, TRMT5, SLC38A6, TMEM30B, PRKCH, LOC101927780, FLJ22447, HIF1A-AS1, HIF1A, HIF1A-AS2, SNAPC1, SYT16, LINC00643, LINC00644, KCNH5, RHOJ, GPHB5, PPP2R5E, WDR89, SGPP1, SYNE2, MIR548H1, ESR2, TEX21P, MTHFD1, ZBTB25, AKAP5, ZBTB1, LOC102723809, HSPA2, PPP1R36, PLEKHG3, SPTB, MIR7855, CHURC1, CHURC1-FNTB, GPX2, RAB15, FNTB, MAX, MIR4706, LOC100506321, LOC100128233, MIR4708, FUT8, FUT8-AS1, MIR625, LINC00238, GPHN, FAM71D, MPP5, ATP6V1D, EIF2S1, PLEK2, MIR5694, TMEM229B, PLEKHH1, PIGH, ARG2, VTI1B, RDH11, RDH12, ZFYVE26, RAD51B, LOC100996664, ZFP36L1, ACTN1, ACTN1-AS1, DCAF5, EXD2, GALNT16, ERH, SLC39A9, PLEKHD1, CCDC177, SUSD6, LOC100289511, SRSF5, SLC10A1, SMOC1, SLC8A3, LOC646548, ADAM21P1, COX16, SYNJ2BP-COX16, SYNJ2BP, ADAM21, ADAM20P1, ADAM20, MED6 | arr[GRCh37] 14q21.2q24.2(45987004_71061844)x1 |
| 1  LM | Loss | 17 | p13.3 | p13.1 | 7,223 | VPS53, FAM57A, GEMIN4, DBIL5P, GLOD4, MRM3, NXN, LOC101927727, TIMM22, ABR, MIR3183, BHLHA9, TUSC5, YWHAE, CRK, MYO1C, INPP5K, PITPNA-AS1, PITPNA, SLC43A2, SCARF1, RILP, PRPF8, TLCD2, MIR22HG, MIR22, WDR81, SERPINF2, SERPINF1, SMYD4, RPA1, RTN4RL1, LOC105371485, DPH1, OVCA2, MIR132, MIR212, HIC1, SMG6, LOC101927839, SRR, TSR1, SNORD91B, SNORD91A, SGSM2, MNT, LOC284009, METTL16, PAFAH1B1, CLUH, MIR6776, LOC105371592, MIR1253, RAP1GAP2, LOC101927911, OR1D5, OR1D2, OR1G1, OR1A2, OR1A1, OR1D4, OR3A2, OR3A1, OR3A4P, OR1E1, OR3A3, OR1E2, SPATA22, ASPA, TRPV3, TRPV1, SHPK, CTNS, TAX1BP3, P2RX5-TAX1BP3, EMC6, P2RX5, ITGAE, GSG2, NCBP3, CAMKK1, P2RX1, ATP2A3, ZZEF1, CYB5D2, ANKFY1, UBE2G1, LOC103021295, SPNS3, SPNS2, MYBBP1A, GGT6, SMTNL2, ALOX15, PELP1, LOC101559451, ARRB2, MED11, CXCL16, ZMYND15, TM4SF5, VMO1, GLTPD2, PSMB6, PLD2, MINK1, CHRNE, C17orf107, GP1BA, SLC25A11, RNF167, PFN1, ENO3, SPAG7, CAMTA2, MIR6864, MIR6865, INCA1, KIF1C, LOC102724009, SLC52A1, ZFP3, ZNF232, LOC101928000, USP6, ZNF594, LOC100130950, SCIMP, RABEP1, NUP88, RPAIN, C1QBP, DHX33, LOC105371506, DERL2, MIS12, LOC728392, NLRP1, LOC339166, WSCD1, AIPL1, FAM64A, PITPNM3, KIAA0753, TXNDC17, MED31, C17orf100, MIR4520-1, MIR4520-2, ALOX15P1, SLC13A5, XAF1, FBXO39, TEKT1, ALOX12P2, ALOX12-AS1, ALOX12, RNASEK, RNASEK-C17orf49, C17orf49, MIR497HG, MIR195, MIR497, BCL6B, SLC16A13, SLC16A11, CLEC10A, ASGR2, ASGR1, DLG4, ACADVL, MIR324, DVL2, PHF23, GABARAP, CTDNEP1, ELP5, CLDN7, SLC2A4, YBX2, EIF5A, GPS2, NEURL4, ACAP1, KCTD11, TMEM95, TNK1, PLSCR3, TMEM256-PLSCR3, TMEM256, NLGN2, SPEM1, C17orf74, TMEM102, FGF11, CHRNB1, ZBTB4, SLC35G6, POLR2A, TNFSF12, TNFSF12-TNFSF13, TNFSF13, SENP3, SENP3-EIF4A1, EIF4A1, SNORA48, SNORD10, SNORA67, CD68, LOC100996842, MPDU1, SOX15, FXR2, SHBG, SAT2, ATP1B2, TP53, WRAP53, EFNB3, DNAH2 | arr[GRCh37] 17p13.3p13.1(400958_7624100)x1-2 |
| 1  LM | Loss | 18 | p11.32 | q23 | 77,995 | LOC102723376, ROCK1P1, MIR8078, USP14, THOC1, COLEC12, LOC105376854, CETN1, CLUL1, TYMSOS, TYMS, ENOSF1, YES1, ADCYAP1, LINC00470, METTL4, NDC80, CBX3P2, SMCHD1, EMILIN2, LPIN2, LOC727896, MYOM1, MYL12A, LOC104968399, MYL12B, TGIF1, GAPLINC, DLGAP1, DLGAP1-AS1, DLGAP1-AS2, DLGAP1-AS3, MIR6718, DLGAP1-AS4, DLGAP1-AS5, AKAIN1, LINC00526, LINC00667, ZBTB14, EPB41L3, MIR3976HG, MIR3976, TMEM200C, L3MBTL4, L3MBTL4-AS1, MIR4317, LINC01387, LOC101927168, ARHGAP28, LINC00668, LAMA1, LOC101927188, LRRC30, PTPRM, LOC100192426, RAB12, GACAT2, MTCL1, NDUFV2, NDUFV2-AS1, ANKRD12, TWSG1, RALBP1, PPP4R1, PPP4R1-AS1, RAB31, TXNDC2, VAPA, LINC01254, APCDD1, NAPG, LOC101927410, PIEZO2, MIR6788, LINC01255, SLC35G4, MIR7153, GNAL, CHMP1B, MPPE1, IMPA2, ANKRD62, C18orf61, CIDEA, TUBB6, AFG3L2, PRELID3A, LOC105371998, SPIRE1, PSMG2, CEP76, LOC100996324, PTPN2, SEH1L, CEP192, LDLRAD4, LDLRAD4-AS1, MIR5190, MIR4526, FAM210A, RNMT, MC5R, MC2R, ZNF519, ANKRD20A5P, CYP4F35P, CXADRP3, POTEC, ANKRD30B, MIR3156-2, LINC01443, LINC01444, LOC644669, ROCK1, GREB1L, ESCO1, SNRPD1, ABHD3, MIR320C1, MIB1, MIR133A1HG, MIR133A1, MIR1-2, GATA6-AS1, GATA6, CTAGE1, LOC101927571, RBBP8, MIR4741, CABLES1, TMEM241, RIOK3, C18orf8, NPC1, ANKRD29, LAMA3, TTC39C, TTC39C-AS1, CABYR, OSBPL1A, MIR320C2, IMPACT, HRH4, LOC729950, LOC105372028, ZNF521, SS18, PSMA8, TAF4B, LINC01543, KCTD1, MIR8057, PCAT18, AQP4, AQP4-AS1, CHST9, LOC105372038, CDH2, MIR302F, DSC3, DSC2, DSCAS, DSC1, DSG1, DSG1-AS1, DSG4, DSG3, DSG2, DSG2-AS1, TTR, B4GALT6, SLC25A52, TRAPPC8, RNF125, RNF138, MEP1B, GAREM1, WBP11P1, KLHL14, CCDC178, ASXL3, NOL4, DTNA, MAPRE2, ZNF397, ZSCAN30, ZNF271P, ZNF24, ZNF396, INO80C, MIR3975, GALNT1, MIR187, MIR3929, C18orf21, RPRD1A, SLC39A6, ELP2, LOC101927809, MOCOS, FHOD3, LOC105372071, TPGS2, KIAA1328, LOC105372069, CELF4, LOC105372068, SNORA111, MIR4318, MIR924HG, MIR924, MIR5583-2, MIR5583-1, LINC01477, KC6, PIK3C3, LINC00907, RIT2, SYT4, LINC01478, LOC105667213, SETBP1, MIR4319, SLC14A2, SLC14A2-AS1, SLC14A1, SIGLEC15, EPG5, PSTPIP2, ATP5A1, HAUS1, C18orf25, RNF165, LOXHD1, ST8SIA5, PIAS2, KATNAL2, TCEB3CL, TCEB3CL2, TCEB3C, TCEB3B, HDHD2, IER3IP1, SKOR2, MIR4527, SMAD2, ZBTB7C, CTIF, MIR4743, SMAD7, DYM, MIR4744, C18orf32, RPL17-C18orf32, MIR1539, RPL17, SNORD58C, SNORD58A, SNORD58B, LIPG, ACAA2, SCARNA17, SNHG22, MYO5B, MIR4320, CFAP53, MBD1, CXXC1, SKA1, MAPK4, MRO, ME2, ELAC1, SMAD4, MEX3C, LINC01630, DCC, MIR4528, LOC102724651, LOC101928167, MBD2, SNORA37, POLI, STARD6, C18orf54, DYNAP, RAB27B, CCDC68, LOC101927229, TCF4, TCF4-AS1, MIR4529, LINC01416, LINC01539, TXNL1, WDR7, LINC-ROR, BOD1L2, ST8SIA3, ONECUT2, FECH, NARS, LOC100505549, ATP8B1, NEDD4L, MIR122, MIR3591, ALPK2, SNORA108, LOC101927322, MALT1, ZNF532, OACYLP, SEC11C, GRP, RAX, CPLX4, LMAN1, CCBE1, PMAIP1, MC4R, CDH20, LINC01544, RNF152, PIGN, KIAA1468, TNFRSF11A, ZCCHC2, PHLPP1, BCL2, KDSR, VPS4B, SERPINB5, SERPINB12, SERPINB13, SERPINB4, SERPINB3, SERPINB11, SERPINB7, SERPINB2, SERPINB10, HMSD, SERPINB8, LINC00305, LOC284294, LINC01538, CDH7, CDH19, MIR5011, DSEL, LOC643542, TMX3, CCDC102B, DOK6, LOC105372179, CD226, RTTN, SOCS6, LOC101927481, LOC101060542, GTSCR1, LINC01541, LOC102724913, CBLN2, NETO1, MIR548AV, LOC100505797, LOC400655, LOC100505817, FBXO15, TIMM21, CYB5A, C18orf63, LOC101927606, FAM69C, CNDP2, CNDP1, LINC00909, ZNF407, ZADH2, TSHZ1, SMIM21, LOC100505853, LOC339298, ZNF516, LOC101927989, C18orf65, LINC00908, LINC00683, LOC101927651, LOC400661, LOC100131655, ZNF236, MBP, GALR1, LINC01029, SALL3, ATP9B, NFATC1, LOC284241, CTDP1, KCNG2, PQLC1, HSBP1L1, TXNL4A, RBFA, RBFADN, ADNP2, PARD6G-AS1, PARD6G | arr[GRCh37] 18p11.32q23(12841_78007784)x1 |
| 1  BM | Gain | 3 | p26.3 | p22.1 | 41,898 | LOC102723448, CHL1, CHL1-AS1, LINC01266, CNTN6, CNTN4, CNTN4-AS2, CNTN4-AS1, IL5RA, TRNT1, CRBN, LRRN1, SETMAR, SUMF1, ITPR1-AS1, ITPR1, EGOT, BHLHE40-AS1, BHLHE40, ARL8B, EDEM1, MIR4790, GRM7-AS3, GRM7, GRM7-AS2, GRM7-AS1, LOC101927394, LMCD1-AS1, LMCD1, LINC00312, SSUH2, CAV3, OXTR, RAD18, SRGAP3, LOC101927416, SRGAP3-AS3, THUMPD3, THUMPD3-AS1, SETD5, LHFPL4, MTMR14, CPNE9, BRPF1, OGG1, CAMK1, TADA3, ARPC4, ARPC4-TTLL3, TTLL3, RPUSD3, CIDEC, JAGN1, IL17RE, IL17RC, CRELD1, PRRT3, PRRT3-AS1, EMC3, EMC3-AS1, LOC401052, CIDECP, FANCD2, FANCD2OS, BRK1, VHL, IRAK2, TATDN2, GHRLOS, LINC00852, GHRL, SEC13, ATP2B2, MIR378B, MIR885, ATP2B2-IT2, LINC00606, SLC6A11, SLC6A1, SLC6A1-AS1, HRH1, ATG7, VGLL4, TAMM41, SYN2, TIMP4, PPARG, TSEN2, MKRN2OS, MKRN2, RAF1, TMEM40, CAND2, RPL32, SNORA7A, LOC105376955, IQSEC1, NUP210, HDAC11-AS1, HDAC11, FBLN2, SNORA93, LINC00620, WNT7A, FGD5P1, TPRXL, CHCHD4, TMEM43, XPC, LSM3, LINC01267, SLC6A6, GRIP2, CCDC174, C3orf20, LOC152274, FGD5, FGD5-AS1, NR2C2, MRPS25, RBSN, COL6A4P1, CAPN7, SH3BP5-AS1, SH3BP5, METTL6, EAF1, COLQ, MIR4270, HACL1, BTD, ANKRD28, MIR3134, MIR563, GALNT15, DPH3, OXNAD1, RFTN1, LINC00690, DAZL, PLCL2, MIR3714, TBC1D5, LOC105376975, LOC339862, SATB1, SATB1-AS1, KCNH8, MIR4791, EFHB, RAB5A, PP2D1, KAT2B, MIR3135A, SGO1, SGO1-AS1, LOC101927829, VENTXP7, ZNF385D, ZNF385D-AS1, ZNF385D-AS2, UBE2E2-AS1, UBE2E2, MIR548AC, UBE2E1-AS1, UBE2E1, NKIRAS1, RPL15, NR1D2, LINC00691, THRB, LOC101927854, THRB-AS1, MIR4792, RARB, LOC105376997, TOP2B, MIR4442, NGLY1, OXSM, LINC00692, LRRC3B, NEK10, SLC4A7, EOMES, LOC100996624, CMC1, AZI2, ZCWPW2, LINC00693, RBMS3-AS3, RBMS3, RBMS3-AS1, TGFBR2, GADL1, MIR466, STT3B, OSBPL10, OSBPL10-AS1, ZNF860, GPD1L, CMTM8, CMTM7, CMTM6, MIR548AY, DYNC1LI1, CNOT10, TRIM71, CCR4, GLB1, TMPPE, CRTAP, SUSD5, FBXL2, UBP1, CLASP2, PDCD6IP, LOC101928135, ARPP21, MIR128-2, STAC, DCLK3, TRANK1, EPM2AIP1, MLH1, LRRFIP2, GOLGA4, C3orf35, ITGA9, ITGA9-AS1, CTDSPL, MIR26A1, VILL, PLCD1, DLEC1, ACAA1, MYD88, OXSR1, SLC22A13, SLC22A14, XYLB, ACVR2B-AS1, ACVR2B, EXOG, SCN5A, SCN10A, SCN11A, WDR48, GORASP1, TTC21A, MIR6822, CSRNP1, XIRP1, CX3CR1, CCR8, SLC25A38, RPSA, SNORA6, SNORA62, MOBP, MYRIP, EIF1B-AS1, EIF1B, ENTPD3, ENTPD3-AS1, RPL14, ZNF619, ZNF620, ZNF621, CTNNB1, ULK4 | arr[GRCh37] 3p26.3p22.1(63410_41961168)x3 |
| 1  BM | Gain | 4 | p16.3 | q35.2 | 190,846 | ZNF595, ZNF718, ZNF876P, ZNF732, ZNF141, MIR571, ABCA11P, ZNF721, PIGG, PDE6B, ATP5I, MYL5, MFSD7, PCGF3, LOC100129917, CPLX1, GAK, TMEM175, DGKQ, SLC26A1, IDUA, FGFRL1, RNF212, LOC105374344, TMED11P, SPON2, LOC100130872, CTBP1-AS, CTBP1, CTBP1-AS2, MAEA, UVSSA, CRIPAK, NKX1-1, FAM53A, SLBP, TMEM129, TACC3, FGFR3, LETM1, WHSC1, SCARNA22, NELFA, MIR943, C4orf48, NAT8L, POLN, HAUS3, MXD4, MIR4800, ZFYVE28, CFAP99, RNF4, FAM193A, TNIP2, SH3BP2, ADD1, MFSD10, NOP14-AS1, NOP14, GRK4, HTT-AS, HTT, MSANTD1, RGS12, HGFAC, DOK7, LRPAP1, LINC00955, LOC100133461, ADRA2C, FAM86EP, OTOP1, TMEM128, LYAR, ZBTB49, NSG1, STX18, STX18-IT1, STX18-AS1, LOC101928279, LINC01396, MSX1, LOC101928306, CYTL1, STK32B, LINC01587, EVC2, EVC, CRMP1, MIR378D1, JAKMIP1, LOC285484, WFS1, PPP2R2C, MAN2B2, MRFAP1, LOC105374366, LOC93622, S100P, MRFAP1L1, BLOC1S4, KIAA0232, TBC1D14, LOC100129931, CCDC96, TADA2B, GRPEL1, FLJ36777, SORCS2, MIR4798, PSAPL1, MIR4274, AFAP1-AS1, AFAP1, LOC389199, ABLIM2, MIR95, SH3TC1, HTRA3, ACOX3, TRMT44, GPR78, CPZ, HMX1, LOC650293, USP17L10, USP17L20, USP17L18, USP17L11, USP17L12, USP17L21, USP17L13, USP17L15, USP17L17, USP17L19, USP17L22, USP17L25, USP17L30, USP17L26, USP17L24, USP17L5, USP17L28, USP17L27, USP17L29, USP17L9P, USP17L6P, DEFB131, MIR548I2, DRD5, SLC2A9, WDR1, MIR3138, ZNF518B, CLNK, MIR572, HS3ST1, LOC101929019, RAB28, LINC01097, NKX3-2, LINC01096, BOD1L1, MIR5091, LINC01182, LINC01085, LINC00504, CPEB2-AS1, CPEB2, LOC101929095, C1QTNF7, CC2D2A, FBXL5, FAM200B, BST1, CD38, FGFBP1, FGFBP2, PROM1, TAPT1, TAPT1-AS1, LDB2, LOC101929123, QDPR, CLRN2, LAP3, MED28, FAM184B, DCAF16, NCAPG, LCORL, SLIT2, SLIT2-IT1, MIR218-1, PACRGL, KCNIP4, MIR7978, LOC105374516, KCNIP4-IT1, LOC100505912, ADGRA3, GBA3, PPARGC1A, MIR573, DHX15, SOD3, CCDC149, LGI2, SEPSECS, SEPSECS-AS1, PI4K2B, ZCCHC4, ANAPC4, LOC101929161, SLC34A2, SEL1L3, SMIM20, RBPJ, CCKAR, TBC1D19, LOC105374546, STIM2, LOC101929199, MIR4275, PCDH7, LOC102723778, LOC102723828, LOC105377651, LOC101927363, LOC101928622, ARAP2, LOC439933, DTHD1, MIR1255B1, LOC100508631, MIR4801, NWD2, C4orf19, RELL1, PGM2, TBC1D1, PTTG2, LINC01258, KLF3-AS1, KLF3, TLR10, TLR1, TLR6, FAM114A1, MIR574, TMEM156, KLHL5, WDR19, RFC1, KLB, MIR5591, RPL9, LIAS, LOC401127, UGDH, UGDH-AS1, SMIM14, UBE2K, PDS5A, LOC344967, N4BP2, RHOH, LOC101060498, CHRNA9, RBM47, MIR4802, NSUN7, APBB2, UCHL1-AS1, UCHL1, LIMCH1, PHOX2B, LINC00682, TMEM33, DCAF4L1, SLC30A9, BEND4, LOC105374428, SHISA3, ATP8A1, GRXCR1, LVCAT1, KCTD8, YIPF7, GUF1, GNPDA2, GABRG1, GABRA2, COX7B2, GABRA4, GABRB1, COMMD8, ATP10D, CORIN, MIR8053, LOC101927179, NFXL1, LOC101927157, CNGA1, NIPAL1, TXK, TEC, SLAIN2, SLC10A4, ZAR1, FRYL, OCIAD1, OCIAD2, CWH43, DCUN1D4, LRRC66, SGCB, SPATA18, USP46, USP46-AS1, DANCR, MIR4449, SNORA26, ERVMER34-1, LINC01618, RASL11B, SCFD2, FIP1L1, LNX1, LNX1-AS1, LNX1-AS2, LOC100506444, RPL21P44, CHIC2, GSX2, PDGFRA, LOC339978, KIT, KDR, SRD5A3, SRD5A3-AS1, TMEM165, CLOCK, PDCL2, NMU, LOC644145, EXOC1, CEP135, MIR7641-2, KIAA1211, AASDH, PPAT, PAICS, SRP72, ARL9, THEGL, HOPX, SPINK2, REST, NOA1, POLR2B, IGFBP7, IGFBP7-AS1, LOC101928851, LOC105377671, LOC105377247, LOC105377245, MIR548AG1, ADGRL3, ADGRL3-AS1, TECRL, LOC401134, EPHA5, EPHA5-AS1, MIR1269A, LOC101927237, CENPC, STAP1, UBA6, UBA6-AS1, GNRHR, TMPRSS11D, TMPRSS11A, TMPRSS11GP, TMPRSS11F, LOC550113, SYT14P1, FTLP10, TMPRSS11BNL, TMPRSS11B, YTHDC1, TMPRSS11E, UGT2B17, UGT2B15, UGT2B10, UGT2A3, UGT2B7, LOC105377267, UGT2B11, UGT2B28, UGT2B4, UGT2A2, UGT2A1, SULT1B1, SULT1E1, CSN1S1, CSN2, STATH, HTN3, HTN1, CSN1S2AP, CSN1S2BP, PRR27, ODAM, FDCSP, CSN3, CABS1, SMR3A, SMR3B, OPRPN, MUC7, AMTN, AMBN, ENAM, JCHAIN, UTP3, RUFY3, GRSF1, MOB1B, DCK, SLC4A4, GC, NPFFR2, ADAMTS3, COX18, ANKRD17, ALB, AFP, AFM, LOC728040, RASSF6, CXCL8, CXCL6, PF4V1, CXCL1, PF4, PPBP, CXCL5, CXCL3, PPBPP2, CXCL2, MTHFD2L, EPGN, EREG, AREG, BTC, PARM1, LOC100507388, LOC441025, RCHY1, THAP6, C4orf26, CDKL2, G3BP2, USO1, PPEF2, NAAA, SDAD1, LOC101928809, CXCL9, ART3, CXCL10, CXCL11, NUP54, SCARB2, FAM47E, FAM47E-STBD1, STBD1, CCDC158, SHROOM3, MIR4450, MIR548AH, SOWAHB, SEPT11, CCNI, CCNG2, CXCL13, CNOT6L, MRPL1, FRAS1, ANXA3, LINC01094, BMP2K, MIR5096, PAQR3, LINC01088, NAA11, GK2, LINC00989, PCAT4, ANTXR2, PRDM8, FGF5, C4orf22, BMP3, PRKG2, LOC101928942, RASGEF1B, HNRNPD, HNRNPDL, ENOPH1, TMEM150C, LINC00575, SCD5, MIR575, SEC31A, THAP9-AS1, SNORD143, SNORD144, THAP9, LIN54, COPS4, PLAC8, COQ2, HPSE, HELQ, MRPS18C, FAM175A, GPAT3, LOC101928978, NKX6-1, CDS1, WDFY3, WDFY3-AS2, ARHGAP24, MIR4451, MAPK10, LOC101929064, MIR4452, PTPN13, SLC10A6, C4orf36, LOC100506746, AFF1, KLHL8, MIR5705, HSD17B13, HSD17B11, NUDT9, SPARCL1, DSPP, DMP1, IBSP, MEPE, SPP1, PKD2, ABCG2, PPM1K, LOC105369192, HERC6, HERC5, PIGY, PYURF, LOC101929134, HERC3, NAP1L5, FAM13A-AS1, FAM13A, TIGD2, GPRIN3, SNCA, SNCA-AS1, MMRN1, CCSER1, LOC101929194, GRID2, ATOH1, LOC101929210, SMARCAD1, HPGDS, PDLIM5, BMPR1B-AS1, BMPR1B, UNC5C, PDHA2, STPG2-AS1, STPG2, RAP1GDS1, TSPAN5, EIF4E, METAP1, MIR3684, ADH5, LOC100507053, ADH4, PCNAP1, ADH6, ADH1A, ADH1B, ADH1C, ADH7, C4orf17, TRMT10A, MTTP, DAPP1, LAMTOR3, DNAJB14, H2AFZ, LOC256880, DDIT4L, LOC101929353, SNORA101A, EMCN, LINC01216, PPP3CA, MIR8066, MIR1255A, FLJ20021, BANK1, SLC39A8, LOC105377621, NFKB1, MANBA, LOC102723704, UBE2D3, LOC105377348, CISD2, SLC9B1, SLC9B2, BDH2, CENPE, LOC101929448, TACR3, CXXC4, LOC101929468, TET2, TET2-AS1, PPA2, ARHGEF38, ARHGEF38-IT1, INTS12, GSTCD, LOC101929529, NPNT, LOC101929577, TBCK, AIMP1, GIMD1, DKK2, PAPSS1, SGMS2, LOC101929595, CYP2U1, HADH, LEF1, LEF1-AS1, RPL34-AS1, LOC101929621, RPL34, OSTC, ETNPPL, COL25A1, SEC24B-AS1, SEC24B, MCUB, CASP6, PLA2G12A, CFI, GAR1, RRH, LRIT3, EGF, ELOVL6, ENPEP, PITX2, C4orf32, AP1AR, TIFA, ALPK1, NEUROG2, ZGRF1, LARP7, MIR367, MIR302D, MIR302A, MIR302C, MIR302B, ANK2, MIR1243, MIR8082, CAMK2D, ARSJ, UGT8, MIR577, NDST4, MIR1973, TRAM1L1, LINC01378, NDST3, SNHG8, SNORA24, PRSS12, CEP170P1, LOC729218, LOC101929741, METTL14, SEC24D, SYNPO2, MYOZ2, LOC101929762, USP53, C4orf3, FABP2, LINC01061, GTF2IP12, LOC645513, PDE5A, LINC01365, LOC100996694, MAD2L1, PRDM5, NDNF, TNIP3, QRFPR, ANXA5, TMEM155, PP12613, EXOSC9, CCNA2, BBS7, TRPC3, KIAA1109, ADAD1, IL2, IL21, IL21-AS1, CETN4P, BBS12, FGF2, NUDT6, SPATA5, SPRY1, LINC01091, LOC101927087, ANKRD50, FAT4, MIR2054, INTU, SLC25A31, HSPA4L, PLK4, MFSD8, ABHD18, LARP1B, PGRMC2, LOC100507487, JADE1, SCLT1, C4orf33, LOC101927282, LOC101927305, LINC01256, LOC101927359, PCDH10, PABPC4L, LINC00613, LOC729307, PCDH18, LINC00616, SLC7A11-AS1, SLC7A11, LINC00499, LOC105377448, NOCT, ELF2, MGARP, NDUFC1, NAA15, RAB33B, SETD7, MGST2, MAML3, SCOC, SCOC-AS1, CLGN, MGAT4D, ELMOD2, UCP1, TBC1D9, TNRC18P1, RNF150, ZNF330, LOC100507639, IL15, INPP4B, LOC105377623, USP38, GAB1, MIR3139, SMARCA5, SMARCA5-AS1, GUSBP5, FREM3, LOC105377458, GYPE, LOC101927636, GYPB, GYPA, HHIP-AS1, HHIP, ANAPC10, ABCE1, OTUD4, SMAD1, SMAD1-AS2, SMAD1-AS1, MMAA, C4orf51, ZNF827, LINC01095, LSM6, SLC10A7, MIR7849, POU4F2, TTC29, EDNRA, TMEM184C, PRMT9, ARHGAP10, MIR4799, NR3C2, LOC105377480, LOC101927849, DCLK2, LRBA, MAB21L2, RPS3A, SNORD73A, SH3D19, PRSS48, FAM160A1, GATB, LOC100996286, FBXW7, DEAR, MIR3140, DKFZP434I0714, MIR4453, TMEM154, TIGD4, ARFIP1, LOC729870, FHDC1, TRIM2, ANXA2P1, MND1, KIAA0922, LOC100419170, TLR2, RNF175, SFRP2, DCHS2, PLRG1, FGB, FGA, FGG, LRAT, RBM46, NPY2R, MAP9, LOC102724776, GUCY1A3, GUCY1B3, ASIC5, TDO2, CTSO, PDGFC, GLRB, GRIA2, LOC340017, FAM198B, TMEM144, RXFP1, C4orf46, ETFDH, PPID, FNIP2, C4orf45, MIR3688-1, MIR3688-2, RAPGEF2, FSTL5, LOC101928052, MIR4454, NAF1, NPY1R, NPY5R, TKTL2, TMA16, MARCH1, ANP32C, LINC01207, APELA, TRIM61, FAM218A, TRIM60, TMEM192, KLHL2, GK3P, MSMO1, CPE, MIR578, LINC01179, LOC101928131, TLL1, SPOCK3, ANXA10, DDX60, DDX60L, PALLD, CBR4, SH3RF1, NEK1, CLCN3, HPF1, LOC100506085, MFAP3L, AADAT, LINC01612, LOC100506107, LOC100506122, MIR6082, LOC441052, GALNTL6, LOC101928314, LOC101930370, GALNT7, HMGB2, SAP30, SCRG1, HAND2, HAND2-AS1, LOC101928509, FBXO8, CEP44, MIR4276, HPGD, GLRA3, LOC101928551, ADAM29, GPM6A, LOC101928590, WDR17, SPATA4, ASB5, SPCS3, VEGFC, NEIL3, AGA, LINC01098, LINC01099, LINC00290, LOC90768, MIR1305, TENM3, DCTD, FAM92A1P2, WWC2-AS2, WWC2, WWC2-AS1, CLDN22, CLDN24, CDKN2AIP, LOC389247, ING2, RWDD4, TRAPPC11, STOX2, ENPP6, LOC728175, LOC102723766, IRF2, LVCAT8, CASP3, PRIMPOL, CENPU, ACSL1, SLED1, MIR3945HG, MIR3945, LINC01093, MIR4455, HELT, SLC25A4, CFAP97, SNX25, LRP2BP, ANKRD37, UFSP2, C4orf47, CCDC110, LOC105377590, PDLIM3, SORBS2, TLR3, FAM149A, FLJ38576, CYP4V2, KLKB1, F11, F11-AS1, MTNR1A, FAT1, LOC339975, LOC100506272, ZFP42, TRIML2, TRIML1, LINC01060, LINC01262, LOC105379514, LINC01596, FRG1 | arr[GRCh37] 4p16.3q35.2(69403_190915650)x3 |
| 1  BM | Gain | 5 | p15.33 | q11.2 | 54,070 | PLEKHG4B, LRRC14B, CCDC127, SDHA, HRAT5, PDCD6, AHRR, EXOC3-AS1, EXOC3, PP7080, SLC9A3, LOC100288152, MIR4456, LOC100996325, CEP72, TPPP, ZDHHC11, BRD9, TRIP13, LOC100506688, NKD2, SLC12A7, MIR4635, CTD-3080P12.3, SLC6A19, SLC6A18, TERT, MIR4457, CLPTM1L, LINC01511, SLC6A3, LPCAT1, MIR6075, SDHAP3, LOC728613, MIR4277, MRPL36, NDUFS6, LOC101929034, IRX4, CTD-2194D22.4, LOC100506858, IRX2, C5orf38, LOC105374620, LINC01377, LINC01019, LINC01017, IRX1, LOC101929153, LINC01020, LOC105374631, CTD-2297D10.2, ADAMTS16, ICE1, FLJ33360, MED10, UBE2QL1, LINC01018, NSUN2, SRD5A1, LOC100505625, PAPD7, MIR4278, MIR4454, LOC442132, LOC101929261, ADCY2, C5orf49, FASTKD3, MTRR, LOC729506, MIR4458HG, MIR4458, LOC101929284, SEMA5A, MIR4636, CTD-2201E9.1, SNHG18, SNORD123, TAS2R1, LOC285692, FAM173B, CCT5, CMBL, MARCH6, ROPN1L-AS1, ROPN1L, MIR6131, LOC101929412, LOC389273, ANKRD33B, DAP, CTNND2, LINC01194, DNAH5, TRIO, FAM105A, SNORD141B, SNORD141A, OTULIN, ANKH, LOC100130744, MIR4637, LOC101929454, FBXL7, CTD-2350J17.1, MIR887, MARCH11, LOC101929505, ZNF622, FAM134B, LOC101929524, MYO10, LOC285696, BASP1, LOC401177, LOC101929544, LOC102723526, LOC646241, CDH18, GUSBP1, CDH12, SNORA105A, SNORA105B, PMCHL1, PRDM9, C5orf17, CDH10, LOC340107, LOC105374693, CDH9, LINC01021, LOC105374698, LSP1P3, LOC101929645, LOC101929660, LOC101929681, LOC105374704, CDH6, DROSHA, C5orf22, PDZD2, MIR4279, GOLPH3, MTMR12, ZFR, MIR579, SUB1, NPR3, LOC340113, TARS, ADAMTS12, RXFP3, SLC45A2, AMACR, C1QTNF3-AMACR, C1QTNF3, RAI14, MIR7641-2, TTC23L, RAD1, BRIX1, DNAJC21, AGXT2, PRLR, SPEF2, IL7R, CAPSL, LOC100506406, UGT3A1, UGT3A2, LMBRD2, MIR580, SKP2, NADK2, RANBP3L, SLC1A3, NIPBL-AS1, NIPBL, C5orf42, LOC105374727, NUP155, WDR70, GDNF, GDNF-AS1, LOC105374729, LOC101929745, EGFLAM, EGFLAM-AS4, EGFLAM-AS2, LIFR, LIFR-AS1, MIR3650, OSMR-AS1, LINC01265, OSMR, RICTOR, FYB, C9, DAB2, LOC101926940, LINC00603, PTGER4, TTC33, PRKAA1, LOC100506548, RPL37, SNORD72, CARD6, C7, MROH2B, C6, PLCXD3, OXCT1, OXCT1-AS1, C5orf51, FBXO4, LOC101926960, GHR, CCDC152, SEPP1, FLJ32255, LOC648987, ANXA2R, LOC153684, LOC100132356, LOC100506639, ZNF131, NIM1K, HMGCS1, CCL28, TMEM267, C5orf34, PAIP1, NNT-AS1, NNT, FGF10, FGF10-AS1, BRCAT107, BRCAT54, MRPS30, HCN1, EMB, PARP8, LOC100287592, LOC642366, ISL1, PELO, ITGA1, ITGA2, MOCS2, LOC257396, FST, NDUFS4, ARL15, MIR581, MIR4459, LINC01033, HSPB3, SNX18, LOC102467080 | arr[GRCh37] 5p15.33q11.2(38138_54107893)x3 |
| 1  BM | Gain | 6 | p25.3 | q26 | 161,600 | LOC285766, DUSP22, IRF4, EXOC2, HUS1B, LOC101927691, LINC01622, FOXQ1, FOXF2, MIR6720, FOXCUT, FOXC1, GMDS, GMDS-AS1, LINC01600, MYLK4, WRNIP1, SERPINB1, MIR4645, SERPINB9P1, LOC101927730, SERPINB9, SERPINB6, LINC01011, NQO2, HTATSF1P2, LOC101927759, RIPK1, BPHL, TUBB2A, LOC100507194, TUBB2B, PSMG4, SLC22A23, PXDC1, FAM50B, PRPF4B, FAM217A, C6orf201, ECI2, LOC100507506, LOC102724096, MIR7641-2, KU-MEL-3, CDYL, RPP40, LYRM4-AS1, LYRM4, PPP1R3G, MIR3691, FARS2, LOC101927972, LOC101927950, NRN1, F13A1, MIR7853, MIR5683, LY86-AS1, LY86, RREB1, SSR1, CAGE1, RIOK1, DSP, SNRNP48, BMP6, TXNDC5, BLOC1S5-TXNDC5, PIP5K1P1, BLOC1S5, EEF1E1-BLOC1S5, EEF1E1, SCARNA27, SLC35B3, LOC100506207, HULC, TFAP2A, TFAP2A-AS1, LINC00518, MIR5689HG, MIR5689, GCNT2, C6orf52, PAK1IP1, TMEM14C, TMEM14B, MAK, GCM2, SYCP2L, LOC101928191, ELOVL2, ELOVL2-AS1, SMIM13, ERVFRD-1, NEDD9, TMEM170B, ADTRP, LOC101928253, HIVEP1, EDN1, PHACTR1, TBC1D7-LOC100130357, LOC100130357, TBC1D7, GFOD1, SIRT5, NOL7, RANBP9, MCUR1, RNF182, CD83, LINC01108, JARID2, JARID2-AS1, DTNBP1, MYLIP, MIR4639, GMPR, ATXN1, LOC101928433, STMND1, RBM24, CAP2, LOC101928491, FAM8A1, NUP153, LOC105374952, KIF13A, NHLRC1, TPMT, KDM1B, DEK, RNF144B, MIR548A1, LOC101928519, LOC105374960, LOC100506885, ID4, MBOAT1, E2F3, CDKAL1, LINC00581, SOX4, CASC15, NBAT1, PRL, HDGFL1, LOC105374972, NRSN1, DCDC2, KAAG1, MRS2, GPLD1, ALDH5A1, KIAA0319, TDP2, ACOT13, C6orf62, GMNN, C6orf229, FAM65B, CMAHP, LOC101928663, CARMIL1, SCGN, HIST1H2AA, HIST1H2BA, HIST1H2APS1, SLC17A4, SLC17A1, SLC17A3, SLC17A2, TRIM38, HIST1H1A, HIST1H3A, HIST1H4A, HIST1H4B, HIST1H3B, HIST1H2AB, HIST1H2BB, HIST1H3C, HIST1H1C, HFE, HIST1H4C, HIST1H1T, HIST1H2BC, HIST1H2AC, HIST1H1E, HIST1H2BD, HIST1H2BE, HIST1H4D, HIST1H3D, HIST1H2AD, HIST1H2BF, HIST1H4E, HIST1H2BG, HIST1H2AE, HIST1H3E, HIST1H1D, HIST1H4F, HIST1H4G, HIST1H3F, HIST1H2BH, HIST1H3G, HIST1H2BI, HIST1H4H, BTN3A2, BTN2A2, BTN3A1, BTN2A3P, BTN3A3, BTN2A1, LOC285819, BTN1A1, HCG11, HMGN4, LOC105374988, ABT1, ZNF322, GUSBP2, LINC00240, LOC100270746, HIST1H2BJ, HIST1H2AG, HIST1H2BK, HIST1H4I, HIST1H2AH, MIR3143, PRSS16, POM121L2, VN1R10P, ZNF204P, ZNF391, ZNF184, LINC01012, LOC100131289, HIST1H2BL, HIST1H2AI, HIST1H3H, HIST1H2AJ, HIST1H2BM, HIST1H4J, HIST1H4K, HIST1H2AK, HIST1H2BN, HIST1H2AL, HIST1H1B, HIST1H3I, HIST1H4L, HIST1H3J, HIST1H2AM, HIST1H2BO, OR2B2, OR2B6, ZNF165, ZSCAN12P1, ZSCAN16-AS1, ZSCAN16, ZKSCAN8, ZNF192P1, TOB2P1, ZSCAN9, ZKSCAN4, NKAPL, ZSCAN26, PGBD1, ZSCAN31, ZKSCAN3, ZSCAN12, ZSCAN23, GPX6, GPX5, ZBED9, LINC00533, LINC01623, HCG14, TRIM27, LINC01556, HCG16, ZNF311, LOC100129636, OR2W1, OR2B3, OR2J3, OR2J2, LOC101929006, OR14J1, OR5V1, OR12D3, OR12D2, OR11A1, OR10C1, OR2H1, MAS1L, LINC01015, UBD, SNORD32B, OR2H2, GABBR1, MOG, ZFP57, HLA-F, HLA-F-AS1, IFITM4P, HCG4, LOC554223, HLA-G, HLA-H, HCG4B, HLA-A, HCG9, ZNRD1ASP, HLA-J, HCG8, ZNRD1, PPP1R11, RNF39, TRIM31, TRIM31-AS1, TRIM40, TRIM10, TRIM15, TRIM26, HCG17, HLA-L, MIR6891, HCG18, TRIM39, TRIM39-RPP21, RPP21, HLA-E, GNL1, PRR3, ABCF1, MIR877, PPP1R10, MRPS18B, ATAT1, C6orf136, DHX16, PPP1R18, NRM, MDC1, MDC1-AS1, TUBB, FLOT1, IER3, LINC00243, LOC105375014, DDR1, MIR4640, GTF2H4, VARS2, SFTA2, DPCR1, MUC21, MUC22, HCG22, C6orf15, PSORS1C1, CDSN, PSORS1C2, CCHCR1, TCF19, POU5F1, PSORS1C3, HCG27, HLA-C, HLA-B, MICA, HCP5, HCG26, MICB, MCCD1, DDX39B, ATP6V1G2-DDX39B, SNORD117, SNORD84, DDX39B-AS1, ATP6V1G2, NFKBIL1, LTA, TNF, LTB, LST1, NCR3, AIF1, PRRC2A, SNORA38, MIR6832, BAG6, APOM, C6orf47, GPANK1, CSNK2B, LY6G5B, LY6G5C, ABHD16A, MIR4646, LY6G6F, LY6G6E, LY6G6D, LY6G6C, C6orf25, DDAH2, CLIC1, MSH5, MSH5-SAPCD1, SAPCD1, SAPCD1-AS1, VWA7, VARS, LSM2, HSPA1L, HSPA1A, HSPA1B, C6orf48, SNORD48, SNORD52, NEU1, SLC44A4, EHMT2, C2, ZBTB12, C2-AS1, CFB, NELFE, MIR1236, SKIV2L, DXO, STK19, C4A, C4B_2, C4B, CYP21A2, CYP21A1P, TNXA, TNXB, ATF6B, FKBPL, PRRT1, LOC100507547, PPT2, PPT2-EGFL8, EGFL8, AGPAT1, MIR6721, RNF5, RNF5P1, MIR6833, AGER, PBX2, GPSM3, NOTCH4, LOC101929163, C6orf10, HCG23, BTNL2, HLA-DRA, HLA-DRB5, HLA-DRB6, HLA-DRB1, HLA-DQA1, HLA-DQB1, HLA-DQB1-AS1, HLA-DQA2, MIR3135B, HLA-DQB2, HLA-DOB, TAP2, PSMB8, PSMB8-AS1, TAP1, PSMB9, LOC100294145, HLA-DMB, HLA-DMA, BRD2, HLA-DOA, HLA-DPA1, HLA-DPB1, HLA-DPB2, COL11A2, RXRB, SLC39A7, HSD17B8, MIR219A1, RING1, HCG25, VPS52, RPS18, B3GALT4, WDR46, MIR6873, PFDN6, MIR6834, RGL2, TAPBP, ZBTB22, MIR1234, DAXX, KIFC1, PHF1, CUTA, SYNGAP1, MIR5004, ZBTB9, BAK1, GGNBP1, LINC00336, ITPR3, LOC101929188, UQCC2, MIR3934, IP6K3, LEMD2, MLN, LINC01016, MIR7159, MIR1275, GRM4, HMGA1, MIR6835, C6orf1, NUDT3, RPS10-NUDT3, RPS10, PACSIN1, SPDEF, C6orf106, LOC101929243, SNRPC, UHRF1BP1, TAF11, ANKS1A, TCP11, SCUBE3, ZNF76, DEF6, PPARD, FANCE, RPL10A, MIR7111, TEAD3, TULP1, FKBP5, MIR5690, LOC285847, ARMC12, CLPSL2, CLPSL1, CLPS, LHFPL5, SRPK1, SLC26A8, MAPK14, MAPK13, BRPF3, PNPLA1, C6orf222, ETV7, PXT1, KCTD20, STK38, SRSF3, MIR3925, PANDAR, CDKN1A, RAB44, CPNE5, PPIL1, C6orf89, PI16, MTCH1, FGD2, PIM1, TMEM217, TBC1D22B, RNF8, CMTR1, CCDC167, LOC100505530, MIR4462, MDGA1, ZFAND3, BTBD9, GLO1, DNAH8, LOC100131047, GLP1R, SAYSD1, KCNK5, KCNK17, KCNK16, KIF6, DAAM2, LOC100505635, MOCS1, LINC00951, TDRG1, LRFN2, LOC101929555, UNC5CL, TSPO2, APOBEC2, OARD1, NFYA, ADCY10P1, TREML1, TREM2, TREML2, TREML3P, TREML4, TREML5P, TREM1, NCR2, LINC01276, FOXP4-AS1, FOXP4, MIR4641, MDFI, TFEB, PGC, FRS3, PRICKLE4, TOMM6, USP49, MED20, BYSL, CCND3, TAF8, C6orf132, GUCA1A, GUCA1B, MRPS10, TRERF1, UBR2, PRPH2, ATP6V0CP3, TBCC, GLTSCR1L, LOC401261, RPL7L1, C6orf226, PTCRA, CNPY3, CNPY3-GNMT, GNMT, PEX6, PPP2R5D, MEA1, KLHDC3, RRP36, CUL7, MRPL2, KLC4, PTK7, SRF, CUL9, DNPH1, TTBK1, SLC22A7, CRIP3, ZNF318, ABCC10, MIR6780B, DLK2, TJAP1, LRRC73, YIPF3, POLR1C, XPO5, POLH, GTPBP2, MAD2L1BP, RSPH9, MRPS18A, VEGFA, LINC01512, LOC101929705, C6orf223, MRPL14, TMEM63B, CAPN11, LOC101929726, SLC29A1, HSP90AB1, SLC35B2, MIR4647, NFKBIE, TMEM151B, TCTE1, AARS2, SPATS1, CDC5L, MIR4642, LOC105375075, SUPT3H, MIR586, RUNX2, CLIC5, ENPP4, ENPP5, RCAN2, LOC101926915, LOC101926898, CYP39A1, SLC25A27, LOC101926934, TDRD6, PLA2G7, ANKRD66, MEP1A, ADGRF5, LOC101926962, ADGRF1, TNFRSF21, CD2AP, ADGRF2, ADGRF4, OPN5, PTCHD4, MUT, CENPQ, GLYATL3, C6orf141, RHAG, CRISP2, CRISP3, PGK2, LOC101927020, LOC101927048, CRISP1, DEFB133, DEFB114, DEFB113, DEFB110, DEFB112, TFAP2D, TFAP2B, PKHD1, LOC101927082, MIR206, LINCMD1, MIR133B, IL17A, IL17F, MCM3, PAQR8, EFHC1, TRAM2, TRAM2-AS1, LOC730101, TMEM14A, GSTA7P, GSTA2, GSTA1, GSTA5, GSTA3, GSTA4, ICK, FBXO9, GCM1, ELOVL5, MIR5685, RPS16P5, GCLC, LOC101927136, LINC01564, KLHL31, LRRC1, LOC101927189, MLIP-IT1, MLIP, TINAG, FAM83B, HCRTR2, GFRAL, HMGCLL1, BMP5, COL21A1, DST, LOC101930010, BEND6, KIAA1586, ZNF451, LOC101927211, BAG2, RAB23, LOC100506188, PRIM2, MIR548U, GUSBP4, LINC00680-GUSBP4, LINC00680, MTRNR2L9, KHDRBS2, LGSN, PTP4A1, PHF3, EYS, LOC441155, SLC25A51P1, LOC102723883, LOC101928280, LOC101928307, ADGRB3, LMBRD1, COL19A1, COL9A1, EVADR, FAM135A, SDHAF4, SMAP1, B3GAT2, OGFRL1, MIR30C2, MIR30A, LINC00472, LINC01626, RIMS1, KCNQ5, KCNQ5-IT1, MIR4282, KCNQ5-AS1, KHDC1L, KHDC1, DPPA5, KHDC3L, OOEP, DDX43, MB21D1, MTO1, EEF1A1, SNORD141B, SNORD141A, SLC17A5, LOC101928489, CD109, LOC101928516, COL12A1, COX7A2, TMEM30A, LOC100506804, FILIP1, LOC101928540, MIR4463, SENP6, MYO6, IMPG1, HTR1B, MEI4, IRAK1BP1, PHIP, HMGN3, HMGN3-AS1, LCAL1, LCA5, SH3BGRL2, RNY4, LINC01621, ELOVL4, TTK, BCKDHB, FAM46A, LINC01526, IBTK, TPBG, UBE3D, DOPEY1, PGM3, RWDD2A, ME1, PRSS35, SNAP91, LOC105377879, RIPPLY2, CYB5R4, MRAP2, CEP162, LINC01611, TBX18-AS1, TBX18, LOC101928820, NT5E, SNX14, SYNCRIP, SNHG5, SNORD50A, SNORD50B, HTR1E, CGA, ZNF292, GJB7, SMIM8, C6orf163, LINC01590, CFAP206, SLC35A1, RARS2, ORC3, AKIRIN2, LOC101928911, SPACA1, CNR1, LOC101928936, RNGTT, PNRC1, SRSF12, PM20D2, GABRR1, GABRR2, UBE2J1, RRAGD, ANKRD6, LYRM2, LOC101929057, MDN1, CASP8AP2, GJA10, BACH2, MIR4464, MAP3K7, MIR4643, CASC6, EPHA7, TSG1, MANEA-AS1, MANEA, FUT9, UFL1, FHL5, GPR63, NDUFAF4, KLHL32, MIR548H3, MMS22L, LOC101927314, MIR2113, POU3F2, FBXL4, MIR548AI, FAXC, COQ3, PNISR, LOC101927365, USP45, TSTD3, CCNC, PRDM13, MCHR2, MCHR2-AS1, SIM1, ASCC3, GRIK2, HACE1, LIN28B-AS1, LIN28B, BVES, BVES-AS1, POPDC3, PREP, PRDM1, ATG5, LOC105377924, AIM1, RTN4IP1, QRSL1, LOC100422737, MIR587, C6orf203, BEND3, PDSS2, SOBP, SCML4, SEC63, OSTM1, NR2E1, SNX3, LACE1, FOXO3, LINC00222, ARMC2, ARMC2-AS1, SESN1, CEP57L1, LOC100996634, CCDC162P, CD164, PPIL6, SMPD2, MICAL1, ZBTB24, AK9, FIG4, GPR6, WASF1, CDC40, METTL24, DDO, SLC22A16, CDK19, AMD1, GTF3C6, RPF2, GSTM2P1, SLC16A10, MFSD4B, REV3L, TRAF3IP2-AS1, TRAF3IP2, FYN, WISP3, TUBE1, FAM229B, LAMA4, LOC101927640, RFPL4B, LOC101927686, MARCKS, LINC01268, FLJ34503, HDAC2, LOC101927768, HS3ST5, LOC105377962, FRK, TPI1P3, NT5DC1, COL10A1, TSPYL4, DSE, TSPYL1, FAM26F, TRAPPC3L, FAM26E, FAM26D, RWDD1, RSPH4A, ZUFSP, KPNA5, FAM162B, GPRC6A, RFX6, VGLL2, ROS1, DCBLD1, GOPC, LOC101927919, NUS1, SLC35F1, LOC105377967, CEP85L, BRD7P3, PLN, LOC100287632, MCM9, ASF1A, FAM184A, MIR548B, MAN1A1, LOC285762, LOC105377975, MIR3144, TBC1D32, GJA1, HSF2, SERINC1, PKIB, FABP7, SMPDL3A, CLVS2, TRDN, HRAT13, NKAIN2, RNF217-AS1, RNF217, TPD52L1, HDDC2, LOC643623, HEY2, NCOA7, NCOA7-AS1, HINT3, TRMT11, MIR5695, CENPW, MIR588, RSPO3, RNF146, ECHDC1, KIAA0408, SOGA3, C6orf58, THEMIS, PTPRK, LOC101928140, LAMA2, ARHGAP18, TMEM244, L3MBTL3, SAMD3, TMEM200A, SMLR1, EPB41L2, AKAP7, ARG1, MED23, ENPP3, OR2A4, CTAGE9, MIR548H5, ENPP1, CTGF, MIR548AJ1, LINC01013, MOXD1, STX7, TAAR9, TAAR8, TAAR6, TAAR5, TAAR3, TAAR2, TAAR1, VNN1, VNN3, VNN2, SLC18B1, RPS12, SNORD101, SNORD100, SNORA33, LINC00326, EYA4, TARID, LINC01312, TCF21, TBPL1, SLC2A12, HMGA1P7, SGK1, LOC101928231, LINC01010, LOC101928304, ALDH8A1, HBS1L, MIR3662, MYB, MIR548A2, AHI1, LINC00271, PDE7B, MTFR2, BCLAF1, MAP7, MAP3K5, LOC101928461, LOC101928429, PEX7, SLC35D3, NHEG1, IL20RA, IL22RA2, IFNGR1, OLIG3, LOC102723649, LOC100507406, LOC100130476, TNFAIP3, PERP, ARFGEF3, PBOV1, HEBP2, NHSL1, MIR3145, FLJ46906, GVQW2, CCDC28A, ECT2L, REPS1, ABRACL, HECA, TXLNB, CITED2, LINC01625, LOC100132735, LOC100507477, LOC103352541, MIR3668, MIR4465, NMBR, VTA1, ADGRG6, LOC153910, HIVEP2, LINC01277, AIG1, ADAT2, PEX3, FUCA2, PHACTR2-AS1, PHACTR2, LTV1, ZC2HC1B, PLAGL1, HYMAI, SF3B5, STX11, UTRN, SNORA98, EPM2A, LOC100507557, FBXO30, SHPRH, GRM1, RAB32, LOC101928661, ADGB, KATNBL1P6, STXBP5-AS1, LUADT1, STXBP5, SAMD5, SASH1, UST, UST-AS1, LOC105378047, TAB2, SUMO4, ZC3H12D, PPIL4, GINM1, RPS18P9, KATNA1, LATS1, LOC645967, NUP43, PCMT1, LRP11, RAET1E-AS1, RAET1E, RAET1G, LOC105378052, ULBP2, ULBP1, RAET1K, RAET1L, ULBP3, PPP1R14C, IYD, PLEKHG1, MTHFD1L, LOC102723831, AKAP12, ZBTB2, RMND1, ARMT1, CCDC170, ESR1, SYNE1, MIR3163, SYNE1-AS1, MYCT1, VIP, FBXO5, MTRF1L, RGS17, OPRM1, IPCEF1, CNKSR3, SCAF8, MIR1273C, TIAM2, TFB1M, CLDN20, NOX3, LOC105378068, MIR1202, ARID1B, MIR4466, TMEM242, ZDHHC14, MIR3692, SNX9, SYNJ2, SYNJ2-IT1, SERAC1, GTF2H5, TULP4, TMEM181, MIR7161, DYNLT1, SYTL3, MIR3918, EZR, EZR-AS1, OSTCP1, C6orf99, RSPH3, TAGAP, LOC101929122, FNDC1, LOC102724053, SOD2, WTAP, LOC100129518, ACAT2, TCP1, SNORA20, SNORA29, MRPL18, PNLDC1, MAS1, IGF2R, AIRN, LOC729603, SLC22A1, SLC22A2, SLC22A3, LPAL2, LPA, PLG, MAP3K4, AGPAT4, AGPAT4-IT1, PARK2 | arr[GRCh37] 6p25.3q26(204908_161804651)x3 |
| 1  BM | Gain | 6 | q26 | q27 | 7,511 | PACRG, PACRG-AS2, PACRG-AS3, PACRG-AS1, DKFZp451B082, CAHM, QKI, LOC102724152, MEAT6, C6orf118, PDE10A, MIR7641-2, LINC00473, LINC00602, T, LOC101929297, PRR18, SFT2D1, LOC100289495, MPC1, RPS6KA2, RPS6KA2-IT1, MIR1913, RPS6KA2-AS1, RNASET2, MIR3939, FGFR1OP, CCR6, GPR31, LOC105378123, TCP10L2, UNC93A, TTLL2, TCP10, LOC105378127, LOC401286, LOC441178, LINC01558, MLLT4-AS1, MLLT4, HGC6.3, KIF25-AS1, KIF25, FRMD1, LOC105378137, LOC101929420, DACT2, SMOC2, LOC105378146, LOC101929460, LOC102724357, LINC01615, LOC101929504, LOC101929523, THBS2, WDR27, C6orf120, PHF10, TCTE3, ERMARD, LINC00242, LINC00574, LOC102724511, LOC154449, LOC285804, LINC01624, DLL1, FAM120B, MIR4644, PSMB1, TBP, PDCD2 | arr[GRCh37] 6q26q27(163401853_170913051)x3 |
| 1  BM | Gain | 7 | p22.3 | q11.21 | 66,608 | LOC102723672, LOC100507642, LOC105375115, FAM20C, WI2-2373I1.2, LOC442497, PDGFA, HRAT92, PRKAR1B, LOC101927000, LOC101926963, DNAAF5, SUN1, GET4, ADAP1, COX19, CYP2W1, C7orf50, MIR339, GPR146, GPER1, ZFAND2A, LOC101927021, UNCX, MICALL2, INTS1, MAFK, TMEM184A, PSMG3, PSMG3-AS1, TFAMP1, ELFN1, ELFN1-AS1, MAD1L1, MIR4655, MRM2, NUDT1, SNX8, MIR6836, EIF3B, CHST12, LOC101927181, GRIFIN, LFNG, MIR4648, BRAT1, IQCE, TTYH3, AMZ1, GNA12, CARD11, LOC100129603, SDK1, FOXK1, AP5Z1, MIR4656, RADIL, PAPOLB, MMD2, RNF216P1, RBAK, RBAK-RBAKDN, RBAKDN, ZNF890P, WIPI2, SLC29A4, TNRC18, FBXL18, MIR589, LOC221946, ACTB, FSCN1, RNF216, RNF216-IT1, MIR6874, ZNF815P, OCM, CCZ1, RSPH10B2, RSPH10B, PMS2, AIMP2, EIF2AK1, ANKRD61, USP42, CYTH3, FAM220A, RAC1, DAGLB, KDELR2, GRID2IP, ZDHHC4, C7orf26, ZNF853, ZNF316, ZNF12, PMS2CL, CCZ1B, MIR3683, LOC100131257, C1GALT1, LOC101927354, COL28A1, LOC101927391, MIOS, RPA3, UMAD1, LOC100505921, GLCCI1, ICA1, LOC100505938, NXPH1, PER4, NDUFA4, PHF14, THSD7A, TMEM106B, VWDE, LOC102725191, SCIN, ARL4A, ETV1, DGKB, AGMO, MEOX2, LOC105375166, MEOX2-AS1, ISPD, ISPD-AS1, SOSTDC1, LRRC72, ANKMY2, BZW2, TSPAN13, AGR2, AGR3, AHR, KCCAT333, LOC101927630, SNX13, PRPS1L1, HDAC9, MIR1302-6, TWIST1, FERD3L, TWISTNB, MIR3146, TMEM196, LOC101927668, MACC1, MACC1-AS1, LOC100506098, LOC101927769, LOC101927811, ITGB8, ABCB5, SP8, RPL23P8, LINC01162, SP4, MIR1183, DNAH11, CDCA7L, RAPGEF5, STEAP1B, LOC100506178, LOC401312, LOC541472, IL6, TOMM7, SNORD93, FAM126A, KLHL7-AS1, KLHL7, NUPL2, GPNMB, MALSU1, IGF2BP3, RPS2P32, TRA2A, CLK2P1, CCDC126, FAM221A, STK31, NPY, MPP6, DFNA5, OSBPL3, CYCS, C7orf31, NPVF, MIR148A, NFE2L3, HNRNPA2B1, CBX3, SNX10, LOC105375304, LOC441204, KIAA0087, C7orf71, SKAP2, HOXA1, HOTAIRM1, HOXA2, HOXA3, HOXA-AS2, HOXA4, HOXA-AS3, HOXA5, HOXA6, HOXA7, HOXA9, HOXA10-HOXA9, HOXA10-AS, MIR196B, HOXA10, HOXA11, HOXA11-AS, HOXA13, HOTTIP, EVX1-AS, EVX1, HIBADH, TSL, TAX1BP1, JAZF1, JAZF1-AS1, CREB5, TRIL, LOC100506497, CPVL, LOC101928168, CHN2, LOC102724484, PRR15, LOC646762, MIR550A3, ZNRF2P2, DPY19L2P3, WIPF3, SCRN1, FKBP14, PLEKHA8, MTURN, LOC105375218, ZNRF2, MIR550B1, MIR550A1, DKFZP586I1420, LINC01176, NOD1, GGCT, LOC401320, GARS, CRHR2, INMT, INMT-FAM188B, FAM188B, AQP1, GHRHR, ADCYAP1R1, NEUROD6, CCDC129, PPP1R17, PDE1C, LOC100130673, LSM5, AVL9, DPY19L1P1, ZNRF2P1, MIR550B2, MIR550A2, LINC00997, DPY19L1P2, KBTBD2, RP9P, FKBP9, NT5C3A, RP9, BBS9, BMPER, NPSR1-AS1, NPSR1, DPY19L1, DPY19L2P1, TBX20, LOC401324, HERPUD2, LOC101930085, LOC100506725, SEPT7-AS1, SEPT7, LOC101928618, EEPD1, KIAA0895, ANLN, AOAH, AOAH-IT1, ELMO1, MIR1200, ELMO1-AS1, GPR141, NME8, SFRP4, EPDR1, STARD3NL, TARP, TRG-AS1, AMPH, FAM183BP, VPS41, POU6F2, POU6F2-AS1, YAE1D1, RALA, LINC00265, CDK13, MPLKIP, SUGCT, LINC01450, LINC01449, INHBA, INHBA-AS1, GLI3, LINC01448, C7orf25, PSMA2, MRPL32, HECW1, HECW1-IT1, MIR3943, LOC100506895, STK17A, COA1, BLVRA, MRPS24, URGCP-MRPS24, URGCP, UBE2D4, POLR2J4, SPDYE1, RASA4CP, LINC00957, DBNL, MIR6837, PGAM2, POLM, MIR6838, AEBP1, MIR4649, POLD2, MYL7, GCK, YKT6, CAMK2B, NUDCD3, NPC1L1, DDX56, TMED4, OGDH, ZMIZ2, PPIA, H2AFV, PURB, MIR4657, MYO1G, SNHG15, SNORA9, CCM2, NACAD, TBRG4, SNORA5A, SNORA5C, SNORA5B, RAMP3, ADCY1, SEPT7P2, IGFBP1, IGFBP3, LOC730338, TNS3, LINC01447, C7orf65, LINC00525, PKD1L1, C7orf69, HUS1, SUN3, C7orf57, UPP1, ABCA13, CDC14C, VWC2, ZPBP, C7orf72, IKZF1, FIGNL1, DDC, DDC-AS1, GRB10, COBL, POM121L12, LINC01446, HPVC1, LINC01445, VSTM2A, VSTM2A-OT1, SEC61G, LOC100996654, EGFR, EGFR-AS1, ELDR, LANCL2, VOPP1, FKBP9P1, SEPT14, ZNF713, MRPS17, GBAS, PSPH, CCT6A, SNORA15, SUMF2, PHKG1, CHCHD2, NUPR2, LOC650226, LOC100240728, DKFZp434L192, LOC101928401, LOC401357, LOC100130849, MIR4283-1, MIR4283-2, ZNF479, GUSBP10, LOC105375297, MIR3147, ZNF716, ZNF733P, LOC102724738, LOC100287704, LOC100287834, LINC01005, ZNF727, ZNF735, ZNF679, ZNF736, YWHAEP1, ZNF680, LOC100128885, LOC641746, ZNF107, MIR6839, ZNF138, ZNF273, ZNF117, ERV3-1, CCT6P3, ZNF92, LOC441242, INTS4P2, CCT6P1, SNORA22, VKORC1L1, GUSB, ASL, CRCP, TPST1, LINC00174, GS1-124K5.4, GS1-124K5.11, KCTD7, LOC100996437, RABGEF1, GTF2IRD1P1, GTF2IP23, LOC644794, TMEM248, SBDS, TYW1, MIR4650-2, MIR4650-1 | arr[GRCh37] 7p22.3q11.21(41420_66649301)x3 |
| 1  BM | Gain | 7 | q11.22 | q36.3 | 89,554 | AUTS2, WBSCR17, MIR3914-1, MIR3914-2, CALN1, TYW1B, MIR4650-2, MIR4650-1, SBDSP1, SPDYE7P, POM121, NSUN5P2, TRIM74, LOC541473, LOC100101148, STAG3L1, STAG3L3, PMS2P7, PMS2P5, PMS2P2, SPDYE8P, GTF2IP4, GTF2IP1, NCF1B, NSUN5, TRIM50, FKBP6, FZD9, BAZ1B, BCL7B, TBL2, MLXIPL, VPS37D, DNAJC30, WBSCR22, STX1A, MIR4284, ABHD11-AS1, ABHD11, CLDN3, CLDN4, WBSCR27, WBSCR28, ELN, LIMK1, EIF4H, MIR590, LAT2, RFC2, CLIP2, GTF2IRD1, GTF2I, LOC101926943, NCF1, GTF2IRD2, STAG3L2, RCC1L, GTF2IRD2B, NCF1C, GATSL2, TRIM73, NSUN5P1, POM121C, SPDYE5, PMS2P3, HIP1, CCL26, CCL24, RHBDD2, POR, MIR4651, SNORA14A, TMEM120A, STYXL1, MDH2, GTF2IP7, SRRM3, HSPB1, YWHAG, SSC4D, ZP3, DTX2, FDPSP2, UPK3B, LOC100133091, POMZP3, DTX2P1-UPK3BP1-PMS2P11, PMS2P9, CCDC146, FGL2, GSAP, LOC101927243, PTPN12, APTR, RSBN1L, TMEM60, PHTF2, MAGI2, RPL13AP17, MAGI2-AS2, MAGI2-AS3, GNAI1, LOC101927269, GNAT3, CD36, SEMA3C, LOC105369146, LOC100128317, HGF, CACNA2D1, LOC101927356, PCLO, SEMA3E, SEMA3A, LOC101927378, SEMA3D, LINC00972, GRM3, KIAA1324L, LOC101927420, DMTF1, TMEM243, TP53TG1, CROT, ABCB4, ABCB1, RUNDC3B, SLC25A40, DBF4, ADAM22, SRI, LOC102723885, STEAP4, ZNF804B, C7orf62, STEAP2-AS1, DPY19L2P4, STEAP1, STEAP2, CFAP69, LOC101927446, GTPBP10, LOC101409256, CLDN12, CDK14, FZD1, MTERF1, AKAP9, CYP51A1, CYP51A1-AS1, LRRD1, KRIT1, ANKIB1, LOC105375396, GATAD1, PEX1, RBM48, FAM133B, FAM133DP, CDK6, LOC101927497, SAMD9, SAMD9L, HEPACAM2, VPS50, CALCR, MIR653, MIR489, MIR4652, TFPI2, LOC105375401, GNGT1, GNG11, BET1, COL1A2, CASD1, SGCE, PEG10, PPP1R9A, PON1, PON3, PON2, ASB4, PDK4, DYNC1I1, SLC25A13, MIR591, C7orf76, LOC100506136, SHFM1, DLX6-AS1, DLX6, DLX5, SDHAF3, TAC1, ASNS, MIR5692A1, MIR5692A2, MIR5692C2, MGC72080, OCM2, LMTK2, BHLHA15, TECPR1, BRI3, BAIAP2L1, NPTX2, TMEM130, TRRAP, MIR3609, SCARNA28, LOC101927550, SMURF1, KPNA7, MYH16, ARPC1A, ARPC1B, PDAP1, BUD31, PTCD1, ATP5J2-PTCD1, CPSF4, ATP5J2, ZNF789, ZNF394, ZKSCAN5, FAM200A, ZNF655, GS1-259H13.2, ZSCAN25, CYP3A5, CYP3A7-CYP3A51P, CYP3A7, CYP3A4, CYP3A43, OR2AE1, TRIM4, GJC3, AZGP1, AZGP1P1, ZKSCAN1, ZSCAN21, ZNF3, COPS6, MCM7, MIR25, MIR93, MIR106B, AP4M1, TAF6, CNPY4, MBLAC1, LAMTOR4, C7orf43, MIR4658, GAL3ST4, GPC2, STAG3, GATS, PVRIG, SPDYE3, PMS2P1, STAG3L5P-PVRIG2P-PILRB, STAG3L5P, PVRIG2P, MIR6840, PILRB, PILRA, ZCWPW1, MEPCE, PPP1R35, C7orf61, TSC22D4, NYAP1, AGFG2, SAP25, LRCH4, ZASP, FBXO24, PCOLCE-AS1, PCOLCE, MOSPD3, TFR2, ACTL6B, LOC105375429, GNB2, GIGYF1, POP7, EPO, ZAN, EPHB4, SLC12A9, TRIP6, MIR6875, SRRT, UFSP1, ACHE, MUC3A, MUC12, LOC102724094, MUC17, TRIM56, SERPINE1, AP1S1, MIR4653, VGF, NAT16, MOGAT3, PLOD3, ZNHIT1, CLDN15, FIS1, LOC101927746, IFT22, COL26A1, LINC01007, MYL10, CUX1, SH2B2, MIR4285, SPDYE6, LOC100289561, LOC100630923, PRKRIP1, MIR548O, ORAI2, ALKBH4, LRWD1, MIR5090, MIR4467, POLR2J, RASA4B, POLR2J3, SPDYE2B, SPDYE2, RASA4, POLR2J2, UPK3BL, FAM185A, FBXL13, LRRC17, ARMC10, NAPEPLD, RPL19P12, DPY19L2P2, PMPCB, DNAJC2, PSMC2, SLC26A5, LOC101927870, RELN, ORC5, LHFPL3, LHFPL3-AS1, LHFPL3-AS2, LINC01004, KMT2E-AS1, KMT2E, SRPK2, PUS7, RINT1, EFCAB10, ATXN7L1, CDHR3, SYPL1, NAMPT, CCDC71L, PIK3CG, PRKAR2B, HBP1, COG5, GPR22, DUS4L, BCAP29, SLC26A4-AS1, SLC26A4, CBLL1, SLC26A3, DLD, LAMB1, LAMB4, NRCAM, PNPLA8, THAP5, DNAJB9, C7orf66, EIF3IP1, IMMP2L, LRRN3, DOCK4, DOCK4-AS1, ZNF277, IFRD1, LSMEM1, LOC100996249, LOC101928012, TMEM168, BMT2, HRAT17, GPR85, LINC00998, PPP1R3A, FOXP2, MIR3666, MDFIC, LINC01393, LINC01392, TFEC, TES, LOC102724434, CAV2, CAV1, LINC01510, MET, CAPZA2, ST7-AS1, ST7, ST7-OT4, MIR6132, ST7-AS2, ST7-OT3, WNT2, ASZ1, CFTR, CTTNBP2, LSM8, ANKRD7, LVCAT5, KCND2, TSPAN12, ING3, CPED1, WNT16, FAM3C, PTPRZ1, AASS, FEZF1, FEZF1-AS1, CADPS2, RNF133, RNF148, TAS2R16, SLC13A1, IQUB, NDUFA5, ASB15, LOC102724555, LMOD2, WASL, RNU6-2, HYALP1, HYAL4, SPAM1, LOC105375483, TMEM229A, LOC101928211, GPR37, C7orf77, POT1, POT1-AS1, LOC101928283, LOC101928254, GRM8, MIR592, LOC101928333, ZNF800, LOC100506682, GCC1, ARF5, FSCN3, PAX4, SND1, SND1-IT1, LRRC4, MIR593, MIR129-1, LEP, MGC27345, RBM28, PRRT4, IMPDH1, HILPDA, METTL2B, LINC01000, FAM71F2, FAM71F1, CALU, OPN1SW, CCDC136, FLNC, ATP6V1F, LOC100130705, KCP, IRF5, TNPO3, TPI1P2, LOC407835, TSPAN33, SMO, AHCYL2, STRIP2, SMKR1, NRF1, MIR182, MIR96, MIR183, UBE2H, ZC3HC1, KLHDC10, TMEM209, SSMEM1, CPA2, CPA4, CPA5, LOC105375504, CPA1, CEP41, MEST, MESTIT1, MIR335, COPG2, TSGA13, KLF14, MIR29A, MIR29B1, LINC-PINT, LOC100506860, MKLN1, MKLN1-AS, PODXL, LOC101928782, PLXNA4, LOC101928807, FLJ40288, LOC100506937, CHCHD3, MIR3654, LOC105375512, EXOC4, MIR6133, LOC101928861, LRGUK, SLC35B4, AKR1B1, AKR1B10, AKR1B15, BPGM, CALD1, AGBL3, C7orf49, TMEM140, WDR91, MIR6509, STRA8, CNOT4, NUP205, C7orf73, SLC13A4, FAM180A, MTPN, LUZP6, CHRM2, LOC349160, MIR490, PTN, DGKI, CREB3L2, LOC100130880, AKR1D1, MIR4468, TRIM24, SVOPL, ATP6V0A4, TMEM213, KIAA1549, ZC3HAV1L, ZC3HAV1, TTC26, UBN2, LUC7L2, FMC1, C7orf55-LUC7L2, LOC100129148, KLRG2, CLEC2L, HIPK2, TBXAS1, PARP12, KDM7A, JHDM1D-AS1, SLC37A3, RAB19, MKRN1, DENND2A, ADCK2, NDUFB2-AS1, NDUFB2, BRAF, MRPS33, TMEM178B, AGK, KIAA1147, WEE2-AS1, WEE2, SSBP1, TAS2R3, TAS2R4, TAS2R5, PRSS37, OR9A4, CLEC5A, TAS2R38, MGAM, MGAM2, MOXD2P, PRSS58, TRY2P, MTRNR2L6, PRSS1, PRSS3P2, EPHB6, TRPV6, TRPV5, C7orf34, KEL, OR9A2, OR6V1, OR6W1P, PIP, TAS2R39, TAS2R40, LOC105375545, GSTK1, TMEM139, CASP2, CLCN1, FAM131B, LOC100507507, ZYX, MIR6892, EPHA1, EPHA1-AS1, TAS2R60, TAS2R41, CTAGE15, TCAF2, TCAF2P1, CTAGE6, LOC154761, TCAF1, OR2F2, OR2F1, OR6B1, OR2A5, OR2A25, OR2A12, OR2A2, OR2A14, CTAGE4, ARHGEF35, LOC101928605, OR2A1-AS1, OR2A1, OR2A42, OR2A9P, OR2A20P, OR2A7, ARHGEF34P, CTAGE8, ARHGEF5, NOBOX, TPK1, CNTNAP2, LOC101928700, MIR548F4, LOC105375556, MIR548T, C7orf33, CUL1, EZH2, GHET1, PDIA4, ZNF786, ZNF425, ZNF398, ZNF282, ZNF212, ZNF783, LOC155060, ZNF777, ZNF746, ZNF767P, KRBA1, ZNF467, SSPO, ZNF862, ATP6V0E2-AS1, ATP6V0E2, ACTR3C, LRRC61, ZBED6CL, RARRES2, REPIN1, ZNF775, LOC728743, LINC00996, GIMAP8, GIMAP7, GIMAP4, GIMAP6, GIMAP2, GIMAP1, GIMAP1-GIMAP5, GIMAP5, TMEM176B, TMEM176A, AOC1, KCNH2, NOS3, ATG9B, ABCB8, ASIC3, CDK5, SLC4A2, FASTK, TMUB1, AGAP3, GBX1, ASB10, IQCA1L, ABCF2, CHPF2, MIR671, SMARCD3, NUB1, WDR86, WDR86-AS1, CRYGN, MIR3907, RHEB, PRKAG2, PRKAG2-AS1, GALNTL5, GALNT11, KMT2C, FABP5P3, LINC01003, XRCC2, ACTR3B, LINC01287, DPP6, LOC101929998, PAXIP1-AS2, PAXIP1, PAXIP1-AS1, HTR5A-AS1, HTR5A, INSIG1, BLACE, EN2, CNPY1, LOC100506302, RBM33, SHH, LOC389602, LOC285889, LINC01006, LINC00244, C7orf13, RNF32, LMBR1, NOM1, MNX1, MNX1-AS1, UBE3C, DNAJB6, LOC101927914, PTPRN2, MIR153-2, LOC100506585, MIR595, LINC01022, MIR5707, NCAPG2, ESYT2, WDR60, LINC00689, VIPR2 | arr[GRCh37] 7q11.22q36.3(69564375_159118443)x3 |
| 1  BM | Gain | 8 | p12 | q11.21 | 22,337 | LINC00589, LOC101929450, LOC101929470, FAM183CP, MIR3148, SARAF, LEPROTL1, MBOAT4, DCTN6, RBPMS-AS1, RBPMS, GTF2E2, SMIM18, GSR, UBXN8, PPP2CB, TEX15, PURG, WRN, NRG1, NRG1-IT1, NRG1-IT3, FUT10, MAK16, TTI2, RNF122, DUSP26, LINC01288, UNC5D, LOC101929550, KCNU1, MIR1268A, LINC01605, ZNF703, LOC101929622, LOC102723701, ERLIN2, LOC728024, PROSC, ADGRA2, BRF2, RAB11FIP1, GOT1L1, ADRB3, EIF4EBP1, ASH2L, STAR, LSM1, BAG4, DDHD2, PLPP5, WHSC1L1, LETM2, FGFR1, C8orf86, RNF5P1, TACC1, PLEKHA2, HTRA4, TM2D2, ADAM9, ADAM32, ADAM5, ADAM3A, LOC100130964, ADAM18, ADAM2, IDO1, IDO2, C8orf4, ZMAT4, SFRP1, MIR548AO, GOLGA7, GINS4, LOC102723729, GPAT4, NKX6-3, ANK1, MIR486-1, MIR486-2, KAT6A, LOC105379393, AP3M2, PLAT, LOC101929897, IKBKB, POLB, DKK4, VDAC3, SLC20A2, SMIM19, CHRNB3, CHRNA6, THAP1, RNF170, MIR4469, HOOK3, FNTA, POMK, HGSNAT, POTEA, LINC00293, LOC100287846, SPIDR, CEBPD, PRKDC, MCM4, UBE2V2, LOC101929268, LOC101929217, EFCAB1, SNAI2, C8orf22, LOC100507464, SNTG1 | arr[GRCh37] 8p12q11.21(29490607_51827773)x3 |
| 1  BM | Gain | 8 | q21.13 | q24.3 | 63,073 | LOC101927141, LINC01419, RALYL, LRRCC1, LOC102723322, E2F5, C8orf59, CA13, CA1, CA3, CA3-AS1, CA2, REXO1L2P, PSKH2, ATP6V0D2, SLC7A13, WWP1, RMDN1, CPNE3, CNGB3, CNBD1, DCAF4L2, MMP16, LOC101929709, RIPK2, OSGIN2, NBN, DECR1, CALB1, LINC00534, LINC01030, TMEM64, NECAB1, C8orf88, TMEM55A, OTUD6B-AS1, OTUD6B, LRRC69, MIR4661, SLC26A7, RUNX1T1, MIR7641-2, LOC102724710, FLJ46284, TRIQK, MIR8084, C8orf87, LINC00535, FAM92A1, RBM12B, RBM12B-AS1, TMEM67, MIR378D2, PDP1, CDH17, GEM, RAD54B, FSBP, KIAA1429, LOC100288748, ESRP1, DPY19L4, INTS8, CCNE2, TP53INP1, NDUFAF6, LOC105375650, MIR3150B, MIR3150A, PLEKHF2, LINC01298, C8orf37, C8orf37-AS1, LOC100500773, GDF6, UQCRB, MTERF3, PTDSS1, LOC102724804, SDC2, CPQ, LOC101927066, TSPYL5, MTDH, LAPTM4B, MATN2, RPL30, SNORA72, ERICH5, RIDA, POP1, NIPAL2, KCNS2, STK3, OSR2, VPS13B, MIR599, MIR875, COX6C, RGS22, MIR1273A, FBXO43, POLR2K, SPAG1, RNF19A, MIR4471, ANKRD46, SNX31, PABPC1, MIR7705, YWHAZ, FLJ42969, ZNF706, NACAP1, GRHL2, NCALD, LOC104054148, MIR5680, RRM2B, UBR5-AS1, UBR5, ODF1, KLF10, AZIN1, AZIN1-AS1, ATP6V1C1, BAALC-AS2, BAALC, MIR3151, BAALC-AS1, FZD6, CTHRC1, SLC25A32, DCAF13, RIMS2, DCSTAMP, DPYS, LRP12, ZFPM2, ZFPM2-AS1, OXR1, ABRA, ANGPT1, RSPO2, EIF3E, EMC2, TMEM74, TRHR, NUDCD1, ENY2, PKHD1L1, EBAG9, SYBU, LOC100132813, KCNV1, LINC01608, LINC01609, CSMD3, MIR2053, TRPS1, LINC00536, EIF3H, LOC105375713, UTP23, RAD21, RAD21-AS1, MIR3610, AARD, SLC30A8, MED30, EXT1, SAMD12, SAMD12-AS1, TNFRSF11B, COLEC10, LOC101927513, MAL2, MIR548AZ, NOV, ENPP2, TAF2, DSCC1, DEPTOR, COL14A1, MRPL13, MTBP, SNTB1, LOC101927543, HAS2, HAS2-AS1, LOC105375734, LINC01151, ZHX2, DERL1, TBC1D31, FAM83A, FAM83A-AS1, MIR4663, C8orf76, ZHX1-C8orf76, ZHX1, ATAD2, MIR548D1, WDYHV1, FBXO32, KLHL38, ANXA13, FAM91A1, FER1L6, FER1L6-AS1, FER1L6-AS2, LOC101927588, TMEM65, TRMT12, RNF139-AS1, RNF139, TATDN1, MIR6844, NDUFB9, MTSS1, MIR4662B, MIR4662A, LINC00964, ZNF572, LOC105375744, SQLE, KIAA0196, NSMCE2, TRIB1, LINC00861, LOC101927657, FAM84B, PCAT1, PCAT2, PRNCR1, CASC19, CCAT1, CASC21, CASC8, CCAT2, POU5F1B, CASC11, MYC, PVT1, MIR1204, TMEM75, MIR1205, MIR1206, MIR1207, MIR1208, LINC00824, LINC00977, CCDC26, MIR3686, GSDMC, FAM49B, MIR5194, ASAP1, ASAP1-IT2, ASAP1-IT1, ADCY8, EFR3A, OC90, HHLA1, KCNQ3, HPYR1, LRRC6, TMEM71, PHF20L1, TG, SLA, MIR7848, WISP1, NDRG1, ST3GAL1, LOC105375773, LOC101927798, LOC101927822, ZFAT, ZFAT-AS1, MIR30B, MIR30D, NCRNA00250, LOC101927845, LINC01591, KHDRBS3, LOC101927915, FAM135B, COL22A1, KCNK9, TRAPPC9, CHRAC1, AGO2, PTK2, DENND3, SLC45A4, LOC105375787, LINC01300, GPR20, PTP4A3, MROH5, MIR1302-7, MIR4539, MIR4472-1, LINC00051, TSNARE1, ADGRB1, ARC, LOC101928087, JRK, PSCA, LY6K, LOC100288181, THEM6, SLURP1, LYPD2, LYNX1, LY6D, GML, CYP11B1, CYP11B2, LOC100133669, CDC42P3, LY6E, C8orf31, LY6H, GPIHBP1, ZFP41, GLI4, MINCR, ZNF696, TOP1MT, RHPN1-AS1, RHPN1, MAFA-AS1, MAFA, ZC3H3, GSDMD, MROH6, NAPRT, EEF1D, TIGD5, PYCRL, TSTA3, ZNF623, ZNF707, BREA2, CCDC166, LOC101928160, MAPK15, FAM83H, MIR4664, FAM83H-AS1, LOC105375800, SCRIB, MIR937, PUF60, NRBP2, MIR6845, EPPK1, PLEC, MIR661, PARP10, GRINA, SPATC1, OPLAH, MIR6846, EXOSC4, MIR6847, GPAA1, CYC1, SHARPIN, MAF1, WDR97, HGH1, MROH1, MIR7112, SCX, BOP1, HSF1, DGAT1, MIR6848, SCRT1, TMEM249, FBXL6, SLC52A2, LOC101928902, ADCK5, CPSF1, MIR939, MIR6849, SLC39A4, VPS28, TONSL, TONSL-AS1, MIR6893, CYHR1, KIFC2, FOXH1, PPP1R16A, GPT, MFSD3, RECQL4, LRRC14, LRRC24, C8orf82, ARHGAP39, ZNF251, ZNF34, RPL8, MIR6850, ZNF517, ZNF7, COMMD5, ZNF250, ZNF16, ZNF252P, TMED10P1, ZNF252P-AS1, C8orf33 | arr[GRCh37] 8q21.13q24.3(83219271_146292734)x3 |
| 1  BM | Gain | 10 | p14 | p11.1 | 28,105 | CELF2, CELF2-AS2, CELF2-AS1, USP6NL, ECHDC3, PROSER2, PROSER2-AS1, UPF2, DHTKD1, SEC61A2, MIR548AK, NUDT5, CDC123, CAMK1D, MIR4480, MIR4481, MIR548Q, CCDC3, OPTN, MCM10, RNU6-2, UCMA, PHYH, SEPHS1, BEND7, PRPF18, FRMD4A, LOC101928453, MIR4293, MIR1265, FAM107B, CDNF, HSPA14, SUV39H2, DCLRE1C, MEIG1, LOC105376430, OLAH, ACBD7, C10orf111, RPP38, NMT2, PPIAP30, FAM171A1, ITGA8, FAM188A, PTER, C1QL3, RSU1, CUBN, TRDMT1, VIM-AS1, VIM, ST8SIA6, ST8SIA6-AS1, HACD1, STAM-AS1, STAM, TMEM236, MRC1, MIR511, SLC39A12, SLC39A12-AS1, CACNB2, NSUN6, ARL5B, MALRD1, LOC101928834, PLXDC2, MIR4675, NEBL, C10orf113, NEBL-AS1, CASC10, MIR1915, SKIDA1, MLLT10, DNAJC1, EBLN1, LOC100130992, COMMD3, COMMD3-BMI1, BMI1, SPAG6, LOC100499489, PIP4K2A, ARMC3, MSRB2, PTF1A, C10orf67, MIR1254-2, OTUD1, KIAA1217, MIR603, ARHGAP21, PRTFDC1, ENKUR, THNSL1, LINC01516, GPR158-AS1, GPR158, LINC00836, LOC101929073, MYO3A, GAD2, APBB1IP, LOC101929117, LINC00264, LINC00202-2, PDSS1, ABI1, LINC00202-1, ANKRD26, YME1L1, MASTL, ACBD5, LRRC37A6P, PTCHD3, RAB18, MKX, MKX-AS1, ARMC4, MPP7, SNORD130, MIR8086, LOC105376468, WAC-AS1, WAC, BAMBI, LINC01517, LINC00837, C10orf126, LYZL1, SVIL-AS1, SVIL, MIR604, MIR938, KIAA1462, LOC101929279, MTPAP, GOLGA2P6, MIR7162, MAP3K8, LYZL2, SVILP1, LOC105376480, ZNF438, LOC101929352, ZEB1-AS1, ZEB1, ARHGAP12, KIF5B, EPC1, LOC102031319, LOC101929431, CCDC7, ITGB1, SNORA86, NRP1, LINC00838, PARD3, PARD3-AS1, CUL2, MIR3611, CREM, CCNY, GJD4, FZD8, MIR4683, PCAT5, ANKRD30A, LINC00993, MTRNR2L7, ZNF248, ZNF33BP1, ZNF25, ZNF33A, ZNF37A, LOC100129055, HSD17B7P2, SEPT7P9, LINC00999, ACTR3BP5 | arr[GRCh37] 10p14p11.1(11041280_39146676)x3 |
| 8460-8477_3.OSCHP | Gain | 11 | q14.1 | q25 | 49,932 | DLG2, TMEM126B, TMEM126A, CREBZF, CCDC89, SYTL2, CCDC83, PICALM, EED, MIR6755, HIKESHI, CCDC81, ME3, PRSS23, OR7E2P, FZD4, LOC100506368, TMEM135, LOC105369423, RAB38, MIR3166, CTSC, GRM5-AS1, GRM5, TYR, NOX4, FOLH1B, TRIM77, TRIM49, TRIM53AP, TRIM64B, TRIM49D2, TRIM49D1, TRIM64, TRIM49C, UBTFL1, NAALAD2, CHORDC1, DISC1FP1, MIR4490, MIR1261, FAT3, LOC105369431, MTNR1B, SLC36A4, CCDC67, SMCO4, CEP295, SCARNA9, SNORA25, SNORA32, SNORD6, SNORA1, SNORA8, SNORD5, SNORA18, MIR1304, SNORA40, TAF1D, C11orf54, MED17, VSTM5, HEPHL1, PANX1, IZUMO1R, GPR83, MRE11A, MIR548L, ANKRD49, C11orf97, FUT4, LOC105369438, PIWIL4, AMOTL1, CWC15, KDM4D, KDM4E, SRSF8, ENDOD1, LOC101929295, SESN3, LOC100129203, FAM76B, CEP57, MTMR2, MAML2, MIR1260B, CCDC82, JRKL, JRKL-AS1, LOC105369443, CNTN5, LOC100128386, ARHGAP42, TMEM133, PGR, LOC101054525, TRPC6, MIR3920, ANGPTL5, CEP126, C11orf70, YAP1, BIRC3, BIRC2, TMEM123, LOC101928424, LOC102723838, MMP7, MMP20, MMP27, MMP8, MMP10, WTAPP1, MMP1, MMP3, MMP12, MMP13, DCUN1D5, DYNC2H1, MIR4693, PDGFD, DDI1, MIR7641-1, LOC102723895, CASP12, LOC643733, CASP4, CASP5, CASP1, CARD16, CASP1P2, CARD17, CARD18, GRIA4, MSANTD4, KBTBD3, AASDHPPT, LOC105369473, LOC101928535, GUCY1A2, CWF19L2, ALKBH8, ELMOD1, LOC643923, SLN, SLC35F2, RAB39A, CUL5, ACAT1, NPAT, ATM, C11orf65, KDELC2, EXPH5, DDX10, C11orf87, ZC3H12C, RDX, LOC105369486, FDX1, ARHGAP20, C11orf53, COLCA1, COLCA2, MIR4491, POU2AF1, LOC100132078, BTG4, MIR34B, MIR34C, C11orf88, LAYN, SIK2, PPP2R1B, ALG9, FDXACB1, C11orf1, CRYAB, HSPB2, HSPB2-C11orf52, C11orf52, DIXDC1, DLAT, PIH1D2, C11orf57, TIMM8B, SDHD, IL18, TEX12, BCO2, PTS, PLET1, LOC100132686, LOC283140, LOC101928823, LOC387810, LOC101928847, NCAM1, NCAM1-AS1, TTC12, ANKK1, DRD2, MIR4301, TMPRSS5, ZW10, CLDN25, USP28, HTR3B, HTR3A, ZBTB16, NNMT, LOC101928940, C11orf71, RBM7, REXO2, NXPE1, NXPE4, NXPE2, CADM1, LOC105369509, LOC105369507, LOC101928985, LINC00900, LOC101929011, BUD13, ZPR1, APOA5, APOA4, APOC3, APOA1, APOA1-AS, SIK3, PAFAH1B2, SIDT2, LOC100652768, TAGLN, PCSK7, RNF214, BACE1, BACE1-AS, CEP164, DSCAML1, FXYD2, FXYD6-FXYD2, FXYD6, TMPRSS13, IL10RA, TMPRSS4-AS1, TMPRSS4, SCN4B, SCN2B, JAML, MPZL3, MPZL2, CD3E, CD3D, CD3G, UBE4A, LOC100131626, ATP5L, KMT2A, LOC101929089, TTC36, TMEM25, IFT46, ARCN1, PHLDB1, MIR6716, TREH, DDX6, CXCR5, BCL9L, MIR4492, UPK2, FOXR1, CCDC84, RPL23AP64, RPS25, TRAPPC4, MIR3656, SLC37A4, HYOU1, VPS11, HMBS, H2AFX, DPAGT1, C2CD2L, HINFP, ABCG4, NLRX1, PDZD3, CCDC153, CBL, MCAM, MIR6756, RNF26, C1QTNF5, MFRP, USP2, USP2-AS1, THY1, NECTIN1, LOC102724301, TRIM29, OAF, POU2F3, LOC649133, TMEM136, ARHGEF12, GRIK4, LOC105369532, LOC101929227, LOC101929208, TBCEL, TECTA, SC5D, SORL1, MIR100HG, MIR125B1, BLID, MIRLET7A2, MIR100, UBASH3B, CRTAM, C11orf63, BSX, LOC341056, HSPA8, CLMP, MIR4493, GRAMD1B, SCN3B, ZNF202, OR6X1, OR6M1, TMEM225, OR8D4, OR4D5, OR6T1, OR10S1, OR10G4, OR10G9, OR10G8, OR10G7, VWA5A, OR8G2, OR8G1, OR8G5, OR8D1, OR8D2, OR8B2, OR8B3, OR8B4, OR8B8, OR8B12, OR8A1, PANX3, TBRG1, SIAE, MIR7641-2, SPA17, NRGN, VSIG2, ESAM, LOC101929340, MSANTD2, LOC100507283, ROBO3, ROBO4, HEPN1, HEPACAM, CCDC15, SLC37A2, TMEM218, PKNOX2-AS1, PKNOX2, FEZ1, LOC403312, EI24, STT3A-AS1, STT3A, CHEK1, ACRV1, PATE1, PATE2, PATE3, PATE4, HYLS1, PUS3, DDX25, CDON, RPUSD4, FAM118B, SRPRA, FOXRED1, TIRAP, DCPS, ST3GAL4-AS1, ST3GAL4, KIRREL3, LOC101929427, KIRREL3-AS2, MIR3167, KIRREL3-AS3, LOC101929473, LOC101929497, ETS1, MIR6090, LOC101929517, LOC101929538, FLI1, SENCR, KCNJ1, KCNJ5, C11orf45, TP53AIP1, ARHGAP32, BARX2, LINC01395, TMEM45B, NFRKB, PRDM10, LINC00167, APLP2, ST14, ZBTB44, ADAMTS8, ADAMTS15, MIR8052, C11orf44, LOC100507431, LOC103611081, SNX19, NTM, LOC101929653, NTM-AS1, NTM-IT, OPCML, LOC646522, SPATA19, MIR4697HG, MIR4697, IGSF9B, LOC100128239, JAM3, NCAPD3, VPS26B, THYN1, ACAD8, GLB1L3, GLB1L2, B3GAT1, LOC283177, LOC100507548 | arr[GRCh37] 11q14.1q25(85007025_134938847)x3 |
| 1  BM | Gain | 12 | p13.33 | q24.33 | 133,629 | IQSEC3, LOC574538, SLC6A12, LOC101929384, SLC6A13, LOC102723544, KDM5A, CCDC77, B4GALNT3, NINJ2, LOC105369595, LOC100049716, WNK1, RAD52, ERC1, LINC00942, FBXL14, WNT5B, MIR3649, ADIPOR2, CACNA2D4, LRTM2, LINC00940, DCP1B, CACNA1C-IT2, CACNA1C, CACNA1C-AS4, CACNA1C-IT3, CACNA1C-AS2, CACNA1C-AS1, LOC283440, FKBP4, ITFG2, NRIP2, LOC100507424, FOXM1, RHNO1, TULP3, TEAD4, TSPAN9, PRMT8, THCAT155, CRACR2A, PARP11, CCND2-AS1, CCND2, TIGAR, FGF23, FGF6, C12orf4, RAD51AP1, DYRK4, AKAP3, NDUFA9, LOC101929549, GALNT8, KCNA6, KCNA1, KCNA5, LOC101929584, NTF3, ANO2, VWF, CD9, PLEKHG6, TNFRSF1A, SCNN1A, LTBR, CD27-AS1, CD27, TAPBPL, VAMP1, MRPL51, NCAPD2, SCARNA10, GAPDH, IFFO1, NOP2, CHD4, SCARNA11, LPAR5, ACRBP, ING4, ZNF384, PIANP, COPS7A, MLF2, PTMS, LAG3, CD4, GPR162, P3H3, GNB3, CDCA3, USP5, TPI1, SPSB2, LOC105369632, RPL13P5, DSTNP2, LRRC23, ENO2, ATN1, C12orf57, PTPN6, LOC105369635, MIR200C, MIR141, PHB2, SCARNA12, EMG1, LPCAT3, C1S, C1R, C1RL, C1RL-AS1, RBP5, CLSTN3, PEX5, ACSM4, CD163L1, CD163, APOBEC1, GDF3, DPPA3, CLEC4C, NANOGNB, NANOG, SLC2A14, SLC2A3, FOXJ2, C3AR1, NECAP1, CLEC4A, POU5F1P3, ZNF705A, FAM66C, FAM90A1, FAM86FP, LOC101927905, LINC00937, CLEC6A, CLEC4D, CLEC4E, AICDA, MFAP5, RIMKLB, A2ML1, PHC1, M6PR, KLRG1, LINC00612, A2M-AS1, A2M, PZP, A2MP1, MIR1244-4, MIR1244-3, MIR1244-1, MIR1244-2, LINC00987, LOC642846, LOC101930452, LOC101928030, DDX12P, KLRB1, LOC374443, CLEC2D, CLECL1, CD69, KLRF1, CLEC2B, KLRF2, CLEC2A, LOC100506159, LOC400002, CLEC12A, CLEC1B, CLEC12B, LOC102724020, CLEC9A, CLEC1A, CLEC7A, OLR1, TMEM52B, GABARAPL1, KLRD1, LOC101928100, KLRK1, KLRC4-KLRK1, KLRC4, KLRC3, KLRC2, KLRC1, KLRA1P, MAGOHB, STYK1, YBX3, LOC101928162, TAS2R7, TAS2R8, TAS2R9, TAS2R10, PRR4, PRH1-PRR4, PRH1, TAS2R13, PRH2, PRH1-TAS2R14, TAS2R14, TAS2R50, TAS2R20, TAS2R19, TAS2R31, TAS2R46, TAS2R43, TAS2R30, SMIM10L1, TAS2R42, PRB3, PRB4, PRB1, PRB2, LINC01252, ETV6, BCL2L14, LRP6, MANSC1, LOH12CR2, BORCS5, DUSP16, CREBL2, GPR19, CDKN1B, APOLD1, MIR613, DDX47, RPL13AP20, GPRC5A, MIR614, GPRC5D, HEBP1, LOC100506314, HTR7P1, FAM234B, MIR7641-2, GSG1, EMP1, LINC01559, GRIN2B, ATF7IP, PLBD1, PLBD1-AS1, GUCY2C, HIST4H4, H2AFJ, WBP11, C12orf60, SMCO3, ART4, MGP, ERP27, ARHGDIB, PDE6H, LINC01489, RERG, RERG-AS1, PTPRO, EPS8, STRAP, DERA, SLC15A5, MGST1, LMO3, SKP1P2, MIR3974, RERGL, PIK3C2G, PLCZ1, CAPZA3, PLEKHA5, AEBP2, LOC100506393, PDE3A, SLCO1C1, SLCO1B3, SLCO1B7, SLCO1B1, SLCO1A2, IAPP, PYROXD1, RECQL, GOLT1B, SPX, GYS2, LDHB, KCNJ8, ABCC9, CMAS, ST8SIA1, C2CD5, LOC105369691, ETNK1, LOC101928441, SOX5, MIR920, LOC101928471, LINC00477, BCAT1, C12orf77, LOC645177, LRMP, CASC1, LYRM5, KRAS, LMNTD1, MIR4302, RASSF8-AS1, RASSF8, BHLHE41, SSPN, ITPR2, ASUN, FGFR1OP2, TM7SF3, MED21, C12orf71, STK38L, ARNTL2, ARNTL2-AS1, SMCO2, PPFIBP1, REP15, MRPS35, MANSC4, KLHL42, PTHLH, CCDC91, FAR2, LOC100506606, ERGIC2, OVCH1-AS1, OVCH1, TMTC1, IPO8, CAPRIN2, LOC645485, LINC00941, TSPAN11, DDX11-AS1, DDX11, FAM60A, FLJ13224, DENND5B, DENND5B-AS1, ETFBKMT, AMN1, H3F3C, LOC105369723, KIAA1551, BICD1, FGD4, DNM1L, YARS2, PKP2, SYT10, ALG10, ALG10B, CPNE8, KIF21A, ABCD2, C12orf40, SLC2A13, LRRK2, MUC19, CNTN1, PDZRN4, LOC101927038, GXYLT1, YAF2, ZCRB1, MIR7851, PPHLN1, PRICKLE1, LOC101927058, LOC105369738, LOC105369739, ADAMTS20, PUS7L, IRAK4, TWF1, TMEM117, NELL2, DBX2, RACGAP1P, PLEKHA8P1, RNY5, ANO6, LINC00938, ARID2, SCAF11, SLC38A1, SLC38A2, LOC100288798, SLC38A4, AMIGO2, PCED1B, MIR4698, PCED1B-AS1, LOC105369747, MIR4494, RPAP3, ENDOU, RAPGEF3, SLC48A1, HDAC7, VDR, TMEM106C, COL2A1, SENP1, PFKM, MIR6505, ASB8, CCDC184, OR10AD1, H1FNT, ZNF641, ANP32D, C12orf54, OR8S1, LALBA, KANSL2, SNORA2C, MIR1291, SNORA2A, SNORA2B, CCNT1, LINC00935, ADCY6, MIR4701, LOC100506125, CACNB3, DDX23, RND1, CCDC65, FKBP11, ARF3, WNT10B, WNT1, DDN, PRKAG1, KMT2D, RHEBL1, DHH, LMBR1L, TUBA1B, TUBA1A, TUBA1C, LOC101927267, PRPH, TROAP, C1QL4, DNAJC22, SPATS2, LOC100335030, KCNH3, MCRS1, FAM186B, PRPF40B, FMNL3, TMBIM6, NCKAP5L, BCDIN3D-AS1, BCDIN3D, FAIM2, LOC283332, LOC101927292, AQP2, LOC101927318, AQP5, AQP6, RACGAP1, ASIC1, SMARCD1, GPD1, COX14, CERS5, LIMA1, MIR1293, FAM186A, LARP4, SNORD133, DIP2B, ATF1, TMPRSS12, METTL7A, HIGD1C, SLC11A2, LETMD1, CSRNP2, TFCP2, POU6F1, DAZAP2, SMAGP, BIN2, CELA1, GALNT6, SLC4A8, SCN8A, FIGNL2, LOC105369971, ANKRD33, ACVRL1, ACVR1B, GRASP, NR4A1, ATG101, OR7E47P, KRT80, C12orf80, LINC00592, KRT7, KRT86, KRT81, KRT83, KRT85, KRT84, KRT82, KRT75, KRT6B, KRT6C, KRT6A, KRT5, KRT71, KRT74, KRT72, KRT73, KRT73-AS1, KRT2, KRT1, KRT77, KRT76, KRT3, KRT4, KRT79, KRT78, KRT8, KRT18, EIF4B, LOC283335, TNS2, MIR6757, SPRYD3, IGFBP6, SOAT2, CSAD, ZNF740, ITGB7, RARG, MFSD5, ESPL1, PFDN5, C12orf10, AAAS, SP7, SP1, AMHR2, PRR13, PCBP2, PCBP2-OT1, MAP3K12, TARBP2, NPFF, ATF7, LOC100652999, ATP5G2, CALCOCO1, CISTR, HOXC13-AS, HOXC13, HOXC12, HOTAIR, HOXC11, HOXC-AS3, HOXC10, MIR196A2, HOXC-AS2, HOXC-AS1, HOXC9, HOXC8, HOXC6, HOXC5, HOXC4, MIR615, FLJ12825, LOC100240735, LOC100240734, LOC400043, SMUG1, CBX5, MIR3198-2, HNRNPA1, HNRNPA1P10, NFE2, COPZ1, MIR148B, LOC102724050, GPR84, ZNF385A, ITGA5, GTSF1, NCKAP1L, PDE1B, PPP1R1A, GLYCAM1, LACRT, DCD, MUCL1, TESPA1, NEUROD4, OR9K2, OR10A7, OR6C74, OR6C6, OR6C1, OR6C3, OR6C75, OR6C65, OR6C76, OR6C2, OR6C70, OR6C68, OR6C4, OR2AP1, OR10P1, METTL7B, ITGA7, BLOC1S1, BLOC1S1-RDH5, RDH5, CD63, GDF11, SARNP, ORMDL2, DNAJC14, TMEM198B, MMP19, PYM1, DGKA, PMEL, CDK2, RAB5B, SUOX, LOC105369781, IKZF4, RPS26, ERBB3, PA2G4, RPL41, ZC3H10, ESYT1, MYL6B, MYL6, SMARCC2, RNF41, NABP2, SLC39A5, ANKRD52, COQ10A, CS, CNPY2, PAN2, IL23A, STAT2, APOF, TIMELESS, MIP, SPRYD4, GLS2, SNORA105C, RBMS2, BAZ2A, ATP5B, SNORD59B, SNORD59A, PTGES3, NACA, PRIM1, HSD17B6, SDR9C7, RDH16, GPR182, ZBTB39, TAC3, MYO1A, NEMP1, NAB2, STAT6, LRP1, LRP1-AS, MIR1228, NXPH4, SHMT2, NDUFA4L2, STAC3, R3HDM2, INHBC, INHBE, GLI1, ARHGAP9, MARS, MIR6758, DDIT3, MIR616, MBD6, DCTN2, KIF5A, PIP4K2C, DTX3, ARHGEF25, LOC101927583, SLC26A10, B4GALNT1, OS9, AGAP2, AGAP2-AS1, TSPAN31, CDK4, MIR6759, MARCH9, CYP27B1, METTL1, METTL21B, TSFM, AVIL, CTDSP2, MIR26A2, LOC100506844, ATP23, LOC105369785, LOC101927653, LOC100506869, LRIG3, SLC16A7, FAM19A2, USP15, MIR6125, MON2, LINC01465, MIRLET7I, PPM1H, AVPR1A, DPY19L2, TMEM5, TMEM5-AS1, SRGAP1, C12orf66, C12orf56, XPOT, TBK1, RASSF3, MIR548Z, MIR548C, GNS, TBC1D30, FLJ41278, WIF1, LEMD3, MSRB3, LOC100507065, LOC105369187, RPSAP52, HMGA2, LOC100129940, MIR6074, LLPH, LLPH-AS1, TMBIM4, IRAK3, MIR6502, HELB, GRIP1, LOC102724421, CAND1, LOC100507175, DYRK2, LOC101927901, LINC01479, IFNG-AS1, IFNG, IL26, IL22, MDM1, LOC100507195, RAP1B, SNORA70G, LOC100507250, NUP107, SLC35E3, LOC100130075, MDM2, CPM, CPSF6, MIR1279, LYZ, YEATS4, FRS2, MIR3913-1, MIR3913-2, CCT2, LRRC10, BEST3, LOC101928002, RAB3IP, MYRFL, LINC01481, CNOT2, KCNMB4, PTPRB, PTPRR, TSPAN8, LGR5, ZFC3H1, THAP2, TMEM19, RAB21, TBC1D15, MRS2P2, TPH2, TRHDE-AS1, TRHDE, LOC101928137, LOC100507377, ATXN7L3B, KCNC2, CAPS2, GLIPR1L1, GLIPR1L2, GLIPR1, KRR1, PHLDA1, NAP1L1, BBS10, OSBPL8, ZDHHC17, CSRP2, E2F7, NAV3, LOC105369860, SYT1, MIR1252, PAWR, PPP1R12A, OTOGL, PTPRQ, MYF6, MYF5, LINC01490, LIN7A, MIR617, MIR618, ACSS3, MIR4699, PPFIA2, LOC102724663, LOC101928449, CCDC59, METTL25, TMTC2, SLC6A15, TSPAN19, LRRIQ1, ALX1, RASSF9, NTS, MGAT4C, LOC105369879, MKRN9P, C12orf50, C12orf29, CEP290, TMTC3, KITLG, LOC728084, DUSP6, POC1B, GALNT4, POC1B-GALNT4, ATP2B1, LINC00936, LOC105369891, LOC105369893, LINC00615, CCER1, EPYC, KERA, LUM, DCN, LINC01619, BTG1, LOC101928617, CLLU1OS, CLLU1, C12orf74, PLEKHG7, EEA1, LOC643339, LOC102724933, NUDT4, NUDT4P2, NUDT4P1, UBE2N, MRPL42, SOCS2-AS1, SOCS2, CRADD, LOC101928731, LOC105369911, PLXNC1, CEP83, CEP83-AS1, MIR5700, TMCC3, MIR7844, MIR492, KRT19P2, NDUFA12, NR2C1, FGD6, VEZT, MIR331, MIR3685, METAP2, USP44, PGAM1P5, NTN4, LOC105369921, LOC105369920, SNRPF, CCDC38, AMDHD1, HAL, LTA4H, ELK3, CDK17, CFAP54, NEDD1, RMST, MIR1251, MIR135A2, LOC643711, MIR4495, MIR4303, SLC9A7P1, LOC643770, TMPO-AS1, TMPO, SLC25A3, SNORA53, IKBIP, APAF1, ANKS1B, LOC101928937, FAM71C, UHRF1BP1L, GOLGA2P5, MIR1827, ACTR6, DEPDC4, SCYL2, SLC17A8, NR1H4, GAS2L3, ANO4, SLC5A8, UTP20, ARL1, SPIC, MYBPC1, CHPT1, SYCP3, GNPTAB, DRAM1, CCDC53, NUP37, PARPBP, PMCH, IGF1, LINC00485, PAH, ASCL1, LOC101929058, C12orf42, LOC105369945, LOC101929084, STAB2, NT5DC3, TTC41P, HSP90B1, MIR3652, C12orf73, TDG, GLT8D2, HCFC2, NFYB, TXNRD1, EID3, CHST11, MIR3922, SLC41A2, C12orf45, ALDH1L2, LOC414300, KIAA1033, APPL2, KCCAT198, C12orf75, CASC18, NUAK1, CKAP4, TCP11L2, POLR3B, LOC100287944, RFX4, LOC100505978, RIC8B, TMEM263, MTERF2, CRY1, BTBD11, PWP1, PRDM4, LOC101929162, ASCL4, LOC728739, WSCD2, CMKLR1, LINC01498, FICD, SART3, ISCU, TMEM119, SELPLG, MIR4496, CORO1C, SSH1, MIR619, DAO, SVOP, USP30, USP30-AS1, ALKBH2, UNG, ACACB, FOXN4, LINC01486, MYO1H, KCTD10, UBE3B, MMAB, MVK, FAM222A, FAM222A-AS1, TRPV4, MIR4497, GLTP, TCHP, GIT2, ANKRD13A, C12orf76, IFT81, ATP2A2, ANAPC7, ARPC3, GPN3, FAM216A, VPS29, RAD9B, PPTC7, TCTN1, HVCN1, PPP1CC, CCDC63, MYL2, LINC01405, LOC105369980, CUX2, MIR6760, FAM109A, SH2B3, ATXN2, BRAP, ACAD10, ALDH2, MIR6761, MAPKAPK5-AS1, MAPKAPK5, ADAM1A, TMEM116, ERP29, NAA25, MIR3657, TRAFD1, HECTD4, MIR6861, RPL6, PTPN11, MIR1302-1, RPH3A, OAS1, OAS3, OAS2, DTX1, RASAL1, CFAP73, DDX54, MIR7106, RITA1, IQCD, TPCN1, MIR6762, SLC8B1, PLBD2, SDS, SDSL, LHX5, LHX5-AS1, LINC01234, RBM19, TBX5, TBX5-AS1, TBX3, MED13L, MIR620, MIR4472-2, LINC00173, MAP1LC3B2, C12orf49, RNFT2, HRK, FBXW8, LOC100506551, TESC, TESC-AS1, FBXO21, NOS1, KSR2, RFC5, WSB2, VSIG10, PEBP1, TAOK3, SUDS3, LOC105370014, LOC105370016, SRRM4, LOC105370024, HSPB8, LINC00934, CCDC60, TMEM233, PRKAB1, CIT, MIR1178, BICDL1, RAB35, GCN1, MIR4498, RPLP0, PXN-AS1, PXN, SIRT4, PLA2G1B, MSI1, COX6A1, TRIAP1, GATC, SRSF9, DYNLL1, NRAV, COQ5, RNF10, POP5, CABP1, MLEC, UNC119B, MIR4700, ACADS, SPPL3, XLOC_009911, HNF1A-AS1, HNF1A, C12orf43, OASL, P2RX7, P2RX4, CAMKK2, ANAPC5, RNF34, KDM2B, MIR7107, MIR548AQ, ORAI1, MORN3, TMEM120B, RHOF, LINC01089, SETD1B, HPD, PSMD9, WDR66, BCL7A, LOC100506691, MLXIP, LRRC43, IL31, B3GNT4, DIABLO, LOC101593348, VPS33A, CLIP1, CLIP1-AS1, ZCCHC8, RSRC2, KNTC1, HCAR2, HCAR3, HCAR1, DENR, CCDC62, HIP1R, VPS37B, ABCB9, OGFOD2, ARL6IP4, PITPNM2, MIR4304, LOC100507091, MPHOSPH9, C12orf65, CDK2AP1, SBNO1, MIR8072, KMT5A, RILPL2, SNRNP35, RILPL1, MIR3908, LOC101927415, TMED2, DDX55, EIF2B1, GTF2H3, TCTN2, ATP6V0A2, DNAH10, CCDC92, ZNF664, ZNF664-FAM101A, FAM101A, NCOR2, MIR6880, SCARB1, UBC, MIR5188, DHX37, BRI3BP, THRIL, AACS, TMEM132B, LINC00939, LOC101927464, LOC100128554, LOC100996671, LINC00944, LINC00943, LOC440117, LOC101927592, LOC101927616, LOC101927637, LOC105370068, FLJ37505, LINC00508, LINC00507, CRAT8, LOC100996679, LOC101927694, MIR4419B, TMEM132C, MIR3612, SLC15A4, GLT1D1, TMEM132D, LOC283352, LOC101927735, LOC100190940, FZD10-AS1, FZD10, PIWIL1, RIMBP2, STX2, RAN, ADGRD1, LACAT8, LINC01257, LOC107161159, LOC338797, SFSWAP, MMP17, ULK1, PUS1, EP400, SNORA49, EP400NL, DDX51, NOC4L, GALNT9, LOC100130238, LOC101928416, FBRSL1, MIR6763, LRCOL1, P2RX2, POLE, PXMP2, PGAM5, ANKLE2, GOLGA3, CHFR, LOC101928530, ZNF605, ZNF26, LOC101928597, ZNF84, ZNF140, ZNF891, ZNF10, ZNF268, ANHX | arr[GRCh37] 12p13.33q24.33(189399_133818115)x3 |
| 1  BM | Gain | 13 | q11 | q34 | 96,018 | LINC00417, ANKRD20A9P, LINC00408, LINC00442, TUBA3C, LOC101928697, ANKRD26P3, LINC00421, TPTE2, LINC00350, MPHOSPH8, PSPC1, ZMYM5, ZMYM2, LINC01072, GJA3, GJB2, GJB6, CRYL1, MIR4499, IFT88, IL17D, EEF1AKMT1, XPO4, LINC00367, LATS2, SAP18, SKA3, MRPL57, LINC01046, MIPEPP3, LINC00539, ZDHHC20, MICU2, FGF9, LINC00424, LINC00540, BASP1P1, SGCG, SACS, SACS-AS1, LINC00327, TNFRSF19, MIPEP, C1QTNF9B-AS1, C1QTNF9B, ANKRD20A19P, SPATA13, MIR2276, SPATA13-AS1, C1QTNF9, LINC00566, PARP4, TPTE2P6, ATP12A, RNF17, CENPJ, TPTE2P1, PABPC3, AMER2, LINC00463, LINC01053, MTMR6, NUP58, ATP8A2, SHISA2, RNF6, CDK8, WASF3, GPR12, USP12, USP12-AS1, USP12-AS2, LINC00412, RPL21, RPL21P28, SNORD102, SNORA27, RASL11A, GTF3A, MTIF3, LNX2, POLR1D, GSX1, PDX1-AS1, PDX1, ATP5EP2, LINC00543, CDX2, URAD, FLT3, PAN3-AS1, PAN3, FLT1, POMP, SLC46A3, MTUS2, MTUS2-AS1, SLC7A1, LOC102723345, UBL3, LINC00297, LINC00572, LINC00544, LINC00365, KATNAL1, LINC00426, LINC01058, HMGB1, USPL1, ALOX5AP, LINC00398, LINC00545, TEX26-AS1, MEDAG, TEX26, HSPH1, B3GLCT, RXFP2, EEF1DP3, FRY-AS1, FRY, ZAR1L, BRCA2, N4BP2L1, N4BP2L2, MINOS1P1, N4BP2L2-IT2, PDS5B, LINC00423, KL, STARD13, STARD13-AS, RFC3, LINC00457, NBEA, MAB21L1, LINC00445, DCLK1, SOHLH2, CCDC169-SOHLH2, CCDC169, SPG20, SPG20-AS1, CCNA1, SERTM1, RFXAP, SMAD9, ALG5, EXOSC8, SUPT20H, CSNK1A1L, LINC01048, LINC00547, POSTN, TRPC4, LINC00571, UFM1, LINC00437, LINC00366, FREM2, STOML3, PROSER1, NHLRC3, LHFP, COG6, MIR4305, LINC00332, LINC00548, LINC00598, FOXO1, MIR320D1, MRPS31, SLC25A15, TPTE2P5, MIR621, SUGT1P3, ELF1, WBP4, MIR3168, KBTBD6, LOC101929140, KBTBD7, MTRF1, NAA16, OR7E37P, RGCC, VWA8, MIR5006, VWA8-AS1, DGKH, AKAP11, LOC105370177, TNFSF11, FAM216B, LINC01050, LINC00428, EPSTI1, DNAJC15, LINC00400, ENOX1, ENOX1-AS2, CCDC122, LACC1, LINC00284, LINC00390, SMIM2-AS1, SMIM2, SMIM2-IT1, MIR8079, SERP2, TUSC8, TSC22D1, TSC22D1-AS1, LINC00330, NUFIP1, GPALPP1, LOC101929259, GTF2F2, KCTD4, TPT1, SNORA31, TPT1-AS1, SLC25A30, SLC25A30-AS1, COG3, ERICH6B, LINC01055, SPERT, SIAH3, ZC3H13, CPB2-AS1, CPB2, LCP1, LRRC63, LINC00563, KIAA0226L, LINC01198, LRCH1, ESD, HTR2A, HTR2A-AS1, LINC00562, SUCLA2, NUDT15, MED4, MED4-AS1, ITM2B, LINC00441, RB1, LPAR6, RCBTB2, LINC00462, CYSLTR2, FNDC3A, MLNR, LOC105370203, CDADC1, CAB39L, SETDB2, SETDB2-PHF11, PHF11, RCBTB1, ARL11, EBPL, KPNA3, CTAGE10P, SPRYD7, DLEU2, MIR3613, TRIM13, KCNRG, MIR16-1, MIR15A, DLEU1, ST13P4, DLEU1-AS1, DLEU7, DLEU7-AS1, RNASEH2B-AS1, RNASEH2B, GUCY1B2, LINC00371, FAM124A, SERPINE3, MIR5693, INTS6, INTS6-AS1, MIR4703, WDFY2, DHRS12, LINC00282, CCDC70, ATP7B, ALG11, UTP14C, NEK5, LOC101929657, NEK3, MRPS31P5, LOC103191607, THSD1, VPS36, CKAP2, TPTE2P3, HNRNPA1L2, SUGT1, LECT1, MIR759, PCDH8, OLFM4, LINC01065, LINC00558, LINC00458, MIR1297, MIR5007, PRR20A, PRR20D, PRR20E, PRR20B, PRR20C, PCDH17, LOC101926897, LINC00374, DIAPH3, DIAPH3-AS1, DIAPH3-AS2, LINC00434, TDRD3, LINC00378, MIR3169, PCDH20, LOC101926951, LINC00358, LINC01075, LINC00448, LINC00376, LINC00395, OR7E156P, LOC102723968, LINC01052, MIR548X2, MIR4704, PCDH9, PCDH9-AS2, PCDH9-AS3, PCDH9-AS4, LINC00364, LINC00550, LINC00383, KLHL1, ATXN8OS, LINC00348, DACH1, MZT1, BORA, DIS3, PIBF1, KLF5, LINC00392, KLF12, LINC00381, LINC00347, CTAGE11P, LINC01078, TBC1D4, COMMD6, UCHL3, LMO7-AS1, LMO7, LMO7DN, LMO7DN-IT1, KCTD12, BTF3P11, ACOD1, CLN5, FBXL3, MYCBP2, MYCBP2-AS1, SCEL, SCEL-AS1, LOC100129307, SLAIN1, MIR3665, EDNRB-AS1, EDNRB, LINC01069, LINC00446, RNF219-AS1, POU4F1, RNF219, LINC00331, RBM26, RBM26-AS1, NDFIP2-AS1, NDFIP2, LINC01068, LINC01038, LINC00382, LINC01080, SPRY2, LINC00377, LINC00564, SLITRK1, LINC00333, SNORA107, LINC00375, LINC00351, SLITRK6, LINC00430, MIR4500HG, MIR4500, SLITRK5, LINC00397, LOC105370306, LINC00433, LINC01047, LINC00440, LINC01040, LINC00353, LINC00559, MIR622, LINC01049, LINC00410, LINC00380, LINC00379, MIR17HG, MIR17, MIR18A, MIR19A, MIR20A, MIR19B1, MIR92A1, GPC5, GPC5-AS2, MIR548AS, GPC5-AS1, LINC00363, GPC6, GPC6-AS2, GPC6-AS1, DCT, TGDS, GPR180, LOC101927248, SOX21, SOX21-AS1, LOC101927284, LINC00557, ABCC4, CLDN10, CLDN10-AS1, DZIP1, DNAJC3-AS1, DNAJC3, UGGT2, HS6ST3, MIR4501, LINC00359, OXGR1, LINC00456, MBNL2, RAP2A, IPO5, FARP1, RNF113B, MIR3170, STK24, SLC15A1, DOCK9, DOCK9-AS1, DOCK9-AS2, UBAC2-AS1, UBAC2, GPR18, GPR183, FKSG29, MIR623, LINC01232, LINC00449, TM9SF2, LINC01039, CLYBL, MIR4306, CLYBL-AS2, CLYBL-AS1, LOC101927437, ZIC5, ZIC2, LINC00554, LOC105370333, PCCA, PCCA-AS1, GGACT, TMTC4, NALCN-AS1, LINC00411, NALCN, ITGBL1, FGF14, MIR2681, MIR4705, FGF14-IT1, FGF14-AS1, FGF14-AS2, TPP2, METTL21C, CCDC168, TEX30, KDELC1, BIVM, BIVM-ERCC5, ERCC5, METTL21EP, SLC10A2, LINC01309, DAOA-AS1, DAOA, LINC00343, LINC00460, EFNB2, ARGLU1, LINC00551, LINC00443, FAM155A, MIR1267, FAM155A-IT1, LIG4, ABHD13, TNFSF13B, MYO16, MYO16-AS1, LINC00399, LINC00676, IRS2, LINC00396, COL4A1, COL4A2, MIR8073, COL4A2-AS2, COL4A2-AS1, RAB20, NAXD, CARS2, ING1, LINC00567, LINC00346, ANKRD10, LINC00431, LINC00368, ARHGEF7-AS2, ARHGEF7, ARHGEF7-AS1, LOC101060553, TEX29, LOC105370369, LINC00354, LINC00403, SOX1, LOC100506016, LINC01070, LOC101928730, LINC01043, LINC01044, SPACA7, TUBGCP3, ATP11AUN, ATP11A, ATP11A-AS1, MCF2L-AS1, MCF2L, F7, F10, F10-AS1, PROZ, PCID2, CUL4A, MIR8075, LAMP1, GRTP1, GRTP1-AS1, LOC101928841, ADPRHL1, DCUN1D2, TMCO3, TFDP1, ATP4B, GRK1, LINC00552, TMEM255B, GAS6-AS1, GAS6, GAS6-AS2, LINC00452, LINC00565, RASA3, CDC16, MIR548AR, MIR4502, UPF3A, CHAMP1, LINC01054 | arr[GRCh37] 13q11q34(19084822_115103150)x3 |
| 1  BM | Gain | 14 | q12 | q13.1 | 5,767 | FOXG1-AS1, FOXG1, LINC01551, LOC105370424, PRKD1, G2E3, SCFD1, COCH, LOC100506071, STRN3, MIR624, AP4S1, HECTD1, HEATR5A, LOC101927124, DTD2, GPR33, NUBPL, ARHGAP5-AS1, ARHGAP5, RNU6-2, AKAP6, NPAS3, SNORA89, EGLN3, SPTSSA | arr[GRCh37] 14q12q13.1(29166323_34933810)x3 |
| 1  BM | Gain | 14 | q32.12 | q32.33 | 14,439 | FBLN5, TRIP11, ATXN3, NDUFB1, CPSF2, SLC24A4, RIN3, LGMN, GOLGA5, LOC101929002, CHGA, ITPK1, ITPK1-AS1, MOAP1, TMEM251, C14orf142, UBR7, BTBD7, UNC79, COX8C, PRIMA1, FAM181A-AS1, FAM181A, ASB2, MIR4506, LINC00521, OTUB2, DDX24, IFI27L1, IFI27, IFI27L2, PPP4R4, SERPINA10, SERPINA6, SERPINA2, SERPINA1, SERPINA11, SERPINA9, SERPINA12, SERPINA4, SERPINA5, SERPINA3, SERPINA13P, GSC, DICER1, MIR3173, DICER1-AS1, CLMN, LOC101929080, LINC00341, SYNE3, SNHG10, SCARNA13, GLRX5, TCL6, TCL1B, TCL1A, TUNAR, C14orf132, BDKRB2, BDKRB1, ATG2B, GSKIP, AK7, LOC730202, PAPOLA, VRK1, LINC00618, LOC101929241, LOC100129345, LINC01550, C14orf177, BCL11B, SETD3, CCNK, CCDC85C, HHIPL1, CYP46A1, EML1, EVL, MIR151B, MIR342, DEGS2, YY1, MIR6764, SLC25A29, MIR345, SLC25A47, WARS, WDR25, BEGAIN, LINC00523, DLK1, MIR2392, MEG3, MIR770, MIR493, MIR337, MIR665, RTL1, MIR431, MIR433, MIR127, MIR432, MIR136, MEG8, MIR370, SNORD113-1, SNORD113-2, SNORD113-4, SNORD113-5, SNORD113-6, SNORD113-7, SNORD113-9, SNORD114-1, SNORD114-2, SNORD114-3, SNORD114-4, SNORD114-5, SNORD114-6, SNORD114-7, SNORD114-8, SNORD114-9, SNORD114-10, SNHG24, SNORD114-11, SNORD114-12, SNORD114-13, SNORD114-14, SNORD114-15, SNORD114-16, SNORD114-17, SNORD114-18, SNORD114-19, SNORD114-20, SNORD114-21, SNORD114-22, SNORD114-23, SNORD114-24, SNORD114-25, SNORD114-26, SNORD114-27, SNORD114-28, SNORD114-29, SNORD114-30, SNORD114-31, MIR379, MIR411, MIR299, MIR380, MIR1197, MIR323A, MIR758, MIR329-1, MIR329-2, MIR494, MIR1193, MIR543, MIR495, MIR376C, MIR376A2, MIR654, MIR376B, MIR376A1, MIR300, MIR1185-1, MIR1185-2, MIR381HG, MIR381, MIR487B, MIR539, MIR889, MIR544A, MIR655, MIR487A, MIR382, MIR134, MIR668, MIR485, MIR323B, MIR154, MIR496, MIR377, MIR541, MIR409, MIR412, MIR369, MIR410, MIR656, MEG9, LINC00524, LOC100507277, DIO3OS, MIR1247, DIO3, LINC00239, PPP2R5C, DYNC1H1, HSP90AA1, WDR20, MOK, ZNF839, CINP, TECPR2, ANKRD9, MIR4309, RCOR1, TRAF3, AMN, CDC42BPB, EXOC3L4, TNFAIP2, LINC00605, LOC105378183, EIF5, SNORA28, MARK3, CKB, TRMT61A, BAG5, APOPT1, KLC1, XRCC3, ZFYVE21, PPP1R13B, LINC00637, C14orf2, TDRD9, RD3L, ASPG, MIR203A, MIR203B, KIF26A, C14orf180, TMEM179, LOC101929634, MIR4710, INF2, ADSSL1, SIVA1, AKT1, ZBTB42, LINC00638, CEP170B, PLD4, AHNAK2, C14orf79, CDCA4, GPR132, LOC102723354, JAG2, MIR6765, NUDT14, BRF1, BTBD6, PACS2, TEX22, LOC100507437, MTA1, CRIP2, CRIP1, C14orf80, TMEM121, LOC105370697, MIR8071-1, MIR8071-2, ELK2AP, MIR4507, MIR4538, MIR4537, MIR4539, KIAA0125, ADAM6, LINC00226 | arr[GRCh37] 14q32.12q32.33(92389046_106827735)x3 |
| 1  BM | Gain | 15 | q11.1 | q26.3 | 82,236 | CHEK2P2, HERC2P3, GOLGA6L6, GOLGA8CP, NBEAP1, MIR3118-3, MIR3118-4, MIR3118-2, POTEB2, POTEB, POTEB3, NF1P2, MIR5701-3, MIR5701-2, MIR5701-1, LINC01193, LOC646214, CXADRP2, LOC101927079, LOC727924, OR4M2, OR4N4, OR4N3P, LOC102724760, IGHV1OR15-1, LOC642131, IGHV1OR15-3, MIR1268A, REREP3, MIR4509-3, MIR4509-1, MIR4509-2, GOLGA8DP, GOLGA6L1, GOLGA6L22, TUBGCP5, CYFIP1, NIPA2, NIPA1, LOC283683, WHAMMP3, GOLGA8IP, HERC2P2, HERC2P7, GOLGA8EP, GOLGA8S, GOLGA6L2, MIR4508, MKRN3, MAGEL2, NDN, PWRN4, PWRN2, PWRN3, PWRN1, NPAP1, SNRPN, SNURF, SNORD107, PWARSN, PWAR5, SNORD64, SNORD108, SNORD109B, SNORD109A, SNORD116-1, SNORD116-2, SNORD116-3, SNORD116-9, SNORD116-4, SNORD116-7, SNORD116-5, SNORD116-6, SNORD116-8, SNORD116-10, SNORD116-11, SNORD116-12, SNORD116-13, SNORD116-14, SNORD116-15, SNORD116-16, SNORD116-17, SNORD116-19, SNORD116-18, SNORD116-20, SNORD116-21, SNORD116-22, SNORD116-23, SNORD116-24, SNORD116-25, SNORD116-26, SNORD116-27, SNORD116-28, SNORD116-29, SNORD116-30, IPW, PWAR1, SNORD115-1, SNORD115-2, SNORD115-3, SNORD115-4, SNORD115-5, SNORD115-9, SNORD115-12, SNORD115-10, SNORD115-6, SNORD115-7, SNORD115-8, SNORD115-43, SNORD115-11, SNORD115-29, SNORD115-36, SNORD115-13, SNORD115-14, SNORD115-16, SNORD115-19, SNORD115-18, SNORD115-17, SNORD115-20, SNORD115-21, SNORD115-15, SNORD115-22, PWAR4, SNORD115-23, SNORD115-24, SNORD115-25, SNORD115-26, SNORD115-27, SNORD115-28, SNORD115-30, SNORD115-31, SNORD115-32, SNORD115-33, SNORD115-34, SNORD115-35, SNORD115-37, SNORD115-38, SNORD115-39, SNORD115-40, SNORD115-41, SNORD115-42, SNORD115-44, SNORD115-45, SNORD115-46, SNORD115-47, SNORD115-48, UBE3A, ATP10A, MIR4715, LOC100128714, LINC00929, GABRB3, GABRA5, GABRG3, GABRG3-AS1, OCA2, HERC2, GOLGA8F, GOLGA8G, HERC2P9, GOLGA8M, WHAMMP2, LOC100289656, PDCD6IPP2, GOLGA6L7P, APBA2, FAM189A1, NSMCE3, LOC100130111, TJP1, GOLGA8J, ULK4P3, GOLGA8T, DKFZP434L187, CHRFAM7A, GOLGA8R, ULK4P1, ULK4P2, GOLGA8H, ARHGAP11B, LOC100288637, HERC2P10, FAN1, MTMR10, TRPM1, MIR211, LOC102725022, LOC283710, KLF13, OTUD7A, CHRNA7, GOLGA8K, GOLGA8O, WHAMMP1, LOC100996255, LOC101928042, GOLGA8N, ARHGAP11A, SCG5, LOC105370757, GREM1, LOC100131315, FMN1, TMCO5B, LOC101928134, RYR3, AVEN, CHRM5, EMC7, PGBD4, KATNBL1, EMC4, SLC12A6, NOP10, NUTM1, LPCAT4, GOLGA8A, MIR1233-2, MIR1233-1, GOLGA8B, GJD2, LOC101928174, ACTC1, AQR, ZNF770, ANP32AP1, DPH6, MIR3942, DPH6-AS1, MIR4510, C15orf41, CSNK1A1P1, LOC145845, MEIS2, MIR8063, TMCO5A, LOC101928227, SPRED1, FAM98B, RASGRP1, C15orf53, C15orf54, THBS1, FSIP1, LOC105370941, GPR176, EIF2AK4, SRP14, SRP14-AS1, BMF, BUB1B, BUB1B-PAK6, PAK6, C15orf56, ANKRD63, PLCB2, INAFM2, C15orf52, PHGR1, DISP2, KNSTRN, IVD, BAHD1, CHST14, C15orf57, MRPL42P5, RPUSD2, KNL1, RAD51-AS1, RAD51, RMDN3, GCHFR, DNAJC17, C15orf62, ZFYVE19, PPP1R14D, SPINT1, RHOV, VPS18, LOC105370943, DLL4, CHAC1, INO80, EXD1, CHP1, OIP5-AS1, OIP5, NUSAP1, NDUFAF1, RTF1, ITPKA, LTK, RPAP1, TYRO3, MGA, MIR626, MAPKBP1, JMJD7, JMJD7-PLA2G4B, PLA2G4B, SPTBN5, MIR4310, LOC105370792, EHD4, EHD4-AS1, PLA2G4E-AS1, PLA2G4E, PLA2G4D, PLA2G4F, VPS39, MIR627, TMEM87A, GANC, CAPN3, ZNF106, SNAP23, LRRC57, HAUS2, STARD9, CDAN1, TTBK2, UBR1, TMEM62, CCNDBP1, EPB42, TGM5, TGM7, LCMT2, ADAL, ZSCAN29, TUBGCP4, TP53BP1, MAP1A, PPIP5K1, CKMT1B, STRC, CATSPER2, CKMT1A, CATSPER2P1, PDIA3, ELL3, SERF2, SERF2-C15ORF63, MIR1282, SERINC4, HYPK, MFAP1, WDR76, FRMD5, PIN4P1, CASC4, CTDSPL2, EIF3J-AS1, EIF3J, SPG11, PATL2, B2M, LOC100419583, TRIM69, TERB2, SORD, DUOX2, DUOXA2, DUOXA1, DUOX1, SHF, LOC101928414, SLC28A2, GATM, SPATA5L1, C15orf48, MIR147B, SLC30A4, HMGN2P46, BLOC1S6, SQRDL, LOC105370802, SEMA6D, LINC01491, SLC24A5, MYEF2, CTXN2, SLC12A1, DUT, FBN1, CEP152, SHC4, EID1, SECISBP2L, COPS2, GALK2, NDUFAF4P1, MIR4716, FAM227B, FGF7, DTWD1, ATP8B4, SLC27A2, HDC, GABPB1, FLJ10038, GABPB1-AS1, MIR4712, USP8, USP50, TRPM7, SPPL2A, AP4E1, DCAF13P3, TNFAIP8L3, CYP19A1, MIR4713, MIR7973-2, MIR7973-1, GLDN, DMXL2, SCG3, LYSMD2, TMOD2, TMOD3, LOC100422556, LEO1, MAPK6, BCL2L10, GNB5, LOC100129973, MYO5C, MIR1266, MYO5A, ARPP19, FAM214A, ONECUT1, WDR72, UNC13C, LOC105370829, RSL24D1, RAB27A, PIGBOS1, PIGB, CCPG1, DYX1C1-CCPG1, MIR628, C15orf65, DYX1C1, PYGO1, PRTG, NEDD4, RFX7, TEX9, MNS1, ZNF280D, LOC145783, TCF12, LINC00926, LINC01413, CGNL1, MYZAP, GCOM1, POLR2M, ALDH1A2, AQP9, LIPC, LIPC-AS1, ADAM10, HSP90AB4P, LOC101928725, FAM63B, SLTM, RNF111, CCNB2, MYO1E, MIR2116, LDHAL6B, FAM81A, GCNT3, GTF2A2, BNIP2, FOXB1, ANXA2, ICE2, RORA-AS1, RORA, RORA-AS2, VPS13C, LOC101928907, C2CD4A, C2CD4B, GOLGA2P11, MIR8067, MIR6085, MGC15885, TLN2, MIR190A, TPM1, LACTB, RPS27L, RAB8B, APH1B, CA12, LOC102723344, USP3, USP3-AS1, FBXL22, HERC1, MIR422A, DAPK2, LOC101928988, FAM96A, SNX1, SNX22, PPIB, CSNK1G1, KIAA0101, TRIP4, ZNF609, OAZ2, RBPMS2, MIR1272, PIF1, PLEKHO2, ANKDD1A, SPG21, MTFMT, SLC51B, RASL12, KBTBD13, UBAP1L, PDCD7, CLPX, CILP, PARP16, IGDCC3, IGDCC4, DPP8, HACD3, VWA9, SLC24A1, DENND4A, MIR4511, RAB11A, MEGF11, MIR4311, DIS3L, TIPIN, SCARNA14, MAP2K1, SNAPC5, MIR4512, RPL4, SNORD18C, SNORD18B, SNORD16, SNORD18A, ZWILCH, LCTL, LINC01169, SMAD6, LOC102723481, LOC102723493, SMAD3, AAGAB, IQCH, IQCH-AS1, C15orf61, MAP2K5, SKOR1, LOC101929076, RNU6-2, PIAS1, CALML4, CLN6, FEM1B, ITGA11, CORO2B, ANP32A, MIR4312, ANP32A-IT1, MIR548H4, SPESP1, NOX5, EWSAT1, GLCE, PAQR5, LOC145694, KIF23, RPLP1, DRAIC, PCAT29, LINC00593, TLE3, MIR629, LOC101929151, SALRNA3, SALRNA2, UACA, LARP6, LRRC49, THAP10, CT62, THSD4, THSD4-AS1, THSD4-AS2, NR2E3, MYO9A, SENP8, GRAMD2, PKM, PARP6, CELF6, HEXA, HEXA-AS1, TMEM202, LOC105370888, ARIH1, MIR630, LOC102723640, GOLGA6B, HIGD2B, BBS4, ADPGK, ADPGK-AS1, NEO1, HCN4, REC114, NPTN, NPTN-IT1, CD276, C15orf59, C15orf59-AS1, TBC1D21, LOXL1-AS1, LOXL1, STOML1, PML, GOLGA6A, LOC283731, ISLR2, ISLR, STRA6, CCDC33, CYP11A1, LOC729739, SEMA7A, MIR6881, UBL7, UBL7-AS1, ARID3B, CLK3, EDC3, CYP1A1, CYP1A2, CSK, MIR4513, LMAN1L, CPLX3, ULK3, MIR6882, SCAMP2, MPI, FAM219B, COX5A, RPP25, SCAMP5, PPCDC, C15orf39, LOC105376731, GOLGA6C, GOLGA6D, COMMD4, NEIL1, MIR631, MAN2C1, SIN3A, PTPN9, SNUPN, IMP3, SNX33, CSPG4, ODF3L1, DNM1P35, MIR4313, UBE2Q2, FBXO22, FBXO22-AS1, NRG4, TMEM266, LOC101929439, ETFA, TYRO3P, ISL2, SCAPER, MIR3713, RCN2, PSTPIP1, TSPAN3, PEAK1, LINC00597, HMG20A, LOC101929457, LINGO1, LINGO1-AS1, LINGO1-AS2, LOC645752, LOC91450, TBC1D2B, SH2D7, CIB2, IDH3A, ACSBG1, DNAJA4, WDR61, CRABP1, IREB2, HYKK, PSMA4, CHRNA5, CHRNA3, CHRNB4, LOC646938, ADAMTS7, MORF4L1, CTSH, RASGRF1, ANKRD34C-AS1, MIR184, ANKRD34C, TMED3, KIAA1024, MTHFS, ST20-MTHFS, ST20, ST20-AS1, BCL2A1, ZFAND6, FAH, LINC01314, LINC00927, ARNT2, LOC101929586, MIR5572, ABHD17C, CEMIP, MIR549A, MESDC2, MIR4514, MESDC1, CFAP161, IL16, STARD5, TMC3-AS1, TMC3, MEX3B, LINC01583, EFL1, SAXO2, ADAMTS7P1, GOLGA6L10, UBE2Q2P2, GOLGA6L9, GOLGA2P10, GOLGA6L17P, RPS17, LOC727751, LOC102724034, CPEB1, CPEB1-AS1, AP3B2, LOC338963, ACTG1P17, SNHG21, FSD2, SCARNA15, WHAMM, HOMER2, FAM103A1, C15orf40, BTBD1, MIR4515, TM6SF1, HDGFRP3, BNC1, SH3GL3, ADAMTSL3, EFTUD1P1, UBE2Q2L, LOC440300, GOLGA2P7, LOC642423, GOLGA6L4, LOC103171574, DNM1P41, GOLGA6L5P, UBE2Q2P1, LINC00933, ZSCAN2, SCAND2P, WDR73, NMB, SEC11A, ZNF592, ALPK3, SLC28A1, PDE8A, GOLGA6L3, MIR7706, AKAP13, LOC101929679, KLHL25, MIR1276, MIR548AP, AGBL1, LINC01584, AGBL1-AS1, LOC105370954, LOC102724452, LINC00052, NTRK3, NTRK3-AS1, MRPL46, MRPS11, DET1, LINC01586, MIR1179, MIR7-2, MIR3529, AEN, ISG20, ACAN, HAPLN3, MFGE8, ABHD2, RLBP1, FANCI, POLG, MIR6766, MIR9-3HG, MIR9-3, RHCG, LINC00928, TICRR, KIF7, PLIN1, PEX11A, WDR93, MESP1, MESP2, ANPEP, AP3S2, C15orf38-AP3S2, MIR5094, MIR5009, ARPIN, ZNF710, MIR3174, IDH2, SEMA4B, CIB1, GDPGP1, TTLL13P, NGRN, GABARAPL3, ZNF774, IQGAP1, CRTC3, CRTC3-AS1, LINC01585, BLM, FURIN, FES, MAN2A2, UNC45A, HDDC3, RCCD1, PRC1, PRC1-AS1, VPS33B, LOC101926911, SV2B, CRAT37, SLCO3A1, ST8SIA2, LOC104613533, C15orf32, LINC00930, FAM174B, ASB9P1, LINC01578, CHD2, MIR3175, RGMA, LOC101927153, LINC01580, LINC01581, MCTP2, LOC440311, LINC01197, LINC00924, NR2F2-AS1, NR2F2, MIR1469, SPATA8-AS1, SPATA8, LOC101927286, LOC101927310, LINC00923, ARRDC4, LINC01582, FAM169B, IRAIN, IGF1R, MIR4714, PGPEP1L, LUNAR1, SYNM, TTC23, LRRC28, HSP90B2P, MEF2A, LYSMD4, DNM1P46, LOC400464, ADAMTS17, SPATA41, CERS3-AS1, CERS3, PRKXP1, LOC102723335, LINS1, ASB7, ALDH1A3, LOC101927751, LRRK1, CHSY1, VIMP, SNRPA1, PCSK6, LOC100507472, PCSK6-AS1, TM2D3, TARSL2, LOC100128108, OR4F6, OR4F15, OR4F13P | arr[GRCh37] 15q11.1q26.3(20161371_102397317)x3 |
| 1  BM | Gain | 16 | p12.2 | q24.3 | 66,754 | COG7, GGA2, EARS2, UBFD1, NDUFAB1, PALB2, DCTN5, PLK1, ERN2, CHP2, PRKCB, MIR1273H, CACNG3, RBBP6, LINC01567, TNRC6A, SLC5A11, ARHGAP17, LOC554206, LOC283887, LCMT1-AS1, LCMT1, LCMT1-AS2, AQP8, ZKSCAN2, HS3ST4, MIR548W, C16orf82, KDM8, NSMCE1, FLJ21408, IL4R, IL21R, IL21R-AS1, GTF3C1, KIAA0556, GSG1L, XPO6, SBK1, NPIPB6, EIF3CL, EIF3C, MIR6862-1, MIR6862-2, CLN3, APOBR, IL27, NUPR1, SGF29, SULT1A2, SULT1A1, NPIPB8, NPIPB9, ATXN2L, TUFM, MIR4721, SH2B1, ATP2A1, ATP2A1-AS1, RABEP2, CD19, NFATC2IP, MIR4517, SPNS1, LAT, RRN3P2, SNX29P2, NPIPB11, SMG1P6, BOLA2-SMG1P6, LOC606724, BOLA2, BOLA2B, SLX1B, SLX1A, SLX1B-SULT1A4, SLX1A-SULT1A3, SULT1A4, SULT1A3, LOC388242, LOC613038, SMG1P2, MIR3680-1, MIR3680-2, SPN, QPRT, C16orf54, ZG16, KIF22, MAZ, PRRT2, PAGR1, MVP, CDIPT, CDIPT-AS1, SEZ6L2, ASPHD1, KCTD13, TMEM219, TAOK2, HIRIP3, INO80E, DOC2A, C16orf92, FAM57B, ALDOA, PPP4C, TBX6, YPEL3, LOC101928595, GDPD3, MAPK3, CORO1A, LOC613037, SMG1P5, CD2BP2, TBC1D10B, MYLPF, SEPT1, ZNF48, ZNF771, DCTPP1, SEPHS2, ITGAL, MIR4518, ZNF768, ZNF747, ZNF764, ZNF688, ZNF785, ZNF689, PRR14, FBRS, LOC730183, SRCAP, SNORA30, TMEM265, PHKG2, CCDC189, RNF40, ZNF629, BCL7C, MIR4519, MIR762HG, MIR762, CTF1, FBXL19-AS1, FBXL19, ORAI3, SETD1A, HSD3B7, STX1B, STX4, ZNF668, ZNF646, PRSS53, VKORC1, BCKDK, KAT8, PRSS8, PRSS36, FUS, PYCARD, PYCARD-AS1, TRIM72, PYDC1, ITGAM, ITGAX, ITGAD, COX6A2, ZNF843, ARMC5, TGFB1I1, SLC5A2, C16orf58, AHSP, FRG2KP, YBX3P1, CLUHP3, ZNF720, ZNF267, LOC102723753, HERC2P4, TP53TG3D, LOC390705, TP53TG3, TP53TG3B, TP53TG3C, SLC6A10P, ENPP7P13, LINC00273, UBE2MP1, LINC01566, FRG2DP, TP53TG3HP, FLJ26245, ANKRD26P1, SHCBP1, VPS35, ORC6, MYLK3, C16orf87, GPT2, DNAJA2, NETO2, ITFG1-AS1, ITFG1, PHKB, LOC100507534, LOC101927132, ABCC12, ABCC11, LONP2, MIR5095, LOC100507577, SIAH1, N4BP1, CBLN1, C16orf78, ZNF423, CNEP1R1, HEATR3, PAPD5, ADCY7, MIR6771, BRD7, NKD1, SNX20, LOC101927272, NOD2, CYLD, MIR3181, LOC101927334, SALL1, LINC01571, C16orf97, LINC00919, LOC102467079, CASC22, TOX3, CASC16, LOC105371267, CHD9, LOC643802, LOC102723373, RBL2, AKTIP, RPGRIP1L, FTO, FTO-IT1, LOC100996338, IRX3, LOC100996345, LOC101927480, CRNDE, IRX5, IRX6, MMP2, LPCAT2, CAPNS2, SLC6A2, CES1P2, CES1P1, CES1, CES5A, LOC283856, GNAO1, DKFZP434H168, MIR3935, AMFR, NUDT21, OGFOD1, BBS2, MT4, MT3, MT2A, MT1L, MT1E, MT1M, MT1JP, MT1A, MT1DP, MT1B, MT1F, MT1G, MT1H, MT1IP, MT1X, NUP93, MIR138-2, SLC12A3, MIR6863, HERPUD1, CETP, NLRC5, CPNE2, FAM192A, RSPRY1, ARL2BP, PLLP, CCL22, CX3CL1, CCL17, CIAPIN1, COQ9, POLR2C, DOK4, CCDC102A, ADGRG5, ADGRG1, ADGRG3, DRC7, KATNB1, KIFC3, MIR6772, LOC388282, CNGB1, TEPP, ZNF319, USB1, MMP15, CFAP20, CSNK2A2, CCDC113, PRSS54, GINS3, NDRG4, SETD6, CNOT1, SNORA46, SNORA50A, SLC38A7, GOT2, APOOP5, LOC101927580, LOC729159, MIR4426, CDH8, CDH11, LOC101927650, LINC00922, CDH5, LINC00920, BEAN1, BEAN1-AS1, TK2, CKLF, CKLF-CMTM1, CMTM1, CMTM2, CMTM3, CMTM4, DYNC1LI2, LOC106699570, TERB1, NAE1, CA7, PDP2, CDH16, RRAD, FAM96B, CES2, CES3, CES4A, CBFB, C16orf70, B3GNT9, TRADD, FBXL8, HSF4, NOL3, KIAA0895L, EXOC3L1, E2F4, ELMO3, MIR328, LRRC29, TMEM208, FHOD1, SLC9A5, PLEKHG4, KCTD19, LRRC36, TPPP3, ZDHHC1, HSD11B2, ATP6V0D1, AGRP, LOC100505942, FAM65A, CTCF, CARMIL2, ACD, PARD6A, ENKD1, C16orf86, GFOD2, RANBP10, TSNAXIP1, CENPT, THAP11, NUTF2, EDC4, NRN1L, PSKH1, CTRL, PSMB10, LCAT, SLC12A4, DPEP3, DPEP2, LOC100131303, DDX28, DUS2, NFATC3, ESRP2, MIR6773, PLA2G15, SLC7A6, SLC7A6OS, PRMT7, SMPD3, ZFP90, CDH3, CDH1, MIR7641-2, TANGO6, HAS3, CHTF8, UTP4, SNTB2, VPS4A, PDF, COG8, NIP7, TMED6, TERF2, CYB5B, MIR1538, NFAT5, NQO1, NOB1, WWP2, MIR140, CLEC18A, PDXDC2P, MIR1972-2, MIR1972-1, PDPR, LOC400541, CLEC18C, LOC105371328, SMG1P7, EXOSC6, AARS, DDX19B, LOC100506083, DDX19A, ST3GAL2, FUK, COG4, SF3B3, SNORD111B, SNORD111, IL34, MTSS1L, VAC14, VAC14-AS1, HYDIN, CMTR2, CALB2, ZNF23, ZNF19, LOC105371335, CHST4, TAT-AS1, TAT, MARVELD3, PHLPP2, SNORA70D, AP1G1, SNORD71, ATXN1L, ZNF821, IST1, PKD1L3, DHODH, HP, HPR, TXNL4B, DHX38, PMFBP1, LINC01572, ZFHX3, HCCAT5, C16orf47, LINC01568, LOC101928035, PSMD7, LOC283922, NPIPB15, LOC105376772, CLEC18B, GLG1, RFWD3, MLKL, FA2H, WDR59, ZNRF1, LDHD, ZFP1, CTRB2, CTRB1, LOC100506281, BCAR1, CFDP1, TMEM170A, CHST6, CHST5, TMEM231, GABARAPL2, ADAT1, KARS, TERF2IP, CNTNAP4, LOC101928203, MIR4719, MON1B, SYCE1L, ADAMTS18, NUDT7, VAT1L, CLEC3A, WWOX, MAF, MAFTRR, LINC01229, LOC102724084, DYNLRB2, LINC01227, CDYL2, PRCAT47, CMC2, CENPN, ATMIN, C16orf46, GCSH, PKD1L2, BCO1, GAN, MIR4720, CMIP, MIR7854, MIR6504, LOC100129617, PLCG2, SDR42E1, HSD17B2, MPHOSPH6, CDH13, MIR8058, LOC101928446, LOC101928417, MIR3182, LOC102724163, HSBP1, MLYCD, OSGIN1, NECAB2, SLC38A8, MBTPS1, HSDL1, DNAAF1, TAF1C, ADAD2, KCNG4, WFDC1, ATP2C2, TLDC1, COTL1, KLHL36, USP10, CRISPLD2, ZDHHC7, KIAA0513, FAM92B, LOC400548, LINC00311, MIR5093, GSE1, GINS2, C16orf74, MIR1910, EMC8, LOC101928557, COX4I1, IRF8, MIR6774, LINC01082, LINC01081, LOC146513, LINC00917, FENDRR, FOXF1, MTHFSD, FLJ30679, FOXC2-AS1, FOXC2, FOXL1, LOC101928614, LOC102724344, LOC440390, LOC101928708, LOC101928682, C16orf95, LOC101928659, FBXO31, MAP1LC3B, ZCCHC14, LOC101928737, JPH3, KLHDC4, LOC102724467, SLC7A5, MIR6775, CA5A, BANP, LOC400553, LOC101928880, ZNF469, ZFPM1, MIR5189, ZC3H18, IL17C, CYBA, MVD, SNAI3-AS1, SNAI3, RNF166, CTU2, PIEZO1, MIR4722, LOC100289580, LOC339059, CDT1, APRT, GALNS, TRAPPC2L, PABPN1L, CBFA2T3, LOC101927793, LOC100129697, ACSF3, LINC00304, LOC400558, CDH15, SLC22A31, ZNF778, ANKRD11, LOC105371414, LOC100287036, LOC101927817, SPG7, RPL13, SNORD68, CPNE7, DPEP1, CHMP1A, SPATA33, CDK10, SPATA2L, VPS9D1, VPS9D1-AS1, ZNF276, FANCA, SPIRE2, TCF25, MC1R, TUBB3, DEF8, CENPBD1, AFG3L1P, DBNDD1, GAS8, GAS8-AS1, URAHP, PRDM7 | arr[GRCh37] 16p12.2q24.3(23403743_90158005)x3 |
| 1  BM | Gain | 17 | p11.2 | q24.3 | 48,398 | CCDC144CP, FAM106B, LGALS9B, KRT16P3, CDRT15L2, LOC100287072, CCDC144NL, CCDC144NL-AS1, LOC339260, USP22, LINC01563, DHRS7B, TMEM11, NATD1, MAP2K3, KCNJ12, KCNJ18, C17orf51, FAM27E5, FLJ36000, MTRNR2L1, LOC105371703, MIR4522, WSB1, TBC1D3P5, KSR1, LGALS9, NOS2, LYRM9, NLK, PYY2, PPY2P, KRT18P55, TMEM97, IFT20, TNFAIP1, POLDIP2, TMEM199, MIR4723, SEBOX, VTN, SARM1, SLC46A1, SLC13A2, FOXN1, UNC119, PIGS, ALDOC, SPAG5, SPAG5-AS1, SGK494, KIAA0100, SDF2, SUPT6H, PROCA1, RAB34, NARR, RPL23A, SNORD42B, SNORD4A, SNORD42A, SNORD4B, TLCD1, NEK8, TRAF4, FAM222B, ERAL1, MIR451A, MIR451B, MIR144, MIR4732, FLOT2, DHRS13, PHF12, LOC101927018, SEZ6, PIPOX, MYO18A, TIAF1, CRYBA1, NUFIP2, MIR4523, TAOK1, ABHD15, TP53I13, GIT1, ANKRD13B, CORO6, SSH2, EFCAB5, NSRP1, MIR423, MIR3184, SLC6A4, BLMH, TMIGD1, CPD, GOSR1, TBC1D29, LOC107133515, SH3GL1P2, SUZ12P1, CRLF3, ATAD5, TEFM, ADAP2, RNF135, DPRXP4, MIR4733, NF1, OMG, EVI2B, EVI2A, RAB11FIP4, MIR4724, MIR193A, MIR4725, MIR365B, COPRS, UTP6, SUZ12, LRRC37B, SH3GL1P1, LOC105371730, RHOT1, ARGFXP2, RHBDL3, C17orf75, MIR632, ZNF207, PSMD11, CDK5R1, MYO1D, TMEM98, SPACA3, ASIC2, AA06, LOC101927239, CCL2, CCL7, CCL11, CCL8, CCL13, CCL1, C17orf102, TMEM132E, CCT6B, ZNF830, LIG3, RFFL, RAD51L3-RFFL, RAD51D, FNDC8, NLE1, UNC45B, SLC35G3, SLFN5, SLFN11, SLFN12, SLFN13, SLFN12L, SLFN14, LOC105371743, SNORD7, PEX12, AP2B1, RASL10B, GAS2L2, MMP28, C17orf50, TAF15, HEATR9, CCL5, LRRC37A8P, RDM1, LYZL6, CCL16, CCL14, CCL15-CCL14, CCL15, CCL23, CCL18, CCL3, CCL4, CCL3L3, CCL3L1, CCL4L1, CCL4L2, TBC1D3H, TBC1D3G, TBC1D3F, TBC1D3B, ZNHIT3, MYO19, PIGW, GGNBP2, DHRS11, MRM1, LOC102723471, LHX1, AATF, MIR2909, ACACA, SNORA90, C17orf78, TADA2A, DUSP14, SYNRG, DDX52, MIR378J, HNF1B, YWHAEP7, TBC1D3C, TBC1D3L, TBC1D3, TBC1D3I, TBC1D3K, LOC101060389, TBC1D3E, LOC440434, MRPL45, GPR179, SOCS7, ARHGAP23, SRCIN1, C17orf96, MIR4734, MLLT6, MIR4726, CISD3, PCGF2, PSMB3, PIP4K2B, CWC25, MIR4727, C17orf98, RPL23, SNORA21, LASP1, MIR6779, LINC00672, FBXO47, LOC105371766, LRRC37A11P, LOC100131347, PLXDC1, ARL5C, CACNB1, RPL19, STAC2, FBXL20, MED1, CDK12, NEUROD2, PPP1R1B, STARD3, TCAP, PNMT, PGAP3, ERBB2, MIR4728, MIEN1, GRB7, IKZF3, ZPBP2, GSDMB, ORMDL3, LRRC3C, GSDMA, PSMD3, CSF3, MED24, MIR6884, SNORD124, THRA, NR1D1, MSL1, CASC3, MIR6866, RAPGEFL1, MIR6867, WIPF2, CDC6, RARA, RARA-AS1, GJD3, TOP2A, IGFBP4, TNS4, CCR7, SMARCE1, KRT222, KRT24, KRT25, KRT26, KRT27, KRT28, KRT10, TMEM99, KRT12, KRT20, KRT23, KRT39, KRT40, KRTAP3-3, KRTAP3-2, KRTAP3-1, KRTAP1-5, KRTAP1-4, KRTAP1-3, KRTAP1-1, KRTAP2-1, KRTAP2-2, KRTAP2-3, KRTAP2-4, KRTAP4-7, KRTAP4-8, KRTAP4-9, KRTAP4-11, KRTAP4-12, KRTAP4-6, KRTAP4-5, KRTAP4-4, KRTAP4-3, KRTAP4-2, KRTAP4-1, KRTAP9-1, KRTAP9-2, KRTAP9-3, KRTAP9-9, KRTAP9-8, KRTAP9-4, KRTAP9-6, KRTAP9-7, KRTAP29-1, KRTAP16-1, KRTAP17-1, KRT33A, KRT33B, KRT34, KRT31, LOC100505782, KRT37, KRT38, KRT32, KRT35, KRT36, KRT13, KRT15, MIR6510, KRT19, LINC00974, KRT9, KRT14, KRT16, KRT17, KRT42P, EIF1, GAST, HAP1, JUP, P3H4, FKBP10, NT5C3B, KLHL10, KLHL11, ACLY, TTC25, CNP, DNAJC7, NKIRAS2, ZNF385C, DHX58, KAT2A, HSPB9, RAB5C, KCNH4, HCRT, GHDC, STAT5B, STAT5A, STAT3, PTRF, ATP6V0A1, MIR548AT, MIR5010, NAGLU, HSD17B1, COASY, MLX, PSMC3IP, FAM134C, TUBG1, TUBG2, PLEKHH3, CCR10, CNTNAP1, EZH1, MIR6780A, RAMP2-AS1, RAMP2, VPS25, WNK4, COA3, CNTD1, BECN1, MIR6781, PSME3, AOC2, AOC3, AOC4P, LINC00671, G6PC, AARSD1, PTGES3L-AARSD1, PTGES3L, RUNDC1, RPL27, IFI35, VAT1, RND2, BRCA1, NBR2, LOC101929767, NBR1, TMEM106A, LINC00854, LINC00910, ARL4D, MIR2117, DHX8, ETV4, MEOX1, SOST, DUSP3, C17orf105, MPP3, CD300LG, MPP2, FAM215A, LOC107546764, PPY, PYY, NAGS, TMEM101, LSM12, G6PC3, HDAC5, LOC105371789, C17orf53, ASB16, ASB16-AS1, TMUB2, ATXN7L3, UBTF, MIR6782, SLC4A1, RUNDC3A-AS1, RUNDC3A, SLC25A39, GRN, FAM171A2, ITGA2B, GPATCH8, FZD2, LINC01180, MEIOC, CCDC43, DBF4B, ADAM11, GJC1, HIGD1B, EFTUD2, CCDC103, GFAP, KIF18B, MIR6783, C1QL1, DCAKD, NMT1, PLCD3, MIR6784, ACBD4, HEXIM1, HEXIM2, LOC105371795, FMNL1, MAP3K14-AS1, SPATA32, MAP3K14, ARHGAP27, PLEKHM1, MIR4315-2, MIR4315-1, LRRC37A4P, LOC644172, MGC57346, MGC57346-CRHR1, CRHR1-IT1, CRHR1, MAPT-AS1, SPPL2C, MAPT, MAPT-IT1, STH, KANSL1, KANSL1-AS1, LRRC37A, ARL17A, ARL17B, NSFP1, LRRC37A2, NSF, WNT3, WNT9B, GOSR2, MIR5089, RPRML, CDC27, MYL4, ITGB3, THCAT158, EFCAB13, MRPL45P2, NPEPPS, KPNB1, TBKBP1, TBX21, OSBPL7, MRPL10, LRRC46, SCRN2, SP6, LOC102724532, SP2, SP2-AS1, PNPO, PRR15L, CDK5RAP3, COPZ2, MIR152, NFE2L1, CBX1, SNX11, SKAP1, MIR1203, THRA1/BTR, LOC101927166, HOXB1, HOXB2, HOXB-AS1, HOXB3, HOXB4, MIR10A, HOXB-AS3, HOXB5, HOXB6, HOXB7, HOXB8, HOXB9, MIR196A1, PRAC1, PRAC2, MIR3185, HOXB13, TTLL6, CALCOCO2, LOC105371814, ATP5G1, UBE2Z, SNF8, GIP, IGF2BP1, B4GALNT2, GNGT2, ABI3, PHOSPHO1, FLJ40194, MIR6129, ZNF652, LOC102724596, PHB, LOC101927207, NGFR, LOC100288866, MIR6165, NXPH3, SPOP, SLC35B1, FAM117A, KAT7, TAC4, FLJ45513, DLX4, DLX3, LOC284080, ITGA3, PDK2, SAMD14, PPP1R9B, SGCA, HILS1, COL1A1, LOC101927230, TMEM92, TMEM92-AS1, XYLT2, MRPL27, EME1, LRRC59, ACSF2, CHAD, RSAD1, MYCBPAP, EPN3, LOC105371824, SPATA20, CACNA1G-AS1, CACNA1G, ABCC3, ANKRD40, LUC7L3, LINC00483, MIR8059, WFIKKN2, TOB1, TOB1-AS1, SPAG9, NME1-NME2, NME1, NME2, MBTD1, UTP18, LOC101927274, LOC440446, CA10, C17orf112, KIF2B, TOM1L1, COX11, STXBP4, HLF, MMD, TMEM100, PCTP, ANKFN1, NOG, C17orf67, DGKE, MTVR2, TRIM25, MIR3614, COIL, SCPEP1, RNF126P1, AKAP1, MSI2, LOC101927557, LOC101927539, CCDC182, MRPS23, CUEDC1, VEZF1, SRSF1, LOC101927666, DYNLL2, OR4D1, MSX2P1, OR4D2, EPX, MKS1, LPO, MPO, TSPOAP1, TSPOAP1-AS1, MIR142, MIR4736, SUPT4H1, RNF43, HSF5, MTMR4, SEPT4-AS1, SEPT4, C17orf47, TEX14, RAD51C, PPM1E, TRIM37, SKA2, MIR454, MIR301A, PRR11, SMG8, GDPD1, YPEL2, MIR4729, LINC01476, DHX40, CLTC, PTRH2, VMP1, MIR21, TUBD1, RPS6KB1, RNFT1, TBC1D3P1-DHX40P1, LOC101927755, MIR4737, HEATR6, LOC105371849, WFDC21P, LOC653653, CA4, USP32, SCARNA20, C17orf64, APPBP2, LOC388406, PPM1D, BCAS3, LOC101927855, TBX2-AS1, TBX2, C17orf82, TBX4, NACA2, BRIP1, INTS2, MED13, TBC1D3P2, EFCAB3, METTL2A, TLK2, MRC2, MARCH10, LOC101927877, MIR633, TANC2, CYB561, ACE, KCNH6, DCAF7, TACO1, MAP3K3, LIMD2, LOC729683, STRADA, CCDC47, DDX42, FTSJ3, PSMC5, SMARCD2, TCAM1P, CSH2, GH2, CSH1, CSHL1, GH1, CD79B, SCN4A, PRR29-AS1, PRR29, ICAM2, ERN1, SNHG25, SNORD104, SNORA50C, TEX2, PECAM1, MILR1, POLG2, DDX5, MIR3064, MIR5047, CEP95, SMURF2, LOC146880, MIR6080, PLEKHM1P1, LRRC37A3, AMZ2P1, GNA13, LOC100507002, RGS9, CRAT40, AXIN2, CEP112, APOH, PRKCA, PRKCA-AS1, MIR634, CACNG5, CACNG4, CACNG1, HELZ, LOC101928021, PSMD12, PITPNC1, MIR548AA2, MIR548D2, NOL11, SNORA38B, BPTF, C17orf58, KPNA2, LINC00674, LOC440461, AMZ2, ARSG, SLC16A6, PRKAR1A, WIPI1, MIR635, FAM20A, LINC01482, ABCA8, ABCA9, ABCA9-AS1, ABCA6, MIR4524B, MIR4524A, ABCA10, PRO1804, ABCA5, MAP2K6, LINC01483, LINC01497, LINC01028, KCNJ16, KCNJ2-AS1, KCNJ2 | arr[GRCh37] 17p11.2q24.3(20301296_68699516)x3 |
| 1  BM | Gain | 17 | q25.2 | q25.3 | 5,349 | MGAT5B, LOC105371899, SNHG20, SEC14L1, SCARNA16, MIR6516, LOC105371907, SEPT9, MIR4316, LOC100507351, LOC100132174, FLJ45079, TNRC6C, TNRC6C-AS1, TMC6, TMC8, C17orf99, SYNGR2, TK1, AFMID, BIRC5, TMEM235, LOC100996291, SOCS3, LOC101928674, PGS1, DNAH17, DNAH17-AS1, LOC101928710, CYTH1, USP36, TIMP2, CEP295NL, LGALS3BP, CANT1, C1QTNF1-AS1, C1QTNF1, ENGASE, RBFOX3, MIR4739, HP09025, ENPP7, CBX2, CBX8, CBX4, LOC101928766, LOC101928738, TBC1D16, CCDC40, GAA, EIF4A3, CARD14, SGSH, SLC26A11, RNF213, LOC100294362, ENDOV, MIR4730, NPTX1, RPTOR, LOC101928855, CHMP6, BAIAP2-AS1, BAIAP2, AATK, MIR657, MIR3065, MIR338, MIR1250, AATK-AS1, CEP131, TEPSIN, LOC105371925, C17orf89, SLC38A10, LINC00482, TMEM105, LOC100130370, BAHCC1, MIR4740, MIR3186, ACTG1, FSCN2, FAAP100, NPLOC4, TSPAN10, PDE6G, OXLD1, CCDC137, ARL16, HGS, MIR6786, MRPL12, SLC25A10, GCGR, MCRIP1, PPP1R27, P4HB, ARHGDIA, ALYREF, ANAPC11, NPB, PCYT2, SIRT7, MAFG, MAFG-AS1, PYCR1, MYADML2, NOTUM, ASPSCR1, STRA13, LRRC45, RAC3, DCXR, RFNG, GPS1, DUS1L, FASN, SNORD134, CCDC57, SLC16A3, MIR6787, CSNK1D, LOC101929511 | arr[GRCh37] 17q25.2q25.3(74914157_80263427)x3 |
| 1  BM | Gain | 19 | p13.3 | p12 | 23,443 | PLPP2, MIER2, THEG, C2CD4C, SHC2, ODF3L2, MADCAM1, TPGS1, CDC34, GZMM, BSG, HCN2, POLRMT, FGF22, RNF126, FSTL3, PRSS57, PALM, MISP, PTBP1, MIR4745, PLPPR3, MIR3187, AZU1, PRTN3, ELANE, CFD, MED16, RNU6-2, R3HDM4, KISS1R, ARID3A, WDR18, GRIN3B, TMEM259, CNN2, ABCA7, ARHGAP45, POLR2E, GPX4, SBNO2, STK11, CBARP, ATP5D, MIDN, CIRBP-AS1, CIRBP, C19orf24, EFNA2, MUM1, NDUFS7, GAMT, DAZAP1, RPS15, APC2, C19orf25, PCSK4, REEP6, ADAMTSL5, PLK5, MEX3D, MBD3, UQCR11, TCF3, ONECUT3, ATP8B3, REXO1, MIR1909, LOC100288123, KLF16, ABHD17A, ADAT3, SCAMP4, CSNK1G2, CSNK1G2-AS1, BTBD2, MKNK2, MOB3A, IZUMO4, AP3D1, DOT1L, PLEKHJ1, MIR1227, MIR6789, SF3A2, AMH, MIR4321, JSRP1, OAZ1, C19orf35, LINGO3, LSM7, SPPL2B, TMPRSS9, TIMM13, LMNB2, MIR7108, LOC101928602, GADD45B, GNG7, MIR7850, DIRAS1, SLC39A3, SGTA, THOP1, ZNF554, ZNF555, ZNF556, ZNF57, ZNF77, TLE6, TLE2, AES, GNA11, GNA15, LOC100996351, S1PR4, NCLN, CELF5, NFIC, SMIM24, DOHH, FZR1, C19orf71, MFSD12, HMG20B, GIPC3, TBXA2R, CACTIN-AS1, CACTIN, PIP5K1C, TJP3, MIR1268A, APBA3, MRPL54, RAX2, MATK, ZFR2, ATCAY, NMRK2, DAPK3, MIR637, EEF2, SNORD37, PIAS4, ZBTB7A, MAP2K2, CREB3L3, SIRT6, ANKRD24, EBI3, CCDC94, SHD, TMIGD2, FSD1, STAP2, MPND, SH3GL1, CHAF1A, UBXN6, MIR4746, HDGFRP2, PLIN4, PLIN5, LRG1, SEMA6B, TNFAIP8L1, MYDGF, DPP9, DPP9-AS1, MIR7-3HG, MIR7-3, FEM1A, TICAM1, PLIN3, ARRDC5, UHRF1, MIR4747, KDM4B, PTPRS, ZNRF4, TINCR, SAFB2, SAFB, C19orf70, HSD11B1L, RPL36, LONP1, CATSPERD, PRR22, DUS3L, NRTN, FUT6, FUT3, LOC101928844, FUT5, NDUFA11, VMAC, CAPS, RANBP3, LOC100128568, RFX2, ACSBG2, MLLT1, ACER1, CLPP, ALKBH7, PSPN, GTF2F1, MIR6885, MIR6790, LOC390877, KHSRP, MIR3940, SLC25A41, SLC25A23, CRB3, DENND1C, TUBB4A, TNFSF9, CD70, TNFSF14, C3, GPR108, MIR6791, TRIP10, SH2D3A, VAV1, ADGRE1, ADGRE4P, FLJ25758, MBD3L5, MBD3L4, MBD3L2, MBD3L3, ZNF557, INSR, ARHGEF18, LOC100128573, PEX11G, C19orf45, ZNF358, MCOLN1, PNPLA6, CAMSAP3, MIR6792, XAB2, PET100, PCP2, STXBP2, RETN, MCEMP1, TRAPPC5, FCER2, CLEC4G, CD209, CLEC4M, CLEC4GP1, EVI5L, PRR36, LYPLA2P2, LRRC8E, MAP2K7, TGFBR3L, SNAPC2, CTXN1, TIMM44, ELAVL1, CCL25, FBN3, CERS4, CD320, NDUFA7, RPS28, KANK3, ANGPTL4, RAB11B-AS1, MIR4999, RAB11B, MARCH2, HNRNPM, PRAM1, ZNF414, MYO1F, ADAMTS10, ACTL9, OR2Z1, ZNF558, MBD3L1, MUC16, OR1M1, OR7G2, OR7G1, OR7G3, ZNF317, OR7D2, OR7D4, OR7E24, ZNF699, ZNF559, ZNF559-ZNF177, ZNF177, ZNF266, ZNF560, ZNF426, LOC101928238, ZNF121, ZNF561, ZNF561-AS1, ZNF562, ZNF846, FBXL12, UBL5, PIN1, OLFM2, COL5A3, RDH8, MIR5589, C3P1, C19orf66, ANGPTL6, PPAN, PPAN-P2RY11, SNORD105, SNORD105B, P2RY11, EIF3G, DNMT1, S1PR2, MIR4322, MRPL4, ICAM1, ICAM4, ICAM5, ZGLP1, FDX1L, RAVER1, ICAM3, TYK2, CDC37, MIR1181, PDE4A, KEAP1, S1PR5, ATG4D, MIR1238, KRI1, CDKN2D, AP1M2, SLC44A2, ILF3-AS1, ILF3, QTRT1, DNM2, MIR638, MIR4748, MIR199A1, MIR6793, TMED1, C19orf38, CARM1, YIPF2, C19orf52, SMARCA4, LDLR, MIR6886, SPC24, KANK2, DOCK6, LOC105372273, ANGPTL8, TSPAN16, RAB3D, TMEM205, CCDC159, PLPPR2, SWSAP1, EPOR, RGL3, CCDC151, PRKCSH, ELAVL3, ZNF653, MIR7974, ECSIT, CNN1, ELOF1, ACP5, ZNF627, ZNF833P, ZNF823, ZNF441, ZNF491, ZNF440, ZNF439, ZNF69, ZNF700, ZNF763, LOC101928464, ZNF433, ZNF878, ZNF844, ZNF788, ZNF20, ZNF625-ZNF20, ZNF625, ZNF136, LOC100289333, ZNF44, ZNF563, ZNF442, ZNF799, ZNF443, ZNF709, ZNF564, ZNF490, ZNF791, MAN2B1, WDR83, WDR83OS, DHPS, LOC105372280, FBXW9, TNPO2, SNORD135, SNORD41, C19orf43, ASNA1, BEST2, HOOK2, MIR5684, JUNB, PRDX2, RNASEH2A, RTBDN, MAST1, MIR6794, DNASE2, KLF1, GCDH, SYCE2, FARSA, CALR, MIR6515, RAD23A, GADD45GIP1, DAND5, NFIX, LYL1, TRMT1, NACC1, STX10, IER2, CACNA1A, CCDC130, MRI1, C19orf53, ZSWIM4, LOC284454, MIR24-2, MIR27A, MIR23A, MIR181C, MIR181D, NANOS3, C19orf57, CC2D1A, PODNL1, DCAF15, RFX1, RLN3, IL27RA, PALM3, LOC113230, MIR1199, C19orf67, SAMD1, PRKACA, ASF1B, LOC100507373, ADGRL1, LOC105372288, LOC101928845, ADGRE5, DDX39A, PKN1, PTGER1, GIPC1, DNAJB1, MIR639, TECR, NDUFB7, CLEC17A, ADGRE3, SNORA104, ZNF333, ADGRE2, OR7C1, OR7A5, OR7A10, OR7A17, OR7C2, SLC1A6, CCDC105, CASP14, OR1I1, SYDE1, ILVBL, NOTCH3, MIR6795, EPHX3, BRD4, AKAP8, AKAP8L, WIZ, MIR1470, RASAL3, PGLYRP2, CYP4F22, CYP4F8, CYP4F3, CYP4F12, OR10H2, OR10H3, CYP4F24P, OR10H5, OR10H1, UCA1, LOC102724279, CYP4F2, CYP4F11, OR10H4, LINC00661, LINC00905, TPM4, RAB8A, HSH2D, CIB3, FAM32A, AP1M1, KLF2, EPS15L1, CALR3, C19orf44, CHERP, SLC35E1, MED26, SMIM7, TMEM38A, NWD1, SIN3B, F2RL3, CPAMD8, HAUS8, MYO9B, USE1, OCEL1, NR2F6, USHBP1, BABAM1, ANKLE1, ABHD8, MRPL34, DDA1, ANO8, GTPBP3, PLVAP, BST2, BISPR, MVB12A, TMEM221, NXNL1, SLC27A1, PGLS, FAM129C, COLGALT1, UNC13A, MAP1S, FCHO1, B3GNT3, INSL3, JAK3, RPL18A, SNORA68, SLC5A5, CCDC124, KCNN1, ARRDC2, IL12RB1, MAST3, PIK3R2, IFI30, MPV17L2, RAB3A, LOC102725254, PDE4C, LOC729966, KIAA1683, JUND, MIR3188, LSM4, PGPEP1, GDF15, MIR3189, LRRC25, SSBP4, ISYNA1, ELL, FKBP8, KXD1, UBA52, C19orf60, CRLF1, TMEM59L, KLHL26, CRTC1, COMP, UPF1, GDF1, CERS1, COPE, DDX49, HOMER3, LOC102724360, SUGP2, ARMC6, SLC25A42, TMEM161A, MEF2B, BORCS8-MEF2B, BORCS8, RFXANK, NR2C2AP, NCAN, HAPLN4, TM6SF2, SUGP1, MAU2, GATAD2A, MIR640, TSSK6, NDUFA13, YJEFN3, CILP2, PBX4, LPAR2, GMIP, ATP13A1, ZNF101, ZNF14, LINC00663, ZNF506, ZNF253, ZNF93, ZNF682, ZNF90, ZNF486, MIR1270, ZNF826P, ZNF737, ZNF626, ZNF85, ZNF430, ZNF714, ZNF431, ZNF708, ZNF738, ZNF493, LINC00664, ZNF429, ZNF100, LOC641367, ZNF43, ZNF208, ZNF257, ZNF676, ZNF729, ZNF98, LOC101929124, LINC01233, GOLGA2P9, LOC100996349, LOC374890, ZNF492, ZNF99, ZNF728, LOC101929164, LOC101929144, ZNF730, ZNF724P, IPO5P1, ZNF91, LINC01224 | arr[GRCh37] 19p13.3p12(247231_23690418)x3 |
| 1  BM | Gain | 20 | p13 | p12.1 | 14,371 | DEFB125, DEFB126, DEFB127, DEFB128, DEFB129, DEFB132, C20orf96, ZCCHC3, NRSN2-AS1, SOX12, NRSN2, TRIB3, RBCK1, TBC1D20, CSNK2A1, TCF15, SRXN1, SCRT2, SLC52A3, FAM110A, ANGPT4, RSPO4, PSMF1, LOC105372493, TMEM74B, C20orf202, RAD21L1, SNPH, SDCBP2, FKBP1A-SDCBP2, SDCBP2-AS1, FKBP1A, MIR6869, NSFL1C, SIRPB2, SIRPD, SIRPB1, SIRPG, SIRPG-AS1, LOC100289473, SIRPA, LOC727993, PDYN, STK35, LOC388780, TGM3, TGM6, SNRPB, SNORD119, ZNF343, TMC2, NOP56, MIR1292, SNORD110, SNORA51, SNORD86, SNORD56, SNORD57, IDH3B, EBF4, CPXM1, C20orf141, TMEM239, PCED1A, VPS16, PTPRA, GNRH2, MRPS26, OXT, AVP, UBOX5-AS1, UBOX5, FASTKD5, LZTS3, DDRGK1, ITPA, SLC4A11, C20orf194, ATRN, GFRA4, ADAM33, SIGLEC1, HSPA12B, C20orf27, SPEF1, CENPB, CDC25B, LOC101929125, AP5S1, MAVS, PANK2, MIR103A2, MIR103B2, RNF24, SMOX, LINC01433, ADRA1D, PRNP, PRND, PRNT, RASSF2, SLC23A2, TMEM230, PCNA, PCNA-AS1, CDS2, PROKR2, LINC00658, LOC643406, LINC00654, LOC101929207, GPCPD1, C20orf196, CHGB, TRMT6, MCM8, MCM8-AS1, CRLS1, LRRN4, FERMT1, CASC20, BMP2, LINC01428, LOC101929288, LOC101929312, MIR8062, HAO1, TMX4, PLCB1, PLCB4, LAMP5-AS1, LAMP5, PAK5, LOC101929371, SNAP25-AS1, ANKEF1, SNAP25, MKKS, SLX4IP, JAG1, MIR6870, LOC101929395, LOC101929413, LOC339593, LINC00687, BTBD3, LOC101929486, LOC102606466, LOC100505515, SPTLC3, ISM1, ISM1-AS1, TASP1, ESF1, NDUFAF5, SEL1L2, MACROD2, FLRT3 | arr[GRCh37] 20p13p12.1(69093_14440202)x3 |
| 1  BM | Gain | 20 | p12.1 | p11.1 | 10,580 | MACROD2, LOC613266, KIF16B, SNRPB2, OTOR, PCSK2, BFSP1, DSTN, RRBP1, BANF2, SNX5, SNORD17, MGME1, OVOL2, PET117, KAT14, ZNF133, LINC00851, DZANK1, POLR3F, MIR3192, RBBP9, SEC23B, LINC00493, DTD1, LOC101929526, LINC00652, LOC100270804, C20orf78, SCP2D1, SLC24A3, LOC100130264, RIN2, NAA20, CRNKL1, CFAP61, INSM1, RALGAPA2, KIZ, KIZ-AS1, XRN2, NKX2-4, NKX2-2, LOC101929625, LOC101929608, PAX1, LINC01432, LINC01427, LOC284788, LINC00261, FOXA2, LINC01384, SSTR4, THBD, CD93, LINC00656, NXT1, LINC01431, GZF1, NAPB, CSTL1, CST11, CST8, CST13P, CST9L, CST9, CST3, CST4, CST1, CST2, CST5, GGTLC1, FLJ33581, SYNDIG1, CST7, APMAP, ACSS1, VSX1, LOC284798, LOC101926889, ENTPD6, PYGB, ABHD12, GINS1, NINL, NANP, ZNF337-AS1, ZNF337, LOC105372582, LOC105379511, FAM182B, LOC101926935, LOC101926955, LOC100134868, FAM182A, NCOR1P1, MIR663AHG, MIR663A | arr[GRCh37] 20p12.1p11.1(15729313_26309255)x3 |
| 1  BM | Gain | 20 | q11.21 | q13.33 | 33,393 | LINC01598, FRG1BP, FRG1DP, MLLT10P1, DEFB115, DEFB116, DEFB118, DEFB119, DEFB121, DEFB122, DEFB123, DEFB124, REM1, LINC00028, HM13, HM13-AS1, ID1, MIR3193, COX4I2, BCL2L1, ABALON, TPX2, MYLK2, FOXS1, DUSP15, TTLL9, PDRG1, XKR7, MIR7641-2, CCM2L, HCK, TM9SF4, TSPY26P, PLAGL2, POFUT1, MIR1825, KIF3B, ASXL1, NOL4L, LOC101929698, LOC149950, C20orf203, COMMD7, DNMT3B, MAPRE1, SUN5, BPIFB2, BPIFB6, BPIFB3, BPIFB4, BPIFA2, BPIFA4P, BPIFA3, BPIFA1, BPIFB1, CDK5RAP1, SNTA1, CBFA2T2, NECAB3, C20orf144, ACTL10, E2F1, PXMP4, ZNF341, ZNF341-AS1, CHMP4B, RALY-AS1, RALY, MIR4755, EIF2S2, ASIP, AHCY, ITCH, MIR644A, DYNLRB1, MAP1LC3A, PIGU, TP53INP2, NCOA6, HMGB3P1, GGT7, ACSS2, GSS, MYH7B, MIR499A, MIR499B, TRPC4AP, EDEM2, PROCR, MMP24, MMP24-AS1, EIF6, FAM83C-AS1, FAM83C, UQCC1, GDF5, MIR1289-1, CEP250, C20orf173, ERGIC3, FER1L4, SPAG4, CPNE1, RBM12, NFS1, ROMO1, RBM39, PHF20, SCAND1, CNBD2, NORAD, EPB41L1, AAR2, DLGAP4, DLGAP4-AS1, MYL9, TGIF2, TGIF2-C20orf24, C20orf24, SLA2, NDRG3, DSN1, SOGA1, TLDC2, SAMHD1, RBL1, MROH8, RPN2, GHRH, MANBAL, SRC, BLCAP, NNAT, LINC00489, LOC100287792, CTNNBL1, VSTM2L, TTI1, RPRD1B, TGM2, KIAA1755, LOC149684, BPI, LBP, SNHG17, SNORA71B, SNORA71A, SNORA71C, SNORA71D, SNHG11, SNORA71E, SNORA60, RALGAPB, MIR548O2, ADIG, ARHGAP40, SLC32A1, ACTR5, PPP1R16B, FAM83D, DHX35, LOC339568, LINC01370, MAFB, LOC100128988, TOP1, PLCG1-AS1, PLCG1, MIR6871, ZHX3, LPIN3, EMILIN3, CHD6, PTPRT, LOC101927159, SRSF6, L3MBTL1, SGK2, IFT52, MYBL2, GTSF1L, LOC105372626, TOX2, JPH2, OSER1, OSER1-AS1, GDAP1L1, FITM2, R3HDML, HNF4A, HNF4A-AS1, MIR3646, LINC01430, LINC01620, TTPAL, SERINC3, PKIG, ADA, LINC01260, KCNK15-AS1, WISP2, KCNK15, RIMS4, YWHAB, PABPC1L, TOMM34, STK4-AS1, STK4, KCNS1, WFDC5, WFDC12, PI3, SEMG1, SEMG2, SLPI, MATN4, RBPJL, SDC4, SYS1, SYS1-DBNDD2, TP53TG5, DBNDD2, PIGT, MIR6812, WFDC2, SPINT3, WFDC6, EPPIN-WFDC6, EPPIN, WFDC8, WFDC9, WFDC10A, WFDC11, WFDC10B, WFDC13, MIR3617, SPINT4, WFDC3, DNTTIP1, UBE2C, TNNC2, SNX21, ACOT8, ZSWIM3, ZSWIM1, SPATA25, NEURL2, CTSA, PLTP, PCIF1, ZNF335, MMP9, SLC12A5, NCOA5, CD40, CDH22, SLC35C2, ELMO2, ZNF663P, MKRN7P, ZNF334, OCSTAMP, SLC13A3, TP53RK, SLC2A10, EYA2, MIR3616, ZMYND8, LOC100131496, LOC101927377, NCOA3, SULF2, LINC01522, LINC01523, LINC00494, PREX1, ARFGEF2, CSE1L-AS1, CSE1L, STAU1, DDX27, ZNFX1, ZFAS1, SNORD12C, SNORD12B, SNORD12, KCNB1, PTGIS, B4GALT5, SLC9A8, SPATA2, LOC105372653, RNF114, SNAI1, TRERNA1, UBE2V1, TMEM189-UBE2V1, TMEM189, LINC01273, CEBPB-AS1, CEBPB, LINC01272, LINC01270, LINC01271, PTPN1, MIR645, FAM65C, MIR1302-5, LOC100506175, PARD6B, BCAS4, ADNP, ADNP-AS1, DPM1, MOCS3, KCNG1, NFATC2, MIR3194, ATP9A, SALL4, LINC01429, ZFP64, LINC01524, TSHZ2, LOC101927770, ZNF217, LOC105372672, SUMO1P1, BCAS1, MIR4756, CYP24A1, PFDN4, DOK5, LINC01441, LINC01440, CBLN4, MC3R, FAM210B, AURKA, CSTF1, CASS4, RTFDC1, GCNT7, FAM209A, FAM209B, LOC105372682, TFAP2C, BMP7, BMP7-AS1, MIR4325, SPO11, RAE1, MTRNR2L3, RBM38, CTCFL, PCK1, ZBP1, PMEPA1, NKILA, MIR4532, C20orf85, ANKRD60, PPP4R1L, RAB22A, VAPB, APCDD1L, APCDD1L-AS1, LOC79160, STX16, STX16-NPEPL1, NPEPL1, LOC105372695, MIR296, MIR298, GNAS-AS1, GNAS, LOC101927932, NELFCD, CTSZ, TUBB1, ATP5E, SLMO2-ATP5E, PRELID3B, ZNF831, EDN3, PHACTR3, LOC100506384, SYCP2, FAM217B, PPP1R3D, CDH26, C20orf197, LOC729296, MIR646HG, MIR646, LOC101928048, MIR4533, MIR548AG2, LOC100506470, CDH4, MIR1257, TAF4, MIR3195, LSM14B, PSMA7, SS18L1, MTG2, HRH3, OSBPL2, ADRM1, LAMA5, MIR4758, LAMA5-AS1, RPS21, CABLES2, RBBP8NL, GATA5, C20orf166-AS1, MIR1-1HG, MIR1-1, MIR133A2, SLCO4A1, SLCO4A1-AS1, NTSR1, LINC00659, MRGBP, OGFR-AS1, OGFR, COL9A3, TCFL5, DPH3P1, DIDO1, GID8, SLC17A9, BHLHE23, LOC63930, LINC00029, LINC01056, HAR1B, HAR1A, MIR124-3, YTHDF1, BIRC7, MIR3196, NKAIN4, FLJ16779, ARFGAP1, MIR4326, COL20A1, CHRNA4, LOC100130587, KCNQ2, EEF1A2, PPDPF, PTK6, SRMS, FNDC11, HELZ2, GMEB2, LOC100505771, STMN3, RTEL1, RTEL1-TNFRSF6B, TNFRSF6B, ARFRP1, ZGPAT, LIME1, SLC2A4RG, ZBTB46, ZBTB46-AS1, ABHD16B, TPD52L2, DNAJC5, MIR941-1, MIR941-4, MIR941-3, MIR941-2, MIR941-5, UCKL1, MIR1914, MIR647, UCKL1-AS1, ZNF512B, SAMD10, PRPF6, LINC00176, SOX18, TCEA2, RGS19, MIR6813, OPRL1, LKAAEAR1, NPBWR2, MYT1, PCMTD2 | arr[GRCh37] 20q11.21q13.33(29519155_62912463)x3 |
| 1  BM | Loss | 8 | p23.3 | p12 | 29,297 | RPL23AP53, ZNF596, FAM87A, FBXO25, TDRP, ERICH1, ERICH1-AS1, LOC401442, LOC286083, DLGAP2, DLGAP2-AS1, LOC101927752, CLN8, MIR3674, MIR596, ARHGEF10, LOC101928058, KBTBD11-OT1, KBTBD11, MYOM2, MIR7160, LOC101927815, CSMD1, LOC100287015, MCPH1, ANGPT2, MCPH1-AS1, MIR8055, AGPAT5, MIR4659A, MIR4659B, XKR5, GS1-24F4.2, DEFB1, DEFA6, DEFA4, DEFA8P, DEFA9P, DEFA10P, DEFA1, DEFA1B, DEFT1P2, DEFT1P, DEFA3, DEFA11P, DEFA5, LINC00965, FAM66B, DEFB109P1B, USP17L1, USP17L4, ZNF705G, DEFB4B, DEFB103B, DEFB103A, SPAG11B, DEFB104A, DEFB104B, DEFB106B, DEFB106A, DEFB105B, DEFB105A, DEFB107B, DEFB107A, PRR23D1, PRR23D2, FAM90A7P, FAM90A10P, SPAG11A, DEFB4A, ZNF705B, FAM66E, USP17L8, USP17L3, MIR548I3, FAM86B3P, SGK223, CLDN23, MFHAS1, ERI1, MIR4660, PPP1R3B, LOC101929128, LOC157273, TNKS, MIR597, LINC00599, MIR124-1, MSRA, LINCR-0001, PRSS55, RP1L1, MIR4286, C8orf74, SOX7, PINX1, MIR1322, LOC101929229, XKR6, MIR598, LOC101929269, MTMR9, SLC35G5, TDH, FAM167A-AS1, FAM167A, BLK, LINC00208, GATA4, SNORA99, C8orf49, NEIL2, FDFT1, CTSB, DEFB136, DEFB135, DEFB134, LOC100133267, DEFB130, ZNF705D, FAM66D, LOC392196, USP17L7, USP17L2, FAM90A2P, FAM86B1, FAM66A, LOC649352, DEFB109P1, FAM90A25P, FAM86B2, LOC100506990, LOC729732, MIR5692A1, MIR5692A2, LONRF1, MIR3926-1, MIR3926-2, LOC340357, LINC00681, KIAA1456, DLC1, C8orf48, LOC102725080, SGCZ, MIR383, TUSC3, MSR1, FGF20, MICU3, ZDHHC2, CNOT7, VPS37A, MTMR7, SLC7A2, PDGFRL, MTUS1, MIR548V, FGL1, PCM1, ASAH1, LOC101929066, NAT1, NAT2, PSD3, LOC100128993, SH2D4A, CSGALNACT1, INTS10, LPL, SLC18A1, ATP6V1B2, LZTS1, LZTS1-AS1, LOC102467222, LOC286114, LOC101929172, GFRA2, DOK2, XPO7, NPM2, FGF17, DMTN, FAM160B2, NUDT18, HR, REEP4, LGI3, SFTPC, BMP1, PHYHIP, MIR320A, POLR3D, LOC100507071, PIWIL2, SLC39A14, PPP3CC, SORBS3, PDLIM2, C8orf58, CCAR2, BIN3, BIN3-IT1, EGR3, PEBP4, LOC101929237, RHOBTB2, TNFRSF10B, LOC286059, LOC254896, TNFRSF10C, TNFRSF10D, TNFRSF10A, LOC389641, CHMP7, R3HCC1, LOXL2, LOC100507156, ENTPD4, SLC25A37, NKX3-1, NKX2-6, STC1, ADAM28, LOC101929294, ADAMDEC1, ADAM7, LOC101929315, NEFM, NEFL, MIR6841, DOCK5, MIR6876, GNRH1, KCTD9, CDCA2, EBF2, PPP2R2A, BNIP3L, PNMA2, DPYSL2, ADRA1A, STMN4, TRIM35, PTK2B, MIR6842, CHRNA2, EPHX2, CLU, MIR6843, SCARA3, MIR3622B, MIR3622A, CCDC25, ESCO2, PBK, SCARA5, MIR4287, NUGGC, ELP3, PNOC, ZNF395, FBXO16, FZD3, MIR4288, MIR7641-2, EXTL3-AS1, EXTL3, INTS9, HMBOX1, KIF13B, DUSP4 | arr[GRCh37] 8p23.3p12(172416_29469606)x1 |
| 1  BM | Loss | 8 | q21.11 | q21.13 | 7,538 | MIR2052HG, PI15, CRISPLD1, CASC9, HNF4G, LINC01111, ZFHX4-AS1, ZFHX4, MIR3149, PEX2, LOC102724874, PKIA, PKIA-AS1, ZC2HC1A, LOC101241902, IL7, STMN2, HEY1, LINC01607, LOC101927040, MRPS28, TPD52, MIR5708, ZBTB10, ZNF704, PAG1, FABP5, PMP2, FABP9, FABP4, FABP12, IMPA1, SLC10A5, ZFAND1, CHMP4C, SNX16 | arr[GRCh37] 8q21.11q21.13(75657105_83195460)x1 |
| 1  BM | Loss | 10 | q11.21 | q22.3 | 34,113 | TMEM72-AS1, TMEM72, RASSF4, C10orf10, C10orf25, ZNF22, RSU1P2, ANKRD30BP3, MIR3156-1, OR13A1, ALOX5, LOC102724323, MARCH8, ZFAND4, FAM21C, AGAP4, PTPN20, FRMPD2B, BMS1P5, FAM35BP, LOC102724593, SYT15, GPRIN2, NPY4R, CH17-360D5.1, LINC00842, HNRNPA1P33, ANXA8, FAM25C, FAM25G, AGAP9, BMS1P6, FAM35DP, ANTXRLP1, ANTXRL, FAM25BP, ANXA8L1, CTSLP2, LOC107001062, LOC105378292, ZNF488, RBP3, GDF2, GDF10, GLUD1P7, AGAP12P, FRMPD2, MAPK8, ARHGAP22, WDFY4, LRRC18, MIR4294, VSTM4, FAM170B-AS1, FAM170B, C10orf128, C10orf71-AS1, C10orf71, DRGX, ERCC6, PGBD3, CHAT, SLC18A3, C10orf53, OGDHL, PARG, PARGP1, TIMM23B, AGAP7P, MSMB, NCOA4, TIMM23, AGAP6, FAM21EP, FAM21A, ASAH2, SGMS1, SGMS1-AS1, ASAH2B, A1CF, PRKG1, LOC102724719, MIR605, CSTF2T, PRKG1-AS1, DKK1, LINC01468, MBL2, PCDH15, LOC105378311, MIR548F1, MTRNR2L5, ZWINT, MIR3924, IPMK, CISD1, UBE2D1, TFAM, BICC1, FAM133CP, LINC00844, CCEPR, PHYHIPL, FAM13C, SLC16A9, MRLN, CCDC6, LINC01553, ANK3, CDK1, RHOBTB1, LINC00845, TMEM26, TMEM26-AS1, C10orf107, ARID5B, RTKN2, LOC283045, ZNF365, ADO, EGR2, NRBF2, JMJD1C, MIR1296, LOC105378330, JMJD1C-AS1, REEP3, ANXA2P3, LOC101928887, LINC01515, CTNNA3, LOC101928961, LRRTM3, MIR7151, DNAJC12, SIRT1, HERC4, POU5F1P5, MYPN, ATOH7, PBLD, HNRNPH3, RUFY2, DNA2, SLC25A16, TET1, CCAR1, SNORD98, MIR1254-1, STOX1, DDX50, DDX21, KIAA1279, SRGN, VPS26A, SUPV3L1, LOC101928994, HKDC1, HK1, TACR2, TSPAN15, NEUROG3, C10orf35, COL13A1, H2AFY2, AIFM2, TYSND1, SAR1A, PPA1, NPFFR1, LRRC20, EIF4EBP2, NODAL, PALD1, PRF1, ADAMTS14, TBATA, SGPL1, PCBD1, LOC105378349, UNC5B, UNC5B-AS1, SLC29A3, CDH23, LOC102723377, C10orf105, C10orf54, MIR7152, PSAP, CHST3, SPOCK2, ASCC1, ANAPC16, DDIT4, DNAJB12, MICU1, MCU, MIR4676, OIT3, PLA2G12B, P4HA1, NUDT13, ECD, FAM149B1, DNAJC9, DNAJC9-AS1, MRPS16, CFAP70, ANXA7, MSS51, PPP3CB, PPP3CB-AS1, USP54, MYOZ1, SYNPO2L, AGAP5, BMS1P4, GLUD1P3, SEC24C, FUT11, CHCHD1, ZSWIM8, ZSWIM8-AS1, NDST2, CAMK2G, C10orf55, PLAU, VCL, AP3M1, ADK, LOC102723439, KAT6B, LOC101929165, DUPD1, DUSP13, SAMD8, VDAC2, COMTD1, ZNF503, ZNF503-AS1, ZNF503-AS2, LOC101929234, C10orf11, MIR606, LOC105378367, KCNMA1, KCNMA1-AS1, KCNMA1-AS2, KCNMA1-AS3 | arr[GRCh37] 10q11.21q22.3(45103361_79216839)x1 |
| 1  BM | Loss | 14 | q21.2 | q24.2 | 25,249 | LINC00871, RPL10L, MDGA2, MIR548Y, LINC00648, RPS29, LRR1, RPL36AL, MGAT2, DNAAF2, POLE2, KLHDC1, KLHDC2, NEMF, ARF6, MIR6076, LINC01588, LINC01599, VCPKMT, SOS2, L2HGDH, MIR4504, ATP5S, CDKL1, MAP4K5, ATL1, SAV1, NIN, LOC105370489, ABHD12B, PYGL, TRIM9, TMX1, LINC00640, FRMD6-AS2, FRMD6, FRMD6-AS1, GNG2, LOC102723604, C14orf166, NID2, PTGDR, PTGER2, TXNDC16, GPR137C, ERO1A, PSMC6, STYX, GNPNAT1, FERMT2, DDHD1, LOC101927620, MIR5580, BMP4, CDKN3, CNIH1, GMFB, CGRRF1, SAMD4A, GCH1, MIR4308, WDHD1, SOCS4, MAPK1IP1L, LGALS3, DLGAP5, FBXO34, ATG14, TBPL2, KTN1-AS1, KTN1, RPL13AP3, LINC00520, PELI2, LOC101927690, TMEM260, OTX2, OTX2-AS1, EXOC5, AP5M1, NAA30, C14orf105, SLC35F4, C14orf37, ACTR10, PSMA3, PSMA3-AS1, ARID4A, TOMM20L, TIMM9, KIAA0586, DACT1, LINC01500, DAAM1, GPR135, L3HYPDH, JKAMP, CCDC175, RTN1, MIR5586, LRRC9, PCNX4, DHRS7, PPM1A, C14orf39, SIX6, SALRNA1, SIX1, SIX4, MNAT1, TRMT5, SLC38A6, TMEM30B, PRKCH, LOC101927780, FLJ22447, HIF1A-AS1, HIF1A, HIF1A-AS2, SNAPC1, SYT16, LINC00643, LINC00644, KCNH5, RHOJ, GPHB5, PPP2R5E, WDR89, SGPP1, SYNE2, MIR548H1, ESR2, TEX21P, MTHFD1, ZBTB25, AKAP5, ZBTB1, LOC102723809, HSPA2, PPP1R36, PLEKHG3, SPTB, MIR7855, CHURC1, CHURC1-FNTB, GPX2, RAB15, FNTB, MAX, MIR4706, LOC100506321, LOC100128233, MIR4708, FUT8, FUT8-AS1, MIR625, LINC00238, GPHN, FAM71D, MPP5, ATP6V1D, EIF2S1, PLEK2, MIR5694, TMEM229B, PLEKHH1, PIGH, ARG2, VTI1B, RDH11, RDH12, ZFYVE26, RAD51B, LOC100996664, ZFP36L1, ACTN1, ACTN1-AS1, DCAF5, EXD2, GALNT16, ERH, SLC39A9, PLEKHD1, CCDC177, SUSD6, LOC100289511, SRSF5, SLC10A1, SMOC1, SLC8A3, LOC646548, ADAM21P1, COX16, SYNJ2BP-COX16, SYNJ2BP, ADAM21, ADAM20P1, ADAM20, MED6, LOC101928075, TTC9, LINC01269, MAP3K9 | arr[GRCh37] 14q21.2q24.2(46029524_71278113)x1 |
| 1  BM | Loss | 21 | q11.2 | q21.2 | 8,865 | CYP4F29P, ANKRD20A11P, LIPI, RBM11, ABCC13, HSPA13, SAMSN1, SAMSN1-AS1, LOC388813, NRIP1, USP25, MIR99AHG, MIR99A, MIRLET7C, MIR125B2, LINC01549, CXADR, BTG3, C21orf91-OT1, C21orf91, CHODL-AS1, CHODL, TMPRSS15, MIR548XHG, MIR548X, LINC00320, NCAM2, LINC00317, LINC01425, LOC101927843, LINC00308 | arr[GRCh37] 21q11.2q21.2(15206085_24071413)x1 |
| 1  BM | cn-LOH | 5 | q11.2 | q35.3 | 126,807 | LOC102467080, ESM1, LOC102467081, GZMK, GZMA, CDC20B, GPX8, MIR449A, MIR449B, MIR449C, MCIDAS, CCNO, DHX29, SKIV2L2, PLPP1, MIR5687, RNF138P1, SLC38A9, DDX4, IL31RA, IL6ST, FLJ31104, ANKRD55, LOC102467147, C5orf67, MAP3K1, SETD9, MIER3, GPBP1, ACTBL2, LINCR-0003, LOC101928505, LOC101928539, LOC101928569, PLK2, GAPT, MIR548AE2, LOC101928600, RAB3C, PDE4D, PART1, DEPDC1B, ELOVL7, ERCC8, NDUFAF2, SMIM15, CTC-436P18.1, ZSWIM6, C5orf64, LOC101928651, LOC100506526, KIF2A, DIMT1, IPO11, LRRC70, IPO11-LRRC70, HTR1A, RNF180, RGS7BP, FAM159B, SREK1IP1, CWC27, ADAMTS6, CENPK, PPWD1, TRIM23, TRAPPC13, SGTB, NLN, ERBIN, LOC100303749, SREK1, LOC101928769, MAST4, LOC101928794, CD180, LOC101928858, LOC102467655, PIK3R1, LOC101928885, SLC30A5, CCNB1, CENPH, MRPS36, CDK7, CCDC125, AK6, TAF9, RAD17, MARVELD2, LOC101928924, OCLN, GTF2H2C, GTF2H2C_2, GUSBP3, SERF1A, SERF1B, SMN1, SMN2, SMA4, GTF2H2B, SMA5, LOC441081, GUSBP9, NAIP, GTF2H2, LOC647859, LOC102724392, PMCHL2, BDP1, MCCC2, CARTPT, MAP1B, MIR4803, MRPS27, PTCD2, ZNF366, LOC102503427, LOC102477328, TNPO1, MIR4804, FCHO2, TMEM171, LOC105379030, TMEM174, LOC340090, FOXD1, LINC01386, BTF3, ANKRA2, UTP15, ARHGEF28, LINC01335, LINC01333, LINC01331, ENC1, HEXB, GFM2, NSA2, FAM169A, LOC441086, GCNT4, LINC01336, ANKRD31, HMGCR, COL4A3BP, POLK, ANKDD1B, POC5, SV2C, IQGAP2, LOC101929109, F2RL2, NCRUPAR, F2R, F2RL1, S100Z, CRHBP, AGGF1, ZBED3, SNORA47, ZBED3-AS1, PDE8B, WDR41, OTP, TBCA, LOC101929154, AP3B1, SCAMP1-AS1, SCAMP1, LHFPL2, ARSB, DMGDH, BHMT2, BHMT, JMY, HOMER1, PAPD4, CMYA5, LINC01455, MTX3, THBS4, CTD-2201I18.1, SERINC5, LOC644936, SPZ1, CRSP8P, ZFYVE16, FAM151B, ANKRD34B, LINC01337, DHFR, MTRNR2L2, MSH3, RASGRF2-AS1, RASGRF2, RNU5E-1, RNU5D-1, CKMT2, CKMT2-AS1, ZCCHC9, ACOT12, SSBP2, ATG10, RPS23, ATP6AP1L, MIR3977, LINC01338, TMEM167A, SCARNA18, XRCC4, VCAN, LOC105379054, HAPLN1, EDIL3, NBPF22P, COX7C, MIR3607, LOC100505878, MIR4280, LOC101929380, LOC55338, RASA1, LOC644285, CCNH, TMEM161B, TMEM161B-AS1, LOC102546226, LINC00461, MIR9-2, MEF2C, MEF2C-AS1, MIR3660, LINC01339, CETN3, LOC731157, MBLAC2, POLR3G, LYSMD3, ADGRV1, LUCAT1, ARRDC3, ARRDC3-AS1, NR2F1-AS1, NR2F1, FAM172A, MIR2277, POU5F2, KIAA0825, SLF1, MCTP1, FAM81B, TTC37, ARSK, GPR150, RFESD, SPATA9, RHOBTB3, GLRX, LINC01554, ELL2, LOC101929710, MIR583, PCSK1, CAST, ERAP1, ERAP2, LNPEP, LIX1, RIOK2, LINC01340, RGMB, RGMB-AS1, CHD1, LOC100289230, CTD-2151A2.1, LOC100133050, FAM174A, ST8SIA4, MIR548P, SLCO4C1, SLCO6A1, LINC00492, LINC00491, PAM, GIN1, PPIP5K2, C5orf30, LOC102467212, NUDT12, RAB9BP1, LOC102467213, EFNA5, FBXL17, LINC01023, FER, PJA2, MAN2A1, LOC100289673, TMEM232, MIR548F3, SLC25A46, TSLP, WDR36, CAMK4, STARD4, STARD4-AS1, NREP, NREP-AS1, EPB41L4A-AS1, SNORA13, EPB41L4A, LOC101927023, EPB41L4A-AS2, LOC102467214, LOC102467216, APC, SRP19, REEP5, DCP2, MCC, TSSK1B, YTHDC2, KCNN2, LOC101927078, LOC101927059, TRIM36, PGGT1B, CCDC112, FEM1C, TICAM2, TMED7-TICAM2, LOC101927100, TMED7, LOC102467217, CDO1, ATG12, AP3S1, LVRN, ARL14EPL, COMMD10, LOC101927190, SEMA6A, SEMA6A-AS1, LOC102467223, LINC00992, LOC102467224, HNCAT21, HRAT56, LOC102467225, DTWD2, MIR1244-1, MIR1244-4, MIR1244-3, MIR1244-2, LOC105379143, DMXL1, MIR5706, TNFAIP8, HSD17B4, FAM170A, PRR16, LOC102467226, FTMT, SRFBP1, LOX, ZNF474, LOC100505841, SNCAIP, MGC32805, LOC101927357, LOC101927379, SNX2, SNX24, PPIC, PRDM6, CEP120, CSNK1G3, LINC01170, ZNF608, LOC101927421, LOC101927460, LOC102546228, LOC101927488, GRAMD3, ALDH7A1, PHAX, TEX43, LOC102723557, LMNB1, MARCH3, C5orf63, MEGF10, PRRC1, CTXN3, CCDC192, LINC01184, SLC12A2, FBN2, SLC27A6, ISOC1, MIR4633, MIR4460, ADAMTS19-AS1, ADAMTS19, KIAA1024L, CHSY3, HINT1, LYRM7, CDC42SE2, RAPGEF6, FNIP1, MEIKIN, ACSL6, IL3, CSF2, P4HA2-AS1, P4HA2, MIR6830, PDLIM4, SLC22A4, LOC553103, MIR3936, SLC22A5, C5orf56, IRF1, IL5, RAD50, TH2LCRR, IL13, IL4, LOC105379176, KIF3A, CCNI2, SEPT8, SOWAHA, SHROOM1, GDF9, UQCRQ, LEAP2, AFF4, ZCCHC10, HSPA4, FSTL4, MIR1289-2, WSPAR, C5orf15, LOC105379183, VDAC1, TCF7, SKP1, PPP2CA, MIR3661, CDKL3, UBE2B, CDKN2AIPNL, LOC102546229, LOC101927934, JADE2, SAR1B, SEC24A, CAMLG, DDX46, C5orf24, TXNDC15, PCBD2, MIR4461, CATSPER3, PITX1, C5orf66, C5orf66-AS1, C5orf66-AS2, H2AFY, DCANP1, TIFAB, NEUROG1, CXCL14, LOC340074, MIR5692C1, SLC25A48, IL9, FBXL21, LECT2, TGFBI, VTRNA2-1, SMAD5-AS1, SMAD5, LOC389332, TRPC7, TRPC7-AS2, MIR4454, SPOCK1, LOC105379192, KLHL3, MIR874, HNRNPA0, NPY6R, MYOT, PKD2L2, FAM13B, LOC100130172, WNT8A, NME5, BRD8, KIF20A, CDC23, GFRA3, CDC25C, FAM53C, KDM3B, REEP2, EGR1, ETF1, HSPA9, SNORD63, LOC105379194, CTNNA1, LRRTM2, SIL1, SNHG4, MATR3, SNORA74A, PAIP2, SLC23A1, MZB1, PROB1, SPATA24, DNAJC18, ECSCR, TMEM173, UBE2D2, CXXC5, LOC101929696, PSD2, NRG2, LINC01024, PURA, IGIP, LOC101929719, CYSTM1, PFDN1, HBEGF, SLC4A9, ANKHD1, ANKHD1-EIF4EBP3, EIF4EBP3, SRA1, APBB3, MIR6831, SLC35A4, CD14, TMCO6, NDUFA2, IK, MIR3655, WDR55, DND1, HARS, HARS2, ZMAT2, VTRNA1-1, VTRNA1-2, VTRNA1-3, PCDHA1, PCDHA2, PCDHA3, PCDHA4, PCDHA5, PCDHA6, PCDHA7, PCDHA8, PCDHA9, PCDHA10, PCDHA11, PCDHA12, PCDHA13, PCDHAC1, PCDHAC2, LOC101926905, PCDHB1, PCDHB2, PCDHB3, PCDHB4, PCDHB5, PCDHB6, PCDHB17P, PCDHB7, PCDHB8, PCDHB16, PCDHB9, PCDHB10, PCDHB11, PCDHB12, PCDHB13, PCDHB14, PCDHB18P, PCDHB19P, PCDHB15, SLC25A2, TAF7, PCDHGA1, PCDHGA2, PCDHGA3, PCDHGB1, PCDHGA4, PCDHGB2, PCDHGA5, PCDHGB3, PCDHGA6, PCDHGA7, PCDHGB4, PCDHGA8, PCDHGB5, PCDHGA9, PCDHGB6, PCDHGA10, PCDHGB7, PCDHGA11, PCDHGB8P, PCDHGA12, PCDHGC3, PCDHGC4, PCDHGC5, DIAPH1, LOC100505658, HDAC3, RELL2, FCHSD1, ARAP3, PCDH1, LOC729080, KIAA0141, PCDH12, RNF14, GNPDA1, NDFIP1, SPRY4, SPRY4-IT1, LOC101926941, FGF1, LOC101926975, ARHGAP26, ARHGAP26-AS1, ARHGAP26-IT1, NR3C1, MIR5197, HMHB1, YIPF5, KCTD16, PRELID2, GRXCR2, SH3RF2, PLAC8L1, LARS, RBM27, POU4F3, TCERG1, GPR151, PPP2R2B, PPP2R2B-IT1, STK32A, DPYSL3, JAKMIP2-AS1, JAKMIP2, SPINK1, SCGB3A2, C5orf46, SPINK5, SPINK14, SPINK6, LOC102546294, SPINK13, SPINK7, SPINK9, FBXO38, HTR4, ADRB2, SH3TC2, LOC255187, ABLIM3, AFAP1L1, GRPEL2, GRPEL2-AS1, PCYOX1L, IL17B, CARMN, MIR143, MIR145, CSNK1A1, ARHGEF37, PPARGC1B, MIR378A, PDE6A, LOC644762, SLC26A2, TIGD6, HMGXB3, CSF1R, PDGFRB, CDX1, SLC6A7, CAMK2A, ARSI, TCOF1, CD74, RPS14, LOC102546298, NDST1, SYNPO, MYOZ3, RBM22, DCTN4, SMIM3, IRGM, ZNF300, ZNF300P1, GPX3, TNIP1, ANXA6, CCDC69, GM2A, SLC36A3, SLC36A2, SLC36A1, FAT2, MIR6499, SPARC, CTB-113P19.1, ATOX1, LOC100652758, G3BP1, GLRA1, CTB-12O2.1, NMUR2, LINC01470, GRIA1, FAM114A2, MFAP3, GALNT10, MIR1294, SAP30L-AS1, SAP30L, HAND1, MIR3141, MIR1303, LARP1, FAXDC2, MIR378H, CNOT8, GEMIN5, MRPL22, KIF4B, SGCD, PPP1R2P3, TIMD4, HAVCR1, HAVCR2, MED7, FAM71B, ITK, CYFIP2, FNDC9, LOC102724404, NIPAL4, ADAM19, SOX30, C5orf52, THG1L, LSM11, CLINT1, LOC101927697, EBF1, LOC101927740, RNF145, LOC105377682, UBLCP1, IL12B, LOC285626, LOC285627, LOC101927766, ADRA1B, TTC1, PWWP2A, FABP6, CCNJL, C1QTNF2, ZBED8, SLU7, PTTG1, MIR3142HG, MIR3142, MIR146A, ATP10B, LOC285629, GABRB2, GABRA6, GABRA1, LINC01202, GABRG2, CCNG1, NUDCD2, HMMR, HMMR-AS1, MAT2B, LOC101927835, LOC102546299, CTB-7E3.1, LOC101927908, TENM2, CTB-178M22.2, WWC1, RARS, FBLL1, PANK3, MIR103A1, MIR103B1, SLIT3, LOC101927969, MIR218-2, LOC728095, MIR585, SPDL1, DOCK2, FAM196B, MIR378E, FOXI1, LINC01187, C5orf58, LCP2, LINC01366, KCNIP1, KCNMB1, CTD-2270F17.1, LOC105377716, GABRP, RANBP17, TLX3, MIR3912, NPM1, FGF18, SMIM23, FBXW11, STK10, EFCAB9, UBTD2, LOC100288254, SH3PXD2B, NEURL1B, MIR5003, LOC101928093, DUSP1, ERGIC1, LOC100268168, RPL26L1, ATP6V0E1, SNORA74B, CREBRF, BNIP1, NKX2-5, STC2, MIR8056, LOC285593, BOD1, LINC01484, LINC01485, CPEB4, C5orf47, HMP19, LINC01411, MSX2, MIR4634, FLJ16171, DRD1, SFXN1, HRH2, CPLX2, THOC3, LOC100996385, FAM153B, LOC100507387, LOC643201, SIMC1, KIAA1191, ARL10, MIR1271, NOP16, HIGD2A, CLTB, FAF2, RNF44, CDHR2, GPRIN1, SNCB, MIR4281, EIF4E1B, TSPAN17, LINC01574, UNC5A, HK3, UIMC1, ZNF346, FGFR4, NSD1, RAB24, PRELID1, MXD3, LMAN2, RGS14, SLC34A1, PFN3, F12, GRK6, PRR7-AS1, PRR7, DBN1, PDLIM7, DOK3, DDX41, FAM193B, TMED9, B4GALT7, LOC202181, FAM153A, LOC728554, PROP1, FAM153C, N4BP3, RMND5B, NHP2, GMCL1P1, HNRNPAB, PHYKPL, COL23A1, CLK4, ZNF354A, AACSP1, ZNF354B, ZFP2, ZNF454, GRM6, ZNF879, ZNF354C, ADAMTS2, RUFY1, LOC101928445, HNRNPH1, C5orf60, LOC105377763, CBY3, CANX, MAML1, LTC4S, MGAT4B, MIR1229, SQSTM1, C5orf45, LOC100996419, TBC1D9B, RNF130, MIR340, RASGEF1C, MAPK9, GFPT2, CNOT6, SCGB3A1, FLT4, OR2Y1, MGAT1, HEIH, LINC00847, ZFP62, BTNL8, BTNL3, BTNL9, MIR8089, OR2V1, OR2V2, LOC102577426, TRIM7, MIR4638, TRIM41, RACK1, SNORD96A, SNORD95, CTC-338M12.4, TRIM52, TRIM52-AS1 | arr[GRCh37] 5q11.2q35.3(53891523_180698312) hmz |
| 1  BM | cn-LOH | 8 | q11.21 | q21.11 | 23,250 | PXDNL, PCMTD1, ST18, LOC101929341, FAM150A, RB1CC1, NPBWR1, OPRK1, ATP6V1H, RGS20, TCEA1, LYPLA1, MRPL15, SOX17, RP1, XKR4, SBF1P1, LOC105375843, TMEM68, TGS1, LYN, RPS20, SNORD54, MOS, PLAG1, CHCHD7, SDR16C5, SDR16C6P, PENK, LOC101929415, LINC00968, IMPAD1, LINC01606, LOC286177, LINC00588, LOC101929488, LOC286178, LINC01602, FAM110B, LOC101929528, UBXN2B, CYP7A1, SDCBP, NSMAF, TOX, CA8, LINC01301, RAB2A, CHD7, LOC100130298, CLVS1, ASPH, MIR4470, NKAIN3, UG0898H09, GGH, TTPA, YTHDF3-AS1, YTHDF3, LOC102724612, LINC01289, LOC102724623, MIR124-2HG, MIR124-2, LOC401463, BHLHE22, CYP7B1, LINC00251, LINC01299, ARMC1, MTFR1, PDE7A, DNAJC5B, TRIM55, CRH, LINC00967, RRS1-AS1, RRS1, ADHFE1, C8orf46, MYBL1, VCPIP1, C8orf44, C8orf44-SGK3, SGK3, PTTG3P, MCMDC2, SNHG6, SNORD87, TCF24, PPP1R42, COPS5, CSPP1, ARFGEF1, LOC102724708, CPA6, PREX2, C8orf34-AS1, C8orf34, LINC01592, LINC01603, SULF1, SLCO5A1, PRDM14, NCOA2, LOC101926892, TRAM1, LACTB2-AS1, LACTB2, XKR9, EYA1, MSC, MSC-AS1, TRPA1, LOC392232, KCNB2, LOC101926908, TERF1, SBSPON, C8orf89, RPL7, RDH10, RDH10-AS1, STAU2-AS1, STAU2, UBE2W, TCEB1, TMEM70, LY96, JPH1 | arr[GRCh37] 8q11.21q21.11(51908365_75157868) hmz |
| 1  BM | cn-LOH | 9 | p24.3 | p13.1 | 38,979 | C9orf66, DOCK8, KANK1, DMRT1, DMRT3, LINC01230, DMRT2, SMARCA2, VLDLR-AS1, VLDLR, KCNV2, PUM3, LINC01231, RFX3, RFX3-AS1, GLIS3, GLIS3-AS1, SLC1A1, SPATA6L, PLPP6, CDC37L1-AS1, CDC37L1, AK3, RCL1, MIR101-2, JAK2, INSL6, INSL4, RLN2, RLN1, PLGRKT, CD274, PDCD1LG2, RIC1, ERMP1, MLANA, KIAA2026, MIR4665, RANBP6, IL33, TPD52L3, UHRF2, GLDC, KDM4C, TMEM261, PTPRD, PTPRD-AS1, LOC105375972, PTPRD-AS2, TYRP1, LURAP1L-AS1, LURAP1L, SNORD137, MPDZ, FLJ41200, LINC00583, NFIB, ZDHHC21, CER1, FREM1, LOC389705, TTC39B, SNAPC3, PSIP1, CCDC171, C9orf92, BNC2, CNTLN, SH3GL2, ADAMTSL1, MIR3152, SAXO1, RRAGA, HAUS6, SCARNA8, PLIN2, DENND4C, RPS6, ACER2, SLC24A2, MLLT3, MIR4473, MIR4474, FOCAD, FOCAD-AS1, MIR491, HACD4, IFNB1, IFNW1, IFNA21, IFNA4, IFNA7, IFNA10, IFNA16, IFNA17, IFNA14, IFNA22P, IFNA5, KLHL9, IFNA6, IFNA13, IFNA2, IFNA8, IFNA1, MIR31HG, IFNE, MIR31, MTAP, CDKN2A-AS1, CDKN2A, CDKN2B-AS1, CDKN2B, DMRTA1, LINC01239, LOC101929563, ELAVL2, IZUMO3, TUSC1, LINC01241, LOC100506422, CAAP1, PLAA, IFT74, IFT74-AS1, LRRC19, TEK, LINC00032, EQTN, MOB3B, IFNK, C9orf72, LINGO2, MIR876, MIR873, LINC01242, LINC01243, ACO1, DDX58, TOPORS, TOPORS-AS1, NDUFB6, TAF1L, TMEM215, APTX, DNAJA1, SMU1, B4GALT1, B4GALT1-AS1, SPINK4, BAG1, CHMP5, NFX1, AQP7, AQP3, NOL6, MIR6851, SUGT1P1, ANKRD18B, ANXA2P2, PTENP1, PTENP1-AS, LINC01251, PRSS3, UBE2R2, UBAP2, SNORD121B, SNORD121A, DCAF12, UBAP1, KIF24, NUDT2, KIAA1161, C9orf24, FAM219A, DNAI1, ENHO, CNTFR, CNTFR-AS1, RPP25L, DCTN3, ARID3C, SIGMAR1, GALT, IL11RA, CCL27, LOC730098, CCL19, CCL21, FAM205A, FAM205BP, FAM205C, PHF24, DNAJB5-AS1, DNAJB5, C9orf131, VCP, FANCG, PIGO, STOML2, FAM214B, UNC13B, ATP8B5P, RUSC2, FAM166B, TESK1, MIR4667, CD72, LOC101926948, SIT1, RMRP, CCDC107, ARHGEF39, CA9, TPM2, TLN1, MIR6852, CREB3, MIR6853, GBA2, RGP1, MSMP, NPR2, SPAG8, HINT2, FAM221B, TMEM8B, LINC00950, OR13J1, HRCT1, LINC00961, OR2S2, RECK, GLIPR2, CCIN, CLTA, GNE, RNF38, MELK, MIR4475, PAX5, MIR4540, MIR4476, EBLN3, ZCCHC7, GRHPR, ZBTB5, POLR1E, FBXO10, TOMM5, FRMPD1, TRMT10B, EXOSC3, DCAF10, SLC25A51, SHB, ALDH1B1, IGFBPL1, FAM95C, ANKRD18A, FAM201A, CNTNAP3 | arr[GRCh37] 9p24.3p13.1(204737_39184065) hmz |
| 1  BM | cn-LOH | 9 | q21.11 | q34.3 | 70,070 | PGM5, TMEM252, LINC01506, PIP5K1B, FAM122A, LOC101927069, PRKACG, FXN, TJP2, BANCR, FAM189A2, APBA1, PTAR1, C9orf135-AS1, C9orf135, MAMDC2, MAMDC2-AS1, SMC5-AS1, SMC5, KLF9, TRPM3, MIR204, TMEM2, ABHD17B, C9orf85, C9orf57, GDA, LINC01504, ZFAND5, TMC1, LINC01474, ALDH1A1, ANXA1, LOC101927358, RORB-AS1, RORB, TRPM6, C9orf40, C9orf41-AS1, CARNMT1, NMRK1, OSTF1, MIR548H3, PCSK5, RFK, RPSAP9, GCNT1, PRUNE2, PCA3, FOXB2, VPS13A-AS1, VPS13A, GNA14, GNA14-AS1, GNAQ, CEP78, PSAT1, LOC101927450, TLE4, LINC01507, TLE1, LOC101927502, SPATA31D5P, SPATA31D4, SPATA31D3, SPATA31D1, RASEF, FRMD3, IDNK, UBQLN1, LOC105376114, GKAP1, KIF27, C9orf64, HNRNPK, MIR7-1, RMI1, LOC101927575, SLC28A3, NTRK2, AGTPBP1, LOC389765, NAA35, GOLM1, LOC101927623, C9orf153, ISCA1, ZCCHC6, GAS1, GAS1RR, LOC440173, LOC494127, C9orf170, DAPK1, CTSL, CTSL3P, CTSLP8, LOC392364, SPATA31E1, SPATA31C1, CDK20, LOC102724156, SPATA31C2, SPIN1, NXNL2, LOC286238, MIR4289, C9orf47, S1PR3, SHC3, CKS2, MIR3153, SECISBP2, SEMA4D, GADD45G, UNQ6494, LOC101927847, MIR4290HG, MIR4290, LINC01508, LINC01501, DIRAS2, SYK, LOC100129316, LINC00484, AUH, NFIL3, MIR3910-1, MIR3910-2, ROR2, SPTLC1, LOC100128076, LINC00475, IARS, MIR3651, SNORA84, NOL8, CENPP, OGN, OMD, ASPN, ECM2, MIR4670, IPPK, LOC100128361, BICD2, LOC101929748, ANKRD19P, ZNF484, LOC642943, FGD3, LOC101927954, SUSD3, CARD19, NINJ1, WNK2, C9orf129, FAM120AOS, FAM120A, PHF2, MIR548AU, MIR4291, BARX1, PTPDC1, MIRLET7A1, MIRLET7F1, MIRLET7DHG, MIRLET7D, ZNF169, NUTM2F, LOC100132077, MFSD14B, PCAT7, FBP2, FBP1, C9orf3, MIR2278, MIR6081, MIR23B, MIR27B, MIR3074, MIR24-1, FANCC, PTCH1, LOC100507346, LINC00476, ERCC6L2, LINC00092, LOC158435, LOC158434, HSD17B3, SLC35D2, ZNF367, HABP4, CDC14B, AAED1, LOC441455, ZNF510, ZNF782, LOC100132781, LOC441454, NUTM2G, MFSD14C, CTSV, GAS2L1P2, ANKRD18CP, LOC100499484, LOC100499484-C9ORF174, CCDC180, MIR1302-8, LOC286359, TDRD7, TMOD1, TSTD2, NCBP1, XPA, FOXE1, TRMO, HEMGN, ANP32B, NANS, TRIM14, CORO2A, TBC1D2, MIR6854, GABBR2, ANKS6, GALNT12, COL15A1, TGFBR1, ALG2, SEC61B, NAMA, LOC101928438, NR4A3, STX17-AS1, STX17, ERP44, INVS, TEX10, MSANTD3, MSANTD3-TMEFF1, TMEFF1, MURC, PLPPR1, BAAT, MRPL50, ZNF189, ALDOB, TMEM246-AS1, TMEM246, RNF20, GRIN3A, PPP3R2, LINC00587, CYLC2, LINC01492, LOC101928523, SMC2-AS1, SMC2, LOC105376194, OR13F1, OR13C4, OR13C3, OR13C8, OR13C5, OR13C2, OR13C9, OR13D1, NIPSNAP3A, NIPSNAP3B, ABCA1, SLC44A1, FSD1L, FKTN, TAL2, TMEM38B, MIR8081, LINC01505, ZNF462, LOC340512, RAD23B, LINC01509, KLF4, ACTL7B, ACTL7A, IKBKAP, FAM206A, CTNNAL1, TMEM245, MIR32, FRRS1L, EPB41L4B, PTPN3, MIR3927, PALM2, PALM2-AKAP2, AKAP2, C9orf152, TXN, TXNDC8, SVEP1, MUSK, LPAR1, MIR7702, OR2K2, KIAA0368, ZNF483, PTGR1, LRRC37A5P, DNAJC25, DNAJC25-GNG10, GNG10, C9orf84, UGCG, MIR4668, SUSD1, PTBP3, HSDL2, KIAA1958, INIP, SNX30, SLC46A2, ZNF883, ZFP37, FAM225B, FAM225A, SLC31A2, FKBP15, SLC31A1, CDC26, PRPF4, RNF183, WDR31, BSPRY, HDHD3, ALAD, POLE3, C9orf43, RGS3, ZNF618, AMBP, KIF12, COL27A1, MIR455, ORM1, ORM2, AKNA, WHRN, ATP6V1G1, TMEM268, LOC100505478, TNFSF15, TNFSF8, TNC, LOC101928748, DEC1, LOC101928775, LINC00474, PAPPA, PAPPA-AS1, ASTN2, ASTN2-AS1, TRIM32, SNORA70C, LOC101928797, TLR4, BRINP1, LINC01613, MIR147A, CDK5RAP2, MEGF9, FBXW2, LOC100288842, PSMD5, PSMD5-AS1, PHF19, TRAF1, C5, CNTRL, RAB14, GSN, GSN-AS1, STOM, GGTA1P, DAB2IP, TTLL11, MIR4478, NDUFA8, MORN5, LHX6, RBM18, MRRF, PTGS1, OR1J1, OR1J2, OR1J4, OR1N1, OR1N2, OR1L8, OR1Q1, OR1B1, OR1L1, OR1L3, OR1L4, OR1L6, OR5C1, OR1K1, PDCL, RC3H2, SNORD90, ZBTB6, ZBTB26, RABGAP1, GPR21, MIR600HG, MIR600, STRBP, CRB2, DENND1A, MIR601, MIR7150, LOC100505588, LHX2, NEK6, PSMB7, LOC100129034, NR5A1, NR6A1, MIR181A2HG, MIR181A2, MIR181B2, OLFML2A, WDR38, RPL35, ARPC5L, GOLGA1, SCAI, PPP6C, LOC105376271, RABEPK, HSPA5, GAPVD1, MAPKAP1, LOC51145, PBX3, LOC101929116, MVB12B, NRON, LMX1B, ZBTB43, ZBTB34, RALGPS1, ANGPTL2, GARNL3, SLC2A8, ZNF79, RPL12, SNORA65, LRSAM1, FAM129B, STXBP1, MIR3911, CFAP157, PTRH1, TTC16, TOR2A, SH2D3C, MIR3960, MIR2861, CDK9, FPGS, ENG, LOC102723566, AK1, MIR4672, ST6GALNAC6, ST6GALNAC4, PIP5KL1, DPM2, FAM102A, NAIF1, SLC25A25, SLC25A25-AS1, PTGES2, PTGES2-AS1, LCN2, C9orf16, CIZ1, DNM1, MIR199B, MIR3154, GOLGA2, SWI5, TRUB2, COQ4, SLC27A4, MIR1268A, URM1, MIR219A2, MIR219B, CERCAM, ODF2, GLE1, SPTAN1, WDR34, SET, PKN3, ZDHHC12, LOC100506100, ZER1, TBC1D13, ENDOG, C9orf114, KYAT1, LRRC8A, PHYHD1, DOLK, NUP188, SH3GLB2, MIGA2, DOLPP1, CRAT, PTPA, IER5L, LOC101929331, C9orf106, LINC01503, LINC00963, NTMT1, C9orf50, ASB6, PRRX2, PRRX2-AS1, PTGES, TOR1B, TOR1A, C9orf78, USP20, MIR6855, FNBP1, GPR107, LOC401554, NCS1, HMCN2, ASS1, LOC100272217, FUBP3, MIR6856, PRDM12, EXOSC2, ABL1, QRFP, FIBCD1, LAMC3, AIF1L, NUP214, FAM78A, PLPP7, PRRC2B, SNORD62B, SNORD62A, POMT1, UCK1, RAPGEF1, MED27, NTNG2, SETX, TTF1, CFAP77, BARHL1, DDX31, GTF3C4, AK8, SPACA9, TSC1, GFI1B, MIR548AW, SNORD141B, SNORD141A, LOC105376306, GTF3C5, MIR6877, CEL, CELP, RALGDS, GBGT1, OBP2B, ABO, SURF6, MED22, RPL7A, SNORD24, SNORD36B, SNORD36A, SNORD36C, SURF1, SURF2, SURF4, STKLD1, REXO4, ADAMTS13, CACFD1, SLC2A6, TMEM8C, ADAMTSL2, FAM163B, DBH, DBH-AS1, SARDH, VAV2, LINC00094, BRD3, LOC100130548, WDR5, RNU6ATAC, RXRA, MIR4669, COL5A1, LOC101448202, MIR3689A, MIR3689C, MIR3689D1, MIR3689B, MIR3689D2, MIR3689E, MIR3689F, FCN2, FCN1, OLFM1, LOC401557, C9orf62, PPP1R26-AS1, PPP1R26, C9orf116, MRPS2, LOC101928525, LCN1, OBP2A, PAEP, LINC01502, GLT6D1, LCN9, SOHLH1, KCNT1, CAMSAP1, UBAC1, NACC2, C9orf69, LHX3, QSOX2, DKFZP434A062, GPSM1, DNLZ, CARD9, SNAPC4, SDCCAG3, PMPCA, INPP5E, SEC16A, C9orf163, NOTCH1, MIR4673, MIR4674, NALT1, LINC01451, HSPC324, EGFL7, MIR126, AGPAT2, FAM69B, SNHG7, SNORA17B, SNORA17A, LCN10, LCN6, LOC100128593, MIR6722, LCN8, LCN15, TMEM141, CCDC183, CCDC183-AS1, RABL6, MIR4292, C9orf172, PHPT1, MAMDC4, EDF1, TRAF2, MIR4479, FBXW5, C8G, LCN12, PTGDS, LCNL1, C9orf142, CLIC3, ABCA2, C9orf139, FUT7, NPDC1, ENTPD2, SAPCD2, UAP1L1, MAN1B1-AS1, MAN1B1, DPP7, GRIN1, LRRC26, MIR3621, TMEM210, ANAPC2, SSNA1, TPRN, TMEM203, NDOR1, RNF208, CYSRT1, RNF224, SLC34A3, TUBB4B, FAM166A, C9orf173-AS1, STPG3, NELFB, TOR4A, NRARP, EXD3, NOXA1, ENTPD8, NSMF, MIR7114, PNPLA7, MRPL41, DPH7, ZMYND19, ARRDC1, ARRDC1-AS1, EHMT1, EHMT1-IT1, MIR602, LOC100133077, CACNA1B, LOC105376331, LOC101928786, TUBBP5 | arr[GRCh37] 9q21.11q34.3(70984371_141054761) hmz |
| 1  BM | cn-LOH | 10 | p15.3 | p14 | 10,938 | ZMYND11, DIP2C, MIR7641-2, MIR5699, PRR26, LARP4B, LOC101927762, GTPBP4, IDI2, IDI2-AS1, IDI1, WDR37, LINC00200, ADARB2, ADARB2-AS1, LINC00700, MIR6072, LINC00701, LOC105376351, PFKP, SNORD142, PITRM1, PITRM1-AS1, LOC105376360, KLF6, LOC105376365, MIR6078, LOC101927964, LINC00702, LINC00703, LINC00704, LINC00705, AKR1E2, AKR1C6P, AKR1C1, AKR1C2, AKR1C3, AKR1C8P, AKR1C4, UCN3, TUBAL3, NET1, CALML5, CALML3-AS1, CALML3, LOC105376382, ASB13, FAM208B, GDI2, ANKRD16, FBXO18, IL15RA, IL2RA, RBM17, PFKFB3, MIR3155A, MIR3155B, LOC399715, PRKCQ, PRKCQ-AS1, LOC101928150, MIR4454, LINC00706, LINC00707, SFMBT2, SNORD129, ITIH5, ITIH2, KIN, ATP5C1, TAF3, GATA3-AS1, GATA3, LINC00708, LOC105376398, LOC105755953, LOC101928272, LINC00709, LOC101928298, LOC101928322, CELF2, SFTA1P, LINC00710 | arr[GRCh37] 10p15.3p14(126069_11064241) hmz |
| 1  BM | cn-LOH | 17 | p13.3 | p11.2 | 20,348 | VPS53, FAM57A, GEMIN4, DBIL5P, GLOD4, MRM3, NXN, LOC101927727, TIMM22, ABR, MIR3183, BHLHA9, TUSC5, YWHAE, CRK, MYO1C, INPP5K, PITPNA-AS1, PITPNA, SLC43A2, SCARF1, RILP, PRPF8, TLCD2, MIR22HG, MIR22, WDR81, SERPINF2, SERPINF1, SMYD4, RPA1, RTN4RL1, LOC105371485, DPH1, OVCA2, MIR132, MIR212, HIC1, SMG6, LOC101927839, SRR, TSR1, SNORD91B, SNORD91A, SGSM2, MNT, LOC284009, METTL16, PAFAH1B1, CLUH, MIR6776, LOC105371592, MIR1253, RAP1GAP2, LOC101927911, OR1D5, OR1D2, OR1G1, OR1A2, OR1A1, OR1D4, OR3A2, OR3A1, OR3A4P, OR1E1, OR3A3, OR1E2, SPATA22, ASPA, TRPV3, TRPV1, SHPK, CTNS, TAX1BP3, P2RX5-TAX1BP3, EMC6, P2RX5, ITGAE, GSG2, NCBP3, CAMKK1, P2RX1, ATP2A3, ZZEF1, CYB5D2, ANKFY1, UBE2G1, LOC103021295, SPNS3, SPNS2, MYBBP1A, GGT6, SMTNL2, ALOX15, PELP1, LOC101559451, ARRB2, MED11, CXCL16, ZMYND15, TM4SF5, VMO1, GLTPD2, PSMB6, PLD2, MINK1, CHRNE, C17orf107, GP1BA, SLC25A11, RNF167, PFN1, ENO3, SPAG7, CAMTA2, MIR6864, MIR6865, INCA1, KIF1C, LOC102724009, SLC52A1, ZFP3, ZNF232, LOC101928000, USP6, ZNF594, LOC100130950, SCIMP, RABEP1, NUP88, RPAIN, C1QBP, DHX33, LOC105371506, DERL2, MIS12, LOC728392, NLRP1, LOC339166, WSCD1, AIPL1, FAM64A, PITPNM3, KIAA0753, TXNDC17, MED31, C17orf100, MIR4520-1, MIR4520-2, ALOX15P1, SLC13A5, XAF1, FBXO39, TEKT1, ALOX12P2, ALOX12-AS1, ALOX12, RNASEK, RNASEK-C17orf49, C17orf49, MIR497HG, MIR195, MIR497, BCL6B, SLC16A13, SLC16A11, CLEC10A, ASGR2, ASGR1, DLG4, ACADVL, MIR324, DVL2, PHF23, GABARAP, CTDNEP1, ELP5, CLDN7, SLC2A4, YBX2, EIF5A, GPS2, NEURL4, ACAP1, KCTD11, TMEM95, TNK1, PLSCR3, TMEM256-PLSCR3, TMEM256, NLGN2, SPEM1, C17orf74, TMEM102, FGF11, CHRNB1, ZBTB4, SLC35G6, POLR2A, TNFSF12, TNFSF12-TNFSF13, TNFSF13, SENP3, SENP3-EIF4A1, EIF4A1, SNORA48, SNORD10, SNORA67, CD68, LOC100996842, MPDU1, SOX15, FXR2, SHBG, SAT2, ATP1B2, TP53, WRAP53, EFNB3, DNAH2, RPL29P2, KDM6B, TMEM88, NAA38, CYB5D1, CHD3, SCARNA21, LOC284023, KCNAB3, TRAPPC1, CNTROB, GUCY2D, ALOX15B, ALOX12B, MIR4314, ALOXE3, HES7, PER1, MIR6883, VAMP2, TMEM107, MIR4521, BORCS6, AURKB, LINC00324, CTC1, PFAS, SLC25A35, RANGRF, ARHGEF15, ODF4, LOC100128288, KRBA2, RPL26, RNF222, NDEL1, MYH10, CCDC42, SPDYE4, MFSD6L, PIK3R6, PIK3R5, NTN1, LOC101928266, STX8, CFAP52, USP43, DHRS7C, GSG1L2, GLP2R, RCVRN, GAS7, MYH13, MYHAS, MYH8, MYH4, MYH1, MYH2, MYH3, SCO1, ADPRM, TMEM220, MAGOH2P, TMEM220-AS1, LINC00675, PIRT, SHISA6, DNAH9, ZNF18, MAP2K4, MIR744, LINC00670, MYOCD, LOC101928418, LOC100128006, ARHGAP44, MIR1269B, ELAC2, HS3ST3A1, CDRT15P1, COX10-AS1, COX10, CDRT15, HS3ST3B1, MGC12916, LOC101928475, CDRT7, CDRT8, PMP22, MIR4731, TEKT3, CDRT4, TVP23C-CDRT4, TVP23C, CDRT1, TRIM16, ZNF286A, TBC1D26, CDRT15P2, MEIS3P1, LOC101928567, ADORA2B, ZSWIM7, TTC19, NCOR1, PIGL, MIR1288, CENPV, UBB, TRPV2, LRRC75A-AS1, SNORD49B, SNORD49A, SNORD65, LRRC75A, ZNF287, ZNF624, CCDC144A, USP32P1, FAM106CP, KRT16P2, TNFRSF13B, MPRIP, PLD6, FLCN, COPS3, NT5M, MED9, RASD1, PEMT, SMCR2, RAI1, RAI1-AS1, SMCR5, SREBF1, MIR6777, MIR33B, TOM1L2, DRC3, ATPAF2, GID4, DRG2, MYO15A, ALKBH5, LLGL1, FLII, MIEF2, TOP3A, SMCR8, SHMT1, MIR6778, EVPLL, FLJ35934, KRT17P5, KRT16P1, LGALS9C, USP32P2, FAM106A, CCDC144B, TBC1D28, ZNF286B, FOXO3B, TRIM16L, FBXW10, TVP23B, PRPSAP2, SLC5A10, FAM83G, GRAP, LOC79999, LOC388436, GRAPL, EPN2, EPN2-IT1, EPN2-AS1, B9D1, MIR1180, MAPK7, MFAP4, RNF112, SLC47A1, SNORA59B, SNORA59A, ALDH3A2, SLC47A2, ALDH3A1, ULK2, AKAP10, SPECC1, CCDC144CP, FAM106B, LGALS9B, KRT16P3, CDRT15L2, LOC100287072 | arr[GRCh37] 17p13.3p11.2(400958_20749243) hmz |
| 1  BM | cn-LOH | 17 | q24.3 | q25.2 | 6,328 | CASC17, LOC102723505, LINC01152, LOC102723517, SOX9-AS1, LOC101928205, SOX9, LOC146795, LINC00673, LINC00511, SLC39A11, SSTR2, COG1, FAM104A, C17orf80, CPSF4L, CDC42EP4, SDK2, LOC101928251, LOC100134391, LINC00469, LOC400620, RPL38, MGC16275, TTYH2, DNAI2, KIF19, BTBD17, GPR142, GPRC5C, CD300A, CD300LB, CD300C, LOC100130520, CD300LD, C17orf77, CD300E, RAB37, CD300LF, SLC9A3R1, MIR3615, NAT9, TMEM104, GRIN2C, FDXR, FADS6, USH1G, OTOP2, OTOP3, HID1, HID1-AS1, CDR2L, MRPL58, KCTD2, ATP5H, SLC16A5, ARMC7, NT5C, HN1, SUMO2, NUP85, GGA3, MRPS7, MIF4GD, LOC100287042, SLC25A19, GRB2, MIR3678, TMEM94, MIR6785, CASKIN2, TSEN54, LLGL2, MYO15B, RECQL5, SMIM5, SMIM6, SAP30BP, ITGB4, GALK1, H3F3B, MIR4738, UNK, UNC13D, WBP2, TRIM47, TRIM65, MRPL38, FBF1, ACOX1, TEN1, TEN1-CDK3, CDK3, MIR4538, EVPL, SRP68, GALR2, ZACN, EXOC7, MIR6868, FOXJ1, RNF157-AS1, RNF157, UBALD2, QRICH2, PRPSAP1, SPHK1, UBE2O, AANAT, RHBDF2, CYGB, PRCD, SNHG16, SNORD1C, SNORD1B, SNORD1A, ST6GALNAC2, ST6GALNAC1, LOC105274304, MXRA7, JMJD6, METTL23, SRSF2, MIR636, MFSD11, LOC101928514, LINC00868, MGAT5B, LOC105371899 | arr[GRCh37] 17q24.3q25.2(68641402_74968958) hmz |
| 1  BM | cn-LOH | 18 | p11.32 | q23 | 77,995 | LOC102723376, ROCK1P1, MIR8078, USP14, THOC1, COLEC12, LOC105376854, CETN1, CLUL1, TYMSOS, TYMS, ENOSF1, YES1, ADCYAP1, LINC00470, METTL4, NDC80, CBX3P2, SMCHD1, EMILIN2, LPIN2, LOC727896, MYOM1, MYL12A, LOC104968399, MYL12B, TGIF1, GAPLINC, DLGAP1, DLGAP1-AS1, DLGAP1-AS2, DLGAP1-AS3, MIR6718, DLGAP1-AS4, DLGAP1-AS5, AKAIN1, LINC00526, LINC00667, ZBTB14, EPB41L3, MIR3976HG, MIR3976, TMEM200C, L3MBTL4, L3MBTL4-AS1, MIR4317, LINC01387, LOC101927168, ARHGAP28, LINC00668, LAMA1, LOC101927188, LRRC30, PTPRM, LOC100192426, RAB12, GACAT2, MTCL1, NDUFV2, NDUFV2-AS1, ANKRD12, TWSG1, RALBP1, PPP4R1, PPP4R1-AS1, RAB31, TXNDC2, VAPA, LINC01254, APCDD1, NAPG, LOC101927410, PIEZO2, MIR6788, LINC01255, SLC35G4, MIR7153, GNAL, CHMP1B, MPPE1, IMPA2, ANKRD62, C18orf61, CIDEA, TUBB6, AFG3L2, PRELID3A, LOC105371998, SPIRE1, PSMG2, CEP76, LOC100996324, PTPN2, SEH1L, CEP192, LDLRAD4, LDLRAD4-AS1, MIR5190, MIR4526, FAM210A, RNMT, MC5R, MC2R, ZNF519, ANKRD20A5P, CYP4F35P, CXADRP3, POTEC, ANKRD30B, MIR3156-2, LINC01443, LINC01444, LOC644669, ROCK1, GREB1L, ESCO1, SNRPD1, ABHD3, MIR320C1, MIB1, MIR133A1HG, MIR133A1, MIR1-2, GATA6-AS1, GATA6, CTAGE1, LOC101927571, RBBP8, MIR4741, CABLES1, TMEM241, RIOK3, C18orf8, NPC1, ANKRD29, LAMA3, TTC39C, TTC39C-AS1, CABYR, OSBPL1A, MIR320C2, IMPACT, HRH4, LOC729950, LOC105372028, ZNF521, SS18, PSMA8, TAF4B, LINC01543, KCTD1, MIR8057, PCAT18, AQP4, AQP4-AS1, CHST9, LOC105372038, CDH2, MIR302F, DSC3, DSC2, DSCAS, DSC1, DSG1, DSG1-AS1, DSG4, DSG3, DSG2, DSG2-AS1, TTR, B4GALT6, SLC25A52, TRAPPC8, RNF125, RNF138, MEP1B, GAREM1, WBP11P1, KLHL14, CCDC178, ASXL3, NOL4, DTNA, MAPRE2, ZNF397, ZSCAN30, ZNF271P, ZNF24, ZNF396, INO80C, MIR3975, GALNT1, MIR187, MIR3929, C18orf21, RPRD1A, SLC39A6, ELP2, LOC101927809, MOCOS, FHOD3, LOC105372071, TPGS2, KIAA1328, LOC105372069, CELF4, LOC105372068, SNORA111, MIR4318, MIR924HG, MIR924, MIR5583-2, MIR5583-1, LINC01477, KC6, PIK3C3, LINC00907, RIT2, SYT4, LINC01478, LOC105667213, SETBP1, MIR4319, SLC14A2, SLC14A2-AS1, SLC14A1, SIGLEC15, EPG5, PSTPIP2, ATP5A1, HAUS1, C18orf25, RNF165, LOXHD1, ST8SIA5, PIAS2, KATNAL2, TCEB3CL, TCEB3CL2, TCEB3C, TCEB3B, HDHD2, IER3IP1, SKOR2, MIR4527, SMAD2, ZBTB7C, CTIF, MIR4743, SMAD7, DYM, MIR4744, C18orf32, RPL17-C18orf32, MIR1539, RPL17, SNORD58C, SNORD58A, SNORD58B, LIPG, ACAA2, SCARNA17, SNHG22, MYO5B, MIR4320, CFAP53, MBD1, CXXC1, SKA1, MAPK4, MRO, ME2, ELAC1, SMAD4, MEX3C, LINC01630, DCC, MIR4528, LOC102724651, LOC101928167, MBD2, SNORA37, POLI, STARD6, C18orf54, DYNAP, RAB27B, CCDC68, LOC101927229, TCF4, TCF4-AS1, MIR4529, LINC01416, LINC01539, TXNL1, WDR7, LINC-ROR, BOD1L2, ST8SIA3, ONECUT2, FECH, NARS, LOC100505549, ATP8B1, NEDD4L, MIR122, MIR3591, ALPK2, SNORA108, LOC101927322, MALT1, ZNF532, OACYLP, SEC11C, GRP, RAX, CPLX4, LMAN1, CCBE1, PMAIP1, MC4R, CDH20, LINC01544, RNF152, PIGN, KIAA1468, TNFRSF11A, ZCCHC2, PHLPP1, BCL2, KDSR, VPS4B, SERPINB5, SERPINB12, SERPINB13, SERPINB4, SERPINB3, SERPINB11, SERPINB7, SERPINB2, SERPINB10, HMSD, SERPINB8, LINC00305, LOC284294, LINC01538, CDH7, CDH19, MIR5011, DSEL, LOC643542, TMX3, CCDC102B, DOK6, LOC105372179, CD226, RTTN, SOCS6, LOC101927481, LOC101060542, GTSCR1, LINC01541, LOC102724913, CBLN2, NETO1, MIR548AV, LOC100505797, LOC400655, LOC100505817, FBXO15, TIMM21, CYB5A, C18orf63, LOC101927606, FAM69C, CNDP2, CNDP1, LINC00909, ZNF407, ZADH2, TSHZ1, SMIM21, LOC100505853, LOC339298, ZNF516, LOC101927989, C18orf65, LINC00908, LINC00683, LOC101927651, LOC400661, LOC100131655, ZNF236, MBP, GALR1, LINC01029, SALL3, ATP9B, NFATC1, LOC284241, CTDP1, KCNG2, PQLC1, HSBP1L1, TXNL4A, RBFA, RBFADN, ADNP2, PARD6G-AS1, PARD6G | arr[GRCh37] 18p11.32q23(12841_78007784) hmz |
| 1  BM | cn-LOH | 21 | q21.2 | q22.3 | 23,954 | MIR6130, D21S2088E, LOC101927869, LOC105372751, LOC339622, LINC00158, MIR155HG, MIR155, LINC00515, MRPL39, JAM2, ATP5J, GABPA, APP, CYYR1-AS1, CYYR1, ADAMTS1, ADAMTS5, MIR4759, LINC00113, LINC00314, LOC284825, LOC101927973, LINC00161, N6AMT1, LTN1, RWDD2B, USP16, CCT8, MAP3K7CL, LINC00189, BACH1, BACH1-IT2, GRIK1, GRIK1-AS2, GRIK1-AS1, CLDN17, LINC00307, CLDN8, KRTAP24-1, KRTAP25-1, KRTAP26-1, KRTAP27-1, KRTAP23-1, KRTAP13-2, MIR4327, KRTAP13-1, KRTAP13-3, KRTAP13-4, KRTAP15-1, KRTAP19-1, KRTAP19-2, KRTAP19-3, KRTAP19-4, KRTAP19-5, KRTAP19-6, KRTAP19-7, KRTAP22-2, KRTAP6-3, KRTAP6-2, KRTAP22-1, KRTAP6-1, KRTAP20-1, KRTAP20-4, KRTAP20-2, KRTAP20-3, KRTAP21-3, KRTAP21-2, KRTAP21-1, KRTAP8-1, KRTAP7-1, KRTAP11-1, KRTAP19-8, TIAM1, LOC150051, SOD1, SCAF4, HUNK, LINC00159, MIS18A, MRAP, URB1, SNORA80A, URB1-AS1, EVA1C, TCP10L, C21orf59, SYNJ1, PAXBP1-AS1, PAXBP1, C21orf62-AS1, C21orf62, LOC102724502, OLIG2, LINC00945, OLIG1, LOC101928107, LINC01548, IFNAR2, IL10RB-AS1, IL10RB, IFNAR1, IFNGR2, TMEM50B, DNAJC28, GART, SON, MIR6501, DONSON, CRYZL1, ITSN1, ATP5O, LINC00649, LOC101928126, SLC5A3, MRPS6, LINC00310, KCNE2, SMIM11A, SMIM11B, C21orf140, KCNE1, RCAN1, CLIC6, LINC00160, LINC01426, RUNX1, RUNX1-IT1, LOC100506403, MIR802, LOC101928269, LINC01436, SETD4, LOC100133286, CBR1, CBR3-AS1, CBR3, DOPEY2, MORC3, CHAF1B, CLDN14, SIM2, HLCS, RIPPLY3, LOC105372795, PIGP, TTC3, DSCR9, DSCR3, DYRK1A, KCNJ6, DSCR4, DSCR8, DSCR10, KCNJ15, LINC01423, ERG, LINC00114, ETS2, LOC101928398, LOC400867, LOC101928435, PSMG1, BRWD1, BRWD1-IT2, BRWD1-AS1, HMGN1, WRB-SH3BGR, WRB, LCA5L, SH3BGR, MIR6508, B3GALT5-AS1, B3GALT5, IGSF5, PCP4, DSCAM, MIR4760, DSCAM-AS1, DSCAM-IT1, LINC00323, MIR3197, BACE2, PLAC4, FAM3B, MX2, MX1, TMPRSS2, LINC00111, LINC00479, LINC00112, RIPK4, MIR6814, PRDM15, C2CD2, SNORA91, ZBTB21, ZNF295-AS1, UMODL1, UMODL1-AS1, ABCG1, TFF3, TFF2, TFF1, TMPRSS3, UBASH3A, RSPH1, LOC101930094, SLC37A1, LOC101928233, PDE9A, LOC101928311, WDR4, NDUFV3, ERVH48-1, MIR5692B, PKNOX1, CBS, CBSL, U2AF1, U2AF1L5, LOC106780825, FRGCA, CRYAA, LOC102724652, LINC00322, TCONS_00029157, LOC102724428, SIK1, LINC00319, LOC102724354, LINC00313, HSF2BP, MIR6070, RRP1B, PDXK, CSTB, RRP1, AATBC, AGPAT3, TRAPPC10, PWP2, C21orf33, ICOSLG, DNMT3L, LOC105372833, AIRE, PFKL, C21orf2, TRPM2, TRPM2-AS, LRRC3-AS1, LRRC3, TSPEAR, TSPEAR-AS1, TSPEAR-AS2, KRTAP10-1, KRTAP10-2, KRTAP10-3, KRTAP10-4, KRTAP10-5, KRTAP10-6, KRTAP10-7, KRTAP10-8, KRTAP10-9, KRTAP10-10, KRTAP10-11, KRTAP12-4, KRTAP12-3, KRTAP12-2, KRTAP12-1, KRTAP10-12, UBE2G2, LINC01424, SUMO3, PTTG1IP, ITGB2, ITGB2-AS1, LINC01547, FAM207A, LINC00163, PICSAR, SSR4P1, ADARB1, LINC00334, POFUT2, LOC642852, LINC00316, COL18A1, COL18A1-AS2, COL18A1-AS1, MIR6815, SLC19A1, LOC100129027, PCBP3, LOC101928796, COL6A1, COL6A2, FTCD, SPATC1L, LSS, MCM3AP-AS1, MCM3AP, YBEY, C21orf58, PCNT, DIP2A, DIP2A-IT1, S100B, PRMT2 | arr[GRCh37] 21q21.2q22.3(24143817_48097610) hmz |
| 2  CRC | Gain | 2 | q33.1 | q33.3 | 7,987 | PLCL1, LOC101927619, SATB2, SATB2-AS1, LOC101927641, FTCDNL1, C2orf69, TYW5, C2orf47, SPATS2L, KCTD18, SGO2, AOX1, AOX3P-AOX2P, LOC100507140, LOC101927795, BZW1, CLK1, PPIL3, NIF3L1, ORC2, FAM126B, NDUFB3, CFLAR, CFLAR-AS1, CASP10, CASP8, ALS2CR12, TRAK2, STRADB, ALS2CR11, TMEM237, MPP4, ALS2, CDK15, FZD7, KIAA2012, LOC729224, SUMO1, NOP58, SNORD70, SNORD11B, SNORD11, BMPR2, FAM117B, ICA1L, WDR12, CARF, NBEAL1, CYP20A1, ABI2, RAPH1, CD28, CTLA4, ICOS, PARD3B, NRP2 | arr[GRCh37] 2q33.1q33.3(198664026_206651315)x3 |
| 2  CRC | Gain | 5 | q34 | q35.3 | 16,225 | CTB-7E3.1, LOC101927908, TENM2, CTB-178M22.2, WWC1, RARS, FBLL1, PANK3, MIR103A1, MIR103B1, SLIT3, LOC101927969, MIR218-2, LOC728095, MIR585, SPDL1, DOCK2, FAM196B, MIR378E, FOXI1, LINC01187, C5orf58, LCP2, LINC01366, KCNIP1, KCNMB1, CTD-2270F17.1, LOC105377716, GABRP, RANBP17, TLX3, MIR3912, NPM1, FGF18, SMIM23, FBXW11, STK10, EFCAB9, UBTD2, LOC100288254, SH3PXD2B, NEURL1B, MIR5003, LOC101928093, DUSP1, ERGIC1, LOC100268168, RPL26L1, ATP6V0E1, SNORA74B, CREBRF, BNIP1, NKX2-5, STC2, MIR8056, LOC285593, BOD1, LINC01484, LINC01485, CPEB4, C5orf47, HMP19, LINC01411, MSX2, MIR4634, FLJ16171, DRD1, SFXN1, HRH2, CPLX2, THOC3, LOC100996385, FAM153B, LOC100507387, LOC643201, SIMC1, KIAA1191, ARL10, MIR1271, NOP16, HIGD2A, CLTB, FAF2, RNF44, CDHR2, GPRIN1, SNCB, MIR4281, EIF4E1B, TSPAN17, LINC01574, UNC5A, HK3, UIMC1, ZNF346, FGFR4, NSD1, RAB24, PRELID1, MXD3, LMAN2, RGS14, SLC34A1, PFN3, F12, GRK6, PRR7-AS1, PRR7, DBN1, PDLIM7, DOK3, DDX41, FAM193B, TMED9, B4GALT7, LOC202181, FAM153A, LOC728554, PROP1, FAM153C, N4BP3, RMND5B, NHP2, GMCL1P1, HNRNPAB, PHYKPL, COL23A1, CLK4, ZNF354A, AACSP1, ZNF354B, ZFP2, ZNF454, GRM6, ZNF879, ZNF354C, ADAMTS2, RUFY1, LOC101928445, HNRNPH1, C5orf60, LOC105377763, CBY3, CANX, MAML1, LTC4S, MGAT4B, MIR1229, SQSTM1, C5orf45, LOC100996419, TBC1D9B, RNF130, MIR340, RASGEF1C, MAPK9, GFPT2, CNOT6, SCGB3A1, FLT4, OR2Y1, MGAT1, HEIH, LINC00847, ZFP62, BTNL8, BTNL3, BTNL9, MIR8089, OR2V1, OR2V2, LOC102577426, TRIM7, MIR4638, TRIM41, RACK1, SNORD96A, SNORD95, CTC-338M12.4, TRIM52, TRIM52-AS1 | arr[GRCh37] 5q34q35.3(164473642_180698312)x3 |
| 2  CRC | Gain | 7 | p22.3 | q36.3 | 159,077 | LOC102723672, LOC100507642, LOC105375115, FAM20C, WI2-2373I1.2, LOC442497, PDGFA, HRAT92, PRKAR1B, LOC101927000, LOC101926963, DNAAF5, SUN1, GET4, ADAP1, COX19, CYP2W1, C7orf50, MIR339, GPR146, GPER1, ZFAND2A, LOC101927021, UNCX, MICALL2, INTS1, MAFK, TMEM184A, PSMG3, PSMG3-AS1, TFAMP1, ELFN1, ELFN1-AS1, MAD1L1, MIR4655, MRM2, NUDT1, SNX8, MIR6836, EIF3B, CHST12, LOC101927181, GRIFIN, LFNG, MIR4648, BRAT1, IQCE, TTYH3, AMZ1, GNA12, CARD11, LOC100129603, SDK1, FOXK1, AP5Z1, MIR4656, RADIL, PAPOLB, MMD2, RNF216P1, RBAK, RBAK-RBAKDN, RBAKDN, ZNF890P, WIPI2, SLC29A4, TNRC18, FBXL18, MIR589, LOC221946, ACTB, FSCN1, RNF216, RNF216-IT1, MIR6874, ZNF815P, OCM, CCZ1, RSPH10B2, RSPH10B, PMS2, AIMP2, EIF2AK1, ANKRD61, USP42, CYTH3, FAM220A, RAC1, DAGLB, KDELR2, GRID2IP, ZDHHC4, C7orf26, ZNF853, ZNF316, ZNF12, PMS2CL, CCZ1B, MIR3683, LOC100131257, C1GALT1, LOC101927354, COL28A1, LOC101927391, MIOS, RPA3, UMAD1, LOC100505921, GLCCI1, ICA1, LOC100505938, NXPH1, PER4, NDUFA4, PHF14, THSD7A, TMEM106B, VWDE, LOC102725191, SCIN, ARL4A, ETV1, DGKB, AGMO, MEOX2, LOC105375166, MEOX2-AS1, ISPD, ISPD-AS1, SOSTDC1, LRRC72, ANKMY2, BZW2, TSPAN13, AGR2, AGR3, AHR, KCCAT333, LOC101927630, SNX13, PRPS1L1, HDAC9, MIR1302-6, TWIST1, FERD3L, TWISTNB, MIR3146, TMEM196, LOC101927668, MACC1, MACC1-AS1, LOC100506098, LOC101927769, LOC101927811, ITGB8, ABCB5, SP8, RPL23P8, LINC01162, SP4, MIR1183, DNAH11, CDCA7L, RAPGEF5, STEAP1B, LOC100506178, LOC401312, LOC541472, IL6, TOMM7, SNORD93, FAM126A, KLHL7-AS1, KLHL7, NUPL2, GPNMB, MALSU1, IGF2BP3, RPS2P32, TRA2A, CLK2P1, CCDC126, FAM221A, STK31, NPY, MPP6, DFNA5, OSBPL3, CYCS, C7orf31, NPVF, MIR148A, NFE2L3, HNRNPA2B1, CBX3, SNX10, LOC105375304, LOC441204, KIAA0087, C7orf71, SKAP2, HOXA1, HOTAIRM1, HOXA2, HOXA3, HOXA-AS2, HOXA4, HOXA-AS3, HOXA5, HOXA6, HOXA7, HOXA9, HOXA10-HOXA9, HOXA10-AS, MIR196B, HOXA10, HOXA11, HOXA11-AS, HOXA13, HOTTIP, EVX1-AS, EVX1, HIBADH, TSL, TAX1BP1, JAZF1, JAZF1-AS1, CREB5, TRIL, LOC100506497, CPVL, LOC101928168, CHN2, LOC102724484, PRR15, LOC646762, MIR550A3, ZNRF2P2, DPY19L2P3, WIPF3, SCRN1, FKBP14, PLEKHA8, MTURN, LOC105375218, ZNRF2, MIR550B1, MIR550A1, DKFZP586I1420, LINC01176, NOD1, GGCT, LOC401320, GARS, CRHR2, INMT, INMT-FAM188B, FAM188B, AQP1, GHRHR, ADCYAP1R1, NEUROD6, CCDC129, PPP1R17, PDE1C, LOC100130673, LSM5, AVL9, DPY19L1P1, ZNRF2P1, MIR550B2, MIR550A2, LINC00997, DPY19L1P2, KBTBD2, RP9P, FKBP9, NT5C3A, RP9, BBS9, BMPER, NPSR1-AS1, NPSR1, DPY19L1, DPY19L2P1, TBX20, LOC401324, HERPUD2, LOC101930085, LOC100506725, SEPT7-AS1, SEPT7, LOC101928618, EEPD1, KIAA0895, ANLN, AOAH, AOAH-IT1, ELMO1, MIR1200, ELMO1-AS1, GPR141, NME8, SFRP4, EPDR1, STARD3NL, TARP, TRG-AS1, AMPH, FAM183BP, VPS41, POU6F2, POU6F2-AS1, YAE1D1, RALA, LINC00265, CDK13, MPLKIP, SUGCT, LINC01450, LINC01449, INHBA, INHBA-AS1, GLI3, LINC01448, C7orf25, PSMA2, MRPL32, HECW1, HECW1-IT1, MIR3943, LOC100506895, STK17A, COA1, BLVRA, MRPS24, URGCP-MRPS24, URGCP, UBE2D4, POLR2J4, SPDYE1, RASA4CP, LINC00957, DBNL, MIR6837, PGAM2, POLM, MIR6838, AEBP1, MIR4649, POLD2, MYL7, GCK, YKT6, CAMK2B, NUDCD3, NPC1L1, DDX56, TMED4, OGDH, ZMIZ2, PPIA, H2AFV, PURB, MIR4657, MYO1G, SNHG15, SNORA9, CCM2, NACAD, TBRG4, SNORA5A, SNORA5C, SNORA5B, RAMP3, ADCY1, SEPT7P2, IGFBP1, IGFBP3, LOC730338, TNS3, LINC01447, C7orf65, LINC00525, PKD1L1, C7orf69, HUS1, SUN3, C7orf57, UPP1, ABCA13, CDC14C, VWC2, ZPBP, C7orf72, IKZF1, FIGNL1, DDC, DDC-AS1, GRB10, COBL, POM121L12, LINC01446, HPVC1, LINC01445, VSTM2A, VSTM2A-OT1, SEC61G, LOC100996654, EGFR, EGFR-AS1, ELDR, LANCL2, VOPP1, FKBP9P1, SEPT14, ZNF713, MRPS17, GBAS, PSPH, CCT6A, SNORA15, SUMF2, PHKG1, CHCHD2, NUPR2, LOC650226, LOC100240728, DKFZp434L192, LOC101928401, LOC401357, LOC100130849, MIR4283-1, MIR4283-2, ZNF479, GUSBP10, LOC105375297, MIR3147, ZNF716, ZNF733P, LOC102724738, LOC100287704, LOC100287834, LINC01005, ZNF727, ZNF735, ZNF679, ZNF736, YWHAEP1, ZNF680, LOC100128885, LOC641746, ZNF107, MIR6839, ZNF138, ZNF273, ZNF117, ERV3-1, CCT6P3, ZNF92, LOC441242, INTS4P2, CCT6P1, SNORA22, VKORC1L1, GUSB, ASL, CRCP, TPST1, LINC00174, GS1-124K5.4, GS1-124K5.11, KCTD7, LOC100996437, RABGEF1, GTF2IRD1P1, GTF2IP23, LOC644794, TMEM248, SBDS, TYW1, MIR4650-2, MIR4650-1, PMS2P4, STAG3L4, LINC01372, LOC102723427, LOC100507468, AUTS2, WBSCR17, MIR3914-1, MIR3914-2, CALN1, TYW1B, SBDSP1, SPDYE7P, POM121, NSUN5P2, TRIM74, LOC541473, LOC100101148, STAG3L1, STAG3L3, PMS2P7, PMS2P5, PMS2P2, SPDYE8P, GTF2IP4, GTF2IP1, NCF1B, NSUN5, TRIM50, FKBP6, FZD9, BAZ1B, BCL7B, TBL2, MLXIPL, VPS37D, DNAJC30, WBSCR22, STX1A, MIR4284, ABHD11-AS1, ABHD11, CLDN3, CLDN4, WBSCR27, WBSCR28, ELN, LIMK1, EIF4H, MIR590, LAT2, RFC2, CLIP2, GTF2IRD1, GTF2I, LOC101926943, NCF1, GTF2IRD2, STAG3L2, RCC1L, GTF2IRD2B, NCF1C, GATSL2, TRIM73, NSUN5P1, POM121C, SPDYE5, PMS2P3, HIP1, CCL26, CCL24, RHBDD2, POR, MIR4651, SNORA14A, TMEM120A, STYXL1, MDH2, GTF2IP7, SRRM3, HSPB1, YWHAG, SSC4D, ZP3, DTX2, FDPSP2, UPK3B, LOC100133091, POMZP3, DTX2P1-UPK3BP1-PMS2P11, PMS2P9, CCDC146, FGL2, GSAP, LOC101927243, PTPN12, APTR, RSBN1L, TMEM60, PHTF2, MAGI2, RPL13AP17, MAGI2-AS2, MAGI2-AS3, GNAI1, LOC101927269, GNAT3, CD36, SEMA3C, LOC105369146, LOC100128317, HGF, CACNA2D1, LOC101927356, PCLO, SEMA3E, SEMA3A, LOC101927378, SEMA3D, LINC00972, GRM3, KIAA1324L, LOC101927420, DMTF1, TMEM243, TP53TG1, CROT, ABCB4, ABCB1, RUNDC3B, SLC25A40, DBF4, ADAM22, SRI, LOC102723885, STEAP4, ZNF804B, C7orf62, STEAP2-AS1, DPY19L2P4, STEAP1, STEAP2, CFAP69, LOC101927446, GTPBP10, LOC101409256, CLDN12, CDK14, FZD1, MTERF1, AKAP9, CYP51A1, CYP51A1-AS1, LRRD1, KRIT1, ANKIB1, LOC105375396, GATAD1, PEX1, RBM48, FAM133B, FAM133DP, CDK6, LOC101927497, SAMD9, SAMD9L, HEPACAM2, VPS50, CALCR, MIR653, MIR489, MIR4652, TFPI2, LOC105375401, GNGT1, GNG11, BET1, COL1A2, CASD1, SGCE, PEG10, PPP1R9A, PON1, PON3, PON2, ASB4, PDK4, DYNC1I1, SLC25A13, MIR591, C7orf76, LOC100506136, SHFM1, DLX6-AS1, DLX6, DLX5, SDHAF3, TAC1, ASNS, MIR5692A1, MIR5692A2, MIR5692C2, MGC72080, OCM2, LMTK2, BHLHA15, TECPR1, BRI3, BAIAP2L1, NPTX2, TMEM130, TRRAP, MIR3609, SCARNA28, LOC101927550, SMURF1, KPNA7, MYH16, ARPC1A, ARPC1B, PDAP1, BUD31, PTCD1, ATP5J2-PTCD1, CPSF4, ATP5J2, ZNF789, ZNF394, ZKSCAN5, FAM200A, ZNF655, GS1-259H13.2, ZSCAN25, CYP3A5, CYP3A7-CYP3A51P, CYP3A7, CYP3A4, CYP3A43, OR2AE1, TRIM4, GJC3, AZGP1, AZGP1P1, ZKSCAN1, ZSCAN21, ZNF3, COPS6, MCM7, MIR25, MIR93, MIR106B, AP4M1, TAF6, CNPY4, MBLAC1, LAMTOR4, C7orf43, MIR4658, GAL3ST4, GPC2, STAG3, GATS, PVRIG, SPDYE3, PMS2P1, STAG3L5P-PVRIG2P-PILRB, STAG3L5P, PVRIG2P, MIR6840, PILRB, PILRA, ZCWPW1, MEPCE, PPP1R35, C7orf61, TSC22D4, NYAP1, AGFG2, SAP25, LRCH4, ZASP, FBXO24, PCOLCE-AS1, PCOLCE, MOSPD3, TFR2, ACTL6B, LOC105375429, GNB2, GIGYF1, POP7, EPO, ZAN, EPHB4, SLC12A9, TRIP6, MIR6875, SRRT, UFSP1, ACHE, MUC3A, MUC12, LOC102724094, MUC17, TRIM56, SERPINE1, AP1S1, MIR4653, VGF, NAT16, MOGAT3, PLOD3, ZNHIT1, CLDN15, FIS1, LOC101927746, IFT22, COL26A1, LINC01007, MYL10, CUX1, SH2B2, MIR4285, SPDYE6, LOC100289561, LOC100630923, PRKRIP1, MIR548O, ORAI2, ALKBH4, LRWD1, MIR5090, MIR4467, POLR2J, RASA4B, POLR2J3, SPDYE2B, SPDYE2, RASA4, POLR2J2, UPK3BL, FAM185A, FBXL13, LRRC17, ARMC10, NAPEPLD, RPL19P12, DPY19L2P2, PMPCB, DNAJC2, PSMC2, SLC26A5, LOC101927870, RELN, ORC5, LHFPL3, LHFPL3-AS1, LHFPL3-AS2, LINC01004, KMT2E-AS1, KMT2E, SRPK2, PUS7, RINT1, EFCAB10, ATXN7L1, CDHR3, SYPL1, NAMPT, CCDC71L, PIK3CG, PRKAR2B, HBP1, COG5, GPR22, DUS4L, BCAP29, SLC26A4-AS1, SLC26A4, CBLL1, SLC26A3, DLD, LAMB1, LAMB4, NRCAM, PNPLA8, THAP5, DNAJB9, C7orf66, EIF3IP1, IMMP2L, LRRN3, DOCK4, DOCK4-AS1, ZNF277, IFRD1, LSMEM1, LOC100996249, LOC101928012, TMEM168, BMT2, HRAT17, GPR85, LINC00998, PPP1R3A, FOXP2, MIR3666, MDFIC, LINC01393, LINC01392, TFEC, TES, LOC102724434, CAV2, CAV1, LINC01510, MET, CAPZA2, ST7-AS1, ST7, ST7-OT4, MIR6132, ST7-AS2, ST7-OT3, WNT2, ASZ1, CFTR, CTTNBP2, LSM8, ANKRD7, LVCAT5, KCND2, TSPAN12, ING3, CPED1, WNT16, FAM3C, PTPRZ1, AASS, FEZF1, FEZF1-AS1, CADPS2, RNF133, RNF148, TAS2R16, SLC13A1, IQUB, NDUFA5, ASB15, LOC102724555, LMOD2, WASL, RNU6-2, HYALP1, HYAL4, SPAM1, LOC105375483, TMEM229A, LOC101928211, GPR37, C7orf77, POT1, POT1-AS1, LOC101928283, LOC101928254, GRM8, MIR592, LOC101928333, ZNF800, LOC100506682, GCC1, ARF5, FSCN3, PAX4, SND1, SND1-IT1, LRRC4, MIR593, MIR129-1, LEP, MGC27345, RBM28, PRRT4, IMPDH1, HILPDA, METTL2B, LINC01000, FAM71F2, FAM71F1, CALU, OPN1SW, CCDC136, FLNC, ATP6V1F, LOC100130705, KCP, IRF5, TNPO3, TPI1P2, LOC407835, TSPAN33, SMO, AHCYL2, STRIP2, SMKR1, NRF1, MIR182, MIR96, MIR183, UBE2H, ZC3HC1, KLHDC10, TMEM209, SSMEM1, CPA2, CPA4, CPA5, LOC105375504, CPA1, CEP41, MEST, MESTIT1, MIR335, COPG2, TSGA13, KLF14, MIR29A, MIR29B1, LINC-PINT, LOC100506860, MKLN1, MKLN1-AS, PODXL, LOC101928782, PLXNA4, LOC101928807, FLJ40288, LOC100506937, CHCHD3, MIR3654, LOC105375512, EXOC4, MIR6133, LOC101928861, LRGUK, SLC35B4, AKR1B1, AKR1B10, AKR1B15, BPGM, CALD1, AGBL3, C7orf49, TMEM140, WDR91, MIR6509, STRA8, CNOT4, NUP205, C7orf73, SLC13A4, FAM180A, MTPN, LUZP6, CHRM2, LOC349160, MIR490, PTN, DGKI, CREB3L2, LOC100130880, AKR1D1, MIR4468, TRIM24, SVOPL, ATP6V0A4, TMEM213, KIAA1549, ZC3HAV1L, ZC3HAV1, TTC26, UBN2, LUC7L2, FMC1, C7orf55-LUC7L2, LOC100129148, KLRG2, CLEC2L, HIPK2, TBXAS1, PARP12, KDM7A, JHDM1D-AS1, SLC37A3, RAB19, MKRN1, DENND2A, ADCK2, NDUFB2-AS1, NDUFB2, BRAF, MRPS33, TMEM178B, AGK, KIAA1147, WEE2-AS1, WEE2, SSBP1, TAS2R3, TAS2R4, TAS2R5, PRSS37, OR9A4, CLEC5A, TAS2R38, MGAM, MGAM2, MOXD2P, PRSS58, TRY2P, MTRNR2L6, PRSS1, PRSS3P2, EPHB6, TRPV6, TRPV5, C7orf34, KEL, OR9A2, OR6V1, OR6W1P, PIP, TAS2R39, TAS2R40, LOC105375545, GSTK1, TMEM139, CASP2, CLCN1, FAM131B, LOC100507507, ZYX, MIR6892, EPHA1, EPHA1-AS1, TAS2R60, TAS2R41, CTAGE15, TCAF2, TCAF2P1, CTAGE6, LOC154761, TCAF1, OR2F2, OR2F1, OR6B1, OR2A5, OR2A25, OR2A12, OR2A2, OR2A14, CTAGE4, ARHGEF35, LOC101928605, OR2A1-AS1, OR2A1, OR2A42, OR2A9P, OR2A20P, OR2A7, ARHGEF34P, CTAGE8, ARHGEF5, NOBOX, TPK1, CNTNAP2, LOC101928700, MIR548F4, LOC105375556, MIR548T, C7orf33, CUL1, EZH2, GHET1, PDIA4, ZNF786, ZNF425, ZNF398, ZNF282, ZNF212, ZNF783, LOC155060, ZNF777, ZNF746, ZNF767P, KRBA1, ZNF467, SSPO, ZNF862, ATP6V0E2-AS1, ATP6V0E2, ACTR3C, LRRC61, ZBED6CL, RARRES2, REPIN1, ZNF775, LOC728743, LINC00996, GIMAP8, GIMAP7, GIMAP4, GIMAP6, GIMAP2, GIMAP1, GIMAP1-GIMAP5, GIMAP5, TMEM176B, TMEM176A, AOC1, KCNH2, NOS3, ATG9B, ABCB8, ASIC3, CDK5, SLC4A2, FASTK, TMUB1, AGAP3, GBX1, ASB10, IQCA1L, ABCF2, CHPF2, MIR671, SMARCD3, NUB1, WDR86, WDR86-AS1, CRYGN, MIR3907, RHEB, PRKAG2, PRKAG2-AS1, GALNTL5, GALNT11, KMT2C, FABP5P3, LINC01003, XRCC2, ACTR3B, LINC01287, DPP6, LOC101929998, PAXIP1-AS2, PAXIP1, PAXIP1-AS1, HTR5A-AS1, HTR5A, INSIG1, BLACE, EN2, CNPY1, LOC100506302, RBM33, SHH, LOC389602, LOC285889, LINC01006, LINC00244, C7orf13, RNF32, LMBR1, NOM1, MNX1, MNX1-AS1, UBE3C, DNAJB6, LOC101927914, PTPRN2, MIR153-2, LOC100506585, MIR595, LINC01022, MIR5707, NCAPG2, ESYT2, WDR60, LINC00689, VIPR2 | arr[GRCh37] 7p22.3q36.3(41420_159118443)x3 |
| 2  CRC | Gain | 8 | p11.22 | q24.3 | 106,653 | ADAM2, IDO1, IDO2, C8orf4, ZMAT4, SFRP1, MIR548AO, GOLGA7, GINS4, LOC102723729, GPAT4, NKX6-3, ANK1, MIR486-1, MIR486-2, KAT6A, LOC105379393, AP3M2, PLAT, LOC101929897, IKBKB, POLB, DKK4, VDAC3, SLC20A2, SMIM19, CHRNB3, CHRNA6, THAP1, RNF170, MIR4469, HOOK3, FNTA, POMK, HGSNAT, POTEA, LINC00293, LOC100287846, SPIDR, CEBPD, PRKDC, MCM4, UBE2V2, LOC101929268, LOC101929217, EFCAB1, SNAI2, C8orf22, LOC100507464, SNTG1, PXDNL, PCMTD1, ST18, LOC101929341, FAM150A, RB1CC1, NPBWR1, OPRK1, ATP6V1H, RGS20, TCEA1, LYPLA1, MRPL15, SOX17, RP1, XKR4, SBF1P1, LOC105375843, TMEM68, TGS1, LYN, RPS20, SNORD54, MOS, PLAG1, CHCHD7, SDR16C5, SDR16C6P, PENK, LOC101929415, LINC00968, IMPAD1, LINC01606, LOC286177, LINC00588, LOC101929488, LOC286178, LINC01602, FAM110B, LOC101929528, UBXN2B, CYP7A1, SDCBP, NSMAF, TOX, CA8, LINC01301, RAB2A, CHD7, LOC100130298, CLVS1, ASPH, MIR4470, NKAIN3, UG0898H09, GGH, TTPA, YTHDF3-AS1, YTHDF3, LOC102724612, LINC01289, LOC102724623, MIR124-2HG, MIR124-2, LOC401463, BHLHE22, CYP7B1, LINC00251, LINC01299, ARMC1, MTFR1, PDE7A, DNAJC5B, TRIM55, CRH, LINC00967, RRS1-AS1, RRS1, ADHFE1, C8orf46, MYBL1, VCPIP1, C8orf44, C8orf44-SGK3, SGK3, PTTG3P, MCMDC2, SNHG6, SNORD87, TCF24, PPP1R42, COPS5, CSPP1, ARFGEF1, LOC102724708, CPA6, PREX2, C8orf34-AS1, C8orf34, LINC01592, LINC01603, SULF1, SLCO5A1, PRDM14, NCOA2, LOC101926892, TRAM1, LACTB2-AS1, LACTB2, XKR9, EYA1, MSC, MSC-AS1, TRPA1, LOC392232, KCNB2, LOC101926908, TERF1, SBSPON, C8orf89, RPL7, RDH10, RDH10-AS1, STAU2-AS1, STAU2, UBE2W, TCEB1, TMEM70, LY96, JPH1, GDAP1, MIR5681A, MIR5681B, MIR2052HG, MIR2052, PI15, CRISPLD1, CASC9, HNF4G, LINC01111, ZFHX4-AS1, ZFHX4, MIR3149, PEX2, LOC102724874, PKIA, PKIA-AS1, ZC2HC1A, LOC101241902, IL7, STMN2, HEY1, LINC01607, LOC101927040, MRPS28, TPD52, MIR5708, ZBTB10, ZNF704, PAG1, FABP5, PMP2, FABP9, FABP4, FABP12, IMPA1, SLC10A5, ZFAND1, CHMP4C, SNX16, LOC101927141, LINC01419, RALYL, LRRCC1, LOC102723322, E2F5, C8orf59, CA13, CA1, CA3, CA3-AS1, CA2, REXO1L2P, PSKH2, ATP6V0D2, SLC7A13, WWP1, RMDN1, CPNE3, CNGB3, CNBD1, DCAF4L2, MMP16, LOC101929709, RIPK2, OSGIN2, NBN, DECR1, CALB1, LINC00534, LINC01030, TMEM64, NECAB1, C8orf88, TMEM55A, OTUD6B-AS1, OTUD6B, LRRC69, MIR4661, SLC26A7, RUNX1T1, MIR7641-2, LOC102724710, FLJ46284, TRIQK, MIR8084, C8orf87, LINC00535, FAM92A1, RBM12B, RBM12B-AS1, TMEM67, MIR378D2, PDP1, CDH17, GEM, RAD54B, FSBP, KIAA1429, LOC100288748, ESRP1, DPY19L4, INTS8, CCNE2, TP53INP1, NDUFAF6, LOC105375650, MIR3150B, MIR3150A, PLEKHF2, LINC01298, C8orf37, C8orf37-AS1, LOC100500773, GDF6, UQCRB, MTERF3, PTDSS1, LOC102724804, SDC2, CPQ, LOC101927066, TSPYL5, MTDH, LAPTM4B, MATN2, RPL30, SNORA72, ERICH5, RIDA, POP1, NIPAL2, KCNS2, STK3, OSR2, VPS13B, MIR599, MIR875, COX6C, RGS22, MIR1273A, FBXO43, POLR2K, SPAG1, RNF19A, MIR4471, ANKRD46, SNX31, PABPC1, MIR7705, YWHAZ, FLJ42969, ZNF706, NACAP1, GRHL2, NCALD, LOC104054148, MIR5680, RRM2B, UBR5-AS1, UBR5, ODF1, KLF10, AZIN1, AZIN1-AS1, ATP6V1C1, BAALC-AS2, BAALC, MIR3151, BAALC-AS1, FZD6, CTHRC1, SLC25A32, DCAF13, RIMS2, DCSTAMP, DPYS, LRP12, ZFPM2, ZFPM2-AS1, OXR1, ABRA, ANGPT1, RSPO2, EIF3E, EMC2, TMEM74, TRHR, NUDCD1, ENY2, PKHD1L1, EBAG9, SYBU, LOC100132813, KCNV1, LINC01608, LINC01609, CSMD3, MIR2053, TRPS1, LINC00536, EIF3H, LOC105375713, UTP23, RAD21, RAD21-AS1, MIR3610, AARD, SLC30A8, MED30, EXT1, SAMD12, SAMD12-AS1, TNFRSF11B, COLEC10, LOC101927513, MAL2, MIR548AZ, NOV, ENPP2, TAF2, DSCC1, DEPTOR, COL14A1, MRPL13, MTBP, SNTB1, LOC101927543, HAS2, HAS2-AS1, LOC105375734, LINC01151, ZHX2, DERL1, TBC1D31, FAM83A, FAM83A-AS1, MIR4663, C8orf76, ZHX1-C8orf76, ZHX1, ATAD2, MIR548D1, WDYHV1, FBXO32, KLHL38, ANXA13, FAM91A1, FER1L6, FER1L6-AS1, FER1L6-AS2, LOC101927588, TMEM65, TRMT12, RNF139-AS1, RNF139, TATDN1, MIR6844, NDUFB9, MTSS1, MIR4662B, MIR4662A, LINC00964, ZNF572, LOC105375744, SQLE, KIAA0196, NSMCE2, TRIB1, LINC00861, LOC101927657, FAM84B, PCAT1, PCAT2, PRNCR1, CASC19, CCAT1, CASC21, CASC8, CCAT2, POU5F1B, CASC11, MYC, PVT1, MIR1204, TMEM75, MIR1205, MIR1206, MIR1207, MIR1208, LINC00824, LINC00977, CCDC26, MIR3686, GSDMC, FAM49B, MIR5194, ASAP1, ASAP1-IT2, ASAP1-IT1, ADCY8, EFR3A, OC90, HHLA1, KCNQ3, HPYR1, LRRC6, TMEM71, PHF20L1, TG, SLA, MIR7848, WISP1, NDRG1, ST3GAL1, LOC105375773, LOC101927798, LOC101927822, ZFAT, ZFAT-AS1, MIR30B, MIR30D, NCRNA00250, LOC101927845, LINC01591, KHDRBS3, LOC101927915, FAM135B, COL22A1, KCNK9, TRAPPC9, CHRAC1, AGO2, PTK2, DENND3, SLC45A4, LOC105375787, LINC01300, GPR20, PTP4A3, MROH5, MIR1302-7, MIR4539, MIR4472-1, LINC00051, TSNARE1, ADGRB1, ARC, LOC101928087, JRK, PSCA, LY6K, LOC100288181, THEM6, SLURP1, LYPD2, LYNX1, LY6D, GML, CYP11B1, CYP11B2, LOC100133669, CDC42P3, LY6E, C8orf31, LY6H, GPIHBP1, ZFP41, GLI4, MINCR, ZNF696, TOP1MT, RHPN1-AS1, RHPN1, MAFA-AS1, MAFA, ZC3H3, GSDMD, MROH6, NAPRT, EEF1D, TIGD5, PYCRL, TSTA3, ZNF623, ZNF707, BREA2, CCDC166, LOC101928160, MAPK15, FAM83H, MIR4664, FAM83H-AS1, LOC105375800, SCRIB, MIR937, PUF60, NRBP2, MIR6845, EPPK1, PLEC, MIR661, PARP10, GRINA, SPATC1, OPLAH, MIR6846, EXOSC4, MIR6847, GPAA1, CYC1, SHARPIN, MAF1, WDR97, HGH1, MROH1, MIR7112, SCX, BOP1, HSF1, DGAT1, MIR6848, SCRT1, TMEM249, FBXL6, SLC52A2, LOC101928902, ADCK5, CPSF1, MIR939, MIR6849, SLC39A4, VPS28, TONSL, TONSL-AS1, MIR6893, CYHR1, KIFC2, FOXH1, PPP1R16A, GPT, MFSD3, RECQL4, LRRC14, LRRC24, C8orf82, ARHGAP39, ZNF251, ZNF34, RPL8, MIR6850, ZNF517, ZNF7, COMMD5, ZNF250, ZNF16, ZNF252P, TMED10P1, ZNF252P-AS1, C8orf33 | arr[GRCh37] 8p11.22q24.3(39639410_146292734)x3 |
| 2  CRC | Gain | 12 | q11 | q24.33 | 95,915 | ALG10B, CPNE8, KIF21A, ABCD2, C12orf40, SLC2A13, LRRK2, MUC19, CNTN1, PDZRN4, LOC101927038, GXYLT1, YAF2, ZCRB1, MIR7851, PPHLN1, PRICKLE1, LOC101927058, LOC105369738, LOC105369739, ADAMTS20, PUS7L, IRAK4, TWF1, TMEM117, NELL2, DBX2, RACGAP1P, PLEKHA8P1, RNY5, ANO6, LINC00938, ARID2, SCAF11, SLC38A1, SLC38A2, LOC100288798, SLC38A4, AMIGO2, PCED1B, MIR4698, PCED1B-AS1, LOC105369747, MIR4494, RPAP3, ENDOU, RAPGEF3, SLC48A1, HDAC7, VDR, TMEM106C, COL2A1, SENP1, PFKM, MIR6505, ASB8, CCDC184, OR10AD1, H1FNT, ZNF641, ANP32D, C12orf54, OR8S1, LALBA, KANSL2, SNORA2C, MIR1291, SNORA2A, SNORA2B, CCNT1, LINC00935, ADCY6, MIR4701, LOC100506125, CACNB3, DDX23, RND1, CCDC65, FKBP11, ARF3, WNT10B, WNT1, DDN, PRKAG1, KMT2D, RHEBL1, DHH, LMBR1L, TUBA1B, TUBA1A, TUBA1C, LOC101927267, PRPH, TROAP, C1QL4, DNAJC22, SPATS2, LOC100335030, KCNH3, MCRS1, FAM186B, PRPF40B, FMNL3, TMBIM6, NCKAP5L, BCDIN3D-AS1, BCDIN3D, FAIM2, LOC283332, LOC101927292, AQP2, LOC101927318, AQP5, AQP6, RACGAP1, ASIC1, SMARCD1, GPD1, COX14, CERS5, LIMA1, MIR1293, FAM186A, LARP4, SNORD133, DIP2B, ATF1, TMPRSS12, METTL7A, HIGD1C, SLC11A2, LETMD1, CSRNP2, TFCP2, POU6F1, DAZAP2, SMAGP, BIN2, CELA1, GALNT6, SLC4A8, SCN8A, FIGNL2, LOC105369971, ANKRD33, ACVRL1, ACVR1B, GRASP, NR4A1, ATG101, OR7E47P, KRT80, C12orf80, LINC00592, KRT7, KRT86, KRT81, KRT83, KRT85, KRT84, KRT82, KRT75, KRT6B, KRT6C, KRT6A, KRT5, KRT71, KRT74, KRT72, KRT73, KRT73-AS1, KRT2, KRT1, KRT77, KRT76, KRT3, KRT4, KRT79, KRT78, KRT8, KRT18, EIF4B, LOC283335, TNS2, MIR6757, SPRYD3, IGFBP6, SOAT2, CSAD, ZNF740, ITGB7, RARG, MFSD5, ESPL1, PFDN5, C12orf10, AAAS, SP7, SP1, AMHR2, PRR13, PCBP2, PCBP2-OT1, MAP3K12, TARBP2, NPFF, ATF7, LOC100652999, ATP5G2, CALCOCO1, CISTR, HOXC13-AS, HOXC13, HOXC12, HOTAIR, HOXC11, HOXC-AS3, HOXC10, MIR196A2, HOXC-AS2, HOXC-AS1, HOXC9, HOXC8, HOXC6, HOXC5, HOXC4, MIR615, FLJ12825, LOC100240735, LOC100240734, LOC400043, SMUG1, CBX5, MIR3198-2, HNRNPA1, HNRNPA1P10, NFE2, COPZ1, MIR148B, LOC102724050, GPR84, ZNF385A, ITGA5, GTSF1, NCKAP1L, PDE1B, PPP1R1A, GLYCAM1, LACRT, DCD, MUCL1, TESPA1, NEUROD4, OR9K2, OR10A7, OR6C74, OR6C6, OR6C1, OR6C3, OR6C75, OR6C65, OR6C76, OR6C2, OR6C70, OR6C68, OR6C4, OR2AP1, OR10P1, METTL7B, ITGA7, BLOC1S1, BLOC1S1-RDH5, RDH5, CD63, GDF11, SARNP, ORMDL2, DNAJC14, TMEM198B, MMP19, PYM1, DGKA, PMEL, CDK2, RAB5B, SUOX, LOC105369781, IKZF4, RPS26, ERBB3, PA2G4, RPL41, ZC3H10, ESYT1, MYL6B, MYL6, SMARCC2, RNF41, NABP2, SLC39A5, ANKRD52, COQ10A, CS, CNPY2, PAN2, IL23A, STAT2, APOF, TIMELESS, MIP, SPRYD4, GLS2, SNORA105C, RBMS2, BAZ2A, ATP5B, SNORD59B, SNORD59A, PTGES3, NACA, PRIM1, HSD17B6, SDR9C7, RDH16, GPR182, ZBTB39, TAC3, MYO1A, NEMP1, NAB2, STAT6, LRP1, LRP1-AS, MIR1228, NXPH4, SHMT2, NDUFA4L2, STAC3, R3HDM2, INHBC, INHBE, GLI1, ARHGAP9, MARS, MIR6758, DDIT3, MIR616, MBD6, DCTN2, KIF5A, PIP4K2C, DTX3, ARHGEF25, LOC101927583, SLC26A10, B4GALNT1, OS9, AGAP2, AGAP2-AS1, TSPAN31, CDK4, MIR6759, MARCH9, CYP27B1, METTL1, METTL21B, TSFM, AVIL, CTDSP2, MIR26A2, LOC100506844, ATP23, LOC105369785, LOC101927653, LOC100506869, LRIG3, SLC16A7, FAM19A2, USP15, MIR6125, MON2, LINC01465, MIRLET7I, PPM1H, AVPR1A, DPY19L2, TMEM5, TMEM5-AS1, SRGAP1, C12orf66, C12orf56, XPOT, TBK1, RASSF3, MIR548Z, MIR548C, GNS, TBC1D30, FLJ41278, WIF1, LEMD3, MSRB3, LOC100507065, LOC105369187, RPSAP52, HMGA2, LOC100129940, MIR6074, LLPH, LLPH-AS1, TMBIM4, IRAK3, MIR6502, HELB, GRIP1, LOC102724421, CAND1, LOC100507175, DYRK2, LOC101927901, LINC01479, IFNG-AS1, IFNG, IL26, IL22, MDM1, LOC100507195, RAP1B, SNORA70G, LOC100507250, NUP107, SLC35E3, LOC100130075, MDM2, CPM, CPSF6, MIR1279, LYZ, YEATS4, FRS2, MIR3913-1, MIR3913-2, CCT2, LRRC10, BEST3, LOC101928002, RAB3IP, MYRFL, LINC01481, CNOT2, KCNMB4, PTPRB, PTPRR, TSPAN8, LGR5, ZFC3H1, THAP2, TMEM19, RAB21, TBC1D15, MRS2P2, TPH2, TRHDE-AS1, TRHDE, LOC101928137, LOC100507377, ATXN7L3B, KCNC2, CAPS2, GLIPR1L1, GLIPR1L2, GLIPR1, KRR1, PHLDA1, NAP1L1, BBS10, OSBPL8, ZDHHC17, CSRP2, E2F7, NAV3, LOC105369860, SYT1, MIR1252, PAWR, PPP1R12A, OTOGL, PTPRQ, MYF6, MYF5, LINC01490, LIN7A, MIR617, MIR618, ACSS3, MIR4699, PPFIA2, LOC102724663, LOC101928449, CCDC59, METTL25, TMTC2, SLC6A15, TSPAN19, LRRIQ1, ALX1, RASSF9, NTS, MGAT4C, LOC105369879, MKRN9P, C12orf50, C12orf29, CEP290, TMTC3, KITLG, LOC728084, DUSP6, POC1B, GALNT4, POC1B-GALNT4, ATP2B1, LINC00936, LOC105369891, LOC105369893, LINC00615, CCER1, EPYC, KERA, LUM, DCN, LINC01619, BTG1, LOC101928617, CLLU1OS, CLLU1, C12orf74, PLEKHG7, EEA1, LOC643339, LOC102724933, NUDT4, NUDT4P2, NUDT4P1, UBE2N, MRPL42, SOCS2-AS1, SOCS2, CRADD, LOC101928731, LOC105369911, PLXNC1, CEP83, CEP83-AS1, MIR5700, TMCC3, MIR7844, MIR492, KRT19P2, NDUFA12, NR2C1, FGD6, VEZT, MIR331, MIR3685, METAP2, USP44, PGAM1P5, NTN4, LOC105369921, LOC105369920, SNRPF, CCDC38, AMDHD1, HAL, LTA4H, ELK3, CDK17, CFAP54, NEDD1, RMST, MIR1251, MIR135A2, LOC643711, MIR4495, MIR4303, SLC9A7P1, LOC643770, TMPO-AS1, TMPO, SLC25A3, SNORA53, IKBIP, APAF1, ANKS1B, LOC101928937, FAM71C, UHRF1BP1L, GOLGA2P5, MIR1827, ACTR6, DEPDC4, SCYL2, SLC17A8, NR1H4, GAS2L3, ANO4, SLC5A8, UTP20, ARL1, SPIC, MYBPC1, CHPT1, SYCP3, GNPTAB, DRAM1, CCDC53, NUP37, PARPBP, PMCH, IGF1, LINC00485, PAH, ASCL1, LOC101929058, C12orf42, LOC105369945, LOC101929084, STAB2, NT5DC3, TTC41P, HSP90B1, MIR3652, C12orf73, TDG, GLT8D2, HCFC2, NFYB, MIR7641-2, TXNRD1, EID3, CHST11, MIR3922, SLC41A2, C12orf45, ALDH1L2, LOC414300, KIAA1033, APPL2, KCCAT198, C12orf75, CASC18, NUAK1, CKAP4, TCP11L2, POLR3B, LOC100287944, RFX4, LOC100505978, RIC8B, TMEM263, MTERF2, CRY1, BTBD11, PWP1, PRDM4, LOC101929162, ASCL4, LOC728739, WSCD2, CMKLR1, LINC01498, FICD, SART3, ISCU, TMEM119, SELPLG, MIR4496, CORO1C, SSH1, MIR619, DAO, SVOP, USP30, USP30-AS1, ALKBH2, UNG, ACACB, FOXN4, LINC01486, MYO1H, KCTD10, UBE3B, MMAB, MVK, FAM222A, FAM222A-AS1, TRPV4, MIR4497, GLTP, TCHP, GIT2, ANKRD13A, C12orf76, IFT81, ATP2A2, ANAPC7, ARPC3, GPN3, FAM216A, VPS29, RAD9B, PPTC7, TCTN1, HVCN1, PPP1CC, CCDC63, MYL2, LINC01405, LOC105369980, CUX2, MIR6760, FAM109A, SH2B3, ATXN2, BRAP, ACAD10, ALDH2, MIR6761, MAPKAPK5-AS1, MAPKAPK5, ADAM1A, TMEM116, ERP29, NAA25, MIR3657, TRAFD1, HECTD4, MIR6861, RPL6, PTPN11, MIR1302-1, RPH3A, OAS1, OAS3, OAS2, DTX1, RASAL1, CFAP73, DDX54, MIR7106, RITA1, IQCD, TPCN1, MIR6762, SLC8B1, PLBD2, SDS, SDSL, LHX5, LHX5-AS1, LINC01234, RBM19, TBX5, TBX5-AS1, TBX3, MED13L, MIR620, MIR4472-2, LINC00173, MAP1LC3B2, C12orf49, RNFT2, HRK, FBXW8, LOC100506551, TESC, TESC-AS1, FBXO21, NOS1, KSR2, RFC5, WSB2, VSIG10, PEBP1, TAOK3, SUDS3, LOC105370014, LOC105370016, SRRM4, LOC105370024, HSPB8, LINC00934, CCDC60, TMEM233, PRKAB1, CIT, MIR1178, BICDL1, RAB35, GCN1, MIR4498, RPLP0, PXN-AS1, PXN, SIRT4, PLA2G1B, MSI1, COX6A1, TRIAP1, GATC, SRSF9, DYNLL1, NRAV, COQ5, RNF10, POP5, CABP1, MLEC, UNC119B, MIR4700, ACADS, SPPL3, XLOC_009911, HNF1A-AS1, HNF1A, C12orf43, OASL, P2RX7, P2RX4, CAMKK2, ANAPC5, RNF34, KDM2B, MIR7107, MIR548AQ, ORAI1, MORN3, TMEM120B, RHOF, LINC01089, SETD1B, HPD, PSMD9, WDR66, BCL7A, LOC100506691, MLXIP, LRRC43, IL31, B3GNT4, DIABLO, LOC101593348, VPS33A, CLIP1, CLIP1-AS1, ZCCHC8, RSRC2, KNTC1, HCAR2, HCAR3, HCAR1, DENR, CCDC62, HIP1R, VPS37B, ABCB9, OGFOD2, ARL6IP4, PITPNM2, MIR4304, LOC100507091, MPHOSPH9, C12orf65, CDK2AP1, SBNO1, MIR8072, KMT5A, RILPL2, SNRNP35, RILPL1, MIR3908, LOC101927415, TMED2, DDX55, EIF2B1, GTF2H3, TCTN2, ATP6V0A2, DNAH10, CCDC92, ZNF664, ZNF664-FAM101A, FAM101A, NCOR2, MIR6880, SCARB1, UBC, MIR5188, DHX37, BRI3BP, THRIL, AACS, TMEM132B, LINC00939, LOC101927464, LOC100128554, LOC100996671, LINC00944, LINC00943, LOC440117, LOC101927592, LOC101927616, LOC101927637, LOC105370068, FLJ37505, LINC00508, LINC00507, CRAT8, LOC100996679, LOC101927694, MIR4419B, TMEM132C, MIR3612, SLC15A4, GLT1D1, TMEM132D, LOC283352, LOC101927735, LOC100190940, FZD10-AS1, FZD10, PIWIL1, RIMBP2, STX2, RAN, ADGRD1, LACAT8, LINC01257, LOC107161159, LOC338797, SFSWAP, MMP17, ULK1, PUS1, EP400, SNORA49, EP400NL, DDX51, NOC4L, GALNT9, LOC100130238, LOC101928416, FBRSL1, MIR6763, LRCOL1, P2RX2, POLE, PXMP2, PGAM5, ANKLE2, GOLGA3, CHFR, LOC101928530, ZNF605, ZNF26, LOC101928597, ZNF84, ZNF140, ZNF891, ZNF10, ZNF268, ANHX | arr[GRCh37] 12q11q24.33(37902987_133818115)x3 |
| 2  CRC | Gain | 13 | q12.13 | q34 | 89,422 | AMER2, LINC00463, LINC01053, MTMR6, NUP58, ATP8A2, SHISA2, RNF6, CDK8, WASF3, GPR12, USP12, USP12-AS1, USP12-AS2, LINC00412, RPL21, RPL21P28, SNORD102, SNORA27, RASL11A, GTF3A, MTIF3, LNX2, POLR1D, GSX1, PDX1-AS1, PDX1, ATP5EP2, LINC00543, CDX2, URAD, FLT3, PAN3-AS1, PAN3, FLT1, POMP, SLC46A3, MTUS2, MTUS2-AS1, SLC7A1, LOC102723345, UBL3, LINC00297, LINC00572, LINC00544, LINC00365, KATNAL1, LINC00426, LINC01058, HMGB1, USPL1, ALOX5AP, LINC00398, LINC00545, TEX26-AS1, MEDAG, TEX26, HSPH1, B3GLCT, RXFP2, EEF1DP3, FRY-AS1, FRY, ZAR1L, BRCA2, N4BP2L1, N4BP2L2, MINOS1P1, N4BP2L2-IT2, PDS5B, LINC00423, KL, STARD13, STARD13-AS, RFC3, LINC00457, NBEA, MAB21L1, LINC00445, DCLK1, SOHLH2, CCDC169-SOHLH2, CCDC169, SPG20, SPG20-AS1, CCNA1, SERTM1, RFXAP, SMAD9, ALG5, EXOSC8, SUPT20H, CSNK1A1L, LINC01048, LINC00547, POSTN, TRPC4, LINC00571, UFM1, LINC00437, LINC00366, FREM2, STOML3, PROSER1, NHLRC3, LHFP, COG6, MIR4305, LINC00332, LINC00548, LINC00598, FOXO1, MIR320D1, MRPS31, SLC25A15, TPTE2P5, MIR621, SUGT1P3, ELF1, WBP4, MIR3168, KBTBD6, LOC101929140, KBTBD7, MTRF1, NAA16, OR7E37P, RGCC, VWA8, MIR5006, VWA8-AS1, DGKH, AKAP11, LOC105370177, TNFSF11, FAM216B, LINC01050, LINC00428, EPSTI1, DNAJC15, LINC00400, ENOX1, ENOX1-AS2, CCDC122, LACC1, LINC00284, LINC00390, SMIM2-AS1, SMIM2, SMIM2-IT1, MIR8079, SERP2, TUSC8, TSC22D1, TSC22D1-AS1, LINC00330, NUFIP1, GPALPP1, LOC101929259, GTF2F2, KCTD4, TPT1, SNORA31, TPT1-AS1, SLC25A30, SLC25A30-AS1, COG3, ERICH6B, LINC01055, SPERT, SIAH3, ZC3H13, CPB2-AS1, CPB2, LCP1, LRRC63, LINC00563, KIAA0226L, LINC01198, LRCH1, ESD, HTR2A, HTR2A-AS1, LINC00562, SUCLA2, NUDT15, MED4, MED4-AS1, ITM2B, LINC00441, RB1, LPAR6, RCBTB2, LINC00462, CYSLTR2, FNDC3A, MLNR, LOC105370203, CDADC1, CAB39L, SETDB2, SETDB2-PHF11, PHF11, RCBTB1, ARL11, EBPL, KPNA3, CTAGE10P, SPRYD7, DLEU2, MIR3613, TRIM13, KCNRG, MIR16-1, MIR15A, DLEU1, ST13P4, DLEU1-AS1, DLEU7, DLEU7-AS1, RNASEH2B-AS1, RNASEH2B, GUCY1B2, LINC00371, FAM124A, SERPINE3, MIR5693, INTS6, INTS6-AS1, MIR4703, WDFY2, DHRS12, LINC00282, CCDC70, ATP7B, ALG11, UTP14C, NEK5, LOC101929657, NEK3, MRPS31P5, LOC103191607, THSD1, VPS36, CKAP2, TPTE2P3, HNRNPA1L2, SUGT1, LECT1, MIR759, PCDH8, OLFM4, LINC01065, LINC00558, LINC00458, MIR1297, MIR5007, PRR20A, PRR20D, PRR20E, PRR20B, PRR20C, PCDH17, LOC101926897, LINC00374, DIAPH3, DIAPH3-AS1, DIAPH3-AS2, LINC00434, TDRD3, LINC00378, MIR3169, PCDH20, LOC101926951, LINC00358, LINC01075, LINC00448, LINC00376, LINC00395, OR7E156P, LOC102723968, LINC01052, MIR548X2, MIR4704, PCDH9, PCDH9-AS2, PCDH9-AS3, PCDH9-AS4, LINC00364, LINC00550, LINC00383, KLHL1, ATXN8OS, LINC00348, DACH1, MZT1, BORA, DIS3, PIBF1, KLF5, LINC00392, KLF12, LINC00381, LINC00347, CTAGE11P, LINC01078, TBC1D4, COMMD6, UCHL3, LMO7-AS1, LMO7, LMO7DN, LMO7DN-IT1, KCTD12, BTF3P11, ACOD1, CLN5, FBXL3, MYCBP2, MYCBP2-AS1, SCEL, SCEL-AS1, LOC100129307, SLAIN1, MIR3665, EDNRB-AS1, EDNRB, LINC01069, LINC00446, RNF219-AS1, POU4F1, RNF219, LINC00331, RBM26, RBM26-AS1, NDFIP2-AS1, NDFIP2, LINC01068, LINC01038, LINC00382, LINC01080, SPRY2, LINC00377, LINC00564, SLITRK1, LINC00333, SNORA107, LINC00375, LINC00351, SLITRK6, LINC00430, MIR4500HG, MIR4500, SLITRK5, LINC00397, LOC105370306, LINC00433, LINC01047, LINC00440, LINC01040, LINC00353, LINC00559, MIR622, LINC01049, LINC00410, LINC00380, LINC00379, MIR17HG, MIR17, MIR18A, MIR19A, MIR20A, MIR19B1, MIR92A1, GPC5, GPC5-AS2, MIR548AS, GPC5-AS1, LINC00363, GPC6, GPC6-AS2, GPC6-AS1, DCT, TGDS, GPR180, LOC101927248, SOX21, SOX21-AS1, LOC101927284, LINC00557, ABCC4, CLDN10, CLDN10-AS1, DZIP1, DNAJC3-AS1, DNAJC3, UGGT2, HS6ST3, MIR4501, LINC00359, OXGR1, LINC00456, MBNL2, RAP2A, IPO5, FARP1, RNF113B, MIR3170, STK24, SLC15A1, DOCK9, DOCK9-AS1, DOCK9-AS2, UBAC2-AS1, UBAC2, GPR18, GPR183, FKSG29, MIR623, LINC01232, LINC00449, TM9SF2, LINC01039, CLYBL, MIR4306, CLYBL-AS2, CLYBL-AS1, LOC101927437, ZIC5, ZIC2, LINC00554, LOC105370333, PCCA, PCCA-AS1, GGACT, TMTC4, NALCN-AS1, LINC00411, NALCN, ITGBL1, FGF14, MIR2681, MIR4705, FGF14-IT1, FGF14-AS1, FGF14-AS2, TPP2, METTL21C, CCDC168, TEX30, KDELC1, BIVM, BIVM-ERCC5, ERCC5, METTL21EP, SLC10A2, LINC01309, DAOA-AS1, DAOA, LINC00343, LINC00460, EFNB2, ARGLU1, LINC00551, LINC00443, FAM155A, MIR1267, FAM155A-IT1, LIG4, ABHD13, TNFSF13B, MYO16, MYO16-AS1, LINC00399, LINC00676, IRS2, LINC00396, COL4A1, COL4A2, MIR8073, COL4A2-AS2, COL4A2-AS1, RAB20, NAXD, CARS2, ING1, LINC00567, LINC00346, ANKRD10, LINC00431, LINC00368, ARHGEF7-AS2, ARHGEF7, ARHGEF7-AS1, LOC101060553, TEX29, LOC105370369, LINC00354, LINC00403, SOX1, LOC100506016, LINC01070, LOC101928730, LINC01043, LINC01044, SPACA7, TUBGCP3, ATP11AUN, ATP11A, ATP11A-AS1, MCF2L-AS1, MCF2L, F7, F10, F10-AS1, PROZ, PCID2, CUL4A, MIR8075, LAMP1, GRTP1, GRTP1-AS1, LOC101928841, ADPRHL1, DCUN1D2, TMCO3, TFDP1, ATP4B, GRK1, LINC00552, TMEM255B, GAS6-AS1, GAS6, GAS6-AS2, LINC00452, LINC00565, RASA3, CDC16, MIR548AR, MIR4502, UPF3A, CHAMP1, LINC01054 | arr[GRCh37] 13q12.13q34(25680689_115103150)x3 |
| 2  CRC | Gain | 16 | p13.3 | q24.1 | 85,230 | POLR3K, SNRNP25, RHBDF1, MPG, NPRL3, HBZ, HBM, HBA2, HBA1, HBQ1, LUC7L, FAM234A, RGS11, ARHGDIG, PDIA2, AXIN1, MRPL28, TMEM8A, LOC100134368, NME4, DECR2, RAB11FIP3, LINC00235, CAPN15, MIR5587, MIR3176, PRR35, NHLRC4, PIGQ, RAB40C, WFIKKN1, METTL26, MCRIP2, LOC105371038, WDR90, RHOT2, RHBDL1, LOC105371184, STUB1, JMJD8, WDR24, FBXL16, METRN, FAM173A, CCDC78, HAGHL, NARFL, MSLN, MIR662, RPUSD1, CHTF18, GNG13, PRR25, LMF1, LMF1-AS1, SOX8, SSTR5-AS1, SSTR5, C1QTNF8, CACNA1H, TPSG1, TPSB2, TPSAB1, TPSD1, UBE2I, BAIAP3, TSR3, GNPTG, UNKL, C16orf91, CCDC154, CLCN7, PTX4, TELO2, IFT140, TMEM204, LOC105371046, CRAMP1, HN1L, MAPK8IP3, MIR3177, NME3, MRPS34, EME2, SPSB3, NUBP2, IGFALS, HAGH, FAHD1, MEIOB, LINC00254, HS3ST6, MSRB1, RPL3L, NDUFB10, RPS2, SNORA10, SNORA64, SNHG9, SNORA78, RNF151, TBL3, NOXO1, GFER, SYNGR3, ZNF598, NPW, SLC9A3R2, NTHL1, TSC2, PKD1, MIR1225, LOC105371049, MIR6511B1, MIR6511B2, MIR4516, MIR3180-5, RAB26, SNHG19, SNORD60, TRAF7, CASKIN1, MLST8, BRICD5, PGP, E4F1, DNASE1L2, ECI1, RNPS1, LOC106660606, MIR3677, MIR940, MIR4717, ABCA3, ABCA17P, CCNF, MIR6767, C16orf59, MIR6768, LOC729652, NTN3, TBC1D24, ATP6V0C, AMDHD2, CEMP1, MIR3178, PDPK1, LOC652276, FLJ42627, ERVK13-1, KCTD5, PRSS27, SRRM2-AS1, SRRM2, TCEB2, PRSS33, PRSS41, PRSS21, ZG16B, PRSS30P, PRSS22, FLYWCH2, FLYWCH1, KREMEN2, PAQR4, PKMYT1, LINC00514, LOC101929613, CLDN9, CLDN6, TNFRSF12A, HCFC1R1, THOC6, BICDL2, LOC100128770, MMP25, MMP25-AS1, IL32, ZSCAN10, ZNF205-AS1, ZNF205, ZNF213-AS1, ZNF213, CASP16P, OR1F1, OR1F2P, ZNF200, MEFV, LINC00921, ZNF263, TIGD7, ZNF75A, OR2C1, MTRNR2L4, ZSCAN32, ZNF174, ZNF597, NAA60, MIR6126, C16orf90, CLUAP1, NLRC3, SLX4, DNASE1, TRAP1, CREBBP, LOC102724927, ADCY9, SRL, LINC01569, TFAP4, GLIS2, GLIS2-AS1, PAM16, CORO7-PAM16, CORO7, VASN, DNAJA3, NMRAL1, HMOX2, CDIP1, C16orf96, UBALD1, MGRN1, MIR6769A, NUDT16L1, ANKS3, C16orf71, ZNF500, SEPT12, SMIM22, ROGDI, GLYR1, UBN1, PPL, SEC14L5, NAGPA, NAGPA-AS1, C16orf89, ALG1, EEF2KMT, LINC01570, MIR8065, RBFOX1, TMEM114, METTL22, ABAT, TMEM186, PMM2, CARHSP1, USP7, C16orf72, LOC101927009, LINC01177, LINC01195, GRIN2A, ATF7IP2, EMP2, TEKT5, NUBP1, TVP23A, CIITA, DEXI, CLEC16A, SOCS1, TNP2, PRM3, PRM2, PRM1, LOC105371083, RMI2, LOC101927131, LITAF, SNN, TXNDC11, ZC3H7A, BCAR4, RSL1D1, GSPT1, TNFRSF17, SNX29, CPPED1, MIR4718, SHISA9, ERCC4, LOC101927311, LOC101927348, MKL2, MIR193BHG, MIR193B, MIR365A, LOC105447648, PARN, BFAR, PLA2G10, NPIPA2, NPIPA3, ABCC6P2, NOMO1, MIR3179-2, MIR3179-3, MIR3179-1, MIR3179-4, MIR3670-2, MIR3670-4, MIR3670-1, MIR3670-3, MIR3180-3, MIR3180-1, MIR3180-2, LOC100288162, MIR6511A2, MIR6511A3, MIR6511A4, MIR6511A1, MIR6770-2, MIR6770-1, MIR6770-3, NPIPA1, PDXDC1, NTAN1, RRN3, LOC100505915, PKD1P6-NPIPP1, MIR3180-4, NPIPA5, MPV17L, C16orf45, KIAA0430, MIR6506, NDE1, MIR484, MYH11, FOPNL, ABCC1, ABCC6, NOMO3, PKD1P1, NPIPA7, NPIPA8, XYLT1, LOC102723692, NOMO2, ABCC6P1, RPS15A, ARL6IP1, SMG1, TMC7, LOC102723385, COQ7, ITPRIPL2, SYT17, CLEC19A, TMC5, GDE1, CCP110, C16orf62, KNOP1, IQCK, GPRC5B, GPR139, GP2, UMOD, PDILT, ACSM5, ACSM2A, ACSM2B, ACSM1, THUMPD1, ACSM3, ERI2, LOC81691, DCUN1D3, LYRM1, DNAH3, TMEM159, ZP2, ANKS4B, CRYM, CRYM-AS1, SNX29P1, NPIPB3, LOC100190986, SMG1P3, MIR3680-2, MIR3680-1, SLC7A5P2, LOC101927814, METTL9, IGSF6, OTOA, RRN3P1, NPIPB4, UQCRC2, PDZD9, C16orf52, VWA3A, EEF2K, POLR3E, CDR2, MFSD13B, RRN3P3, SMG1P1, NPIPB5, LOC653786, HS3ST2, USP31, SCNN1G, SCNN1B, COG7, GGA2, EARS2, UBFD1, NDUFAB1, PALB2, DCTN5, PLK1, ERN2, CHP2, PRKCB, MIR1273H, CACNG3, RBBP6, LINC01567, TNRC6A, SLC5A11, ARHGAP17, LOC554206, LOC283887, LCMT1-AS1, LCMT1, LCMT1-AS2, AQP8, ZKSCAN2, HS3ST4, MIR548W, C16orf82, KDM8, NSMCE1, FLJ21408, IL4R, IL21R, IL21R-AS1, GTF3C1, KIAA0556, GSG1L, XPO6, SBK1, NPIPB6, EIF3CL, EIF3C, MIR6862-1, MIR6862-2, CLN3, APOBR, IL27, NUPR1, SGF29, SULT1A2, SULT1A1, NPIPB8, NPIPB9, ATXN2L, TUFM, MIR4721, SH2B1, ATP2A1, ATP2A1-AS1, RABEP2, CD19, NFATC2IP, MIR4517, SPNS1, LAT, RRN3P2, SNX29P2, NPIPB11, SMG1P6, BOLA2-SMG1P6, LOC606724, BOLA2, BOLA2B, SLX1B, SLX1A, SLX1B-SULT1A4, SLX1A-SULT1A3, SULT1A4, SULT1A3, LOC388242, LOC613038, SMG1P2, SPN, QPRT, C16orf54, ZG16, KIF22, MAZ, PRRT2, PAGR1, MVP, CDIPT, CDIPT-AS1, SEZ6L2, ASPHD1, KCTD13, TMEM219, TAOK2, HIRIP3, INO80E, DOC2A, C16orf92, FAM57B, ALDOA, PPP4C, TBX6, YPEL3, LOC101928595, GDPD3, MAPK3, CORO1A, LOC613037, SMG1P5, CD2BP2, TBC1D10B, MYLPF, SEPT1, ZNF48, ZNF771, DCTPP1, SEPHS2, ITGAL, MIR4518, ZNF768, ZNF747, ZNF764, ZNF688, ZNF785, ZNF689, PRR14, FBRS, LOC730183, SRCAP, SNORA30, TMEM265, PHKG2, CCDC189, RNF40, ZNF629, BCL7C, MIR4519, MIR762HG, MIR762, CTF1, FBXL19-AS1, FBXL19, ORAI3, SETD1A, HSD3B7, STX1B, STX4, ZNF668, ZNF646, PRSS53, VKORC1, BCKDK, KAT8, PRSS8, PRSS36, FUS, PYCARD, PYCARD-AS1, TRIM72, PYDC1, ITGAM, ITGAX, ITGAD, COX6A2, ZNF843, ARMC5, TGFB1I1, SLC5A2, C16orf58, AHSP, FRG2KP, YBX3P1, CLUHP3, ZNF720, ZNF267, LOC102723753, HERC2P4, TP53TG3D, LOC390705, TP53TG3, TP53TG3B, TP53TG3C, SLC6A10P, ENPP7P13, LINC00273, UBE2MP1, LINC01566, FRG2DP, TP53TG3HP, FLJ26245, ANKRD26P1, SHCBP1, VPS35, ORC6, MYLK3, C16orf87, GPT2, DNAJA2, NETO2, ITFG1-AS1, ITFG1, PHKB, LOC100507534, LOC101927132, ABCC12, ABCC11, LONP2, MIR5095, LOC100507577, SIAH1, N4BP1, CBLN1, C16orf78, ZNF423, CNEP1R1, HEATR3, PAPD5, ADCY7, MIR6771, BRD7, NKD1, SNX20, LOC101927272, NOD2, CYLD, MIR3181, LOC101927334, SALL1, LINC01571, C16orf97, LINC00919, LOC102467079, CASC22, TOX3, CASC16, LOC105371267, CHD9, LOC643802, LOC102723373, RBL2, AKTIP, RPGRIP1L, FTO, FTO-IT1, LOC100996338, IRX3, LOC100996345, LOC101927480, CRNDE, IRX5, IRX6, MMP2, LPCAT2, CAPNS2, SLC6A2, CES1P2, CES1P1, CES1, CES5A, LOC283856, GNAO1, DKFZP434H168, MIR3935, AMFR, NUDT21, OGFOD1, BBS2, MT4, MT3, MT2A, MT1L, MT1E, MT1M, MT1JP, MT1A, MT1DP, MT1B, MT1F, MT1G, MT1H, MT1IP, MT1X, NUP93, MIR138-2, SLC12A3, MIR6863, HERPUD1, CETP, NLRC5, CPNE2, FAM192A, RSPRY1, ARL2BP, PLLP, CCL22, CX3CL1, CCL17, CIAPIN1, COQ9, POLR2C, DOK4, CCDC102A, ADGRG5, ADGRG1, ADGRG3, DRC7, KATNB1, KIFC3, MIR6772, LOC388282, CNGB1, TEPP, ZNF319, USB1, MMP15, CFAP20, CSNK2A2, CCDC113, PRSS54, GINS3, NDRG4, SETD6, CNOT1, SNORA46, SNORA50A, SLC38A7, GOT2, APOOP5, LOC101927580, LOC729159, MIR4426, CDH8, CDH11, LOC101927650, LINC00922, CDH5, LINC00920, BEAN1, BEAN1-AS1, TK2, CKLF, CKLF-CMTM1, CMTM1, CMTM2, CMTM3, CMTM4, DYNC1LI2, LOC106699570, TERB1, NAE1, CA7, PDP2, CDH16, RRAD, FAM96B, CES2, CES3, CES4A, CBFB, C16orf70, B3GNT9, TRADD, FBXL8, HSF4, NOL3, KIAA0895L, EXOC3L1, E2F4, ELMO3, MIR328, LRRC29, TMEM208, FHOD1, SLC9A5, PLEKHG4, KCTD19, LRRC36, TPPP3, ZDHHC1, HSD11B2, ATP6V0D1, AGRP, LOC100505942, FAM65A, CTCF, CARMIL2, ACD, PARD6A, ENKD1, C16orf86, GFOD2, RANBP10, TSNAXIP1, CENPT, THAP11, NUTF2, EDC4, NRN1L, PSKH1, CTRL, PSMB10, LCAT, SLC12A4, DPEP3, DPEP2, LOC100131303, DDX28, DUS2, NFATC3, ESRP2, MIR6773, PLA2G15, SLC7A6, SLC7A6OS, PRMT7, SMPD3, ZFP90, CDH3, CDH1, MIR7641-2, TANGO6, HAS3, CHTF8, UTP4, SNTB2, VPS4A, PDF, COG8, NIP7, TMED6, TERF2, CYB5B, MIR1538, NFAT5, NQO1, NOB1, WWP2, MIR140, CLEC18A, PDXDC2P, MIR1972-2, MIR1972-1, PDPR, LOC400541, CLEC18C, LOC105371328, SMG1P7, EXOSC6, AARS, DDX19B, LOC100506083, DDX19A, ST3GAL2, FUK, COG4, SF3B3, SNORD111B, SNORD111, IL34, MTSS1L, VAC14, VAC14-AS1, HYDIN, CMTR2, CALB2, ZNF23, ZNF19, LOC105371335, CHST4, TAT-AS1, TAT, MARVELD3, PHLPP2, SNORA70D, AP1G1, SNORD71, ATXN1L, ZNF821, IST1, PKD1L3, DHODH, HP, HPR, TXNL4B, DHX38, PMFBP1, LINC01572, ZFHX3, HCCAT5, C16orf47, LINC01568, LOC101928035, PSMD7, LOC283922, NPIPB15, LOC105376772, CLEC18B, GLG1, RFWD3, MLKL, FA2H, WDR59, ZNRF1, LDHD, ZFP1, CTRB2, CTRB1, LOC100506281, BCAR1, CFDP1, TMEM170A, CHST6, CHST5, TMEM231, GABARAPL2, ADAT1, KARS, TERF2IP, CNTNAP4, LOC101928203, MIR4719, MON1B, SYCE1L, ADAMTS18, NUDT7, VAT1L, CLEC3A, WWOX, MAF, MAFTRR, LINC01229, LOC102724084, DYNLRB2, LINC01227, CDYL2, PRCAT47, CMC2, CENPN, ATMIN, C16orf46, GCSH, PKD1L2, BCO1, GAN, MIR4720, CMIP, MIR7854, MIR6504, LOC100129617, PLCG2, SDR42E1, HSD17B2, MPHOSPH6, CDH13, MIR8058, LOC101928446, LOC101928417, MIR3182, LOC102724163, HSBP1, MLYCD, OSGIN1, NECAB2, SLC38A8, MBTPS1, HSDL1, DNAAF1, TAF1C, ADAD2, KCNG4, WFDC1, ATP2C2, TLDC1, COTL1, KLHL36, USP10, CRISPLD2, ZDHHC7, KIAA0513, FAM92B, LOC400548 | arr[GRCh37] 16p13.3q24.1(83886_85313662)x3 |
| 2  CRC | Gain | 20 | p13 | p12.3 | 6,603 | DEFB125, DEFB126, DEFB127, DEFB128, DEFB129, DEFB132, C20orf96, ZCCHC3, NRSN2-AS1, SOX12, NRSN2, TRIB3, RBCK1, TBC1D20, CSNK2A1, TCF15, SRXN1, SCRT2, SLC52A3, FAM110A, ANGPT4, RSPO4, PSMF1, LOC105372493, TMEM74B, C20orf202, RAD21L1, SNPH, SDCBP2, FKBP1A-SDCBP2, SDCBP2-AS1, FKBP1A, MIR6869, NSFL1C, SIRPB2, SIRPD, SIRPB1, SIRPG, SIRPG-AS1, LOC100289473, SIRPA, LOC727993, PDYN, STK35, LOC388780, TGM3, TGM6, SNRPB, SNORD119, ZNF343, TMC2, NOP56, MIR1292, SNORD110, SNORA51, SNORD86, SNORD56, SNORD57, IDH3B, EBF4, CPXM1, C20orf141, TMEM239, PCED1A, VPS16, PTPRA, GNRH2, MRPS26, OXT, AVP, UBOX5-AS1, UBOX5, FASTKD5, LZTS3, DDRGK1, ITPA, SLC4A11, C20orf194, ATRN, GFRA4, ADAM33, SIGLEC1, HSPA12B, C20orf27, SPEF1, CENPB, CDC25B, LOC101929125, AP5S1, MAVS, PANK2, MIR103A2, MIR103B2, RNF24, SMOX, LINC01433, ADRA1D, PRNP, PRND, PRNT, RASSF2, SLC23A2, TMEM230, PCNA, PCNA-AS1, CDS2, PROKR2, LINC00658, LOC643406, LINC00654, LOC101929207, GPCPD1, C20orf196, CHGB, TRMT6, MCM8, MCM8-AS1, CRLS1, LRRN4, FERMT1, CASC20 | arr[GRCh37] 20p13p12.3(69093_6672360)x3 |
| 2  CRC | Gain | 20 | q11.21 | q13.33 | 33,393 | LINC01598, FRG1BP, FRG1DP, MLLT10P1, DEFB115, DEFB116, DEFB118, DEFB119, DEFB121, DEFB122, DEFB123, DEFB124, REM1, LINC00028, HM13, HM13-AS1, ID1, MIR3193, COX4I2, BCL2L1, ABALON, TPX2, MYLK2, FOXS1, DUSP15, TTLL9, PDRG1, XKR7, MIR7641-2, CCM2L, HCK, TM9SF4, TSPY26P, PLAGL2, POFUT1, MIR1825, KIF3B, ASXL1, NOL4L, LOC101929698, LOC149950, C20orf203, COMMD7, DNMT3B, MAPRE1, SUN5, BPIFB2, BPIFB6, BPIFB3, BPIFB4, BPIFA2, BPIFA4P, BPIFA3, BPIFA1, BPIFB1, CDK5RAP1, SNTA1, CBFA2T2, NECAB3, C20orf144, ACTL10, E2F1, PXMP4, ZNF341, ZNF341-AS1, CHMP4B, RALY-AS1, RALY, MIR4755, EIF2S2, ASIP, AHCY, ITCH, MIR644A, DYNLRB1, MAP1LC3A, PIGU, TP53INP2, NCOA6, HMGB3P1, GGT7, ACSS2, GSS, MYH7B, MIR499A, MIR499B, TRPC4AP, EDEM2, PROCR, MMP24, MMP24-AS1, EIF6, FAM83C-AS1, FAM83C, UQCC1, GDF5, MIR1289-1, CEP250, C20orf173, ERGIC3, FER1L4, SPAG4, CPNE1, RBM12, NFS1, ROMO1, RBM39, PHF20, SCAND1, CNBD2, NORAD, EPB41L1, AAR2, DLGAP4, DLGAP4-AS1, MYL9, TGIF2, TGIF2-C20orf24, C20orf24, SLA2, NDRG3, DSN1, SOGA1, TLDC2, SAMHD1, RBL1, MROH8, RPN2, GHRH, MANBAL, SRC, BLCAP, NNAT, LINC00489, LOC100287792, CTNNBL1, VSTM2L, TTI1, RPRD1B, TGM2, KIAA1755, LOC149684, BPI, LBP, SNHG17, SNORA71B, SNORA71A, SNORA71C, SNORA71D, SNHG11, SNORA71E, SNORA60, RALGAPB, MIR548O2, ADIG, ARHGAP40, SLC32A1, ACTR5, PPP1R16B, FAM83D, DHX35, LOC339568, LINC01370, MAFB, LOC100128988, TOP1, PLCG1-AS1, PLCG1, MIR6871, ZHX3, LPIN3, EMILIN3, CHD6, PTPRT, LOC101927159, SRSF6, L3MBTL1, SGK2, IFT52, MYBL2, GTSF1L, LOC105372626, TOX2, JPH2, OSER1, OSER1-AS1, GDAP1L1, FITM2, R3HDML, HNF4A, HNF4A-AS1, MIR3646, LINC01430, LINC01620, TTPAL, SERINC3, PKIG, ADA, LINC01260, KCNK15-AS1, WISP2, KCNK15, RIMS4, YWHAB, PABPC1L, TOMM34, STK4-AS1, STK4, KCNS1, WFDC5, WFDC12, PI3, SEMG1, SEMG2, SLPI, MATN4, RBPJL, SDC4, SYS1, SYS1-DBNDD2, TP53TG5, DBNDD2, PIGT, MIR6812, WFDC2, SPINT3, WFDC6, EPPIN-WFDC6, EPPIN, WFDC8, WFDC9, WFDC10A, WFDC11, WFDC10B, WFDC13, MIR3617, SPINT4, WFDC3, DNTTIP1, UBE2C, TNNC2, SNX21, ACOT8, ZSWIM3, ZSWIM1, SPATA25, NEURL2, CTSA, PLTP, PCIF1, ZNF335, MMP9, SLC12A5, NCOA5, CD40, CDH22, SLC35C2, ELMO2, ZNF663P, MKRN7P, ZNF334, OCSTAMP, SLC13A3, TP53RK, SLC2A10, EYA2, MIR3616, ZMYND8, LOC100131496, LOC101927377, NCOA3, SULF2, LINC01522, LINC01523, LINC00494, PREX1, ARFGEF2, CSE1L-AS1, CSE1L, STAU1, DDX27, ZNFX1, ZFAS1, SNORD12C, SNORD12B, SNORD12, KCNB1, PTGIS, B4GALT5, SLC9A8, SPATA2, LOC105372653, RNF114, SNAI1, TRERNA1, UBE2V1, TMEM189-UBE2V1, TMEM189, LINC01273, CEBPB-AS1, CEBPB, LINC01272, LINC01270, LINC01271, PTPN1, MIR645, FAM65C, MIR1302-5, LOC100506175, PARD6B, BCAS4, ADNP, ADNP-AS1, DPM1, MOCS3, KCNG1, NFATC2, MIR3194, ATP9A, SALL4, LINC01429, ZFP64, LINC01524, TSHZ2, LOC101927770, ZNF217, LOC105372672, SUMO1P1, BCAS1, MIR4756, CYP24A1, PFDN4, DOK5, LINC01441, LINC01440, CBLN4, MC3R, FAM210B, AURKA, CSTF1, CASS4, RTFDC1, GCNT7, FAM209A, FAM209B, LOC105372682, TFAP2C, BMP7, BMP7-AS1, MIR4325, SPO11, RAE1, MTRNR2L3, RBM38, CTCFL, PCK1, ZBP1, PMEPA1, NKILA, MIR4532, C20orf85, ANKRD60, PPP4R1L, RAB22A, VAPB, APCDD1L, APCDD1L-AS1, LOC79160, STX16, STX16-NPEPL1, NPEPL1, LOC105372695, MIR296, MIR298, GNAS-AS1, GNAS, LOC101927932, NELFCD, CTSZ, TUBB1, ATP5E, SLMO2-ATP5E, PRELID3B, ZNF831, EDN3, PHACTR3, LOC100506384, SYCP2, FAM217B, PPP1R3D, CDH26, C20orf197, LOC729296, MIR646HG, MIR646, LOC101928048, MIR4533, MIR548AG2, LOC100506470, CDH4, MIR1257, TAF4, MIR3195, LSM14B, PSMA7, SS18L1, MTG2, HRH3, OSBPL2, ADRM1, LAMA5, MIR4758, LAMA5-AS1, RPS21, CABLES2, RBBP8NL, GATA5, C20orf166-AS1, MIR1-1HG, MIR1-1, MIR133A2, SLCO4A1, SLCO4A1-AS1, NTSR1, LINC00659, MRGBP, OGFR-AS1, OGFR, COL9A3, TCFL5, DPH3P1, DIDO1, GID8, SLC17A9, BHLHE23, LOC63930, LINC00029, LINC01056, HAR1B, HAR1A, MIR124-3, YTHDF1, BIRC7, MIR3196, NKAIN4, FLJ16779, ARFGAP1, MIR4326, COL20A1, CHRNA4, LOC100130587, KCNQ2, EEF1A2, PPDPF, PTK6, SRMS, FNDC11, HELZ2, GMEB2, LOC100505771, STMN3, RTEL1, RTEL1-TNFRSF6B, TNFRSF6B, ARFRP1, ZGPAT, LIME1, SLC2A4RG, ZBTB46, ZBTB46-AS1, ABHD16B, TPD52L2, DNAJC5, MIR941-1, MIR941-4, MIR941-3, MIR941-2, MIR941-5, UCKL1, MIR1914, MIR647, UCKL1-AS1, ZNF512B, SAMD10, PRPF6, LINC00176, SOX18, TCEA2, RGS19, MIR6813, OPRL1, LKAAEAR1, NPBWR2, MYT1, PCMTD2 | arr[GRCh37] 20q11.21q13.33(29519155_62912463)x3 |
| 2  CRC | Loss | 8 | p23.3 | p11.22 | 39,453 | RPL23AP53, ZNF596, FAM87A, FBXO25, TDRP, ERICH1, ERICH1-AS1, LOC401442, LOC286083, DLGAP2, DLGAP2-AS1, LOC101927752, CLN8, MIR3674, MIR596, ARHGEF10, LOC101928058, KBTBD11-OT1, KBTBD11, MYOM2, MIR7160, LOC101927815, CSMD1, LOC100287015, MCPH1, ANGPT2, MCPH1-AS1, MIR8055, AGPAT5, MIR4659A, MIR4659B, XKR5, GS1-24F4.2, DEFB1, DEFA6, DEFA4, DEFA8P, DEFA9P, DEFA10P, DEFA1, DEFA1B, DEFT1P2, DEFT1P, DEFA3, DEFA11P, DEFA5, LINC00965, FAM66B, DEFB109P1B, USP17L1, USP17L4, ZNF705G, DEFB4B, DEFB103B, DEFB103A, SPAG11B, DEFB104A, DEFB104B, DEFB106B, DEFB106A, DEFB105B, DEFB105A, DEFB107B, DEFB107A, PRR23D1, PRR23D2, FAM90A7P, FAM90A10P, SPAG11A, DEFB4A, ZNF705B, FAM66E, USP17L8, USP17L3, MIR548I3, FAM86B3P, SGK223, CLDN23, MFHAS1, ERI1, MIR4660, PPP1R3B, LOC101929128, LOC157273, TNKS, MIR597, LINC00599, MIR124-1, MSRA, LINCR-0001, PRSS55, RP1L1, MIR4286, C8orf74, SOX7, PINX1, MIR1322, LOC101929229, XKR6, MIR598, LOC101929269, MTMR9, SLC35G5, TDH, FAM167A-AS1, FAM167A, BLK, LINC00208, GATA4, SNORA99, C8orf49, NEIL2, FDFT1, CTSB, DEFB136, DEFB135, DEFB134, LOC100133267, DEFB130, ZNF705D, FAM66D, LOC392196, USP17L7, USP17L2, FAM90A2P, FAM86B1, FAM66A, LOC649352, DEFB109P1, FAM90A25P, FAM86B2, LOC100506990, LOC729732, MIR5692A1, MIR5692A2, LONRF1, MIR3926-1, MIR3926-2, LOC340357, LINC00681, KIAA1456, DLC1, C8orf48, LOC102725080, SGCZ, MIR383, TUSC3, MSR1, FGF20, MICU3, ZDHHC2, CNOT7, VPS37A, MTMR7, SLC7A2, PDGFRL, MTUS1, MIR548V, FGL1, PCM1, ASAH1, LOC101929066, NAT1, NAT2, PSD3, LOC100128993, SH2D4A, CSGALNACT1, INTS10, LPL, SLC18A1, ATP6V1B2, LZTS1, LZTS1-AS1, LOC102467222, LOC286114, LOC101929172, GFRA2, DOK2, XPO7, NPM2, FGF17, DMTN, FAM160B2, NUDT18, HR, REEP4, LGI3, SFTPC, BMP1, PHYHIP, MIR320A, POLR3D, LOC100507071, PIWIL2, SLC39A14, PPP3CC, SORBS3, PDLIM2, C8orf58, CCAR2, BIN3, BIN3-IT1, EGR3, PEBP4, LOC101929237, RHOBTB2, TNFRSF10B, LOC286059, LOC254896, TNFRSF10C, TNFRSF10D, TNFRSF10A, LOC389641, CHMP7, R3HCC1, LOXL2, LOC100507156, ENTPD4, SLC25A37, NKX3-1, NKX2-6, STC1, ADAM28, LOC101929294, ADAMDEC1, ADAM7, LOC101929315, NEFM, NEFL, MIR6841, DOCK5, MIR6876, GNRH1, KCTD9, CDCA2, EBF2, PPP2R2A, BNIP3L, PNMA2, DPYSL2, ADRA1A, STMN4, TRIM35, PTK2B, MIR6842, CHRNA2, EPHX2, CLU, MIR6843, SCARA3, MIR3622B, MIR3622A, CCDC25, ESCO2, PBK, SCARA5, MIR4287, NUGGC, ELP3, PNOC, ZNF395, FBXO16, FZD3, MIR4288, MIR7641-2, EXTL3-AS1, EXTL3, INTS9, HMBOX1, KIF13B, DUSP4, LINC00589, LOC101929450, LOC101929470, FAM183CP, MIR3148, SARAF, LEPROTL1, MBOAT4, DCTN6, RBPMS-AS1, RBPMS, GTF2E2, SMIM18, GSR, UBXN8, PPP2CB, TEX15, PURG, WRN, NRG1, NRG1-IT1, NRG1-IT3, FUT10, MAK16, TTI2, RNF122, DUSP26, LINC01288, UNC5D, LOC101929550, KCNU1, MIR1268A, LINC01605, ZNF703, LOC101929622, LOC102723701, ERLIN2, LOC728024, PROSC, ADGRA2, BRF2, RAB11FIP1, GOT1L1, ADRB3, EIF4EBP1, ASH2L, STAR, LSM1, BAG4, DDHD2, PLPP5, WHSC1L1, LETM2, FGFR1, C8orf86, RNF5P1, TACC1, PLEKHA2, HTRA4, TM2D2, ADAM9, ADAM32, ADAM5, ADAM3A, LOC100130964, ADAM18, ADAM2 | arr[GRCh37] 8p23.3p11.22(172416_39625680)x1 |
| 2  CRC | Loss | 12 | p13.33 | p11.1 | 34,639 | IQSEC3, LOC574538, SLC6A12, LOC101929384, SLC6A13, LOC102723544, KDM5A, CCDC77, B4GALNT3, NINJ2, LOC105369595, LOC100049716, WNK1, RAD52, ERC1, LINC00942, FBXL14, WNT5B, MIR3649, ADIPOR2, CACNA2D4, LRTM2, LINC00940, DCP1B, CACNA1C-IT2, CACNA1C, CACNA1C-AS4, CACNA1C-IT3, CACNA1C-AS2, CACNA1C-AS1, LOC283440, FKBP4, ITFG2, NRIP2, LOC100507424, FOXM1, RHNO1, TULP3, TEAD4, TSPAN9, PRMT8, THCAT155, CRACR2A, PARP11, CCND2-AS1, CCND2, TIGAR, FGF23, FGF6, C12orf4, RAD51AP1, DYRK4, AKAP3, NDUFA9, LOC101929549, GALNT8, KCNA6, KCNA1, KCNA5, LOC101929584, NTF3, ANO2, VWF, CD9, PLEKHG6, TNFRSF1A, SCNN1A, LTBR, CD27-AS1, CD27, TAPBPL, VAMP1, MRPL51, NCAPD2, SCARNA10, GAPDH, IFFO1, NOP2, CHD4, SCARNA11, LPAR5, ACRBP, ING4, ZNF384, PIANP, COPS7A, MLF2, PTMS, LAG3, CD4, GPR162, P3H3, GNB3, CDCA3, USP5, TPI1, SPSB2, LOC105369632, RPL13P5, DSTNP2, LRRC23, ENO2, ATN1, C12orf57, PTPN6, LOC105369635, MIR200C, MIR141, PHB2, SCARNA12, EMG1, LPCAT3, C1S, C1R, C1RL, C1RL-AS1, RBP5, CLSTN3, PEX5, ACSM4, CD163L1, CD163, APOBEC1, GDF3, DPPA3, CLEC4C, NANOGNB, NANOG, SLC2A14, SLC2A3, FOXJ2, C3AR1, NECAP1, CLEC4A, POU5F1P3, ZNF705A, FAM66C, FAM90A1, FAM86FP, LOC101927905, LINC00937, CLEC6A, CLEC4D, CLEC4E, AICDA, MFAP5, RIMKLB, A2ML1, PHC1, M6PR, KLRG1, LINC00612, A2M-AS1, A2M, PZP, A2MP1, MIR1244-4, MIR1244-3, MIR1244-1, MIR1244-2, LINC00987, LOC642846, LOC101930452, LOC101928030, DDX12P, KLRB1, LOC374443, CLEC2D, CLECL1, CD69, KLRF1, CLEC2B, KLRF2, CLEC2A, LOC100506159, LOC400002, CLEC12A, CLEC1B, CLEC12B, LOC102724020, CLEC9A, CLEC1A, CLEC7A, OLR1, TMEM52B, GABARAPL1, KLRD1, LOC101928100, KLRK1, KLRC4-KLRK1, KLRC4, KLRC3, KLRC2, KLRC1, KLRA1P, MAGOHB, STYK1, YBX3, LOC101928162, TAS2R7, TAS2R8, TAS2R9, TAS2R10, PRR4, PRH1-PRR4, PRH1, TAS2R13, PRH2, PRH1-TAS2R14, TAS2R14, TAS2R50, TAS2R20, TAS2R19, TAS2R31, TAS2R46, TAS2R43, TAS2R30, SMIM10L1, TAS2R42, PRB3, PRB4, PRB1, PRB2, LINC01252, ETV6, BCL2L14, LRP6, MANSC1, LOH12CR2, BORCS5, DUSP16, CREBL2, GPR19, CDKN1B, APOLD1, MIR613, DDX47, RPL13AP20, GPRC5A, MIR614, GPRC5D, HEBP1, LOC100506314, HTR7P1, FAM234B, MIR7641-2, GSG1, EMP1, LINC01559, GRIN2B, ATF7IP, PLBD1, PLBD1-AS1, GUCY2C, HIST4H4, H2AFJ, WBP11, C12orf60, SMCO3, ART4, MGP, ERP27, ARHGDIB, PDE6H, LINC01489, RERG, RERG-AS1, PTPRO, EPS8, STRAP, DERA, SLC15A5, MGST1, LMO3, SKP1P2, MIR3974, RERGL, PIK3C2G, PLCZ1, CAPZA3, PLEKHA5, AEBP2, LOC100506393, PDE3A, SLCO1C1, SLCO1B3, SLCO1B7, SLCO1B1, SLCO1A2, IAPP, PYROXD1, RECQL, GOLT1B, SPX, GYS2, LDHB, KCNJ8, ABCC9, CMAS, ST8SIA1, C2CD5, LOC105369691, ETNK1, LOC101928441, SOX5, MIR920, LOC101928471, LINC00477, BCAT1, C12orf77, LOC645177, LRMP, CASC1, LYRM5, KRAS, LMNTD1, MIR4302, RASSF8-AS1, RASSF8, BHLHE41, SSPN, ITPR2, ASUN, FGFR1OP2, TM7SF3, MED21, C12orf71, STK38L, ARNTL2, ARNTL2-AS1, SMCO2, PPFIBP1, REP15, MRPS35, MANSC4, KLHL42, PTHLH, CCDC91, FAR2, LOC100506606, ERGIC2, OVCH1-AS1, OVCH1, TMTC1, IPO8, CAPRIN2, LOC645485, LINC00941, TSPAN11, DDX11-AS1, DDX11, FAM60A, FLJ13224, DENND5B, DENND5B-AS1, ETFBKMT, AMN1, H3F3C, LOC105369723, KIAA1551, BICD1, FGD4, DNM1L, YARS2, PKP2, SYT10, ALG10 | arr[GRCh37] 12p13.33p11.1(189399_34828211)x1 |
| 2  CRC | Loss | 17 | p13.3 | p11.2 | 19,819 | VPS53, FAM57A, GEMIN4, DBIL5P, GLOD4, MRM3, NXN, LOC101927727, TIMM22, ABR, MIR3183, BHLHA9, TUSC5, YWHAE, CRK, MYO1C, INPP5K, PITPNA-AS1, PITPNA, SLC43A2, SCARF1, RILP, PRPF8, TLCD2, MIR22HG, MIR22, WDR81, SERPINF2, SERPINF1, SMYD4, RPA1, RTN4RL1, LOC105371485, DPH1, OVCA2, MIR132, MIR212, HIC1, SMG6, LOC101927839, SRR, TSR1, SNORD91B, SNORD91A, SGSM2, MNT, LOC284009, METTL16, PAFAH1B1, CLUH, MIR6776, LOC105371592, MIR1253, RAP1GAP2, LOC101927911, OR1D5, OR1D2, OR1G1, OR1A2, OR1A1, OR1D4, OR3A2, OR3A1, OR3A4P, OR1E1, OR3A3, OR1E2, SPATA22, ASPA, TRPV3, TRPV1, SHPK, CTNS, TAX1BP3, P2RX5-TAX1BP3, EMC6, P2RX5, ITGAE, GSG2, NCBP3, CAMKK1, P2RX1, ATP2A3, ZZEF1, CYB5D2, ANKFY1, UBE2G1, LOC103021295, SPNS3, SPNS2, MYBBP1A, GGT6, SMTNL2, ALOX15, PELP1, LOC101559451, ARRB2, MED11, CXCL16, ZMYND15, TM4SF5, VMO1, GLTPD2, PSMB6, PLD2, MINK1, CHRNE, C17orf107, GP1BA, SLC25A11, RNF167, PFN1, ENO3, SPAG7, CAMTA2, MIR6864, MIR6865, INCA1, KIF1C, LOC102724009, SLC52A1, ZFP3, ZNF232, LOC101928000, USP6, ZNF594, LOC100130950, SCIMP, RABEP1, NUP88, RPAIN, C1QBP, DHX33, LOC105371506, DERL2, MIS12, LOC728392, NLRP1, LOC339166, WSCD1, AIPL1, FAM64A, PITPNM3, KIAA0753, TXNDC17, MED31, C17orf100, MIR4520-1, MIR4520-2, ALOX15P1, SLC13A5, XAF1, FBXO39, TEKT1, ALOX12P2, ALOX12-AS1, ALOX12, RNASEK, RNASEK-C17orf49, C17orf49, MIR497HG, MIR195, MIR497, BCL6B, SLC16A13, SLC16A11, CLEC10A, ASGR2, ASGR1, DLG4, ACADVL, MIR324, DVL2, PHF23, GABARAP, CTDNEP1, ELP5, CLDN7, SLC2A4, YBX2, EIF5A, GPS2, NEURL4, ACAP1, KCTD11, TMEM95, TNK1, PLSCR3, TMEM256-PLSCR3, TMEM256, NLGN2, SPEM1, C17orf74, TMEM102, FGF11, CHRNB1, ZBTB4, SLC35G6, POLR2A, TNFSF12, TNFSF12-TNFSF13, TNFSF13, SENP3, SENP3-EIF4A1, EIF4A1, SNORA48, SNORD10, SNORA67, CD68, LOC100996842, MPDU1, SOX15, FXR2, SHBG, SAT2, ATP1B2, TP53, WRAP53, EFNB3, DNAH2, RPL29P2, KDM6B, TMEM88, NAA38, CYB5D1, CHD3, SCARNA21, LOC284023, KCNAB3, TRAPPC1, CNTROB, GUCY2D, ALOX15B, ALOX12B, MIR4314, ALOXE3, HES7, PER1, MIR6883, VAMP2, TMEM107, MIR4521, BORCS6, AURKB, LINC00324, CTC1, PFAS, SLC25A35, RANGRF, ARHGEF15, ODF4, LOC100128288, KRBA2, RPL26, RNF222, NDEL1, MYH10, CCDC42, SPDYE4, MFSD6L, PIK3R6, PIK3R5, NTN1, LOC101928266, STX8, CFAP52, USP43, DHRS7C, GSG1L2, GLP2R, RCVRN, GAS7, MYH13, MYHAS, MYH8, MYH4, MYH1, MYH2, MYH3, SCO1, ADPRM, TMEM220, MAGOH2P, TMEM220-AS1, LINC00675, PIRT, SHISA6, DNAH9, ZNF18, MAP2K4, MIR744, LINC00670, MYOCD, LOC101928418, LOC100128006, ARHGAP44, MIR1269B, ELAC2, HS3ST3A1, CDRT15P1, COX10-AS1, COX10, CDRT15, HS3ST3B1, MGC12916, LOC101928475, CDRT7, CDRT8, PMP22, MIR4731, TEKT3, CDRT4, TVP23C-CDRT4, TVP23C, CDRT1, TRIM16, ZNF286A, TBC1D26, CDRT15P2, MEIS3P1, LOC101928567, ADORA2B, ZSWIM7, TTC19, NCOR1, PIGL, MIR1288, CENPV, UBB, TRPV2, LRRC75A-AS1, SNORD49B, SNORD49A, SNORD65, LRRC75A, ZNF287, ZNF624, CCDC144A, USP32P1, FAM106CP, KRT16P2, TNFRSF13B, MPRIP, PLD6, FLCN, COPS3, NT5M, MED9, RASD1, PEMT, SMCR2, RAI1, RAI1-AS1, SMCR5, SREBF1, MIR6777, MIR33B, TOM1L2, DRC3, ATPAF2, GID4, DRG2, MYO15A, ALKBH5, LLGL1, FLII, MIEF2, TOP3A, SMCR8, SHMT1, MIR6778, EVPLL, FLJ35934, KRT17P5, KRT16P1, LGALS9C, USP32P2, FAM106A, CCDC144B, TBC1D28, ZNF286B, FOXO3B, TRIM16L, FBXW10, TVP23B, PRPSAP2, SLC5A10, FAM83G, GRAP, LOC79999, LOC388436, GRAPL, EPN2, EPN2-IT1, EPN2-AS1, B9D1, MIR1180, MAPK7, MFAP4, RNF112, SLC47A1, SNORA59B, SNORA59A, ALDH3A2, SLC47A2, ALDH3A1, ULK2, AKAP10, SPECC1 | arr[GRCh37] 17p13.3p11.2(400958_20220266)x1 |
| 2  CRC | Loss | 18 | q11.2 | q23 | 54,417 | SS18, PSMA8, TAF4B, LINC01543, KCTD1, MIR8057, PCAT18, AQP4, AQP4-AS1, CHST9, LOC105372038, CDH2, MIR302F, DSC3, DSC2, DSCAS, DSC1, DSG1, DSG1-AS1, DSG4, DSG3, DSG2, DSG2-AS1, TTR, B4GALT6, SLC25A52, TRAPPC8, RNF125, RNF138, MEP1B, GAREM1, WBP11P1, KLHL14, CCDC178, ASXL3, NOL4, DTNA, MAPRE2, ZNF397, ZSCAN30, ZNF271P, ZNF24, ZNF396, INO80C, MIR3975, GALNT1, MIR187, MIR3929, C18orf21, RPRD1A, SLC39A6, ELP2, LOC101927809, MOCOS, FHOD3, LOC105372071, TPGS2, KIAA1328, LOC105372069, CELF4, LOC105372068, SNORA111, MIR4318, MIR924HG, MIR924, MIR5583-2, MIR5583-1, LINC01477, KC6, PIK3C3, LINC00907, RIT2, SYT4, LINC01478, LOC105667213, SETBP1, MIR4319, SLC14A2, SLC14A2-AS1, SLC14A1, SIGLEC15, EPG5, PSTPIP2, ATP5A1, HAUS1, C18orf25, RNF165, LOXHD1, ST8SIA5, PIAS2, KATNAL2, TCEB3CL, TCEB3CL2, TCEB3C, TCEB3B, HDHD2, IER3IP1, SKOR2, MIR4527, SMAD2, ZBTB7C, CTIF, MIR4743, SMAD7, DYM, MIR4744, C18orf32, RPL17-C18orf32, MIR1539, RPL17, SNORD58C, SNORD58A, SNORD58B, LIPG, ACAA2, SCARNA17, SNHG22, MYO5B, MIR4320, CFAP53, MBD1, CXXC1, SKA1, MAPK4, MRO, ME2, ELAC1, SMAD4, MEX3C, LINC01630, DCC, MIR4528, LOC102724651, LOC101928167, MBD2, SNORA37, POLI, STARD6, C18orf54, DYNAP, RAB27B, CCDC68, LOC101927229, TCF4, TCF4-AS1, MIR4529, LINC01416, LINC01539, TXNL1, WDR7, LINC-ROR, BOD1L2, ST8SIA3, ONECUT2, FECH, NARS, LOC100505549, ATP8B1, NEDD4L, MIR122, MIR3591, ALPK2, SNORA108, LOC101927322, MALT1, ZNF532, OACYLP, SEC11C, GRP, RAX, CPLX4, LMAN1, CCBE1, PMAIP1, MC4R, CDH20, LINC01544, RNF152, PIGN, KIAA1468, TNFRSF11A, ZCCHC2, PHLPP1, BCL2, KDSR, VPS4B, SERPINB5, SERPINB12, SERPINB13, SERPINB4, SERPINB3, SERPINB11, SERPINB7, SERPINB2, SERPINB10, HMSD, SERPINB8, LINC00305, LOC284294, LINC01538, CDH7, CDH19, MIR5011, DSEL, LOC643542, TMX3, CCDC102B, DOK6, LOC105372179, CD226, RTTN, SOCS6, LOC101927481, LOC101060542, GTSCR1, LINC01541, LOC102724913, CBLN2, NETO1, MIR548AV, LOC100505797, LOC400655, LOC100505817, FBXO15, TIMM21, CYB5A, C18orf63, LOC101927606, FAM69C, CNDP2, CNDP1, LINC00909, ZNF407, ZADH2, TSHZ1, SMIM21, LOC100505853, LOC339298, ZNF516, LOC101927989, C18orf65, LINC00908, LINC00683, LOC101927651, LOC400661, LOC100131655, ZNF236, MBP, GALR1, LINC01029, SALL3, ATP9B, NFATC1, LOC284241, CTDP1, KCNG2, PQLC1, HSBP1L1, TXNL4A, RBFA, RBFADN, ADNP2, PARD6G-AS1, PARD6G | arr[GRCh37] 18q11.2q23(23590853_78007784)x1 |
| 2  CRC | Loss | 20 | p12.3 | p11.21 | 16,076 | BMP2, LINC01428, LOC101929288, LOC101929312, MIR8062, HAO1, TMX4, PLCB1, PLCB4, LAMP5-AS1, LAMP5, PAK5, LOC101929371, SNAP25-AS1, ANKEF1, SNAP25, MKKS, SLX4IP, JAG1, MIR6870, LOC101929395, LOC101929413, LOC339593, LINC00687, BTBD3, LOC101929486, LOC102606466, LOC100505515, SPTLC3, ISM1, ISM1-AS1, TASP1, ESF1, NDUFAF5, SEL1L2, MACROD2, FLRT3, MACROD2-IT1, MACROD2-AS1, LOC613266, KIF16B, SNRPB2, OTOR, PCSK2, BFSP1, DSTN, RRBP1, BANF2, SNX5, SNORD17, MGME1, OVOL2, PET117, KAT14, ZNF133, LINC00851, DZANK1, POLR3F, MIR3192, RBBP9, SEC23B, LINC00493, DTD1, LOC101929526, LINC00652, LOC100270804, C20orf78, SCP2D1, SLC24A3, LOC100130264, RIN2, NAA20, CRNKL1, CFAP61, INSM1, RALGAPA2, KIZ, KIZ-AS1, XRN2, NKX2-4, NKX2-2, LOC101929625, LOC101929608, PAX1, LINC01432, LINC01427, LOC284788, LINC00261, FOXA2, LINC01384 | arr[GRCh37] 20p12.3p11.21(6681990_22757960)x1 |
| 2  CRC | Loss | 21 | q11.2 | q22.3 | 33,753 | ANKRD30BP2, MIR3156-3, LOC102724188, POTED, MIR3118-1, MIR8069-1, MIR8069-2, CYP4F29P, ANKRD20A11P, LIPI, RBM11, ABCC13, HSPA13, SAMSN1, SAMSN1-AS1, LOC388813, NRIP1, USP25, MIR99AHG, MIR99A, MIRLET7C, MIR125B2, LINC01549, CXADR, BTG3, C21orf91-OT1, C21orf91, CHODL-AS1, CHODL, TMPRSS15, MIR548XHG, MIR548X, LINC00320, NCAM2, LINC00317, LINC01425, LOC101927843, LINC00308, MIR6130, D21S2088E, LOC101927869, LOC105372751, LOC339622, LINC00158, MIR155HG, MIR155, LINC00515, MRPL39, JAM2, ATP5J, GABPA, APP, CYYR1-AS1, CYYR1, ADAMTS1, ADAMTS5, MIR4759, LINC00113, LINC00314, LOC284825, LOC101927973, LINC00161, N6AMT1, LTN1, RWDD2B, USP16, CCT8, MAP3K7CL, LINC00189, BACH1, BACH1-IT2, GRIK1, GRIK1-AS2, GRIK1-AS1, CLDN17, LINC00307, CLDN8, KRTAP24-1, KRTAP25-1, KRTAP26-1, KRTAP27-1, KRTAP23-1, KRTAP13-2, MIR4327, KRTAP13-1, KRTAP13-3, KRTAP13-4, KRTAP15-1, KRTAP19-1, KRTAP19-2, KRTAP19-3, KRTAP19-4, KRTAP19-5, KRTAP19-6, KRTAP19-7, KRTAP22-2, KRTAP6-3, KRTAP6-2, KRTAP22-1, KRTAP6-1, KRTAP20-1, KRTAP20-4, KRTAP20-2, KRTAP20-3, KRTAP21-3, KRTAP21-2, KRTAP21-1, KRTAP8-1, KRTAP7-1, KRTAP11-1, KRTAP19-8, TIAM1, LOC150051, SOD1, SCAF4, HUNK, LINC00159, MIS18A, MRAP, URB1, SNORA80A, URB1-AS1, EVA1C, TCP10L, C21orf59, SYNJ1, PAXBP1-AS1, PAXBP1, C21orf62-AS1, C21orf62, LOC102724502, OLIG2, LINC00945, OLIG1, LOC101928107, LINC01548, IFNAR2, IL10RB-AS1, IL10RB, IFNAR1, IFNGR2, TMEM50B, DNAJC28, GART, SON, MIR6501, DONSON, CRYZL1, ITSN1, ATP5O, LINC00649, LOC101928126, SLC5A3, MRPS6, LINC00310, KCNE2, SMIM11A, SMIM11B, C21orf140, KCNE1, RCAN1, CLIC6, LINC00160, LINC01426, RUNX1, RUNX1-IT1, LOC100506403, MIR802, LOC101928269, LINC01436, SETD4, LOC100133286, CBR1, CBR3-AS1, CBR3, DOPEY2, MORC3, CHAF1B, CLDN14, SIM2, HLCS, RIPPLY3, LOC105372795, PIGP, TTC3, DSCR9, DSCR3, DYRK1A, KCNJ6, DSCR4, DSCR8, DSCR10, KCNJ15, LINC01423, ERG, LINC00114, ETS2, LOC101928398, LOC400867, LOC101928435, PSMG1, BRWD1, BRWD1-IT2, BRWD1-AS1, HMGN1, WRB-SH3BGR, WRB, LCA5L, SH3BGR, MIR6508, B3GALT5-AS1, B3GALT5, IGSF5, PCP4, DSCAM, MIR4760, DSCAM-AS1, DSCAM-IT1, LINC00323, MIR3197, BACE2, PLAC4, FAM3B, MX2, MX1, TMPRSS2, LINC00111, LINC00479, LINC00112, RIPK4, MIR6814, PRDM15, C2CD2, SNORA91, ZBTB21, ZNF295-AS1, UMODL1, UMODL1-AS1, ABCG1, TFF3, TFF2, TFF1, TMPRSS3, UBASH3A, RSPH1, LOC101930094, SLC37A1, LOC101928233, PDE9A, LOC101928311, WDR4, NDUFV3, ERVH48-1, MIR5692B, PKNOX1, CBS, CBSL, U2AF1, U2AF1L5, LOC106780825, FRGCA, CRYAA, LOC102724652, LINC00322, TCONS_00029157, LOC102724428, SIK1, LINC00319, LOC102724354, LINC00313, HSF2BP, MIR6070, RRP1B, PDXK, CSTB, RRP1, AATBC, AGPAT3, TRAPPC10, PWP2, C21orf33, ICOSLG, DNMT3L, LOC105372833, AIRE, PFKL, C21orf2, TRPM2, TRPM2-AS, LRRC3-AS1, LRRC3, TSPEAR, TSPEAR-AS1, TSPEAR-AS2, KRTAP10-1, KRTAP10-2, KRTAP10-3, KRTAP10-4, KRTAP10-5, KRTAP10-6, KRTAP10-7, KRTAP10-8, KRTAP10-9, KRTAP10-10, KRTAP10-11, KRTAP12-4, KRTAP12-3, KRTAP12-2, KRTAP12-1, KRTAP10-12, UBE2G2, LINC01424, SUMO3, PTTG1IP, ITGB2, ITGB2-AS1, LINC01547, FAM207A, LINC00163, PICSAR, SSR4P1, ADARB1, LINC00334, POFUT2, LOC642852, LINC00316, COL18A1, COL18A1-AS2, COL18A1-AS1, MIR6815, SLC19A1, LOC100129027, PCBP3, LOC101928796, COL6A1, COL6A2, FTCD, SPATC1L, LSS, MCM3AP-AS1, MCM3AP, YBEY, C21orf58, PCNT, DIP2A, DIP2A-IT1, S100B, PRMT2 | arr[GRCh37] 21q11.2q22.3(14344536_48097610)x1 |
| 2  LM | Gain | 2 | q33.1 | q33.3 | 8,003 | PLCL1, LOC101927619, SATB2, SATB2-AS1, LOC101927641, FTCDNL1, C2orf69, TYW5, C2orf47, SPATS2L, KCTD18, SGO2, AOX1, AOX3P-AOX2P, LOC100507140, LOC101927795, BZW1, CLK1, PPIL3, NIF3L1, ORC2, FAM126B, NDUFB3, CFLAR, CFLAR-AS1, CASP10, CASP8, ALS2CR12, TRAK2, STRADB, ALS2CR11, TMEM237, MPP4, ALS2, CDK15, FZD7, KIAA2012, LOC729224, SUMO1, NOP58, SNORD70, SNORD11B, SNORD11, BMPR2, FAM117B, ICA1L, WDR12, CARF, NBEAL1, CYP20A1, ABI2, RAPH1, CD28, CTLA4, ICOS, PARD3B, NRP2 | arr[GRCh37] 2q33.1q33.3(198664026_206666625)x3 |
| 2  LM | Gain | 5 | q33.3 | q35.3 | 22,349 | EBF1, LOC101927740, RNF145, LOC105377682, UBLCP1, IL12B, LOC285626, LOC285627, LOC101927766, ADRA1B, TTC1, PWWP2A, FABP6, CCNJL, C1QTNF2, ZBED8, SLU7, PTTG1, MIR3142HG, MIR3142, MIR146A, ATP10B, LOC285629, GABRB2, GABRA6, GABRA1, LINC01202, GABRG2, CCNG1, NUDCD2, HMMR, HMMR-AS1, MAT2B, LOC101927835, LOC102546299, CTB-7E3.1, LOC101927908, TENM2, CTB-178M22.2, WWC1, RARS, FBLL1, PANK3, MIR103A1, MIR103B1, SLIT3, LOC101927969, MIR218-2, LOC728095, MIR585, SPDL1, DOCK2, FAM196B, MIR378E, FOXI1, LINC01187, C5orf58, LCP2, LINC01366, KCNIP1, KCNMB1, CTD-2270F17.1, LOC105377716, GABRP, RANBP17, TLX3, MIR3912, NPM1, FGF18, SMIM23, FBXW11, STK10, EFCAB9, UBTD2, LOC100288254, SH3PXD2B, NEURL1B, MIR5003, LOC101928093, DUSP1, ERGIC1, LOC100268168, RPL26L1, ATP6V0E1, SNORA74B, CREBRF, BNIP1, NKX2-5, STC2, MIR8056, LOC285593, BOD1, LINC01484, LINC01485, CPEB4, C5orf47, HMP19, LINC01411, MSX2, MIR4634, FLJ16171, DRD1, SFXN1, HRH2, CPLX2, THOC3, LOC100996385, FAM153B, LOC100507387, LOC643201, SIMC1, KIAA1191, ARL10, MIR1271, NOP16, HIGD2A, CLTB, FAF2, RNF44, CDHR2, GPRIN1, SNCB, MIR4281, EIF4E1B, TSPAN17, LINC01574, UNC5A, HK3, UIMC1, ZNF346, FGFR4, NSD1, RAB24, PRELID1, MXD3, LMAN2, RGS14, SLC34A1, PFN3, F12, GRK6, PRR7-AS1, PRR7, DBN1, PDLIM7, DOK3, DDX41, FAM193B, TMED9, B4GALT7, LOC202181, FAM153A, LOC728554, PROP1, FAM153C, N4BP3, RMND5B, NHP2, GMCL1P1, HNRNPAB, PHYKPL, COL23A1, CLK4, ZNF354A, AACSP1, ZNF354B, ZFP2, ZNF454, GRM6, ZNF879, ZNF354C, ADAMTS2, RUFY1, LOC101928445, HNRNPH1, C5orf60, LOC105377763, CBY3, CANX, MAML1, LTC4S, MGAT4B, MIR1229, SQSTM1, C5orf45, LOC100996419, TBC1D9B, RNF130, MIR340, RASGEF1C, MAPK9, GFPT2, CNOT6, SCGB3A1, FLT4, OR2Y1, MGAT1, HEIH, LINC00847, ZFP62, BTNL8, BTNL3, BTNL9, MIR8089, OR2V1, OR2V2, LOC102577426, TRIM7, MIR4638, TRIM41, RACK1, SNORD96A, SNORD95, CTC-338M12.4, TRIM52, TRIM52-AS1 | arr[GRCh37] 5q33.3q35.3(158348911_180698312)x3 |
| 2  LM | Gain | 7 | p22.3 | q36.3 | 159,077 | LOC102723672, LOC100507642, LOC105375115, FAM20C, WI2-2373I1.2, LOC442497, PDGFA, HRAT92, PRKAR1B, LOC101927000, LOC101926963, DNAAF5, SUN1, GET4, ADAP1, COX19, CYP2W1, C7orf50, MIR339, GPR146, GPER1, ZFAND2A, LOC101927021, UNCX, MICALL2, INTS1, MAFK, TMEM184A, PSMG3, PSMG3-AS1, TFAMP1, ELFN1, ELFN1-AS1, MAD1L1, MIR4655, MRM2, NUDT1, SNX8, MIR6836, EIF3B, CHST12, LOC101927181, GRIFIN, LFNG, MIR4648, BRAT1, IQCE, TTYH3, AMZ1, GNA12, CARD11, LOC100129603, SDK1, FOXK1, AP5Z1, MIR4656, RADIL, PAPOLB, MMD2, RNF216P1, RBAK, RBAK-RBAKDN, RBAKDN, ZNF890P, WIPI2, SLC29A4, TNRC18, FBXL18, MIR589, LOC221946, ACTB, FSCN1, RNF216, RNF216-IT1, MIR6874, ZNF815P, OCM, CCZ1, RSPH10B2, RSPH10B, PMS2, AIMP2, EIF2AK1, ANKRD61, USP42, CYTH3, FAM220A, RAC1, DAGLB, KDELR2, GRID2IP, ZDHHC4, C7orf26, ZNF853, ZNF316, ZNF12, PMS2CL, CCZ1B, MIR3683, LOC100131257, C1GALT1, LOC101927354, COL28A1, LOC101927391, MIOS, RPA3, UMAD1, LOC100505921, GLCCI1, ICA1, LOC100505938, NXPH1, PER4, NDUFA4, PHF14, THSD7A, TMEM106B, VWDE, LOC102725191, SCIN, ARL4A, ETV1, DGKB, AGMO, MEOX2, LOC105375166, MEOX2-AS1, ISPD, ISPD-AS1, SOSTDC1, LRRC72, ANKMY2, BZW2, TSPAN13, AGR2, AGR3, AHR, KCCAT333, LOC101927630, SNX13, PRPS1L1, HDAC9, MIR1302-6, TWIST1, FERD3L, TWISTNB, MIR3146, TMEM196, LOC101927668, MACC1, MACC1-AS1, LOC100506098, LOC101927769, LOC101927811, ITGB8, ABCB5, SP8, RPL23P8, LINC01162, SP4, MIR1183, DNAH11, CDCA7L, RAPGEF5, STEAP1B, LOC100506178, LOC401312, LOC541472, IL6, TOMM7, SNORD93, FAM126A, KLHL7-AS1, KLHL7, NUPL2, GPNMB, MALSU1, IGF2BP3, RPS2P32, TRA2A, CLK2P1, CCDC126, FAM221A, STK31, NPY, MPP6, DFNA5, OSBPL3, CYCS, C7orf31, NPVF, MIR148A, NFE2L3, HNRNPA2B1, CBX3, SNX10, LOC105375304, LOC441204, KIAA0087, C7orf71, SKAP2, HOXA1, HOTAIRM1, HOXA2, HOXA3, HOXA-AS2, HOXA4, HOXA-AS3, HOXA5, HOXA6, HOXA7, HOXA9, HOXA10-HOXA9, HOXA10-AS, MIR196B, HOXA10, HOXA11, HOXA11-AS, HOXA13, HOTTIP, EVX1-AS, EVX1, HIBADH, TSL, TAX1BP1, JAZF1, JAZF1-AS1, CREB5, TRIL, LOC100506497, CPVL, LOC101928168, CHN2, LOC102724484, PRR15, LOC646762, MIR550A3, ZNRF2P2, DPY19L2P3, WIPF3, SCRN1, FKBP14, PLEKHA8, MTURN, LOC105375218, ZNRF2, MIR550B1, MIR550A1, DKFZP586I1420, LINC01176, NOD1, GGCT, LOC401320, GARS, CRHR2, INMT, INMT-FAM188B, FAM188B, AQP1, GHRHR, ADCYAP1R1, NEUROD6, CCDC129, PPP1R17, PDE1C, LOC100130673, LSM5, AVL9, DPY19L1P1, ZNRF2P1, MIR550B2, MIR550A2, LINC00997, DPY19L1P2, KBTBD2, RP9P, FKBP9, NT5C3A, RP9, BBS9, BMPER, NPSR1-AS1, NPSR1, DPY19L1, DPY19L2P1, TBX20, LOC401324, HERPUD2, LOC101930085, LOC100506725, SEPT7-AS1, SEPT7, LOC101928618, EEPD1, KIAA0895, ANLN, AOAH, AOAH-IT1, ELMO1, MIR1200, ELMO1-AS1, GPR141, NME8, SFRP4, EPDR1, STARD3NL, TARP, TRG-AS1, AMPH, FAM183BP, VPS41, POU6F2, POU6F2-AS1, YAE1D1, RALA, LINC00265, CDK13, MPLKIP, SUGCT, LINC01450, LINC01449, INHBA, INHBA-AS1, GLI3, LINC01448, C7orf25, PSMA2, MRPL32, HECW1, HECW1-IT1, MIR3943, LOC100506895, STK17A, COA1, BLVRA, MRPS24, URGCP-MRPS24, URGCP, UBE2D4, POLR2J4, SPDYE1, RASA4CP, LINC00957, DBNL, MIR6837, PGAM2, POLM, MIR6838, AEBP1, MIR4649, POLD2, MYL7, GCK, YKT6, CAMK2B, NUDCD3, NPC1L1, DDX56, TMED4, OGDH, ZMIZ2, PPIA, H2AFV, PURB, MIR4657, MYO1G, SNHG15, SNORA9, CCM2, NACAD, TBRG4, SNORA5A, SNORA5C, SNORA5B, RAMP3, ADCY1, SEPT7P2, IGFBP1, IGFBP3, LOC730338, TNS3, LINC01447, C7orf65, LINC00525, PKD1L1, C7orf69, HUS1, SUN3, C7orf57, UPP1, ABCA13, CDC14C, VWC2, ZPBP, C7orf72, IKZF1, FIGNL1, DDC, DDC-AS1, GRB10, COBL, POM121L12, LINC01446, HPVC1, LINC01445, VSTM2A, VSTM2A-OT1, SEC61G, LOC100996654, EGFR, EGFR-AS1, ELDR, LANCL2, VOPP1, FKBP9P1, SEPT14, ZNF713, MRPS17, GBAS, PSPH, CCT6A, SNORA15, SUMF2, PHKG1, CHCHD2, NUPR2, LOC650226, LOC100240728, DKFZp434L192, LOC101928401, LOC401357, LOC100130849, MIR4283-1, MIR4283-2, ZNF479, GUSBP10, LOC105375297, MIR3147, ZNF716, ZNF733P, LOC102724738, LOC100287704, LOC100287834, LINC01005, ZNF727, ZNF735, ZNF679, ZNF736, YWHAEP1, ZNF680, LOC100128885, LOC641746, ZNF107, MIR6839, ZNF138, ZNF273, ZNF117, ERV3-1, CCT6P3, ZNF92, LOC441242, INTS4P2, CCT6P1, SNORA22, VKORC1L1, GUSB, ASL, CRCP, TPST1, LINC00174, GS1-124K5.4, GS1-124K5.11, KCTD7, LOC100996437, RABGEF1, GTF2IRD1P1, GTF2IP23, LOC644794, TMEM248, SBDS, TYW1, MIR4650-2, MIR4650-1, PMS2P4, STAG3L4, LINC01372, LOC102723427, LOC100507468, AUTS2, WBSCR17, MIR3914-1, MIR3914-2, CALN1, TYW1B, SBDSP1, SPDYE7P, POM121, NSUN5P2, TRIM74, LOC541473, LOC100101148, STAG3L1, STAG3L3, PMS2P7, PMS2P5, PMS2P2, SPDYE8P, GTF2IP4, GTF2IP1, NCF1B, NSUN5, TRIM50, FKBP6, FZD9, BAZ1B, BCL7B, TBL2, MLXIPL, VPS37D, DNAJC30, WBSCR22, STX1A, MIR4284, ABHD11-AS1, ABHD11, CLDN3, CLDN4, WBSCR27, WBSCR28, ELN, LIMK1, EIF4H, MIR590, LAT2, RFC2, CLIP2, GTF2IRD1, GTF2I, LOC101926943, NCF1, GTF2IRD2, STAG3L2, RCC1L, GTF2IRD2B, NCF1C, GATSL2, TRIM73, NSUN5P1, POM121C, SPDYE5, PMS2P3, HIP1, CCL26, CCL24, RHBDD2, POR, MIR4651, SNORA14A, TMEM120A, STYXL1, MDH2, GTF2IP7, SRRM3, HSPB1, YWHAG, SSC4D, ZP3, DTX2, FDPSP2, UPK3B, LOC100133091, POMZP3, DTX2P1-UPK3BP1-PMS2P11, PMS2P9, CCDC146, FGL2, GSAP, LOC101927243, PTPN12, APTR, RSBN1L, TMEM60, PHTF2, MAGI2, RPL13AP17, MAGI2-AS2, MAGI2-AS3, GNAI1, LOC101927269, GNAT3, CD36, SEMA3C, LOC105369146, LOC100128317, HGF, CACNA2D1, LOC101927356, PCLO, SEMA3E, SEMA3A, LOC101927378, SEMA3D, LINC00972, GRM3, KIAA1324L, LOC101927420, DMTF1, TMEM243, TP53TG1, CROT, ABCB4, ABCB1, RUNDC3B, SLC25A40, DBF4, ADAM22, SRI, LOC102723885, STEAP4, ZNF804B, C7orf62, STEAP2-AS1, DPY19L2P4, STEAP1, STEAP2, CFAP69, LOC101927446, GTPBP10, LOC101409256, CLDN12, CDK14, FZD1, MTERF1, AKAP9, CYP51A1, CYP51A1-AS1, LRRD1, KRIT1, ANKIB1, LOC105375396, GATAD1, PEX1, RBM48, FAM133B, FAM133DP, CDK6, LOC101927497, SAMD9, SAMD9L, HEPACAM2, VPS50, CALCR, MIR653, MIR489, MIR4652, TFPI2, LOC105375401, GNGT1, GNG11, BET1, COL1A2, CASD1, SGCE, PEG10, PPP1R9A, PON1, PON3, PON2, ASB4, PDK4, DYNC1I1, SLC25A13, MIR591, C7orf76, LOC100506136, SHFM1, DLX6-AS1, DLX6, DLX5, SDHAF3, TAC1, ASNS, MIR5692A1, MIR5692A2, MIR5692C2, MGC72080, OCM2, LMTK2, BHLHA15, TECPR1, BRI3, BAIAP2L1, NPTX2, TMEM130, TRRAP, MIR3609, SCARNA28, LOC101927550, SMURF1, KPNA7, MYH16, ARPC1A, ARPC1B, PDAP1, BUD31, PTCD1, ATP5J2-PTCD1, CPSF4, ATP5J2, ZNF789, ZNF394, ZKSCAN5, FAM200A, ZNF655, GS1-259H13.2, ZSCAN25, CYP3A5, CYP3A7-CYP3A51P, CYP3A7, CYP3A4, CYP3A43, OR2AE1, TRIM4, GJC3, AZGP1, AZGP1P1, ZKSCAN1, ZSCAN21, ZNF3, COPS6, MCM7, MIR25, MIR93, MIR106B, AP4M1, TAF6, CNPY4, MBLAC1, LAMTOR4, C7orf43, MIR4658, GAL3ST4, GPC2, STAG3, GATS, PVRIG, SPDYE3, PMS2P1, STAG3L5P-PVRIG2P-PILRB, STAG3L5P, PVRIG2P, MIR6840, PILRB, PILRA, ZCWPW1, MEPCE, PPP1R35, C7orf61, TSC22D4, NYAP1, AGFG2, SAP25, LRCH4, ZASP, FBXO24, PCOLCE-AS1, PCOLCE, MOSPD3, TFR2, ACTL6B, LOC105375429, GNB2, GIGYF1, POP7, EPO, ZAN, EPHB4, SLC12A9, TRIP6, MIR6875, SRRT, UFSP1, ACHE, MUC3A, MUC12, LOC102724094, MUC17, TRIM56, SERPINE1, AP1S1, MIR4653, VGF, NAT16, MOGAT3, PLOD3, ZNHIT1, CLDN15, FIS1, LOC101927746, IFT22, COL26A1, LINC01007, MYL10, CUX1, SH2B2, MIR4285, SPDYE6, LOC100289561, LOC100630923, PRKRIP1, MIR548O, ORAI2, ALKBH4, LRWD1, MIR5090, MIR4467, POLR2J, RASA4B, POLR2J3, SPDYE2B, SPDYE2, RASA4, POLR2J2, UPK3BL, FAM185A, FBXL13, LRRC17, ARMC10, NAPEPLD, RPL19P12, DPY19L2P2, PMPCB, DNAJC2, PSMC2, SLC26A5, LOC101927870, RELN, ORC5, LHFPL3, LHFPL3-AS1, LHFPL3-AS2, LINC01004, KMT2E-AS1, KMT2E, SRPK2, PUS7, RINT1, EFCAB10, ATXN7L1, CDHR3, SYPL1, NAMPT, CCDC71L, PIK3CG, PRKAR2B, HBP1, COG5, GPR22, DUS4L, BCAP29, SLC26A4-AS1, SLC26A4, CBLL1, SLC26A3, DLD, LAMB1, LAMB4, NRCAM, PNPLA8, THAP5, DNAJB9, C7orf66, EIF3IP1, IMMP2L, LRRN3, DOCK4, DOCK4-AS1, ZNF277, IFRD1, LSMEM1, LOC100996249, LOC101928012, TMEM168, BMT2, HRAT17, GPR85, LINC00998, PPP1R3A, FOXP2, MIR3666, MDFIC, LINC01393, LINC01392, TFEC, TES, LOC102724434, CAV2, CAV1, LINC01510, MET, CAPZA2, ST7-AS1, ST7, ST7-OT4, MIR6132, ST7-AS2, ST7-OT3, WNT2, ASZ1, CFTR, CTTNBP2, LSM8, ANKRD7, LVCAT5, KCND2, TSPAN12, ING3, CPED1, WNT16, FAM3C, PTPRZ1, AASS, FEZF1, FEZF1-AS1, CADPS2, RNF133, RNF148, TAS2R16, SLC13A1, IQUB, NDUFA5, ASB15, LOC102724555, LMOD2, WASL, RNU6-2, HYALP1, HYAL4, SPAM1, LOC105375483, TMEM229A, LOC101928211, GPR37, C7orf77, POT1, POT1-AS1, LOC101928283, LOC101928254, GRM8, MIR592, LOC101928333, ZNF800, LOC100506682, GCC1, ARF5, FSCN3, PAX4, SND1, SND1-IT1, LRRC4, MIR593, MIR129-1, LEP, MGC27345, RBM28, PRRT4, IMPDH1, HILPDA, METTL2B, LINC01000, FAM71F2, FAM71F1, CALU, OPN1SW, CCDC136, FLNC, ATP6V1F, LOC100130705, KCP, IRF5, TNPO3, TPI1P2, LOC407835, TSPAN33, SMO, AHCYL2, STRIP2, SMKR1, NRF1, MIR182, MIR96, MIR183, UBE2H, ZC3HC1, KLHDC10, TMEM209, SSMEM1, CPA2, CPA4, CPA5, LOC105375504, CPA1, CEP41, MEST, MESTIT1, MIR335, COPG2, TSGA13, KLF14, MIR29A, MIR29B1, LINC-PINT, LOC100506860, MKLN1, MKLN1-AS, PODXL, LOC101928782, PLXNA4, LOC101928807, FLJ40288, LOC100506937, CHCHD3, MIR3654, LOC105375512, EXOC4, MIR6133, LOC101928861, LRGUK, SLC35B4, AKR1B1, AKR1B10, AKR1B15, BPGM, CALD1, AGBL3, C7orf49, TMEM140, WDR91, MIR6509, STRA8, CNOT4, NUP205, C7orf73, SLC13A4, FAM180A, MTPN, LUZP6, CHRM2, LOC349160, MIR490, PTN, DGKI, CREB3L2, LOC100130880, AKR1D1, MIR4468, TRIM24, SVOPL, ATP6V0A4, TMEM213, KIAA1549, ZC3HAV1L, ZC3HAV1, TTC26, UBN2, LUC7L2, FMC1, C7orf55-LUC7L2, LOC100129148, KLRG2, CLEC2L, HIPK2, TBXAS1, PARP12, KDM7A, JHDM1D-AS1, SLC37A3, RAB19, MKRN1, DENND2A, ADCK2, NDUFB2-AS1, NDUFB2, BRAF, MRPS33, TMEM178B, AGK, KIAA1147, WEE2-AS1, WEE2, SSBP1, TAS2R3, TAS2R4, TAS2R5, PRSS37, OR9A4, CLEC5A, TAS2R38, MGAM, MGAM2, MOXD2P, PRSS58, TRY2P, MTRNR2L6, PRSS1, PRSS3P2, EPHB6, TRPV6, TRPV5, C7orf34, KEL, OR9A2, OR6V1, OR6W1P, PIP, TAS2R39, TAS2R40, LOC105375545, GSTK1, TMEM139, CASP2, CLCN1, FAM131B, LOC100507507, ZYX, MIR6892, EPHA1, EPHA1-AS1, TAS2R60, TAS2R41, CTAGE15, TCAF2, TCAF2P1, CTAGE6, LOC154761, TCAF1, OR2F2, OR2F1, OR6B1, OR2A5, OR2A25, OR2A12, OR2A2, OR2A14, CTAGE4, ARHGEF35, LOC101928605, OR2A1-AS1, OR2A1, OR2A42, OR2A9P, OR2A20P, OR2A7, ARHGEF34P, CTAGE8, ARHGEF5, NOBOX, TPK1, CNTNAP2, LOC101928700, MIR548F4, LOC105375556, MIR548T, C7orf33, CUL1, EZH2, GHET1, PDIA4, ZNF786, ZNF425, ZNF398, ZNF282, ZNF212, ZNF783, LOC155060, ZNF777, ZNF746, ZNF767P, KRBA1, ZNF467, SSPO, ZNF862, ATP6V0E2-AS1, ATP6V0E2, ACTR3C, LRRC61, ZBED6CL, RARRES2, REPIN1, ZNF775, LOC728743, LINC00996, GIMAP8, GIMAP7, GIMAP4, GIMAP6, GIMAP2, GIMAP1, GIMAP1-GIMAP5, GIMAP5, TMEM176B, TMEM176A, AOC1, KCNH2, NOS3, ATG9B, ABCB8, ASIC3, CDK5, SLC4A2, FASTK, TMUB1, AGAP3, GBX1, ASB10, IQCA1L, ABCF2, CHPF2, MIR671, SMARCD3, NUB1, WDR86, WDR86-AS1, CRYGN, MIR3907, RHEB, PRKAG2, PRKAG2-AS1, GALNTL5, GALNT11, KMT2C, FABP5P3, LINC01003, XRCC2, ACTR3B, LINC01287, DPP6, LOC101929998, PAXIP1-AS2, PAXIP1, PAXIP1-AS1, HTR5A-AS1, HTR5A, INSIG1, BLACE, EN2, CNPY1, LOC100506302, RBM33, SHH, LOC389602, LOC285889, LINC01006, LINC00244, C7orf13, RNF32, LMBR1, NOM1, MNX1, MNX1-AS1, UBE3C, DNAJB6, LOC101927914, PTPRN2, MIR153-2, LOC100506585, MIR595, LINC01022, MIR5707, NCAPG2, ESYT2, WDR60, LINC00689, VIPR2 | arr[GRCh37] 7p22.3q36.3(41420_159118443)x3 |
| 2  LM | Gain | 8 | p11.22 | q24.3 | 106,653 | ADAM2, IDO1, IDO2, C8orf4, ZMAT4, SFRP1, MIR548AO, GOLGA7, GINS4, LOC102723729, GPAT4, NKX6-3, ANK1, MIR486-1, MIR486-2, KAT6A, LOC105379393, AP3M2, PLAT, LOC101929897, IKBKB, POLB, DKK4, VDAC3, SLC20A2, SMIM19, CHRNB3, CHRNA6, THAP1, RNF170, MIR4469, HOOK3, FNTA, POMK, HGSNAT, POTEA, LINC00293, LOC100287846, SPIDR, CEBPD, PRKDC, MCM4, UBE2V2, LOC101929268, LOC101929217, EFCAB1, SNAI2, C8orf22, LOC100507464, SNTG1, PXDNL, PCMTD1, ST18, LOC101929341, FAM150A, RB1CC1, NPBWR1, OPRK1, ATP6V1H, RGS20, TCEA1, LYPLA1, MRPL15, SOX17, RP1, XKR4, SBF1P1, LOC105375843, TMEM68, TGS1, LYN, RPS20, SNORD54, MOS, PLAG1, CHCHD7, SDR16C5, SDR16C6P, PENK, LOC101929415, LINC00968, IMPAD1, LINC01606, LOC286177, LINC00588, LOC101929488, LOC286178, LINC01602, FAM110B, LOC101929528, UBXN2B, CYP7A1, SDCBP, NSMAF, TOX, CA8, LINC01301, RAB2A, CHD7, LOC100130298, CLVS1, ASPH, MIR4470, NKAIN3, UG0898H09, GGH, TTPA, YTHDF3-AS1, YTHDF3, LOC102724612, LINC01289, LOC102724623, MIR124-2HG, MIR124-2, LOC401463, BHLHE22, CYP7B1, LINC00251, LINC01299, ARMC1, MTFR1, PDE7A, DNAJC5B, TRIM55, CRH, LINC00967, RRS1-AS1, RRS1, ADHFE1, C8orf46, MYBL1, VCPIP1, C8orf44, C8orf44-SGK3, SGK3, PTTG3P, MCMDC2, SNHG6, SNORD87, TCF24, PPP1R42, COPS5, CSPP1, ARFGEF1, LOC102724708, CPA6, PREX2, C8orf34-AS1, C8orf34, LINC01592, LINC01603, SULF1, SLCO5A1, PRDM14, NCOA2, LOC101926892, TRAM1, LACTB2-AS1, LACTB2, XKR9, EYA1, MSC, MSC-AS1, TRPA1, LOC392232, KCNB2, LOC101926908, TERF1, SBSPON, C8orf89, RPL7, RDH10, RDH10-AS1, STAU2-AS1, STAU2, UBE2W, TCEB1, TMEM70, LY96, JPH1, GDAP1, MIR5681A, MIR5681B, MIR2052HG, MIR2052, PI15, CRISPLD1, CASC9, HNF4G, LINC01111, ZFHX4-AS1, ZFHX4, MIR3149, PEX2, LOC102724874, PKIA, PKIA-AS1, ZC2HC1A, LOC101241902, IL7, STMN2, HEY1, LINC01607, LOC101927040, MRPS28, TPD52, MIR5708, ZBTB10, ZNF704, PAG1, FABP5, PMP2, FABP9, FABP4, FABP12, IMPA1, SLC10A5, ZFAND1, CHMP4C, SNX16, LOC101927141, LINC01419, RALYL, LRRCC1, LOC102723322, E2F5, C8orf59, CA13, CA1, CA3, CA3-AS1, CA2, REXO1L2P, PSKH2, ATP6V0D2, SLC7A13, WWP1, RMDN1, CPNE3, CNGB3, CNBD1, DCAF4L2, MMP16, LOC101929709, RIPK2, OSGIN2, NBN, DECR1, CALB1, LINC00534, LINC01030, TMEM64, NECAB1, C8orf88, TMEM55A, OTUD6B-AS1, OTUD6B, LRRC69, MIR4661, SLC26A7, RUNX1T1, MIR7641-2, LOC102724710, FLJ46284, TRIQK, MIR8084, C8orf87, LINC00535, FAM92A1, RBM12B, RBM12B-AS1, TMEM67, MIR378D2, PDP1, CDH17, GEM, RAD54B, FSBP, KIAA1429, LOC100288748, ESRP1, DPY19L4, INTS8, CCNE2, TP53INP1, NDUFAF6, LOC105375650, MIR3150B, MIR3150A, PLEKHF2, LINC01298, C8orf37, C8orf37-AS1, LOC100500773, GDF6, UQCRB, MTERF3, PTDSS1, LOC102724804, SDC2, CPQ, LOC101927066, TSPYL5, MTDH, LAPTM4B, MATN2, RPL30, SNORA72, ERICH5, RIDA, POP1, NIPAL2, KCNS2, STK3, OSR2, VPS13B, MIR599, MIR875, COX6C, RGS22, MIR1273A, FBXO43, POLR2K, SPAG1, RNF19A, MIR4471, ANKRD46, SNX31, PABPC1, MIR7705, YWHAZ, FLJ42969, ZNF706, NACAP1, GRHL2, NCALD, LOC104054148, MIR5680, RRM2B, UBR5-AS1, UBR5, ODF1, KLF10, AZIN1, AZIN1-AS1, ATP6V1C1, BAALC-AS2, BAALC, MIR3151, BAALC-AS1, FZD6, CTHRC1, SLC25A32, DCAF13, RIMS2, DCSTAMP, DPYS, LRP12, ZFPM2, ZFPM2-AS1, OXR1, ABRA, ANGPT1, RSPO2, EIF3E, EMC2, TMEM74, TRHR, NUDCD1, ENY2, PKHD1L1, EBAG9, SYBU, LOC100132813, KCNV1, LINC01608, LINC01609, CSMD3, MIR2053, TRPS1, LINC00536, EIF3H, LOC105375713, UTP23, RAD21, RAD21-AS1, MIR3610, AARD, SLC30A8, MED30, EXT1, SAMD12, SAMD12-AS1, TNFRSF11B, COLEC10, LOC101927513, MAL2, MIR548AZ, NOV, ENPP2, TAF2, DSCC1, DEPTOR, COL14A1, MRPL13, MTBP, SNTB1, LOC101927543, HAS2, HAS2-AS1, LOC105375734, LINC01151, ZHX2, DERL1, TBC1D31, FAM83A, FAM83A-AS1, MIR4663, C8orf76, ZHX1-C8orf76, ZHX1, ATAD2, MIR548D1, WDYHV1, FBXO32, KLHL38, ANXA13, FAM91A1, FER1L6, FER1L6-AS1, FER1L6-AS2, LOC101927588, TMEM65, TRMT12, RNF139-AS1, RNF139, TATDN1, MIR6844, NDUFB9, MTSS1, MIR4662B, MIR4662A, LINC00964, ZNF572, LOC105375744, SQLE, KIAA0196, NSMCE2, TRIB1, LINC00861, LOC101927657, FAM84B, PCAT1, PCAT2, PRNCR1, CASC19, CCAT1, CASC21, CASC8, CCAT2, POU5F1B, CASC11, MYC, PVT1, MIR1204, TMEM75, MIR1205, MIR1206, MIR1207, MIR1208, LINC00824, LINC00977, CCDC26, MIR3686, GSDMC, FAM49B, MIR5194, ASAP1, ASAP1-IT2, ASAP1-IT1, ADCY8, EFR3A, OC90, HHLA1, KCNQ3, HPYR1, LRRC6, TMEM71, PHF20L1, TG, SLA, MIR7848, WISP1, NDRG1, ST3GAL1, LOC105375773, LOC101927798, LOC101927822, ZFAT, ZFAT-AS1, MIR30B, MIR30D, NCRNA00250, LOC101927845, LINC01591, KHDRBS3, LOC101927915, FAM135B, COL22A1, KCNK9, TRAPPC9, CHRAC1, AGO2, PTK2, DENND3, SLC45A4, LOC105375787, LINC01300, GPR20, PTP4A3, MROH5, MIR1302-7, MIR4539, MIR4472-1, LINC00051, TSNARE1, ADGRB1, ARC, LOC101928087, JRK, PSCA, LY6K, LOC100288181, THEM6, SLURP1, LYPD2, LYNX1, LY6D, GML, CYP11B1, CYP11B2, LOC100133669, CDC42P3, LY6E, C8orf31, LY6H, GPIHBP1, ZFP41, GLI4, MINCR, ZNF696, TOP1MT, RHPN1-AS1, RHPN1, MAFA-AS1, MAFA, ZC3H3, GSDMD, MROH6, NAPRT, EEF1D, TIGD5, PYCRL, TSTA3, ZNF623, ZNF707, BREA2, CCDC166, LOC101928160, MAPK15, FAM83H, MIR4664, FAM83H-AS1, LOC105375800, SCRIB, MIR937, PUF60, NRBP2, MIR6845, EPPK1, PLEC, MIR661, PARP10, GRINA, SPATC1, OPLAH, MIR6846, EXOSC4, MIR6847, GPAA1, CYC1, SHARPIN, MAF1, WDR97, HGH1, MROH1, MIR7112, SCX, BOP1, HSF1, DGAT1, MIR6848, SCRT1, TMEM249, FBXL6, SLC52A2, LOC101928902, ADCK5, CPSF1, MIR939, MIR6849, SLC39A4, VPS28, TONSL, TONSL-AS1, MIR6893, CYHR1, KIFC2, FOXH1, PPP1R16A, GPT, MFSD3, RECQL4, LRRC14, LRRC24, C8orf82, ARHGAP39, ZNF251, ZNF34, RPL8, MIR6850, ZNF517, ZNF7, COMMD5, ZNF250, ZNF16, ZNF252P, TMED10P1, ZNF252P-AS1, C8orf33 | arr[GRCh37] 8p11.22q24.3(39639410_146292734)x3 |
| 2  LM | Gain | 12 | q11 | q24.33 | 95,915 | ALG10B, CPNE8, KIF21A, ABCD2, C12orf40, SLC2A13, LRRK2, MUC19, CNTN1, PDZRN4, LOC101927038, GXYLT1, YAF2, ZCRB1, MIR7851, PPHLN1, PRICKLE1, LOC101927058, LOC105369738, LOC105369739, ADAMTS20, PUS7L, IRAK4, TWF1, TMEM117, NELL2, DBX2, RACGAP1P, PLEKHA8P1, RNY5, ANO6, LINC00938, ARID2, SCAF11, SLC38A1, SLC38A2, LOC100288798, SLC38A4, AMIGO2, PCED1B, MIR4698, PCED1B-AS1, LOC105369747, MIR4494, RPAP3, ENDOU, RAPGEF3, SLC48A1, HDAC7, VDR, TMEM106C, COL2A1, SENP1, PFKM, MIR6505, ASB8, CCDC184, OR10AD1, H1FNT, ZNF641, ANP32D, C12orf54, OR8S1, LALBA, KANSL2, SNORA2C, MIR1291, SNORA2A, SNORA2B, CCNT1, LINC00935, ADCY6, MIR4701, LOC100506125, CACNB3, DDX23, RND1, CCDC65, FKBP11, ARF3, WNT10B, WNT1, DDN, PRKAG1, KMT2D, RHEBL1, DHH, LMBR1L, TUBA1B, TUBA1A, TUBA1C, LOC101927267, PRPH, TROAP, C1QL4, DNAJC22, SPATS2, LOC100335030, KCNH3, MCRS1, FAM186B, PRPF40B, FMNL3, TMBIM6, NCKAP5L, BCDIN3D-AS1, BCDIN3D, FAIM2, LOC283332, LOC101927292, AQP2, LOC101927318, AQP5, AQP6, RACGAP1, ASIC1, SMARCD1, GPD1, COX14, CERS5, LIMA1, MIR1293, FAM186A, LARP4, SNORD133, DIP2B, ATF1, TMPRSS12, METTL7A, HIGD1C, SLC11A2, LETMD1, CSRNP2, TFCP2, POU6F1, DAZAP2, SMAGP, BIN2, CELA1, GALNT6, SLC4A8, SCN8A, FIGNL2, LOC105369971, ANKRD33, ACVRL1, ACVR1B, GRASP, NR4A1, ATG101, OR7E47P, KRT80, C12orf80, LINC00592, KRT7, KRT86, KRT81, KRT83, KRT85, KRT84, KRT82, KRT75, KRT6B, KRT6C, KRT6A, KRT5, KRT71, KRT74, KRT72, KRT73, KRT73-AS1, KRT2, KRT1, KRT77, KRT76, KRT3, KRT4, KRT79, KRT78, KRT8, KRT18, EIF4B, LOC283335, TNS2, MIR6757, SPRYD3, IGFBP6, SOAT2, CSAD, ZNF740, ITGB7, RARG, MFSD5, ESPL1, PFDN5, C12orf10, AAAS, SP7, SP1, AMHR2, PRR13, PCBP2, PCBP2-OT1, MAP3K12, TARBP2, NPFF, ATF7, LOC100652999, ATP5G2, CALCOCO1, CISTR, HOXC13-AS, HOXC13, HOXC12, HOTAIR, HOXC11, HOXC-AS3, HOXC10, MIR196A2, HOXC-AS2, HOXC-AS1, HOXC9, HOXC8, HOXC6, HOXC5, HOXC4, MIR615, FLJ12825, LOC100240735, LOC100240734, LOC400043, SMUG1, CBX5, MIR3198-2, HNRNPA1, HNRNPA1P10, NFE2, COPZ1, MIR148B, LOC102724050, GPR84, ZNF385A, ITGA5, GTSF1, NCKAP1L, PDE1B, PPP1R1A, GLYCAM1, LACRT, DCD, MUCL1, TESPA1, NEUROD4, OR9K2, OR10A7, OR6C74, OR6C6, OR6C1, OR6C3, OR6C75, OR6C65, OR6C76, OR6C2, OR6C70, OR6C68, OR6C4, OR2AP1, OR10P1, METTL7B, ITGA7, BLOC1S1, BLOC1S1-RDH5, RDH5, CD63, GDF11, SARNP, ORMDL2, DNAJC14, TMEM198B, MMP19, PYM1, DGKA, PMEL, CDK2, RAB5B, SUOX, LOC105369781, IKZF4, RPS26, ERBB3, PA2G4, RPL41, ZC3H10, ESYT1, MYL6B, MYL6, SMARCC2, RNF41, NABP2, SLC39A5, ANKRD52, COQ10A, CS, CNPY2, PAN2, IL23A, STAT2, APOF, TIMELESS, MIP, SPRYD4, GLS2, SNORA105C, RBMS2, BAZ2A, ATP5B, SNORD59B, SNORD59A, PTGES3, NACA, PRIM1, HSD17B6, SDR9C7, RDH16, GPR182, ZBTB39, TAC3, MYO1A, NEMP1, NAB2, STAT6, LRP1, LRP1-AS, MIR1228, NXPH4, SHMT2, NDUFA4L2, STAC3, R3HDM2, INHBC, INHBE, GLI1, ARHGAP9, MARS, MIR6758, DDIT3, MIR616, MBD6, DCTN2, KIF5A, PIP4K2C, DTX3, ARHGEF25, LOC101927583, SLC26A10, B4GALNT1, OS9, AGAP2, AGAP2-AS1, TSPAN31, CDK4, MIR6759, MARCH9, CYP27B1, METTL1, METTL21B, TSFM, AVIL, CTDSP2, MIR26A2, LOC100506844, ATP23, LOC105369785, LOC101927653, LOC100506869, LRIG3, SLC16A7, FAM19A2, USP15, MIR6125, MON2, LINC01465, MIRLET7I, PPM1H, AVPR1A, DPY19L2, TMEM5, TMEM5-AS1, SRGAP1, C12orf66, C12orf56, XPOT, TBK1, RASSF3, MIR548Z, MIR548C, GNS, TBC1D30, FLJ41278, WIF1, LEMD3, MSRB3, LOC100507065, LOC105369187, RPSAP52, HMGA2, LOC100129940, MIR6074, LLPH, LLPH-AS1, TMBIM4, IRAK3, MIR6502, HELB, GRIP1, LOC102724421, CAND1, LOC100507175, DYRK2, LOC101927901, LINC01479, IFNG-AS1, IFNG, IL26, IL22, MDM1, LOC100507195, RAP1B, SNORA70G, LOC100507250, NUP107, SLC35E3, LOC100130075, MDM2, CPM, CPSF6, MIR1279, LYZ, YEATS4, FRS2, MIR3913-1, MIR3913-2, CCT2, LRRC10, BEST3, LOC101928002, RAB3IP, MYRFL, LINC01481, CNOT2, KCNMB4, PTPRB, PTPRR, TSPAN8, LGR5, ZFC3H1, THAP2, TMEM19, RAB21, TBC1D15, MRS2P2, TPH2, TRHDE-AS1, TRHDE, LOC101928137, LOC100507377, ATXN7L3B, KCNC2, CAPS2, GLIPR1L1, GLIPR1L2, GLIPR1, KRR1, PHLDA1, NAP1L1, BBS10, OSBPL8, ZDHHC17, CSRP2, E2F7, NAV3, LOC105369860, SYT1, MIR1252, PAWR, PPP1R12A, OTOGL, PTPRQ, MYF6, MYF5, LINC01490, LIN7A, MIR617, MIR618, ACSS3, MIR4699, PPFIA2, LOC102724663, LOC101928449, CCDC59, METTL25, TMTC2, SLC6A15, TSPAN19, LRRIQ1, ALX1, RASSF9, NTS, MGAT4C, LOC105369879, MKRN9P, C12orf50, C12orf29, CEP290, TMTC3, KITLG, LOC728084, DUSP6, POC1B, GALNT4, POC1B-GALNT4, ATP2B1, LINC00936, LOC105369891, LOC105369893, LINC00615, CCER1, EPYC, KERA, LUM, DCN, LINC01619, BTG1, LOC101928617, CLLU1OS, CLLU1, C12orf74, PLEKHG7, EEA1, LOC643339, LOC102724933, NUDT4, NUDT4P2, NUDT4P1, UBE2N, MRPL42, SOCS2-AS1, SOCS2, CRADD, LOC101928731, LOC105369911, PLXNC1, CEP83, CEP83-AS1, MIR5700, TMCC3, MIR7844, MIR492, KRT19P2, NDUFA12, NR2C1, FGD6, VEZT, MIR331, MIR3685, METAP2, USP44, PGAM1P5, NTN4, LOC105369921, LOC105369920, SNRPF, CCDC38, AMDHD1, HAL, LTA4H, ELK3, CDK17, CFAP54, NEDD1, RMST, MIR1251, MIR135A2, LOC643711, MIR4495, MIR4303, SLC9A7P1, LOC643770, TMPO-AS1, TMPO, SLC25A3, SNORA53, IKBIP, APAF1, ANKS1B, LOC101928937, FAM71C, UHRF1BP1L, GOLGA2P5, MIR1827, ACTR6, DEPDC4, SCYL2, SLC17A8, NR1H4, GAS2L3, ANO4, SLC5A8, UTP20, ARL1, SPIC, MYBPC1, CHPT1, SYCP3, GNPTAB, DRAM1, CCDC53, NUP37, PARPBP, PMCH, IGF1, LINC00485, PAH, ASCL1, LOC101929058, C12orf42, LOC105369945, LOC101929084, STAB2, NT5DC3, TTC41P, HSP90B1, MIR3652, C12orf73, TDG, GLT8D2, HCFC2, NFYB, MIR7641-2, TXNRD1, EID3, CHST11, MIR3922, SLC41A2, C12orf45, ALDH1L2, LOC414300, KIAA1033, APPL2, KCCAT198, C12orf75, CASC18, NUAK1, CKAP4, TCP11L2, POLR3B, LOC100287944, RFX4, LOC100505978, RIC8B, TMEM263, MTERF2, CRY1, BTBD11, PWP1, PRDM4, LOC101929162, ASCL4, LOC728739, WSCD2, CMKLR1, LINC01498, FICD, SART3, ISCU, TMEM119, SELPLG, MIR4496, CORO1C, SSH1, MIR619, DAO, SVOP, USP30, USP30-AS1, ALKBH2, UNG, ACACB, FOXN4, LINC01486, MYO1H, KCTD10, UBE3B, MMAB, MVK, FAM222A, FAM222A-AS1, TRPV4, MIR4497, GLTP, TCHP, GIT2, ANKRD13A, C12orf76, IFT81, ATP2A2, ANAPC7, ARPC3, GPN3, FAM216A, VPS29, RAD9B, PPTC7, TCTN1, HVCN1, PPP1CC, CCDC63, MYL2, LINC01405, LOC105369980, CUX2, MIR6760, FAM109A, SH2B3, ATXN2, BRAP, ACAD10, ALDH2, MIR6761, MAPKAPK5-AS1, MAPKAPK5, ADAM1A, TMEM116, ERP29, NAA25, MIR3657, TRAFD1, HECTD4, MIR6861, RPL6, PTPN11, MIR1302-1, RPH3A, OAS1, OAS3, OAS2, DTX1, RASAL1, CFAP73, DDX54, MIR7106, RITA1, IQCD, TPCN1, MIR6762, SLC8B1, PLBD2, SDS, SDSL, LHX5, LHX5-AS1, LINC01234, RBM19, TBX5, TBX5-AS1, TBX3, MED13L, MIR620, MIR4472-2, LINC00173, MAP1LC3B2, C12orf49, RNFT2, HRK, FBXW8, LOC100506551, TESC, TESC-AS1, FBXO21, NOS1, KSR2, RFC5, WSB2, VSIG10, PEBP1, TAOK3, SUDS3, LOC105370014, LOC105370016, SRRM4, LOC105370024, HSPB8, LINC00934, CCDC60, TMEM233, PRKAB1, CIT, MIR1178, BICDL1, RAB35, GCN1, MIR4498, RPLP0, PXN-AS1, PXN, SIRT4, PLA2G1B, MSI1, COX6A1, TRIAP1, GATC, SRSF9, DYNLL1, NRAV, COQ5, RNF10, POP5, CABP1, MLEC, UNC119B, MIR4700, ACADS, SPPL3, XLOC_009911, HNF1A-AS1, HNF1A, C12orf43, OASL, P2RX7, P2RX4, CAMKK2, ANAPC5, RNF34, KDM2B, MIR7107, MIR548AQ, ORAI1, MORN3, TMEM120B, RHOF, LINC01089, SETD1B, HPD, PSMD9, WDR66, BCL7A, LOC100506691, MLXIP, LRRC43, IL31, B3GNT4, DIABLO, LOC101593348, VPS33A, CLIP1, CLIP1-AS1, ZCCHC8, RSRC2, KNTC1, HCAR2, HCAR3, HCAR1, DENR, CCDC62, HIP1R, VPS37B, ABCB9, OGFOD2, ARL6IP4, PITPNM2, MIR4304, LOC100507091, MPHOSPH9, C12orf65, CDK2AP1, SBNO1, MIR8072, KMT5A, RILPL2, SNRNP35, RILPL1, MIR3908, LOC101927415, TMED2, DDX55, EIF2B1, GTF2H3, TCTN2, ATP6V0A2, DNAH10, CCDC92, ZNF664, ZNF664-FAM101A, FAM101A, NCOR2, MIR6880, SCARB1, UBC, MIR5188, DHX37, BRI3BP, THRIL, AACS, TMEM132B, LINC00939, LOC101927464, LOC100128554, LOC100996671, LINC00944, LINC00943, LOC440117, LOC101927592, LOC101927616, LOC101927637, LOC105370068, FLJ37505, LINC00508, LINC00507, CRAT8, LOC100996679, LOC101927694, MIR4419B, TMEM132C, MIR3612, SLC15A4, GLT1D1, TMEM132D, LOC283352, LOC101927735, LOC100190940, FZD10-AS1, FZD10, PIWIL1, RIMBP2, STX2, RAN, ADGRD1, LACAT8, LINC01257, LOC107161159, LOC338797, SFSWAP, MMP17, ULK1, PUS1, EP400, SNORA49, EP400NL, DDX51, NOC4L, GALNT9, LOC100130238, LOC101928416, FBRSL1, MIR6763, LRCOL1, P2RX2, POLE, PXMP2, PGAM5, ANKLE2, GOLGA3, CHFR, LOC101928530, ZNF605, ZNF26, LOC101928597, ZNF84, ZNF140, ZNF891, ZNF10, ZNF268, ANHX | arr[GRCh37] 12q11q24.33(37902987_133818115)x3 |
| 2  LM | Gain | 13 | q12.13 | q12.3 | 5,928 | AMER2, LINC00463, LINC01053, MTMR6, NUP58, ATP8A2, SHISA2, RNF6, CDK8, WASF3, GPR12, USP12, USP12-AS1, USP12-AS2, LINC00412, RPL21, RPL21P28, SNORD102, SNORA27, RASL11A, GTF3A, MTIF3, LNX2, POLR1D, GSX1, PDX1-AS1, PDX1, ATP5EP2, LINC00543, CDX2, URAD, FLT3, PAN3-AS1, PAN3, FLT1, POMP, SLC46A3, MTUS2, MTUS2-AS1, SLC7A1, LOC102723345, UBL3, LINC00297, LINC00572, LINC00544, LINC00365, KATNAL1, LINC00426, LINC01058, HMGB1, USPL1, ALOX5AP, LINC00398, LINC00545, TEX26-AS1, MEDAG, TEX26 | arr[GRCh37] 13q12.13q12.3(25680689_31608475)x8 |
| 2  LM | Gain | 16 | p13.3 | q24.1 | 85,293 | POLR3K, SNRNP25, RHBDF1, MPG, NPRL3, HBZ, HBM, HBA2, HBA1, HBQ1, LUC7L, FAM234A, RGS11, ARHGDIG, PDIA2, AXIN1, MRPL28, TMEM8A, LOC100134368, NME4, DECR2, RAB11FIP3, LINC00235, CAPN15, MIR5587, MIR3176, PRR35, NHLRC4, PIGQ, RAB40C, WFIKKN1, METTL26, MCRIP2, LOC105371038, WDR90, RHOT2, RHBDL1, LOC105371184, STUB1, JMJD8, WDR24, FBXL16, METRN, FAM173A, CCDC78, HAGHL, NARFL, MSLN, MIR662, RPUSD1, CHTF18, GNG13, PRR25, LMF1, LMF1-AS1, SOX8, SSTR5-AS1, SSTR5, C1QTNF8, CACNA1H, TPSG1, TPSB2, TPSAB1, TPSD1, UBE2I, BAIAP3, TSR3, GNPTG, UNKL, C16orf91, CCDC154, CLCN7, PTX4, TELO2, IFT140, TMEM204, LOC105371046, CRAMP1, HN1L, MAPK8IP3, MIR3177, NME3, MRPS34, EME2, SPSB3, NUBP2, IGFALS, HAGH, FAHD1, MEIOB, LINC00254, HS3ST6, MSRB1, RPL3L, NDUFB10, RPS2, SNORA10, SNORA64, SNHG9, SNORA78, RNF151, TBL3, NOXO1, GFER, SYNGR3, ZNF598, NPW, SLC9A3R2, NTHL1, TSC2, PKD1, MIR1225, LOC105371049, MIR6511B1, MIR6511B2, MIR4516, MIR3180-5, RAB26, SNHG19, SNORD60, TRAF7, CASKIN1, MLST8, BRICD5, PGP, E4F1, DNASE1L2, ECI1, RNPS1, LOC106660606, MIR3677, MIR940, MIR4717, ABCA3, ABCA17P, CCNF, MIR6767, C16orf59, MIR6768, LOC729652, NTN3, TBC1D24, ATP6V0C, AMDHD2, CEMP1, MIR3178, PDPK1, LOC652276, FLJ42627, ERVK13-1, KCTD5, PRSS27, SRRM2-AS1, SRRM2, TCEB2, PRSS33, PRSS41, PRSS21, ZG16B, PRSS30P, PRSS22, FLYWCH2, FLYWCH1, KREMEN2, PAQR4, PKMYT1, LINC00514, LOC101929613, CLDN9, CLDN6, TNFRSF12A, HCFC1R1, THOC6, BICDL2, LOC100128770, MMP25, MMP25-AS1, IL32, ZSCAN10, ZNF205-AS1, ZNF205, ZNF213-AS1, ZNF213, CASP16P, OR1F1, OR1F2P, ZNF200, MEFV, LINC00921, ZNF263, TIGD7, ZNF75A, OR2C1, MTRNR2L4, ZSCAN32, ZNF174, ZNF597, NAA60, MIR6126, C16orf90, CLUAP1, NLRC3, SLX4, DNASE1, TRAP1, CREBBP, LOC102724927, ADCY9, SRL, LINC01569, TFAP4, GLIS2, GLIS2-AS1, PAM16, CORO7-PAM16, CORO7, VASN, DNAJA3, NMRAL1, HMOX2, CDIP1, C16orf96, UBALD1, MGRN1, MIR6769A, NUDT16L1, ANKS3, C16orf71, ZNF500, SEPT12, SMIM22, ROGDI, GLYR1, UBN1, PPL, SEC14L5, NAGPA, NAGPA-AS1, C16orf89, ALG1, EEF2KMT, LINC01570, MIR8065, RBFOX1, TMEM114, METTL22, ABAT, TMEM186, PMM2, CARHSP1, USP7, C16orf72, LOC101927009, LINC01177, LINC01195, GRIN2A, ATF7IP2, EMP2, TEKT5, NUBP1, TVP23A, CIITA, DEXI, CLEC16A, SOCS1, TNP2, PRM3, PRM2, PRM1, LOC105371083, RMI2, LOC101927131, LITAF, SNN, TXNDC11, ZC3H7A, BCAR4, RSL1D1, GSPT1, TNFRSF17, SNX29, CPPED1, MIR4718, SHISA9, ERCC4, LOC101927311, LOC101927348, MKL2, MIR193BHG, MIR193B, MIR365A, LOC105447648, PARN, BFAR, PLA2G10, NPIPA2, NPIPA3, ABCC6P2, NOMO1, MIR3179-2, MIR3179-3, MIR3179-1, MIR3179-4, MIR3670-2, MIR3670-4, MIR3670-1, MIR3670-3, MIR3180-3, MIR3180-1, MIR3180-2, LOC100288162, MIR6511A2, MIR6511A3, MIR6511A4, MIR6511A1, MIR6770-2, MIR6770-1, MIR6770-3, NPIPA1, PDXDC1, NTAN1, RRN3, LOC100505915, PKD1P6-NPIPP1, MIR3180-4, NPIPA5, MPV17L, C16orf45, KIAA0430, MIR6506, NDE1, MIR484, MYH11, FOPNL, ABCC1, ABCC6, NOMO3, PKD1P1, NPIPA7, NPIPA8, XYLT1, LOC102723692, NOMO2, ABCC6P1, RPS15A, ARL6IP1, SMG1, TMC7, LOC102723385, COQ7, ITPRIPL2, SYT17, CLEC19A, TMC5, GDE1, CCP110, C16orf62, KNOP1, IQCK, GPRC5B, GPR139, GP2, UMOD, PDILT, ACSM5, ACSM2A, ACSM2B, ACSM1, THUMPD1, ACSM3, ERI2, LOC81691, DCUN1D3, LYRM1, DNAH3, TMEM159, ZP2, ANKS4B, CRYM, CRYM-AS1, SNX29P1, NPIPB3, LOC100190986, SMG1P3, MIR3680-2, MIR3680-1, SLC7A5P2, LOC101927814, METTL9, IGSF6, OTOA, RRN3P1, NPIPB4, UQCRC2, PDZD9, C16orf52, VWA3A, EEF2K, POLR3E, CDR2, MFSD13B, RRN3P3, SMG1P1, NPIPB5, LOC653786, HS3ST2, USP31, SCNN1G, SCNN1B, COG7, GGA2, EARS2, UBFD1, NDUFAB1, PALB2, DCTN5, PLK1, ERN2, CHP2, PRKCB, MIR1273H, CACNG3, RBBP6, LINC01567, TNRC6A, SLC5A11, ARHGAP17, LOC554206, LOC283887, LCMT1-AS1, LCMT1, LCMT1-AS2, AQP8, ZKSCAN2, HS3ST4, MIR548W, C16orf82, KDM8, NSMCE1, FLJ21408, IL4R, IL21R, IL21R-AS1, GTF3C1, KIAA0556, GSG1L, XPO6, SBK1, NPIPB6, EIF3CL, EIF3C, MIR6862-1, MIR6862-2, CLN3, APOBR, IL27, NUPR1, SGF29, SULT1A2, SULT1A1, NPIPB8, NPIPB9, ATXN2L, TUFM, MIR4721, SH2B1, ATP2A1, ATP2A1-AS1, RABEP2, CD19, NFATC2IP, MIR4517, SPNS1, LAT, RRN3P2, SNX29P2, NPIPB11, SMG1P6, BOLA2-SMG1P6, LOC606724, BOLA2, BOLA2B, SLX1B, SLX1A, SLX1B-SULT1A4, SLX1A-SULT1A3, SULT1A4, SULT1A3, LOC388242, LOC613038, SMG1P2, SPN, QPRT, C16orf54, ZG16, KIF22, MAZ, PRRT2, PAGR1, MVP, CDIPT, CDIPT-AS1, SEZ6L2, ASPHD1, KCTD13, TMEM219, TAOK2, HIRIP3, INO80E, DOC2A, C16orf92, FAM57B, ALDOA, PPP4C, TBX6, YPEL3, LOC101928595, GDPD3, MAPK3, CORO1A, LOC613037, SMG1P5, CD2BP2, TBC1D10B, MYLPF, SEPT1, ZNF48, ZNF771, DCTPP1, SEPHS2, ITGAL, MIR4518, ZNF768, ZNF747, ZNF764, ZNF688, ZNF785, ZNF689, PRR14, FBRS, LOC730183, SRCAP, SNORA30, TMEM265, PHKG2, CCDC189, RNF40, ZNF629, BCL7C, MIR4519, MIR762HG, MIR762, CTF1, FBXL19-AS1, FBXL19, ORAI3, SETD1A, HSD3B7, STX1B, STX4, ZNF668, ZNF646, PRSS53, VKORC1, BCKDK, KAT8, PRSS8, PRSS36, FUS, PYCARD, PYCARD-AS1, TRIM72, PYDC1, ITGAM, ITGAX, ITGAD, COX6A2, ZNF843, ARMC5, TGFB1I1, SLC5A2, C16orf58, AHSP, FRG2KP, YBX3P1, CLUHP3, ZNF720, ZNF267, LOC102723753, HERC2P4, TP53TG3D, LOC390705, TP53TG3, TP53TG3B, TP53TG3C, SLC6A10P, ENPP7P13, LINC00273, UBE2MP1, LINC01566, FRG2DP, TP53TG3HP, FLJ26245, ANKRD26P1, SHCBP1, VPS35, ORC6, MYLK3, C16orf87, GPT2, DNAJA2, NETO2, ITFG1-AS1, ITFG1, PHKB, LOC100507534, LOC101927132, ABCC12, ABCC11, LONP2, MIR5095, LOC100507577, SIAH1, N4BP1, CBLN1, C16orf78, ZNF423, CNEP1R1, HEATR3, PAPD5, ADCY7, MIR6771, BRD7, NKD1, SNX20, LOC101927272, NOD2, CYLD, MIR3181, LOC101927334, SALL1, LINC01571, C16orf97, LINC00919, LOC102467079, CASC22, TOX3, CASC16, LOC105371267, CHD9, LOC643802, LOC102723373, RBL2, AKTIP, RPGRIP1L, FTO, FTO-IT1, LOC100996338, IRX3, LOC100996345, LOC101927480, CRNDE, IRX5, IRX6, MMP2, LPCAT2, CAPNS2, SLC6A2, CES1P2, CES1P1, CES1, CES5A, LOC283856, GNAO1, DKFZP434H168, MIR3935, AMFR, NUDT21, OGFOD1, BBS2, MT4, MT3, MT2A, MT1L, MT1E, MT1M, MT1JP, MT1A, MT1DP, MT1B, MT1F, MT1G, MT1H, MT1IP, MT1X, NUP93, MIR138-2, SLC12A3, MIR6863, HERPUD1, CETP, NLRC5, CPNE2, FAM192A, RSPRY1, ARL2BP, PLLP, CCL22, CX3CL1, CCL17, CIAPIN1, COQ9, POLR2C, DOK4, CCDC102A, ADGRG5, ADGRG1, ADGRG3, DRC7, KATNB1, KIFC3, MIR6772, LOC388282, CNGB1, TEPP, ZNF319, USB1, MMP15, CFAP20, CSNK2A2, CCDC113, PRSS54, GINS3, NDRG4, SETD6, CNOT1, SNORA46, SNORA50A, SLC38A7, GOT2, APOOP5, LOC101927580, LOC729159, MIR4426, CDH8, CDH11, LOC101927650, LINC00922, CDH5, LINC00920, BEAN1, BEAN1-AS1, TK2, CKLF, CKLF-CMTM1, CMTM1, CMTM2, CMTM3, CMTM4, DYNC1LI2, LOC106699570, TERB1, NAE1, CA7, PDP2, CDH16, RRAD, FAM96B, CES2, CES3, CES4A, CBFB, C16orf70, B3GNT9, TRADD, FBXL8, HSF4, NOL3, KIAA0895L, EXOC3L1, E2F4, ELMO3, MIR328, LRRC29, TMEM208, FHOD1, SLC9A5, PLEKHG4, KCTD19, LRRC36, TPPP3, ZDHHC1, HSD11B2, ATP6V0D1, AGRP, LOC100505942, FAM65A, CTCF, CARMIL2, ACD, PARD6A, ENKD1, C16orf86, GFOD2, RANBP10, TSNAXIP1, CENPT, THAP11, NUTF2, EDC4, NRN1L, PSKH1, CTRL, PSMB10, LCAT, SLC12A4, DPEP3, DPEP2, LOC100131303, DDX28, DUS2, NFATC3, ESRP2, MIR6773, PLA2G15, SLC7A6, SLC7A6OS, PRMT7, SMPD3, ZFP90, CDH3, CDH1, MIR7641-2, TANGO6, HAS3, CHTF8, UTP4, SNTB2, VPS4A, PDF, COG8, NIP7, TMED6, TERF2, CYB5B, MIR1538, NFAT5, NQO1, NOB1, WWP2, MIR140, CLEC18A, PDXDC2P, MIR1972-2, MIR1972-1, PDPR, LOC400541, CLEC18C, LOC105371328, SMG1P7, EXOSC6, AARS, DDX19B, LOC100506083, DDX19A, ST3GAL2, FUK, COG4, SF3B3, SNORD111B, SNORD111, IL34, MTSS1L, VAC14, VAC14-AS1, HYDIN, CMTR2, CALB2, ZNF23, ZNF19, LOC105371335, CHST4, TAT-AS1, TAT, MARVELD3, PHLPP2, SNORA70D, AP1G1, SNORD71, ATXN1L, ZNF821, IST1, PKD1L3, DHODH, HP, HPR, TXNL4B, DHX38, PMFBP1, LINC01572, ZFHX3, HCCAT5, C16orf47, LINC01568, LOC101928035, PSMD7, LOC283922, NPIPB15, LOC105376772, CLEC18B, GLG1, RFWD3, MLKL, FA2H, WDR59, ZNRF1, LDHD, ZFP1, CTRB2, CTRB1, LOC100506281, BCAR1, CFDP1, TMEM170A, CHST6, CHST5, TMEM231, GABARAPL2, ADAT1, KARS, TERF2IP, CNTNAP4, LOC101928203, MIR4719, MON1B, SYCE1L, ADAMTS18, NUDT7, VAT1L, CLEC3A, WWOX, MAF, MAFTRR, LINC01229, LOC102724084, DYNLRB2, LINC01227, CDYL2, PRCAT47, CMC2, CENPN, ATMIN, C16orf46, GCSH, PKD1L2, BCO1, GAN, MIR4720, CMIP, MIR7854, MIR6504, LOC100129617, PLCG2, SDR42E1, HSD17B2, MPHOSPH6, CDH13, MIR8058, LOC101928446, LOC101928417, MIR3182, LOC102724163, HSBP1, MLYCD, OSGIN1, NECAB2, SLC38A8, MBTPS1, HSDL1, DNAAF1, TAF1C, ADAD2, KCNG4, WFDC1, ATP2C2, TLDC1, COTL1, KLHL36, USP10, CRISPLD2, ZDHHC7, KIAA0513, FAM92B, LOC400548, LINC00311, MIR5093 | arr[GRCh37] 16p13.3q24.1(83886_85377126)x3 |
| 2  LM | Gain | 20 | p13 | p12.3 | 6,603 | DEFB125, DEFB126, DEFB127, DEFB128, DEFB129, DEFB132, C20orf96, ZCCHC3, NRSN2-AS1, SOX12, NRSN2, TRIB3, RBCK1, TBC1D20, CSNK2A1, TCF15, SRXN1, SCRT2, SLC52A3, FAM110A, ANGPT4, RSPO4, PSMF1, LOC105372493, TMEM74B, C20orf202, RAD21L1, SNPH, SDCBP2, FKBP1A-SDCBP2, SDCBP2-AS1, FKBP1A, MIR6869, NSFL1C, SIRPB2, SIRPD, SIRPB1, SIRPG, SIRPG-AS1, LOC100289473, SIRPA, LOC727993, PDYN, STK35, LOC388780, TGM3, TGM6, SNRPB, SNORD119, ZNF343, TMC2, NOP56, MIR1292, SNORD110, SNORA51, SNORD86, SNORD56, SNORD57, IDH3B, EBF4, CPXM1, C20orf141, TMEM239, PCED1A, VPS16, PTPRA, GNRH2, MRPS26, OXT, AVP, UBOX5-AS1, UBOX5, FASTKD5, LZTS3, DDRGK1, ITPA, SLC4A11, C20orf194, ATRN, GFRA4, ADAM33, SIGLEC1, HSPA12B, C20orf27, SPEF1, CENPB, CDC25B, LOC101929125, AP5S1, MAVS, PANK2, MIR103A2, MIR103B2, RNF24, SMOX, LINC01433, ADRA1D, PRNP, PRND, PRNT, RASSF2, SLC23A2, TMEM230, PCNA, PCNA-AS1, CDS2, PROKR2, LINC00658, LOC643406, LINC00654, LOC101929207, GPCPD1, C20orf196, CHGB, TRMT6, MCM8, MCM8-AS1, CRLS1, LRRN4, FERMT1, CASC20 | arr[GRCh37] 20p13p12.3(69093_6672360)x2 |
| 2  LM | Gain | 20 | q11.21 | q13.33 | 33,393 | LINC01598, FRG1BP, FRG1DP, MLLT10P1, DEFB115, DEFB116, DEFB118, DEFB119, DEFB121, DEFB122, DEFB123, DEFB124, REM1, LINC00028, HM13, HM13-AS1, ID1, MIR3193, COX4I2, BCL2L1, ABALON, TPX2, MYLK2, FOXS1, DUSP15, TTLL9, PDRG1, XKR7, MIR7641-2, CCM2L, HCK, TM9SF4, TSPY26P, PLAGL2, POFUT1, MIR1825, KIF3B, ASXL1, NOL4L, LOC101929698, LOC149950, C20orf203, COMMD7, DNMT3B, MAPRE1, SUN5, BPIFB2, BPIFB6, BPIFB3, BPIFB4, BPIFA2, BPIFA4P, BPIFA3, BPIFA1, BPIFB1, CDK5RAP1, SNTA1, CBFA2T2, NECAB3, C20orf144, ACTL10, E2F1, PXMP4, ZNF341, ZNF341-AS1, CHMP4B, RALY-AS1, RALY, MIR4755, EIF2S2, ASIP, AHCY, ITCH, MIR644A, DYNLRB1, MAP1LC3A, PIGU, TP53INP2, NCOA6, HMGB3P1, GGT7, ACSS2, GSS, MYH7B, MIR499A, MIR499B, TRPC4AP, EDEM2, PROCR, MMP24, MMP24-AS1, EIF6, FAM83C-AS1, FAM83C, UQCC1, GDF5, MIR1289-1, CEP250, C20orf173, ERGIC3, FER1L4, SPAG4, CPNE1, RBM12, NFS1, ROMO1, RBM39, PHF20, SCAND1, CNBD2, NORAD, EPB41L1, AAR2, DLGAP4, DLGAP4-AS1, MYL9, TGIF2, TGIF2-C20orf24, C20orf24, SLA2, NDRG3, DSN1, SOGA1, TLDC2, SAMHD1, RBL1, MROH8, RPN2, GHRH, MANBAL, SRC, BLCAP, NNAT, LINC00489, LOC100287792, CTNNBL1, VSTM2L, TTI1, RPRD1B, TGM2, KIAA1755, LOC149684, BPI, LBP, SNHG17, SNORA71B, SNORA71A, SNORA71C, SNORA71D, SNHG11, SNORA71E, SNORA60, RALGAPB, MIR548O2, ADIG, ARHGAP40, SLC32A1, ACTR5, PPP1R16B, FAM83D, DHX35, LOC339568, LINC01370, MAFB, LOC100128988, TOP1, PLCG1-AS1, PLCG1, MIR6871, ZHX3, LPIN3, EMILIN3, CHD6, PTPRT, LOC101927159, SRSF6, L3MBTL1, SGK2, IFT52, MYBL2, GTSF1L, LOC105372626, TOX2, JPH2, OSER1, OSER1-AS1, GDAP1L1, FITM2, R3HDML, HNF4A, HNF4A-AS1, MIR3646, LINC01430, LINC01620, TTPAL, SERINC3, PKIG, ADA, LINC01260, KCNK15-AS1, WISP2, KCNK15, RIMS4, YWHAB, PABPC1L, TOMM34, STK4-AS1, STK4, KCNS1, WFDC5, WFDC12, PI3, SEMG1, SEMG2, SLPI, MATN4, RBPJL, SDC4, SYS1, SYS1-DBNDD2, TP53TG5, DBNDD2, PIGT, MIR6812, WFDC2, SPINT3, WFDC6, EPPIN-WFDC6, EPPIN, WFDC8, WFDC9, WFDC10A, WFDC11, WFDC10B, WFDC13, MIR3617, SPINT4, WFDC3, DNTTIP1, UBE2C, TNNC2, SNX21, ACOT8, ZSWIM3, ZSWIM1, SPATA25, NEURL2, CTSA, PLTP, PCIF1, ZNF335, MMP9, SLC12A5, NCOA5, CD40, CDH22, SLC35C2, ELMO2, ZNF663P, MKRN7P, ZNF334, OCSTAMP, SLC13A3, TP53RK, SLC2A10, EYA2, MIR3616, ZMYND8, LOC100131496, LOC101927377, NCOA3, SULF2, LINC01522, LINC01523, LINC00494, PREX1, ARFGEF2, CSE1L-AS1, CSE1L, STAU1, DDX27, ZNFX1, ZFAS1, SNORD12C, SNORD12B, SNORD12, KCNB1, PTGIS, B4GALT5, SLC9A8, SPATA2, LOC105372653, RNF114, SNAI1, TRERNA1, UBE2V1, TMEM189-UBE2V1, TMEM189, LINC01273, CEBPB-AS1, CEBPB, LINC01272, LINC01270, LINC01271, PTPN1, MIR645, FAM65C, MIR1302-5, LOC100506175, PARD6B, BCAS4, ADNP, ADNP-AS1, DPM1, MOCS3, KCNG1, NFATC2, MIR3194, ATP9A, SALL4, LINC01429, ZFP64, LINC01524, TSHZ2, LOC101927770, ZNF217, LOC105372672, SUMO1P1, BCAS1, MIR4756, CYP24A1, PFDN4, DOK5, LINC01441, LINC01440, CBLN4, MC3R, FAM210B, AURKA, CSTF1, CASS4, RTFDC1, GCNT7, FAM209A, FAM209B, LOC105372682, TFAP2C, BMP7, BMP7-AS1, MIR4325, SPO11, RAE1, MTRNR2L3, RBM38, CTCFL, PCK1, ZBP1, PMEPA1, NKILA, MIR4532, C20orf85, ANKRD60, PPP4R1L, RAB22A, VAPB, APCDD1L, APCDD1L-AS1, LOC79160, STX16, STX16-NPEPL1, NPEPL1, LOC105372695, MIR296, MIR298, GNAS-AS1, GNAS, LOC101927932, NELFCD, CTSZ, TUBB1, ATP5E, SLMO2-ATP5E, PRELID3B, ZNF831, EDN3, PHACTR3, LOC100506384, SYCP2, FAM217B, PPP1R3D, CDH26, C20orf197, LOC729296, MIR646HG, MIR646, LOC101928048, MIR4533, MIR548AG2, LOC100506470, CDH4, MIR1257, TAF4, MIR3195, LSM14B, PSMA7, SS18L1, MTG2, HRH3, OSBPL2, ADRM1, LAMA5, MIR4758, LAMA5-AS1, RPS21, CABLES2, RBBP8NL, GATA5, C20orf166-AS1, MIR1-1HG, MIR1-1, MIR133A2, SLCO4A1, SLCO4A1-AS1, NTSR1, LINC00659, MRGBP, OGFR-AS1, OGFR, COL9A3, TCFL5, DPH3P1, DIDO1, GID8, SLC17A9, BHLHE23, LOC63930, LINC00029, LINC01056, HAR1B, HAR1A, MIR124-3, YTHDF1, BIRC7, MIR3196, NKAIN4, FLJ16779, ARFGAP1, MIR4326, COL20A1, CHRNA4, LOC100130587, KCNQ2, EEF1A2, PPDPF, PTK6, SRMS, FNDC11, HELZ2, GMEB2, LOC100505771, STMN3, RTEL1, RTEL1-TNFRSF6B, TNFRSF6B, ARFRP1, ZGPAT, LIME1, SLC2A4RG, ZBTB46, ZBTB46-AS1, ABHD16B, TPD52L2, DNAJC5, MIR941-1, MIR941-4, MIR941-3, MIR941-2, MIR941-5, UCKL1, MIR1914, MIR647, UCKL1-AS1, ZNF512B, SAMD10, PRPF6, LINC00176, SOX18, TCEA2, RGS19, MIR6813, OPRL1, LKAAEAR1, NPBWR2, MYT1, PCMTD2 | arr[GRCh37] 20q11.21q13.33(29519155_62912463)x2 |
| 2  LM | Loss | 8 | p23.3 | p11.22 | 39,453 | RPL23AP53, ZNF596, FAM87A, FBXO25, TDRP, ERICH1, ERICH1-AS1, LOC401442, LOC286083, DLGAP2, DLGAP2-AS1, LOC101927752, CLN8, MIR3674, MIR596, ARHGEF10, LOC101928058, KBTBD11-OT1, KBTBD11, MYOM2, MIR7160, LOC101927815, CSMD1, LOC100287015, MCPH1, ANGPT2, MCPH1-AS1, MIR8055, AGPAT5, MIR4659A, MIR4659B, XKR5, GS1-24F4.2, DEFB1, DEFA6, DEFA4, DEFA8P, DEFA9P, DEFA10P, DEFA1, DEFA1B, DEFT1P2, DEFT1P, DEFA3, DEFA11P, DEFA5, LINC00965, FAM66B, DEFB109P1B, USP17L1, USP17L4, ZNF705G, DEFB4B, DEFB103B, DEFB103A, SPAG11B, DEFB104A, DEFB104B, DEFB106B, DEFB106A, DEFB105B, DEFB105A, DEFB107B, DEFB107A, PRR23D1, PRR23D2, FAM90A7P, FAM90A10P, SPAG11A, DEFB4A, ZNF705B, FAM66E, USP17L8, USP17L3, MIR548I3, FAM86B3P, SGK223, CLDN23, MFHAS1, ERI1, MIR4660, PPP1R3B, LOC101929128, LOC157273, TNKS, MIR597, LINC00599, MIR124-1, MSRA, LINCR-0001, PRSS55, RP1L1, MIR4286, C8orf74, SOX7, PINX1, MIR1322, LOC101929229, XKR6, MIR598, LOC101929269, MTMR9, SLC35G5, TDH, FAM167A-AS1, FAM167A, BLK, LINC00208, GATA4, SNORA99, C8orf49, NEIL2, FDFT1, CTSB, DEFB136, DEFB135, DEFB134, LOC100133267, DEFB130, ZNF705D, FAM66D, LOC392196, USP17L7, USP17L2, FAM90A2P, FAM86B1, FAM66A, LOC649352, DEFB109P1, FAM90A25P, FAM86B2, LOC100506990, LOC729732, MIR5692A1, MIR5692A2, LONRF1, MIR3926-1, MIR3926-2, LOC340357, LINC00681, KIAA1456, DLC1, C8orf48, LOC102725080, SGCZ, MIR383, TUSC3, MSR1, FGF20, MICU3, ZDHHC2, CNOT7, VPS37A, MTMR7, SLC7A2, PDGFRL, MTUS1, MIR548V, FGL1, PCM1, ASAH1, LOC101929066, NAT1, NAT2, PSD3, LOC100128993, SH2D4A, CSGALNACT1, INTS10, LPL, SLC18A1, ATP6V1B2, LZTS1, LZTS1-AS1, LOC102467222, LOC286114, LOC101929172, GFRA2, DOK2, XPO7, NPM2, FGF17, DMTN, FAM160B2, NUDT18, HR, REEP4, LGI3, SFTPC, BMP1, PHYHIP, MIR320A, POLR3D, LOC100507071, PIWIL2, SLC39A14, PPP3CC, SORBS3, PDLIM2, C8orf58, CCAR2, BIN3, BIN3-IT1, EGR3, PEBP4, LOC101929237, RHOBTB2, TNFRSF10B, LOC286059, LOC254896, TNFRSF10C, TNFRSF10D, TNFRSF10A, LOC389641, CHMP7, R3HCC1, LOXL2, LOC100507156, ENTPD4, SLC25A37, NKX3-1, NKX2-6, STC1, ADAM28, LOC101929294, ADAMDEC1, ADAM7, LOC101929315, NEFM, NEFL, MIR6841, DOCK5, MIR6876, GNRH1, KCTD9, CDCA2, EBF2, PPP2R2A, BNIP3L, PNMA2, DPYSL2, ADRA1A, STMN4, TRIM35, PTK2B, MIR6842, CHRNA2, EPHX2, CLU, MIR6843, SCARA3, MIR3622B, MIR3622A, CCDC25, ESCO2, PBK, SCARA5, MIR4287, NUGGC, ELP3, PNOC, ZNF395, FBXO16, FZD3, MIR4288, MIR7641-2, EXTL3-AS1, EXTL3, INTS9, HMBOX1, KIF13B, DUSP4, LINC00589, LOC101929450, LOC101929470, FAM183CP, MIR3148, SARAF, LEPROTL1, MBOAT4, DCTN6, RBPMS-AS1, RBPMS, GTF2E2, SMIM18, GSR, UBXN8, PPP2CB, TEX15, PURG, WRN, NRG1, NRG1-IT1, NRG1-IT3, FUT10, MAK16, TTI2, RNF122, DUSP26, LINC01288, UNC5D, LOC101929550, KCNU1, MIR1268A, LINC01605, ZNF703, LOC101929622, LOC102723701, ERLIN2, LOC728024, PROSC, ADGRA2, BRF2, RAB11FIP1, GOT1L1, ADRB3, EIF4EBP1, ASH2L, STAR, LSM1, BAG4, DDHD2, PLPP5, WHSC1L1, LETM2, FGFR1, C8orf86, RNF5P1, TACC1, PLEKHA2, HTRA4, TM2D2, ADAM9, ADAM32, ADAM5, ADAM3A, LOC100130964, ADAM18, ADAM2 | arr[GRCh37] 8p23.3p11.22(172416_39625680)x1 |
| 2  LM | Loss | 12 | p13.33 | p11.1 | 34,639 | IQSEC3, LOC574538, SLC6A12, LOC101929384, SLC6A13, LOC102723544, KDM5A, CCDC77, B4GALNT3, NINJ2, LOC105369595, LOC100049716, WNK1, RAD52, ERC1, LINC00942, FBXL14, WNT5B, MIR3649, ADIPOR2, CACNA2D4, LRTM2, LINC00940, DCP1B, CACNA1C-IT2, CACNA1C, CACNA1C-AS4, CACNA1C-IT3, CACNA1C-AS2, CACNA1C-AS1, LOC283440, FKBP4, ITFG2, NRIP2, LOC100507424, FOXM1, RHNO1, TULP3, TEAD4, TSPAN9, PRMT8, THCAT155, CRACR2A, PARP11, CCND2-AS1, CCND2, TIGAR, FGF23, FGF6, C12orf4, RAD51AP1, DYRK4, AKAP3, NDUFA9, LOC101929549, GALNT8, KCNA6, KCNA1, KCNA5, LOC101929584, NTF3, ANO2, VWF, CD9, PLEKHG6, TNFRSF1A, SCNN1A, LTBR, CD27-AS1, CD27, TAPBPL, VAMP1, MRPL51, NCAPD2, SCARNA10, GAPDH, IFFO1, NOP2, CHD4, SCARNA11, LPAR5, ACRBP, ING4, ZNF384, PIANP, COPS7A, MLF2, PTMS, LAG3, CD4, GPR162, P3H3, GNB3, CDCA3, USP5, TPI1, SPSB2, LOC105369632, RPL13P5, DSTNP2, LRRC23, ENO2, ATN1, C12orf57, PTPN6, LOC105369635, MIR200C, MIR141, PHB2, SCARNA12, EMG1, LPCAT3, C1S, C1R, C1RL, C1RL-AS1, RBP5, CLSTN3, PEX5, ACSM4, CD163L1, CD163, APOBEC1, GDF3, DPPA3, CLEC4C, NANOGNB, NANOG, SLC2A14, SLC2A3, FOXJ2, C3AR1, NECAP1, CLEC4A, POU5F1P3, ZNF705A, FAM66C, FAM90A1, FAM86FP, LOC101927905, LINC00937, CLEC6A, CLEC4D, CLEC4E, AICDA, MFAP5, RIMKLB, A2ML1, PHC1, M6PR, KLRG1, LINC00612, A2M-AS1, A2M, PZP, A2MP1, MIR1244-4, MIR1244-3, MIR1244-1, MIR1244-2, LINC00987, LOC642846, LOC101930452, LOC101928030, DDX12P, KLRB1, LOC374443, CLEC2D, CLECL1, CD69, KLRF1, CLEC2B, KLRF2, CLEC2A, LOC100506159, LOC400002, CLEC12A, CLEC1B, CLEC12B, LOC102724020, CLEC9A, CLEC1A, CLEC7A, OLR1, TMEM52B, GABARAPL1, KLRD1, LOC101928100, KLRK1, KLRC4-KLRK1, KLRC4, KLRC3, KLRC2, KLRC1, KLRA1P, MAGOHB, STYK1, YBX3, LOC101928162, TAS2R7, TAS2R8, TAS2R9, TAS2R10, PRR4, PRH1-PRR4, PRH1, TAS2R13, PRH2, PRH1-TAS2R14, TAS2R14, TAS2R50, TAS2R20, TAS2R19, TAS2R31, TAS2R46, TAS2R43, TAS2R30, SMIM10L1, TAS2R42, PRB3, PRB4, PRB1, PRB2, LINC01252, ETV6, BCL2L14, LRP6, MANSC1, LOH12CR2, BORCS5, DUSP16, CREBL2, GPR19, CDKN1B, APOLD1, MIR613, DDX47, RPL13AP20, GPRC5A, MIR614, GPRC5D, HEBP1, LOC100506314, HTR7P1, FAM234B, MIR7641-2, GSG1, EMP1, LINC01559, GRIN2B, ATF7IP, PLBD1, PLBD1-AS1, GUCY2C, HIST4H4, H2AFJ, WBP11, C12orf60, SMCO3, ART4, MGP, ERP27, ARHGDIB, PDE6H, LINC01489, RERG, RERG-AS1, PTPRO, EPS8, STRAP, DERA, SLC15A5, MGST1, LMO3, SKP1P2, MIR3974, RERGL, PIK3C2G, PLCZ1, CAPZA3, PLEKHA5, AEBP2, LOC100506393, PDE3A, SLCO1C1, SLCO1B3, SLCO1B7, SLCO1B1, SLCO1A2, IAPP, PYROXD1, RECQL, GOLT1B, SPX, GYS2, LDHB, KCNJ8, ABCC9, CMAS, ST8SIA1, C2CD5, LOC105369691, ETNK1, LOC101928441, SOX5, MIR920, LOC101928471, LINC00477, BCAT1, C12orf77, LOC645177, LRMP, CASC1, LYRM5, KRAS, LMNTD1, MIR4302, RASSF8-AS1, RASSF8, BHLHE41, SSPN, ITPR2, ASUN, FGFR1OP2, TM7SF3, MED21, C12orf71, STK38L, ARNTL2, ARNTL2-AS1, SMCO2, PPFIBP1, REP15, MRPS35, MANSC4, KLHL42, PTHLH, CCDC91, FAR2, LOC100506606, ERGIC2, OVCH1-AS1, OVCH1, TMTC1, IPO8, CAPRIN2, LOC645485, LINC00941, TSPAN11, DDX11-AS1, DDX11, FAM60A, FLJ13224, DENND5B, DENND5B-AS1, ETFBKMT, AMN1, H3F3C, LOC105369723, KIAA1551, BICD1, FGD4, DNM1L, YARS2, PKP2, SYT10, ALG10 | arr[GRCh37] 12p13.33p11.1(189399_34828211)x1 |
| 2  LM | Loss | 17 | p13.3 | p11.2 | 19,819 | VPS53, FAM57A, GEMIN4, DBIL5P, GLOD4, MRM3, NXN, LOC101927727, TIMM22, ABR, MIR3183, BHLHA9, TUSC5, YWHAE, CRK, MYO1C, INPP5K, PITPNA-AS1, PITPNA, SLC43A2, SCARF1, RILP, PRPF8, TLCD2, MIR22HG, MIR22, WDR81, SERPINF2, SERPINF1, SMYD4, RPA1, RTN4RL1, LOC105371485, DPH1, OVCA2, MIR132, MIR212, HIC1, SMG6, LOC101927839, SRR, TSR1, SNORD91B, SNORD91A, SGSM2, MNT, LOC284009, METTL16, PAFAH1B1, CLUH, MIR6776, LOC105371592, MIR1253, RAP1GAP2, LOC101927911, OR1D5, OR1D2, OR1G1, OR1A2, OR1A1, OR1D4, OR3A2, OR3A1, OR3A4P, OR1E1, OR3A3, OR1E2, SPATA22, ASPA, TRPV3, TRPV1, SHPK, CTNS, TAX1BP3, P2RX5-TAX1BP3, EMC6, P2RX5, ITGAE, GSG2, NCBP3, CAMKK1, P2RX1, ATP2A3, ZZEF1, CYB5D2, ANKFY1, UBE2G1, LOC103021295, SPNS3, SPNS2, MYBBP1A, GGT6, SMTNL2, ALOX15, PELP1, LOC101559451, ARRB2, MED11, CXCL16, ZMYND15, TM4SF5, VMO1, GLTPD2, PSMB6, PLD2, MINK1, CHRNE, C17orf107, GP1BA, SLC25A11, RNF167, PFN1, ENO3, SPAG7, CAMTA2, MIR6864, MIR6865, INCA1, KIF1C, LOC102724009, SLC52A1, ZFP3, ZNF232, LOC101928000, USP6, ZNF594, LOC100130950, SCIMP, RABEP1, NUP88, RPAIN, C1QBP, DHX33, LOC105371506, DERL2, MIS12, LOC728392, NLRP1, LOC339166, WSCD1, AIPL1, FAM64A, PITPNM3, KIAA0753, TXNDC17, MED31, C17orf100, MIR4520-1, MIR4520-2, ALOX15P1, SLC13A5, XAF1, FBXO39, TEKT1, ALOX12P2, ALOX12-AS1, ALOX12, RNASEK, RNASEK-C17orf49, C17orf49, MIR497HG, MIR195, MIR497, BCL6B, SLC16A13, SLC16A11, CLEC10A, ASGR2, ASGR1, DLG4, ACADVL, MIR324, DVL2, PHF23, GABARAP, CTDNEP1, ELP5, CLDN7, SLC2A4, YBX2, EIF5A, GPS2, NEURL4, ACAP1, KCTD11, TMEM95, TNK1, PLSCR3, TMEM256-PLSCR3, TMEM256, NLGN2, SPEM1, C17orf74, TMEM102, FGF11, CHRNB1, ZBTB4, SLC35G6, POLR2A, TNFSF12, TNFSF12-TNFSF13, TNFSF13, SENP3, SENP3-EIF4A1, EIF4A1, SNORA48, SNORD10, SNORA67, CD68, LOC100996842, MPDU1, SOX15, FXR2, SHBG, SAT2, ATP1B2, TP53, WRAP53, EFNB3, DNAH2, RPL29P2, KDM6B, TMEM88, NAA38, CYB5D1, CHD3, SCARNA21, LOC284023, KCNAB3, TRAPPC1, CNTROB, GUCY2D, ALOX15B, ALOX12B, MIR4314, ALOXE3, HES7, PER1, MIR6883, VAMP2, TMEM107, MIR4521, BORCS6, AURKB, LINC00324, CTC1, PFAS, SLC25A35, RANGRF, ARHGEF15, ODF4, LOC100128288, KRBA2, RPL26, RNF222, NDEL1, MYH10, CCDC42, SPDYE4, MFSD6L, PIK3R6, PIK3R5, NTN1, LOC101928266, STX8, CFAP52, USP43, DHRS7C, GSG1L2, GLP2R, RCVRN, GAS7, MYH13, MYHAS, MYH8, MYH4, MYH1, MYH2, MYH3, SCO1, ADPRM, TMEM220, MAGOH2P, TMEM220-AS1, LINC00675, PIRT, SHISA6, DNAH9, ZNF18, MAP2K4, MIR744, LINC00670, MYOCD, LOC101928418, LOC100128006, ARHGAP44, MIR1269B, ELAC2, HS3ST3A1, CDRT15P1, COX10-AS1, COX10, CDRT15, HS3ST3B1, MGC12916, LOC101928475, CDRT7, CDRT8, PMP22, MIR4731, TEKT3, CDRT4, TVP23C-CDRT4, TVP23C, CDRT1, TRIM16, ZNF286A, TBC1D26, CDRT15P2, MEIS3P1, LOC101928567, ADORA2B, ZSWIM7, TTC19, NCOR1, PIGL, MIR1288, CENPV, UBB, TRPV2, LRRC75A-AS1, SNORD49B, SNORD49A, SNORD65, LRRC75A, ZNF287, ZNF624, CCDC144A, USP32P1, FAM106CP, KRT16P2, TNFRSF13B, MPRIP, PLD6, FLCN, COPS3, NT5M, MED9, RASD1, PEMT, SMCR2, RAI1, RAI1-AS1, SMCR5, SREBF1, MIR6777, MIR33B, TOM1L2, DRC3, ATPAF2, GID4, DRG2, MYO15A, ALKBH5, LLGL1, FLII, MIEF2, TOP3A, SMCR8, SHMT1, MIR6778, EVPLL, FLJ35934, KRT17P5, KRT16P1, LGALS9C, USP32P2, FAM106A, CCDC144B, TBC1D28, ZNF286B, FOXO3B, TRIM16L, FBXW10, TVP23B, PRPSAP2, SLC5A10, FAM83G, GRAP, LOC79999, LOC388436, GRAPL, EPN2, EPN2-IT1, EPN2-AS1, B9D1, MIR1180, MAPK7, MFAP4, RNF112, SLC47A1, SNORA59B, SNORA59A, ALDH3A2, SLC47A2, ALDH3A1, ULK2, AKAP10, SPECC1 | arr[GRCh37] 17p13.3p11.2(400958_20220266)x1 |
| 2  LM | Loss | 18 | q11.2 | q23 | 54,465 | SS18, PSMA8, TAF4B, LINC01543, KCTD1, MIR8057, PCAT18, AQP4, AQP4-AS1, CHST9, LOC105372038, CDH2, MIR302F, DSC3, DSC2, DSCAS, DSC1, DSG1, DSG1-AS1, DSG4, DSG3, DSG2, DSG2-AS1, TTR, B4GALT6, SLC25A52, TRAPPC8, RNF125, RNF138, MEP1B, GAREM1, WBP11P1, KLHL14, CCDC178, ASXL3, NOL4, DTNA, MAPRE2, ZNF397, ZSCAN30, ZNF271P, ZNF24, ZNF396, INO80C, MIR3975, GALNT1, MIR187, MIR3929, C18orf21, RPRD1A, SLC39A6, ELP2, LOC101927809, MOCOS, FHOD3, LOC105372071, TPGS2, KIAA1328, LOC105372069, CELF4, LOC105372068, SNORA111, MIR4318, MIR924HG, MIR924, MIR5583-2, MIR5583-1, LINC01477, KC6, PIK3C3, LINC00907, RIT2, SYT4, LINC01478, LOC105667213, SETBP1, MIR4319, SLC14A2, SLC14A2-AS1, SLC14A1, SIGLEC15, EPG5, PSTPIP2, ATP5A1, HAUS1, C18orf25, RNF165, LOXHD1, ST8SIA5, PIAS2, KATNAL2, TCEB3CL, TCEB3CL2, TCEB3C, TCEB3B, HDHD2, IER3IP1, SKOR2, MIR4527, SMAD2, ZBTB7C, CTIF, MIR4743, SMAD7, DYM, MIR4744, C18orf32, RPL17-C18orf32, MIR1539, RPL17, SNORD58C, SNORD58A, SNORD58B, LIPG, ACAA2, SCARNA17, SNHG22, MYO5B, MIR4320, CFAP53, MBD1, CXXC1, SKA1, MAPK4, MRO, ME2, ELAC1, SMAD4, MEX3C, LINC01630, DCC, MIR4528, LOC102724651, LOC101928167, MBD2, SNORA37, POLI, STARD6, C18orf54, DYNAP, RAB27B, CCDC68, LOC101927229, TCF4, TCF4-AS1, MIR4529, LINC01416, LINC01539, TXNL1, WDR7, LINC-ROR, BOD1L2, ST8SIA3, ONECUT2, FECH, NARS, LOC100505549, ATP8B1, NEDD4L, MIR122, MIR3591, ALPK2, SNORA108, LOC101927322, MALT1, ZNF532, OACYLP, SEC11C, GRP, RAX, CPLX4, LMAN1, CCBE1, PMAIP1, MC4R, CDH20, LINC01544, RNF152, PIGN, KIAA1468, TNFRSF11A, ZCCHC2, PHLPP1, BCL2, KDSR, VPS4B, SERPINB5, SERPINB12, SERPINB13, SERPINB4, SERPINB3, SERPINB11, SERPINB7, SERPINB2, SERPINB10, HMSD, SERPINB8, LINC00305, LOC284294, LINC01538, CDH7, CDH19, MIR5011, DSEL, LOC643542, TMX3, CCDC102B, DOK6, LOC105372179, CD226, RTTN, SOCS6, LOC101927481, LOC101060542, GTSCR1, LINC01541, LOC102724913, CBLN2, NETO1, MIR548AV, LOC100505797, LOC400655, LOC100505817, FBXO15, TIMM21, CYB5A, C18orf63, LOC101927606, FAM69C, CNDP2, CNDP1, LINC00909, ZNF407, ZADH2, TSHZ1, SMIM21, LOC100505853, LOC339298, ZNF516, LOC101927989, C18orf65, LINC00908, LINC00683, LOC101927651, LOC400661, LOC100131655, ZNF236, MBP, GALR1, LINC01029, SALL3, ATP9B, NFATC1, LOC284241, CTDP1, KCNG2, PQLC1, HSBP1L1, TXNL4A, RBFA, RBFADN, ADNP2, PARD6G-AS1, PARD6G | arr[GRCh37] 18q11.2q23(23542721_78007784)x1 |
| 2  LM | Loss | 20 | p12.3 | p11.21 | 16,076 | BMP2, LINC01428, LOC101929288, LOC101929312, MIR8062, HAO1, TMX4, PLCB1, PLCB4, LAMP5-AS1, LAMP5, PAK5, LOC101929371, SNAP25-AS1, ANKEF1, SNAP25, MKKS, SLX4IP, JAG1, MIR6870, LOC101929395, LOC101929413, LOC339593, LINC00687, BTBD3, LOC101929486, LOC102606466, LOC100505515, SPTLC3, ISM1, ISM1-AS1, TASP1, ESF1, NDUFAF5, SEL1L2, MACROD2, FLRT3, MACROD2-IT1, MACROD2-AS1, LOC613266, KIF16B, SNRPB2, OTOR, PCSK2, BFSP1, DSTN, RRBP1, BANF2, SNX5, SNORD17, MGME1, OVOL2, PET117, KAT14, ZNF133, LINC00851, DZANK1, POLR3F, MIR3192, RBBP9, SEC23B, LINC00493, DTD1, LOC101929526, LINC00652, LOC100270804, C20orf78, SCP2D1, SLC24A3, LOC100130264, RIN2, NAA20, CRNKL1, CFAP61, INSM1, RALGAPA2, KIZ, KIZ-AS1, XRN2, NKX2-4, NKX2-2, LOC101929625, LOC101929608, PAX1, LINC01432, LINC01427, LOC284788, LINC00261, FOXA2, LINC01384 | arr[GRCh37] 20p12.3p11.21(6681990_22757960)x1 |
| 2  LM | Loss | 21 | q11.2 | q22.3 | 33,753 | ANKRD30BP2, MIR3156-3, LOC102724188, POTED, MIR3118-1, MIR8069-1, MIR8069-2, CYP4F29P, ANKRD20A11P, LIPI, RBM11, ABCC13, HSPA13, SAMSN1, SAMSN1-AS1, LOC388813, NRIP1, USP25, MIR99AHG, MIR99A, MIRLET7C, MIR125B2, LINC01549, CXADR, BTG3, C21orf91-OT1, C21orf91, CHODL-AS1, CHODL, TMPRSS15, MIR548XHG, MIR548X, LINC00320, NCAM2, LINC00317, LINC01425, LOC101927843, LINC00308, MIR6130, D21S2088E, LOC101927869, LOC105372751, LOC339622, LINC00158, MIR155HG, MIR155, LINC00515, MRPL39, JAM2, ATP5J, GABPA, APP, CYYR1-AS1, CYYR1, ADAMTS1, ADAMTS5, MIR4759, LINC00113, LINC00314, LOC284825, LOC101927973, LINC00161, N6AMT1, LTN1, RWDD2B, USP16, CCT8, MAP3K7CL, LINC00189, BACH1, BACH1-IT2, GRIK1, GRIK1-AS2, GRIK1-AS1, CLDN17, LINC00307, CLDN8, KRTAP24-1, KRTAP25-1, KRTAP26-1, KRTAP27-1, KRTAP23-1, KRTAP13-2, MIR4327, KRTAP13-1, KRTAP13-3, KRTAP13-4, KRTAP15-1, KRTAP19-1, KRTAP19-2, KRTAP19-3, KRTAP19-4, KRTAP19-5, KRTAP19-6, KRTAP19-7, KRTAP22-2, KRTAP6-3, KRTAP6-2, KRTAP22-1, KRTAP6-1, KRTAP20-1, KRTAP20-4, KRTAP20-2, KRTAP20-3, KRTAP21-3, KRTAP21-2, KRTAP21-1, KRTAP8-1, KRTAP7-1, KRTAP11-1, KRTAP19-8, TIAM1, LOC150051, SOD1, SCAF4, HUNK, LINC00159, MIS18A, MRAP, URB1, SNORA80A, URB1-AS1, EVA1C, TCP10L, C21orf59, SYNJ1, PAXBP1-AS1, PAXBP1, C21orf62-AS1, C21orf62, LOC102724502, OLIG2, LINC00945, OLIG1, LOC101928107, LINC01548, IFNAR2, IL10RB-AS1, IL10RB, IFNAR1, IFNGR2, TMEM50B, DNAJC28, GART, SON, MIR6501, DONSON, CRYZL1, ITSN1, ATP5O, LINC00649, LOC101928126, SLC5A3, MRPS6, LINC00310, KCNE2, SMIM11A, SMIM11B, C21orf140, KCNE1, RCAN1, CLIC6, LINC00160, LINC01426, RUNX1, RUNX1-IT1, LOC100506403, MIR802, LOC101928269, LINC01436, SETD4, LOC100133286, CBR1, CBR3-AS1, CBR3, DOPEY2, MORC3, CHAF1B, CLDN14, SIM2, HLCS, RIPPLY3, LOC105372795, PIGP, TTC3, DSCR9, DSCR3, DYRK1A, KCNJ6, DSCR4, DSCR8, DSCR10, KCNJ15, LINC01423, ERG, LINC00114, ETS2, LOC101928398, LOC400867, LOC101928435, PSMG1, BRWD1, BRWD1-IT2, BRWD1-AS1, HMGN1, WRB-SH3BGR, WRB, LCA5L, SH3BGR, MIR6508, B3GALT5-AS1, B3GALT5, IGSF5, PCP4, DSCAM, MIR4760, DSCAM-AS1, DSCAM-IT1, LINC00323, MIR3197, BACE2, PLAC4, FAM3B, MX2, MX1, TMPRSS2, LINC00111, LINC00479, LINC00112, RIPK4, MIR6814, PRDM15, C2CD2, SNORA91, ZBTB21, ZNF295-AS1, UMODL1, UMODL1-AS1, ABCG1, TFF3, TFF2, TFF1, TMPRSS3, UBASH3A, RSPH1, LOC101930094, SLC37A1, LOC101928233, PDE9A, LOC101928311, WDR4, NDUFV3, ERVH48-1, MIR5692B, PKNOX1, CBS, CBSL, U2AF1, U2AF1L5, LOC106780825, FRGCA, CRYAA, LOC102724652, LINC00322, TCONS_00029157, LOC102724428, SIK1, LINC00319, LOC102724354, LINC00313, HSF2BP, MIR6070, RRP1B, PDXK, CSTB, RRP1, AATBC, AGPAT3, TRAPPC10, PWP2, C21orf33, ICOSLG, DNMT3L, LOC105372833, AIRE, PFKL, C21orf2, TRPM2, TRPM2-AS, LRRC3-AS1, LRRC3, TSPEAR, TSPEAR-AS1, TSPEAR-AS2, KRTAP10-1, KRTAP10-2, KRTAP10-3, KRTAP10-4, KRTAP10-5, KRTAP10-6, KRTAP10-7, KRTAP10-8, KRTAP10-9, KRTAP10-10, KRTAP10-11, KRTAP12-4, KRTAP12-3, KRTAP12-2, KRTAP12-1, KRTAP10-12, UBE2G2, LINC01424, SUMO3, PTTG1IP, ITGB2, ITGB2-AS1, LINC01547, FAM207A, LINC00163, PICSAR, SSR4P1, ADARB1, LINC00334, POFUT2, LOC642852, LINC00316, COL18A1, COL18A1-AS2, COL18A1-AS1, MIR6815, SLC19A1, LOC100129027, PCBP3, LOC101928796, COL6A1, COL6A2, FTCD, SPATC1L, LSS, MCM3AP-AS1, MCM3AP, YBEY, C21orf58, PCNT, DIP2A, DIP2A-IT1, S100B, PRMT2 | arr[GRCh37] 21q11.2q22.3(14344536_48097610)x1 |
[truncated: 654,529 more chars]
