# Supplementary material for: SNP array genomic analysis of matched pairs of brain and liver metastases in primary colorectal cancer
Source: J Cancer Res Clin Oncol. 2023 Nov 27;149(20):18173–83. doi: 10.1007/s00432-023-05505-4 (PMC10725338; doi:10.1007/s00432-023-05505-4)
Supplement: Supplementary file 4 — Supplementary file4 (DOCX 42 KB) [file 432_2023_5505_MOESM4_ESM.docx]

**Table 5** **Detailed overview of all detected CNV using SNP array**. For all detected chromosomal aberrations, the length and position as well as the entity (CRC-Colorectal carcinoma; LM – liver metastasis, BM – brain metastasis) where each aberration was detected are given. Additionally, the availability of literature for the detected CNVs are listed. Aberrations of each entity without available literature are marked in bold.

| **ID** | **chromosomal region** | **aberration** | **length (Mb)** | **phys. Location (Mb)** | **CRC** | **LM** | **BM** | **Literature** |
| --- | --- | --- | --- | --- | --- | --- | --- | --- |
| 1 | 3p26.3-p22.1 | Gain | 41,898 | 63,410 - 41,961,168 | - | - | **x** | No |
| 1 | 4p16.3-q35.2 | Gain | 190,846 | 69,403 - 190,915,650 | - | - | x | Yes |
| 1 | 5p15.33-q11.2 | Gain | 54,082 | 38,138 - 54,120,346 | x | - | - | Yes |
| 1 | 5p15.33-q11.2 | Gain | 54,289 | 38,138 - 54,326,830 | - | x | - | Yes |
| 1 | 5p15.33-q11.2 | Gain | 54,070 | 38,138 - 54,107,893 | - | - | x | Yes |
| 1 | 6p25.2-p11.1 | Gain | 58,522 | 204,908 - 58,727,179 | - | x | - | Yes |
| 1 | 6q16.3-q21 | Gain | 5,539 | 102,099,978 - 107,638,569 | - | x | - | Yes |
| 1 | 6p25.3-q26 | Gain | 161,600 | 204,908 - 161,804,651 | - | - | x | Yes |
| 1 | 6q26-q27 | Gain | 7,511 | 163,401,853 - 170,913,051 | - | - | x | Yes |
| 1 | 7p22.3-q36.3 | Gain | 159,077 | 41,420 - 159,118,443 | x | - | - | Yes |
| 1 | 7p22.3-q11.21 | Gain | 66,608 | 41,420 - 66,649,301 | - | - | x | Yes |
| 1 | 7q11.22-q36.3 | Gain | 89,554 | 69,564,375-159,118,443 | - | - | x | Yes |
| 1 | 8p12-q11.21 | Gain | 22,337 | 29,406,607 - 51,827,773 | - | - | x | Yes |
| 1 | 8q21.13-q24.3 | Gain | 63,073 | 83,219,271 - 148,292,734 | - | - | x | Yes |
| 1 | 8q11.1-q24.3 | Gain | 99,396 | 46,896,971 - 146,292,734 | x | - | - | Yes |
| 1 | 8q11.23-q24.3 | Gain | 91,012 | 55,280,830 - 146,292,734 | - | x | - | Yes |
| 1 | 10p14-p11.1 | Gain | 28,105 | 11,041,280 - 39,146,676 | - | - | **x** | No |
| 1 | 11q14.1-q25 | Gain | 49,932 | 85,007,025 - 134,938,847 | - | - | x | Yes |
| 1 | 12p13.33-q13.11 | Gain | 47,932 | 189,399 - 48,121,889 | x | - | - | Yes |
| 1 | 12p13.33-q24.33 | Gain | 133,629 | 189,399 - 133,818,115 | - | - | x | Yes |
| 1 | 12p13.31-q12 | Gain | 32,680 | 7,096,280 - 39,776,702 | - | x | - | Yes |
| 1 | 12q21.31-q21.33 | Gain | 7,251 | 82,687,505 - 89,938,590 | - | x | - | Yes |
| 1 | 13q11-q34 | Gain | 96,018 | 19,084,822 - 115,103,150 | x | x | x | Yes (all) |
| 1 | 14q24.2-q32.33 | Gain | 35,894 | 71,387,979 - 107,282,024 | x | - | - | Yes |
| 1 | 14q12-q13.1 | Gain | 5,767 | 29,166,323 - 34,933,810 | - | - | x | Yes |
| 1 | 14q32.12-q32.33 | Gain | 14,439 | 98,389,046 - 106,827,735 | - | - | x | Yes |
| 1 | 15q11.2-q26.3 | Gain | 82,236 | 20,161,371 - 102,397,317 | - | - | **x** | No |
| 1 | 16p12.2-q24.3 | Gain | 66,754 | 23,403,743 - 90,158,005 | - | - | **x** | No |
| 1 | 17p11.2-q24.3 | Gain | 47,773 | 21,138,333 - 68,911,339 | x | - | - | Yes |
| 1 | 17p11.2-q24.3 | Gain | 48,398 | 20,301,296 - 68,699,516 | - | - | x | Yes |
| 1 | 17q25.1-q25.3 | Gain | 5,349 | 74,914,157 - 80,263,427 | - | - | x | Yes |
| 1 | 17q24.1-q24.3 | Gain | 6,226 | 62,617,312 - 68,883,534 | - | **x** | - | No |
| 1 | 19p13.3-q13.43 | Gain | 58,846 | 247,231 - 59,093,239 | x | - | - | Yes |
| 1 | 19p13.2-q13.43 | Gain | 51,595 | 6,943,409 - 58,538,762 | - | x | - | Yes |
| 1 | 19p13.3-p12 | Gain | 23,443 | 274,231 - 23,690,418 | - | - | **x** | No |
| 1 | 20p13-q13.33 | Gain | 62,843 | 69,093 - 62,912,463 | x | - | - | Yes |
| 1 | 20p12.1-q13.33 | Gain | 45,769 | 17,143,374 - 62,912,463 | - | x | - | Yes |
| 1 | 20p13-p12.1 | Gain | 14,371 | 69,093 - 14,440,202 | - | - | x | Yes |
| 1 | 20p12.1-p11.1 | Gain | 10,580 | 15,729,313 - 26,309,255 | - | - | x | Yes |
| 1 | 20q11.21-q13.33 | Gain | 33,393 | 29,519,155 - 62,912,463 | - | - | x | Yes |
| 1 | 5q11.2-q35.3 | Loss | 126,097 | 54,601,451 - 180,698,312 | x | - | - | Yes |
| 1 | 8p23.3-p12 | Loss | 29,297 | 172,416 - 29,469,606 | - | - | x | Yes |
| 1 | 8q21.11-q21.13 | Loss | 7,538 | 75,657,105 - 83,195,460 | - | - | x | Yes |
| 1 | 8p23.3-p12 | Loss | 29,873 | 172,416 - 30,045,554 | x | - | - | Yes |
| 1 | 8p23.3-p12 | Loss | 29,343 | 172,416 - 29,515,831 | - | x | - | Yes |
| 1 | 9p24.3-q34.3 | Loss | 140,850 | 204,737 - 141,654,761 | x | - | - | Yes |
| 1 | 10q11.21-q22.3 | Loss | 34,113 | 45,103,361 - 79,216,839 | - | - | x | Yes |
| 1 | 14q21.2-q24.2 | Loss | 25,288 | 46,079,882 - 71,367,861 | x | - | - | Yes |
| 1 | 14q21.2-q24.2 | Loss | 25,075 | 45,987,004 - 71,061,844 | - | x | - | Yes |
| 1 | 14q21.2-q24.2 | Loss | 25,249 | 46,029,524 - 71,278,113 | - | - | x | Yes |
| 1 | 17p13.3-p11.2 | Loss | 20,721 | 400,958 - 21,122,322 | x | - | - | Yes |
| 1 | 17q24.3-q25.2 | Loss | 6,072 | 68,913,463 - 74,985,472 | x | - | - | Yes |
| 1 | 17p13.3-p13.1 | Loss | 7,223 | 400,958 - 7,624,100 | - | x | - | Yes |
| 1 | 18p11.32-q23 | Loss | 77,995 | 12,841 - 78,007,784 | x | x | - | Yes (all) |
| 1 | 21q11.2-q21.1 | Loss | 9,271 | 14,344,536 - 23,615,318 | x | - | - | Yes |
| 1 | 21q11.2-q21.2 | Loss | 8,865 | 15,206,085 - 24,071,413 | - | - | x | Yes |
| 2 | 2q33.1-q33.3 | Gain | 7,987 | 198,664,026 - 206,615,315 | + | - | - | Yes |
| 2 | 2q33.1-q33.3 | Gain | 8,003 | 198,664,026 - 206,666,625 | - | **x** | x | Yes: BM  No: LM |
| 2 | 5q34-q35.3 | Gain | 16,225 | 164,473,642 - 180,698,312 | x | - | - | Yes |
| 2 | 5q33.3-q35.3 | Gain | 22,349 | 158,348,911 - 180,698,312 | - | **x** | - | No |
| 2 | 5q33.3-q35.3 | Gain | 22,964 | 157,734,761 - 180,698,312 | - | - | x | Yes |
| 2 | 7p22.3-q36.3 | Gain | 159,077 | 41,420 - 159,118,443 | x | x | x | Yes (all) |
| 2 | 8p11.21-q24.3 | Gain | 106,653 | 39,639,410 - 146,292,734 | x | x | x | Yes (all) |
| 2 | 12q11-q24.33 | Gain | 95,915 | 37,902,987 - 133,818,115 | + | **x** | - | Yes: CRC  No: LM |
| 2 | 12q12-q24.33 | Gain | 95,357 | 38,461,563 - 133,818,115 | - | - | x | Yes |
| 2 | 13q12.13-q12.3 | Gain | 5,928 | 25,680,689 - 31,608,475 | - | x | - | Yes |
| 2 | 13q12.13-q34 | Gain | 89,422 | 25,680,689 - 115,103,150 | x | - | - | Yes |
| 2 | 13q11-q34 | Gain | 96,018 | 19,084,822 - 115,103,150 | - | - | x | Yes |
| 2 | 16p13.3 - q24.1 | Gain | 85,230 | 83,886- 85,313,662 | x | - | x | Yes (all) |
| 2 | 16p13.3 - q24.1 | Gain | 85,293 | 83,886 - 85,377,126 | - | x | - | Yes |
| 2 | 20p13-p12.3 | Gain | 6,603 | 69,063 - 6,672,360 | x | x | x | Yes (all) |
| 2 | 20q11.21 - q13.33 | Gain | 33,393 | 29,519,155 - 62,912,463 | x | x | x | Yes (all) |
| 2 | 4p16.3-q35.2 | Loss | 190,846 | 69,403 - 190,915,650 | - | - | x | Yes |
| 2 | 6q27 | Loss | 4,158 | 166,755,479 - 170,913,051 | - | - | x | Yes |
| 2 | 8p23.3-p11.22 | Loss | 39,453 | 172,416 - 39,625,680 | x | x | x | Yes (all) |
| 2 | 9q22.33-q34.3 | Loss | 39,723 | 101,331,391 - 141,054,761 | - | - | **x** | No |
| 2 | 12p13.33-p11.1 | Loss | 34,639 | 189,399 - 34,828,211 | x | x | **x** | Yes: CRC, LM  No: BM |
| 2 | 17p13.3-p11.2 | Loss | 19,819 | 400,958 - 20,220,266 | x | x | - | Yes (all) |
| 2 | 17p13.3-p11.2 | Loss | 19,859 | 400,958 - 20,260,186 | - | - | x | Yes |
| 2 | 18q11.2 - q23 | Loss | 54,417 | 23,590,853 - 78,007,784 | x | - | - | Yes |
| 2 | 18q11.2-q23 | Loss | 54,465 | 23,542,721 - 78,007,784 | - | x | - | Yes |
| 2 | 18q11.2-q23 | Loss | 54,518 | 23,489,649 - 78,007,784 | - | - | x | Yes |
| 2 | 20p12.3 - p11.21 | Loss | 16,076 | 6,681,990 - 22,757,960 | x | x | **x** | Yes: CRC, LM  No: BM |
| 2 | 21q11.2 - q22.3 | Loss | 33,753 | 14,344,536 - 48,097,610 | x | x | x | Yes (all) |
| 3 | 2q23.3-q37.3 | Gain | 89,701 | 153,351,828 - 243,052,331 | - | - | x | Yes |
| 3 | 4q28.3-q31.21 | Gain | 9,175 | 134,768,895 - 143,943,541 | - | x | - | Yes |
| 3 | 5q14.3-q21.1 | Gain | 6,853 | 91,968,414 - 98,821,072 | - | - | **x** | No |
| 3 | 5q33.2-q34 | Gain | 7,873 | 152,866,374 - 160,739,843 | - | - | x | Yes |
| 3 | 5q35.1-q35.3 | Gain | 10,352 | 170,346,170 - 180,698,312 | - | - | x | Yes |
| 3 | 6p21.31-p12.3 | Gain | 15,261 | 34,197,104 - 49,457,835 | - | - | x | Yes |
| 3 | 7p22.3-q36.3 | Gain | 159,077 | 41,420 - 159,118,443 | - | x | x | Yes (all) |
| 3 | 8p12-q24.3 | Gain | 112,810 | 33,482,410 - 146,292,734 | x | x | - | Yes (all) |
| 3 | 8p12-q22.1 | Gain | 62,467 | 33,482,410 - 95,949,013 | - | - | x | Yes |
| 3 | 8q22.3-q24.3 | Gain | 42,842 | 103,451,072 - 146,292,734 | - | - | x | Yes |
| 3 | 10p11.23-p11.21 | Gain | 5,152 | 31,181,786 - 36,334,282 | - | - | **x** | No |
| 3 | 10q21.2-q23.2 | Gain | 25,854 | 62,856,748 - 88,710,415 | - | - | x | Yes |
| 3 | 11q14.3-q25 | Gain | 46,322 | 88,616,936 - 134,938,847 | - | x | - | Yes |
| 3 | 13q11-q34 | Gain | 96,018 | 19,084,822 - 115,103,150 | x | x | x | Yes (all) |
| 3 | 16p13.3-q24.3 | Gain | 90,074 | 83,886 - 90,158,005 | - | - | x | Yes |
| 3 | 17q12-q21.2 | Gain | 6,150 | 34,168,339 - 40,318,296 | - | x | - | Yes |
| 3 | 17q11.1-q22 | Gain | 28,118 | 25,326,940 - 53,445,130 | - | - | x | Yes |
| 3 | 17q23.2-q25.3 | Gain | 21,681 | 58,582,537 - 80,263,427 | - | - | x | Yes |
| 3 | 20q11.21-q13.33 | Gain | 33,393 | 29,519,155 - 62,912,463 | x | x | x | Yes (all) |
| 3 | 1p36.33-q44 | Loss | 248,459 | 754,191 - 249,212,878 | - | x | - | Yes |
| 3 | 1p36.33-p11.2 | Loss | 119,863 | 1,486,833 - 121,349,358 | - | - | x | Yes |
| 3 | 2p25.3-p23.1 | Loss | 31,872 | 21,493 - 31,893,285 | - | - | **x** | No |
| 3 | 3p26.3-q29 | Loss | 197,789 | 63,410 - 197,852,564 | - | - | x | Yes |
| 3 | 3p26.3-p12.3 | Loss | 78,927 | 63,410 - 78,990,607 | - | x | - | Yes |
| 3 | 4p16.3-q35.2 | Loss | 190,846 | 69,403 - 190,915,650 | - | - | x | Yes |
| 3 | 4p13-q13.3 | Loss | 29,750 | 42,112,733 - 71,863,196 | - | x | - | Yes |
| 3 | 4q21.23-q22.2 | Loss | 8,640 | 86,339,955 - 94,979,809 | - | x | - | Yes |
| 3 | 6p25.3-p24.3 | Loss | 7,995 | 204,908 - 8,200,369 | - | x | - | Yes |
| 3 | 8p23.3-p12 | Loss | 29,718 | 172,416 - 29,890,482 | x | - | - | Yes |
| 3 | 8p23.3-p12 | Loss | 29,765 | 172,416 - 29,937,404 | - | x | x | Yes (all) |
| 3 | 9p24.3-p13.2 | Loss | 36,674 | 204,737 - 36,878,774 | - | x | - | Yes |
| 3 | 9q21.11-q34.3 | Loss | 70,070 | 70,984,371 - 141,054,761 | - | x | - | Yes |
| 3 | 10q21.1-q21.2 | Loss | 5,576 | 57,549,177 - 63,124,768 | x | - | - | Yes |
| 3 | 10q21.1-q21.2 | Loss | 5,324 | 57,669,026 - 62,993,255 | - | x | - | Yes |
| 3 | 10q23.33-q26.3 | Loss | 38,606 | 96,828,322 - 135,434,303 | - | x | - | Yes |
| 3 | 10p15.3-p11.23 | Loss | 31,047 | 126,069 - 31,172,920 | - | - | **x** | No |
| 3 | 10q21.1-q21.2 | Loss | 4,587 | 58,250,200 - 62,837,057 | - | - | x | Yes |
| 3 | 10q23.33-q26.3 | Loss | 37,472 | 96,828,322 - 134,300,069 | - | - | x | Yes |
| 3 | 12p13.33-q24.33 | Loss | 133,629 | 189,399 - 133,818,115 | - | - | x | Yes |
| 3 | 14q11.2-q32.33 | Loss | 87,063 | 20,219,082 - 107,282,024 | - | - | x | Yes |
| 3 | 15q11.2-q26.3 | Loss | 79,122 | 22,752,398 - 101,874,841 | - | x | - | Yes |
| 3 | 15q11.2-q26.3 | Loss | 79,645 | 22,752,398 - 102,397,317 | x | - | - | Yes |
| 3 | 17p13.3-p11.2 | Loss | 20,863 | 400,958 - 21,264,396 | x | - | - | Yes |
| 3 | 17p13.3-p11.2 | Loss | 20,646 | 400,958 - 21,047,073 | - | x | - | Yes |
| 3 | 17q21.31-q24.3 | Loss | 29,128 | 41,470,682 - 70,598,901 | - | x | - | Yes |
| 3 | 17p13.3-p11.2 | Loss | 20,780 | 400,958 - 21,180,726 | - | - | x | Yes |
| 3 | 18q11.1-q23 | Loss | 59,453 | 18,554,306 - 78,007,784 | x | x | x | Yes (all) |
| 3 | 19p13.3-q13.43 | Loss | 58,824 | 247,231 - 59,071,321 | - | x | - | Yes |
| 3 | 19p13.3-q13.43 | Loss | 58,846 | 247,231 - 59,093,239 | - | - | **x** | No |
| 3 | 20p13-p11.1 | Loss | 26,240 | 69,093 - 26,309,255 | x | - | - | Yes |
| 3 | 20p13-p11.1 | Loss | 26,036 | 69,093 - 26,105,444 | - | x | - | Yes |
| 3 | 20p13-p11.1 | Loss | 26,082 | 69,093 - 26,151,507 | - | - | **x** | No |
| 3 | 21q21.2-q21.3 | Loss | 4,594 | 24,362,833 - 28,956,641 | x | - | - | Yes |
| 3 | 21q11.2-q22.3 | Loss | 33,753 | 14,344,536 - 48,097,610 | - | - | x | Yes |
| 3 | 22q11.1-q13.33 | Loss | 34,351 | 16,863,069 - 51,213,826 | - | x | x | Yes (all) |
| 4 | 1q21.1-q41 | Gain | 77,144 | 145,676,265 - 222,820,639 | x |  | - | Yes |
| 4 | 1p31.3 | Gain | 4,477 | 62,798,104 - 67,275,027 | - |  | x | Yes |
| 4 | 1q21.1-q25.2 | Gain | 32,260 | 144,009,052 - 176,268,606 | - |  | x | Yes |
| 4 | 1q31.3-q41 | Gain | 26,336 | 196,438,948 - 222,775,337 | - |  | x | Yes |
| 4 | 2p23.1-p14 | Gain | 33,806 | 30,686,944 - 64,492,516 | x |  | - | Yes |
| 4 | 2q11.1-q13 | Gain | 16,023 | 95,429,196 - 111,451,816 | x |  | - | Yes |
| 4 | 2q22.1-q24.3 | Gain | 25,367 | 142,005,959 - 167,372,934 | x |  | - | Yes |
| 4 | 2q32.3-q37.3 | Gain | 47,094 | 195,958,752 - 243,052,331 | x |  | - | Yes |
| 4 | 2p23.1-p16.2 | Gain | 23,540 | 30,695,785 - 54,235,341 | - |  | x | Yes |
| 4 | 2p16.1-p15 | Gain | 6,529 | 56,701,656 - 63,230,734 | - |  | x | Yes |
| 4 | 2q11.1-q13 | Gain | 15,041 | 95,429,196 - 110,470,543 | - |  | x | Yes |
| 4 | 2q22.3-q31.1 | Gain | 29,870 | 144,332,853 - 174,203,179 | - |  | x | Yes |
| 4 | 2q32.3-q37.3 | Gain | 47,072 | 195,980,759 - 243,052,331 | - |  | x | Yes |
| 4 | 3q23-q29 | Gain | 55,949 | 141,903,904 - 197,852,564 | x |  | - | Yes |
| 4 | 3p14.1-q29 | Gain | 132,402 | 65,450,719 - 197,852,564 | - |  | x | Yes |
| 4 | 4p15.33-p11 | Gain | 36,245 | 12,847,074 - 49,092,454 | - |  | **x** | No |
| 4 | 5p15.33-p11 | Gain | 42,624 | 3,777,393 - 46,401,271 | - |  | x | Yes |
| 4 | 6p25.3-q16.1 | Gain | 95,383 | 204,908 - 95,587,807 | x |  | - | Yes |
| 4 | 6q21-q27 | Gain | 64,895 | 106,017,975 - 170,913,051 | x |  | - | Yes |
| 4 | 6p25.3-p21.31 | Gain | 36,193 | 204,908 - 36,398,302 | - |  | x | Yes |
| 4 | 6p21.2-q15 | Gain | 52,436 | 37,473,197 - 89,909,557 | - |  | x | Yes |
| 4 | 6q21-q27 | Gain | 64,838 | 106,075,246 - 170,913,051 | - |  | x | Yes |
| 4 | 7p15.3-q22.3 | Gain | 82,554 | 23,996,766 - 106,550,661 | x |  | - | Yes |
| 4 | 7p15.3-q33 | Gain | 109,810 | 23,996,766 - 133,807,169 | - |  | x | Yes |
| 4 | 8p11.1-q24.3 | Gain | 102,525 | 43,767,533 - 146,292,734 | x |  | - | Yes |
| 4 | 8q11.1-q24.3 | Gain | 99,396 | 46,896,971 - 146,292,734 | - |  | x | Yes |
| 4 | 9p21.3-21.1 | Gain | 6,176 | 22,445,891 - 28,631,797 | - |  | x | Yes |
| 4 | 9q31.2-q34.3 | Gain | 29,955 | 111,099,675 - 141,054,761 | - |  | x | Yes |
| 4 | 10p11.23-q11.21 | Gain | 12,803 | 29,717,838 - 42,521,092 | **x** |  | - | No |
| 4 | 10q22.1-q22.3 | Gain | 3,429 | 74,894,374 - 78,322,922 | x |  | - | Yes |
| 4 | 10q24.31-q26.3 | Gain | 32,571 | 102,862,926 - 135,434,303 | **x** |  | - | No |
| 4 | 10p11.23-p11.1 | Gain | 9,401 | 29,746,084 - 39,146,676 | - |  | **x** | No |
| 4 | 10q22.1-q22.3 | Gain | 3,876 | 74,446,791 - 78,322,922 | - |  | x | Yes |
| 4 | 10q24.31-q26.3 | Gain | 31,184 | 102,862,926 - 134,046,682 | - |  | **x** | No |
| 4 | 11q13.2-q14.1 | Gain | 10,957 | 67,457,094 - 78,414,040 | - |  | x | Yes |
| 4 | 12p13.33-p12.1 | Gain | 23,637 | 189,399 - 23,826,008 | x |  | - | Yes |
| 4 | 12p13.33-p12.1 | Gain | 23,945 | 189,399 - 24,134,126 | - |  | x | Yes |
| 4 | 13q11-q13.2 | Gain | 15,481 | 19,084,822 - 34,565,411 | x |  | x | Yes (all) |
| 4 | 13q14.3-q34 | Gain | 61,852 | 53,250,838 - 115,103,150 | x |  | - | Yes |
| 4 | 13q14.3-q32.3 | Gain | 47,097 | 53,567,016 - 101,473,830 | - |  | x | Yes |
| 4 | 14q11.2-q24.1 | Gain | 49,484 | 20,219,082 - 69,703,158 | - |  | **x** | No |
| 4 | 14q32.12-q32.33 | Gain | 14,436 | 92,846,293 - 107,282,024 | - |  | **x** | No |
| 4 | 15q11.2-q15.1 | Gain | 15,423 | 24,982,192 - 40,404,898 | **x** |  | - | No |
| 4 | 15q21.2-q21.3 | Gain | 4,615 | 50,874,631 - 55,489,412 | x |  | **x** | Yes: CRC  No: BM |
| 4 | 15q25.2-q26.3 | Gain | 19,177 | 83,220,098 - 102,397,317 | x |  | - | Yes |
| 4 | 15q11.2-q21.1 | Gain | 23,027 | 22,752,398 - 45,779,810 | - |  | **x** | No |
| 4 | 15q24.2-q26.3 | Gain | 26,893 | 75,504,553 - 102,397,317 | - |  | **x** | No |
| 4 | 16p13.3-p11.1 | Gain | 35,188 | 83,886 - 35,271,725 | - |  | x | Yes |
| 4 | 17q11.1-q21.2 | Gain | 13,304 | 25,326,940 - 38,630,459 | - |  | x | Yes |
| 4 | 18p11.32-q21.1 | Gain | 43,858 | 12,841 - 43,870,742 | x |  | - | Yes |
| 4 | 18q22.2-q23 | Gain | 10,112 | 67,895,606 - 78,007,784 | x |  | - | Yes |
| 4 | 18p11.32-p11.21 | Gain | 15,365 | 12,841 - 15,377,471 | - |  | **x** | No |
| 4 | 18q11.1-q21.1 | Gain | 25,541 | 18,554,306 - 44,095,365 | - |  | **x** | No |
| 4 | 18q21.33-q23 | Gain | 17,902 | 60,105,388 - 78,007,784 | - |  | **x** | No |
| 4 | 20p13-q13.33 | Gain | 62,843 | 69,093 - 62,912,463 | x |  | x | Yes (all) |
| 4 | 1p32.3 - 1p31.3 | Loss | 11,702 | 51,086,336 - 62,788,511 | - |  | x | Yes |
| 4 | 1p31.3-p12 | Loss | 53,186 | 67,283,438 - 120,469,147 | - |  | x | Yes |
| 4 | 2p25.3-p23.1 | Loss | 30,538 | 21,493 - 30,559,816 | x |  | - | Yes |
| 4 | 2q13-q22.3 | Loss | 33,324 | 110,861,853 - 144,186,113 | - |  | **x** | No |
| 4 | 3p26.3-p14.1 | Loss | 65,379 | 63,410 - 65,442,138 | - |  | x | Yes |
| 4 | 4p16.3-p15.33 | Loss | 12,769 | 69,403 - 12,838,429 | - |  | x | Yes |
| 4 | 4q23-q25 | Loss | 8,403 | 100,504,984 - 108,907,832 | - |  | x | Yes |
| 4 | 4q32.3-q35.2 | Loss | 20,637 | 167,876,113 - 188,512,818 | - |  | x | Yes |
| 4 | 5p15.33 | Loss | 3,723 | 38,138 - 3,760,907 | - |  | x | Yes |
| 4 | 5q11.1-q31.3 | Loss | 91,605 | 49,441,965 - 141,046,936 | - |  | x | Yes |
| 4 | 6q16.1-q21 | Loss | 12,212 | 93,845,632 - 106,057,283 | - |  | x | Yes |
| 4 | 8p21.2-p11.1 | Loss | 17,024 | 26,743,932 - 43,767,534 | - |  | x | Yes |
| 4 | 9p24.3-p21.3 | Loss | 22,241 | 204,737 - 22,445,831 | - |  | x | Yes |
| 4 | 9p21.1 | Loss | 3,247 | 28,647,748 - 31,894,764 | - |  | x | Yes |
| 4 | 9q21.11-q31.2 | Loss | 40,102 | 70,984,371 - 111,086,396 | - |  | **x** | No |
| 4 | 10q22.3-q24.31 | Loss | 24,512 | 78,342,390 - 102,854,664 | x |  | x | Yes (all) |
| 4 | 10p15.2-p11.23 | Loss | 26,507 | 3,224,808 - 29,731,586 | - |  | **x** | No |
| 4 | 11p15.5-p12 | Loss | 38,541 | 192,763 - 38,733,945 | - |  | **x** | No |
| 4 | 11q14.1-q25 | Loss | 56,510 | 78,428,817 - 134,938,847 | - |  | **x** | No |
| 4 | 13q32.3-q34 | Loss | 13,616 | 101,487,500 - 115,103,150 | - |  | x | Yes |
| 4 | 16q23.1-q24.1 | Loss | 5,780 | 78,472,700 - 84,253,029 | x |  | - | Yes |
| 4 | 16q23.1-q24.3 | Loss | 11,903 | 78,254,731 - 90,158,005 | - |  | x | Yes |
| 4 | 18q21.1-q21.2 | Loss | 23,964 | 43,880,954 - 67,844,534 | x |  | - | Yes |
| 4 | 18q21.1-q21.33 | Loss | 15,981 | 44,117,577 - 60,098,545 | - |  | x | Yes |
| 4 | 21p11.2-q22.3 | Loss | 38,449 | 9,648,314 - 48,097,610 | - |  | x | Yes |
